# Convergent use of aminopeptidase N receptor by hedgehog merbecoviruses

**DOI:** 10.1038/s41564-026-02478-1

**Published:** 2026-09-16

**Authors:** Peng Liu, Qianhui Zhu, Jingyi Liu, Zhixing Ma, Guocui Mou, Yuanling Yu, Chen Liu, Chengbao Ma, Junyu Si, Xiao Yang, Meiling Huang, Yanchu Liu, Yehui Sun, Xiao Yu, Yucheng Sun, Tianci Liu, Lingling Yu, Qianran Wang, Honghao Ma, Qi Lv, Jing Li, Linlin Bao, Yunlong Cao, Xiangxi Wang, Huan Yan

**Affiliations:** 1 State Key Laboratory of Virology and Biosafety, Hubei Provincial Research Center for Basic Biological Sciences, College of Life Sciences, TaiKang Center for Life and Medical Sciences, Wuhan University, Wuhan, China; 2 Key Laboratory of Biomacromolecules (CAS), National Laboratory of Biomacromolecules, CAS Center for Excellence in Biomacromolecules, Institute of Biophysics, Chinese Academy of Sciences, Beijing, China; 3 University of Chinese Academy of Sciences, Beijing, China; 4 Biomedical Pioneering Innovation Center (BIOPIC), Peking University, Beijing, China; 5 Changping Laboratory, Beijing, China; 6 College of Future Technology, Peking University, Beijing, China; 7 Institute of Laboratory Animal Science, Chinese Academy of Medical Sciences (CAMS) & Peking Union Medical College (PUMC), Beijing, China; 8 Peking-Tsinghua Center for Life Sciences, Tsinghua University, Beijing, China

**Keywords:** Virus-host interactions, Virus structures

## Abstract

Coronaviruses circulating in wildlife, such as European and Asian hedgehog merbecoviruses (EriCoVs), pose a potential transmission risk. Knowledge of viral receptors can inform risk assessment and development of countermeasures; however, the functional receptor for EriCoVs is unknown. Here we identify hedgehog aminopeptidase N (APN) as a functional receptor for various EriCoVs. Analysis of APN orthologues from 93 species reveals a restricted APN tropism for EriCoVs, primarily utilizing APN from hedgehogs and to a lesser extent felid APN. Cryogenic electron microscopy analysis of the EriCoV–APN complex reveals an APN-binding mode that is distinct from those used by alpha- and deltacoronaviruses. Functional assays suggest that proteolytic activation of EriCoV spikes can be facilitated by multiple proteases in a context- and strain-dependent manner. Neutralizing antibodies targeting the EriCoV receptor-binding domain and hedgehog APN effectively blocked EriCoV pseudovirus entry or amplification. Receptor-binding-domain-targeting antibodies were further elucidated by cryogenic electron microscopy analysis. Together, these findings can inform assessment of merbecovirus spillover and pathogenesis.

## Abstract (web summary)

Hedgehog merbecoviruses are shown to use aminopeptidase N as a functional receptor through a distinct binding mode, revealing convergent receptor usage among merbecoviruses.

## Main

Several bat-borne merbecoviruses, including strains belonging to *Betacoronavirus cameli* (MERSr-CoV), *Betacoronavirus pipistrelli* (HKU5) or *Betacoronavirus tylonycteridis* (HKU4) have spilled over into humans^1^, camels^2^, pangolins^3^ or minks^4^. In 2013, a new merbecovirus species, *Betacoronavirus erinacei* (EriCoV/ErinCoV), was discovered in European hedgehogs (*Erinaceus europaeus*) in Germany^5^, and a related Asian lineage (HKU31) was subsequently found in Amur hedgehogs (*Erinaceus amurensis*) in China^6^. Until now, EriCoVs have been reported exclusively in hedgehogs with high prevalence and genetic diversity across Eurasia^7–13^. Most viral sequences have been detected in faecal or rectal samples, consistent with enteric tropism and a likely faecal–oral transmission route, although available studies have not demonstrated a clear association between infection and animal health^9,14^. So far, no authentic EriCoV isolate has been reported, and the pathogenicity, host range and zoonotic potential of these viruses remain poorly understood.

Coronavirus host range and transmissibility are primarily determined by interactions between viral receptor-binding domains (RBDs) and cellular receptors^15^. To date, seven proteins have been established as functional coronavirus receptors: carcinoembryonic antigen-related cell adhesion molecule 1a (CEACAM1a)^16^, angiotensin-converting enzyme 2 (ACE2)^17–19^, dipeptidyl peptidase-4 (DPP4)^20^, aminopeptidase N (APN)^21^, transmembrane protease serine 2 (TMPRSS2)^22^, dipeptidase 1 (DPEP1)^23^ and carcinoembryonic antigen-related cell adhesion molecule 6 (CEACAM6)^24^. Among these, the use of both ACE2 and APN has been independently acquired by diverse coronaviruses with distinct RBDs, highlighting recurrent convergent evolution in receptor usage^25–27^. Although DPP4 has long been considered a canonical receptor for merbecoviruses, recent studies have revealed that several merbecovirus lineages co-opted ACE2 usage independently^17,25,26,28–30^. However, EriCoVs do not use either ACE2 or DPP4 for entry, leaving their receptor usage and transmission mechanism unresolved^17,28^; this represents a critical gap in our understanding of receptor evolution in this subgenus.

Here we show that EriCoVs use hedgehog APN as a primary entry receptor through a previously unrecognized APN-binding mode. Our finding resolves the long-standing question of EriCoV receptor usage, reveals convergent evolution of APN utilization in merbecoviruses and provides a framework for assessing the zoonotic potential of these widespread merbecoviruses.

## Results

### Hedgehog APN is a receptor for EriCoVs

**Fig. 1:**
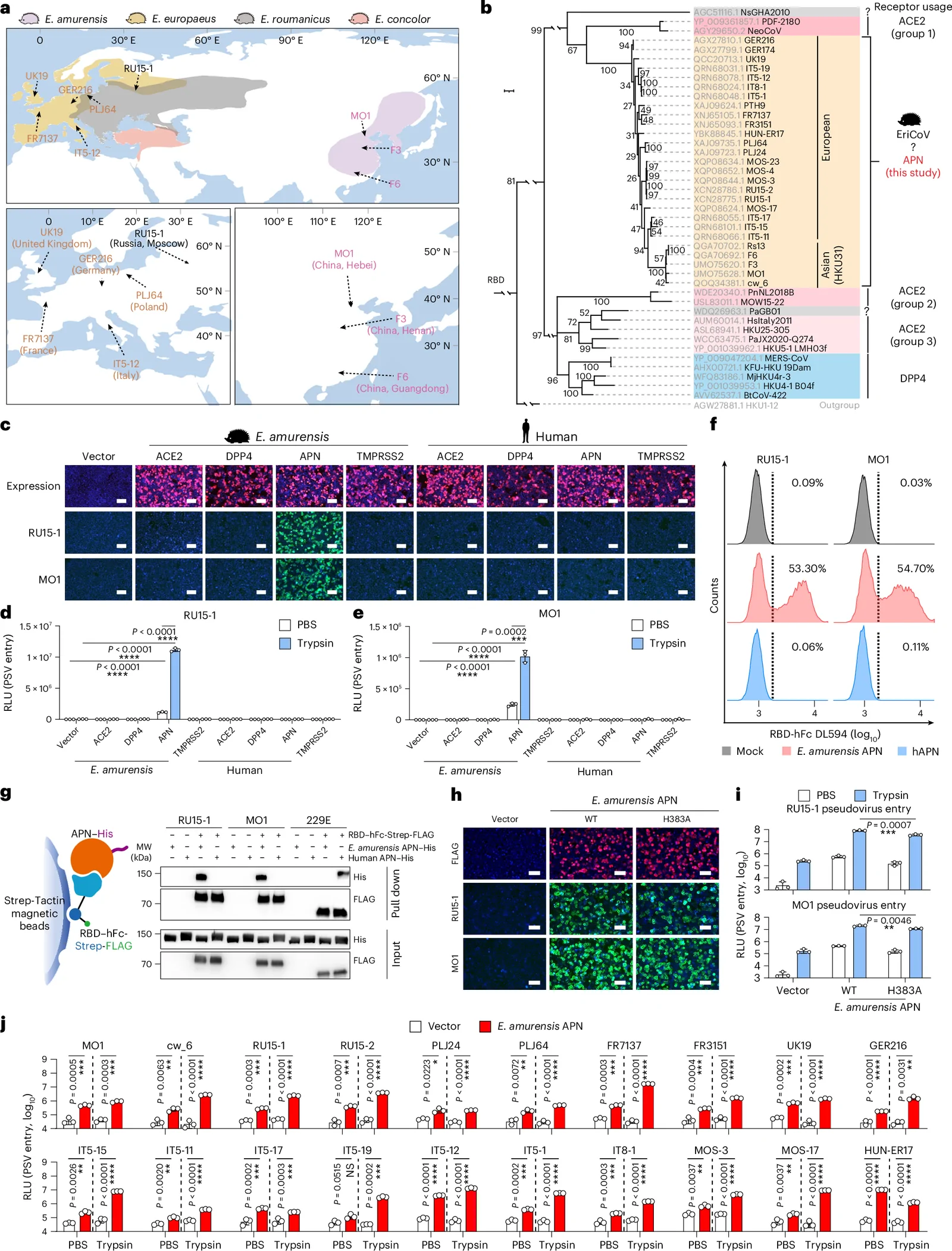
Hedgehog APN serves as a receptor for diverse EriCoV strains. **a**, Geographic distribution of *Erinaceus* hedgehogs and reported sampling locations of selective EriCoV strains. Species distribution data retrieved from the International Union for Conservation of Nature Red List were mapped using GeoScene Pro. RU15-1 was detected in an unconfirmed hedgehog species. **b**, Maximum-likelihood phylogenetic tree of representative merbecovirus RBD amino acid sequences were generated by IQ-TREE. Receptor usage modes are annotated. **c**–**e**, Transiently expressed *E.* *amurensis* APN, but not other receptors, supported EriCoV RU15-1 and MO1 RBD–human IgG1 Fc (RBD–hFc) binding (green) assessed by live-cell immunofluorescence (**c**), as well as VSV-based pseudovirus entry of RU15-1 (**d**) and MO1 (**e**) in HEK293T cells. Receptor expression (red) was validated by immunofluorescence. **f**, Flow cytometry analyses of RU15-1 and MO1 RBD–hFc binding to HEK293T cells transiently expressing human or *E.* *amurensis* APNs. Dashed lines indicate background threshold. **g**, Pull-down assay assessing interaction of soluble human or *E.* *amurensis* APN with immobilized RU15-1 or MO1 RBD–hFc. 229E was included as a control. Data are representative of two independent pull-down experiments with similar results. **h**,**i**, RU15-1 and MO1 RBD–hFc binding (green) (**h**) and pseudovirus entry (**i**) in HEK293T cells transiently expressing wild-type (WT) *E.* *amurensis* APN or its catalytic mutant (H383A). **j**, Trypsin-pretreated EriCoV pseudovirus entry in HEK293T cells transiently expressing the *E.* *amurensis* APN. VSV-based pseudoviruses were pretreated with PBS or 1.25 mg ml^−1^ trypsin for **d**,**e**,**i**,**j**. Mean ± s.d. and *n* = 3 independently infected wells for **d**,**e**,**i**,**j**. Statistical significance was determined using unpaired two-tailed *t*-tests. *P < 0.05, **P < 0.01, ***P < 0.001, ****P < 0.0001; NS, not significant. Representative immunofluorescence images in **c**,**h** are shown. Two independent experiments were performed with similar results. Scale bars, 0.1 substitutions per site (**b**), 100 μm (**c**,**h**). PSV, pseudovirus; RLU, relative light units. Source data

To investigate EriCoV receptor usage, we first analysed publicly available EriCoV genomes and S glycoprotein (S) sequences, with particular focus on the RBD regions (Supplementary Table 1, with strain abbreviations). Several Asian strains (HKU31) were identified in Amur hedgehogs (*E.* *amurensis*) from China, whereas most European strains were identified in European hedgehogs (*E.* *europaeus*) from various European countries (Fig. 1a).

**Extended Data Fig. 1:**
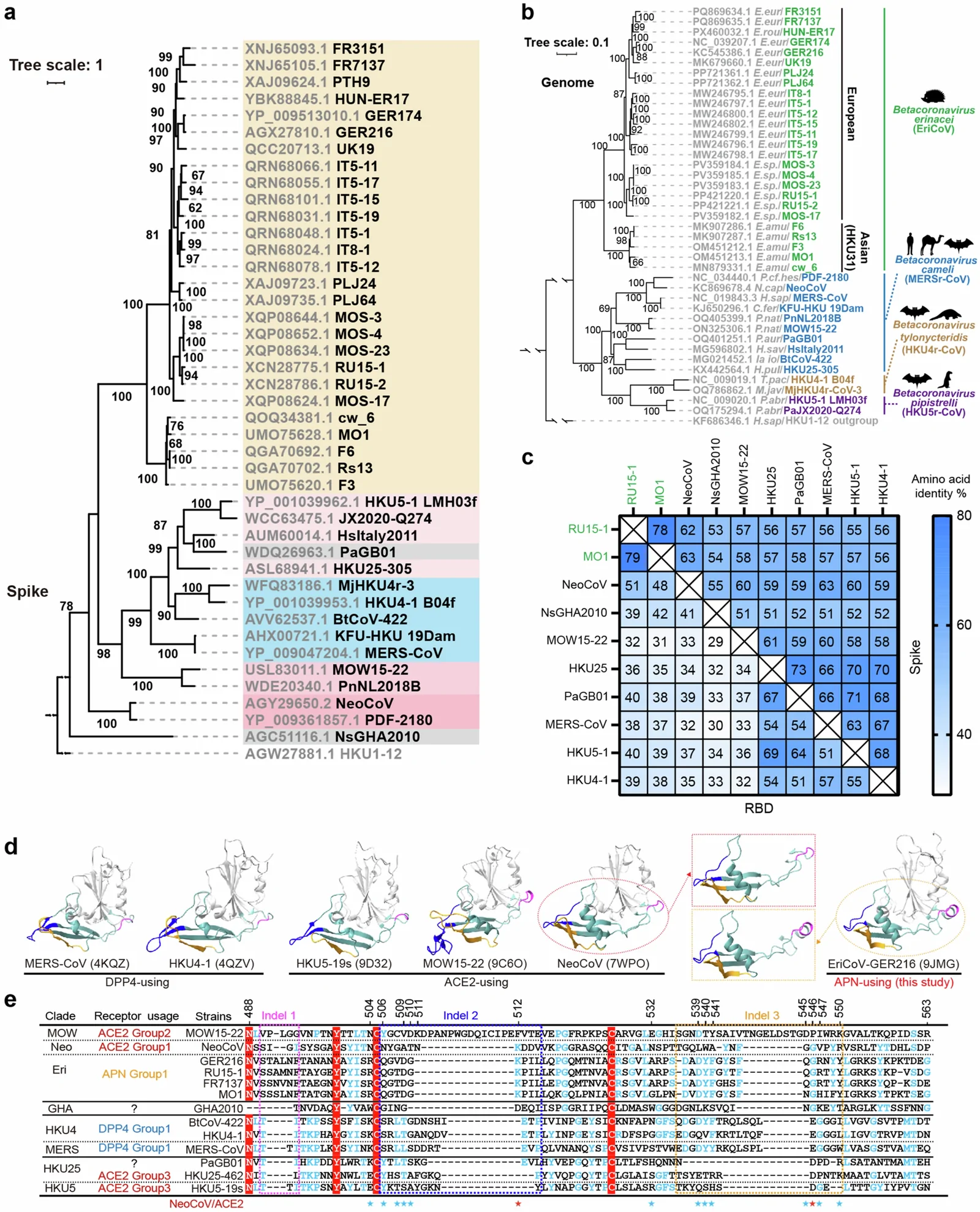
Phylogeny and receptor-binding domain features of EriCoVs. (**a**) Phylogenetic trees based on amino acid sequences of S glycoproteins from representative merbecoviruses were generated using IQ-TREE. Outgroup: HKU1-12. (**b**) Maximum-likelihood phylogenetic tree of representative merbecoviruses based on complete genome sequences. (**c**) Pairwise amino acid identity matrix of RBDs and S glycoproteins of selected merbecoviruses. EriCoV strains were labeled in green. (**d**) Structures of representative merbecovirus RBDs. The conserved RBD core subdomains are rendered in gray and RBMs in dusty teal; structurally divergent RBM indels are highlighted (magenta: indel 1; dark blue: indel 2; orange: indel 3). The magnified RBMs from NeoCoV and EriCoV (GER216) were shown for comparison. (**e**) Manually adjusted multiple sequence alignment of RBMs with optimized indel positioning. Fully conserved residues are shown with a red background; partially conserved residues are colored with light blue. Dashed boxes define indels that correspond with panel **e**. Residues mediating ACE2 interactions by NeoCoV are asterisked (red: conserved in EriCoVs; blue: divergent). NeoCoV residue numbering is shown.

Phylogenetic analyses of genome and S sequences showed that Asian and European EriCoVs form sister lineages, distant from bat-borne merbecoviruses, indicating long-term divergence in hedgehogs across Eurasia (Extended Data Fig. 1a,b). Notably, EriCoV RBD sequences cluster with those of ACE2-using NeoCoV and PDF-2180, implying a potential shared ancestry (Fig. 1b)^17^. EriCoV strains RU15-1 and MO1 share moderate S and RBD amino acid sequence identity (78–79%), but both are markedly divergent from other merbecoviruses (31–51% for RBD and 53–63% for S) (Extended Data Fig. 1c).

Structural comparison revealed that EriCoV and NeoCoV share similar RBD architecture, including characteristic insertion–deletion (indel) patterns, which have been associated with coronavirus receptor usage (Extended Data Fig. 1d)^31^. However, only 2 out of 14 ACE2-interacting NeoCoV residues are conserved in EriCoV, suggesting distinct receptor usage (Extended Data Fig. 1e).

Because previous attempts failed to demonstrate ACE2 usage for EriCoVs^17^, we further examined whether EriCoVs utilize alternative known coronavirus receptors. Sequences of ACE2, DPP4, TMPRSS2 and APN were retrieved from *E. amurensis* RNA-seq datasets (Genome Sequence Archive (GSA) database). Protein-coding sequences of *E.* *amurensis* turned out to be identical to *E.* *europaeus* APN, allowing *E.* *amurensis* APN to represent both species (Supplementary Table 2).

**Extended Data Fig. 2:**
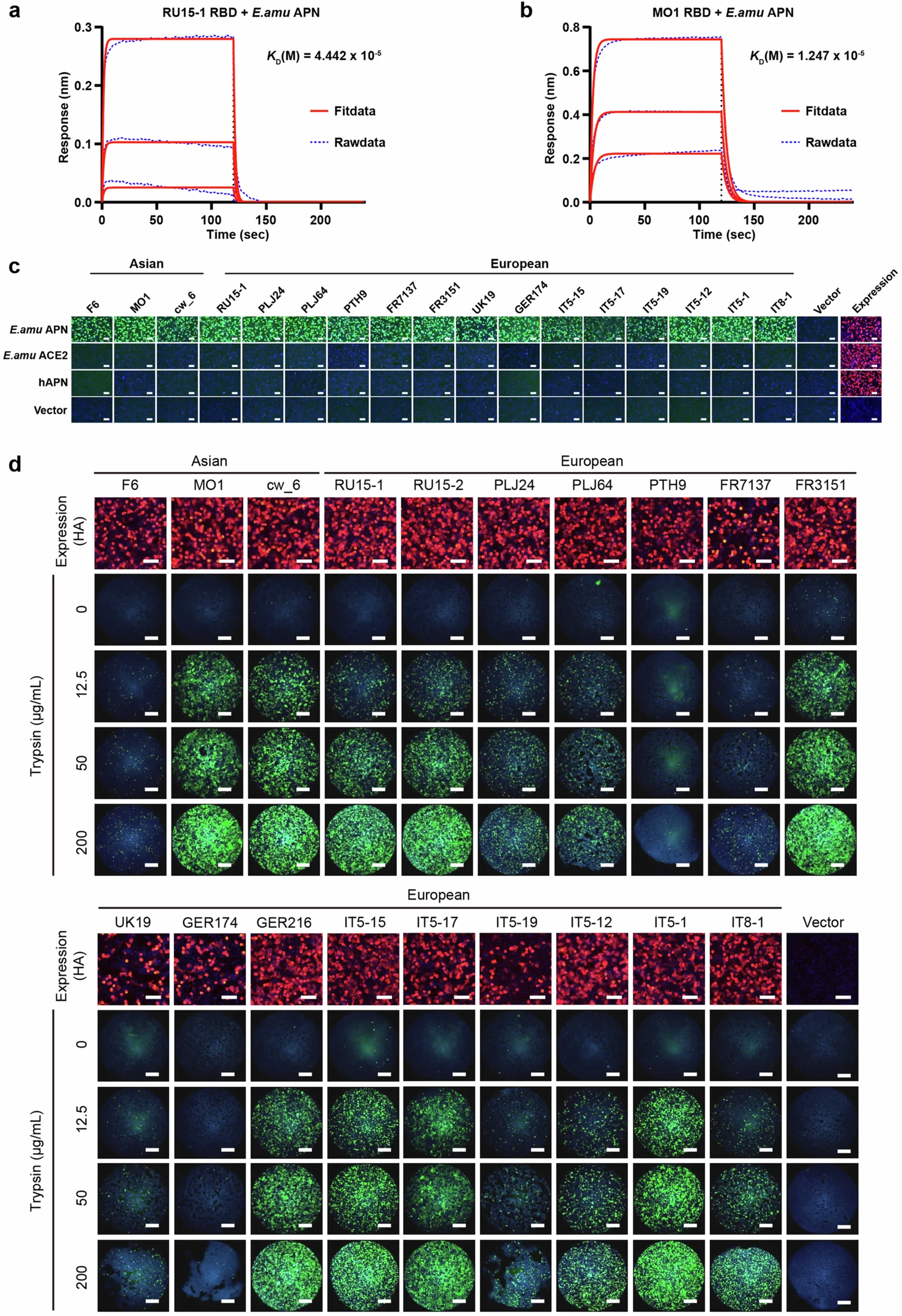
*E.amu* APN-mediated binding of EriCoV RBD-hFc and spike-mediated cell–cell fusion. (**a-b**) BLI analysis of the binding kinetics of *E.amu* APN ectodomains to immobilized monomeric RU15-1 (a) and MO1 (b) RBD-His proteins on Ni-NTA biosensors. The BLI assay was performed using a 1:1 binding model with fixed-phase and mobile-phase interactions. Data were analyzed by global fitting, and the equilibrium dissociation constant (K_D_) was derived from the ratio of the dissociation rate (kd) to the association rate (ka). The fits are shown as red lines. (**c**) RBD–hFc binding of indicated EriCoV strains in HEK293T cells transiently expressing *E.amu* APN, *E.amu* ACE2, human APN (hAPN), or vector control. Receptor expression was evaluated by FLAG-tag immunofluorescence (red). (**d**) S glycoprotein (red) mediated cell–cell fusion (green) of indicated EriCoV strains in HEK293T-*E.amu* APN stable cells following trypsin treatment (37°C for 30 min and quenched by medium with 10% FBS). Representative images from two independent RBD-binding experiments (green in panel **c**) and two independent cell-cell fusion experiments (green in panel **d**) with similar results are shown. Scale bars: 100 μm for c and d (expression, red) and 5 mm for d (fusion, green).

We first evaluated two representative EriCoV strains: RU15-1 (European) and MO1 (Asian). *E.* *amurensis* APN, but not other tested receptors, supported detectable RBD binding (Fig. 1c) and viral entry evaluated by vesicular stomatitis virus (VSV) pseudoviruses (Fig. 1d,e). Flow cytometry and pull-down assays confirmed specific EriCoV RBD binding to *E.* *amurensis* APN but not to human APN (Fig. 1f,g). Biolayer interferometry (BLI) assays displayed relatively low binding affinity and rapid dissociation of soluble *E.* *amurensis* APN binding to immobilized RU15-1 or MO1 RBD (Extended Data Fig. 2a,b). Mutation of the APN catalytic site H383A^32^ only slightly reduced RBD binding or pseudovirus entry, indicating that receptor function is independent of peptidase activity (Fig. 1h,i).

Consistently, *E.* *amurensis* APN, but not *E.* *amurensis* ACE2 or human APN, supported RBD binding from diverse strains tested (Extended Data Fig. 2c). *E.* *amurensis* APN also promoted spike-mediated cell–cell fusion for most strains, especially upon trypsin treatment for proteolytic activation^33,34^ (Extended Data Fig. 2d and Supplementary Fig. 1a). *E.* *amurensis* APN usage and trypsin dependence were further validated using pseudovirus entry of diverse EriCoV strains (Fig. 1j and Supplementary Fig. 1b).

Taken together, hedgehog APN serves as a functional receptor for both Asian and European EriCoVs through specific interaction with their RBDs.

### Spillover risk and host tropism determinants

**Fig. 2:**
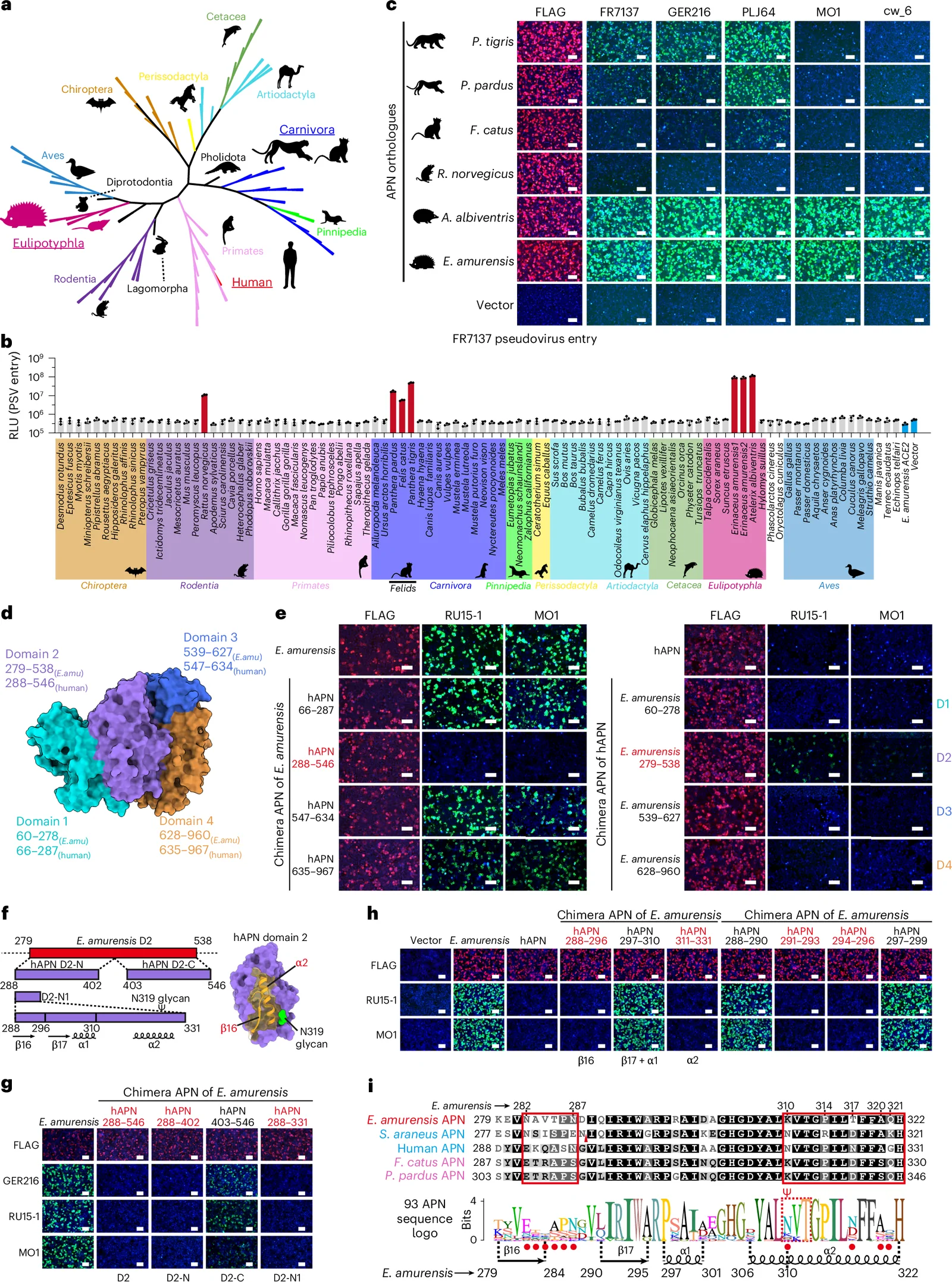
Multispecies APN tropism and host-range determination of EriCoVs. **a**, Maximum-likelihood phylogenetic tree generated using IQ-TREE based on amino acid sequences of 93 APN orthologues (Supplementary Table 3). Taxonomic orders were annotated. **b**, FR7137 pseudovirus entry in HEK293T cells transiently expressing 93 APN orthologues. Data are representative of two independent pseudovirus entry experiments with similar results. **c**, Binding (green) of RBD–hFc from selected EriCoV strains to HEK293T cells transiently expressing the indicated APN orthologues (red). **d**, Human APN structure showing corresponding human/*E.* *amurensis* boundaries of domains 1–4. **e**, EriCoV RBD–hFc binding to HEK293T cells transiently expressing APN chimeras with domain swaps between human and *E.* *amurensis* APNs. Colour coding of domains (D) is the same as in **d**. **f**, Schematic illustration of *E.* *amurensis* APN chimeras with equivalent residues from human APN. Surface localization of β16 and α2 was highlighted. **g**,**h**, Fine mapping of determinants restricting human APN binding with RU15-1 and MO1 RBDs. *E. amurensis* APN chimeras were generated by replacing the corresponding regions with the indicated regions of human APN within residues 288–546 (**g**) or 288–331 (**h**). Notable phenotypic changes are highlighted (red). **i**, Sequence alignment of APN from selected species and logoplot of 93 APN orthologues. Sites with gaps in >95% of the 93 APN sequences were hidden in the logoplot for clarity. The red triangle indicates a hidden lysine (K) in *S.araneus* APN. Key secondary structures and predicted host-range determinants (according to **g**,**h** and Extended Data Fig. 4c,d) are marked with red dots. Red boxes indicate predicted hot-spot regions for host-range determination. Red dashed box indicates glycosylation sequon. Ψ indicates N-glycosylation. Pseudoviruses in **b** were pretreated with 1.25 mg ml^−1^ trypsin. APN expression (red in **c**,**e**,**g**,**h**) was validated via FLAG immunofluorescence. The immunofluorescence of RBD-binding (green in **c**,**e**,**g**,**h**) shown is representative of three independent experiments with similar results. Data are presented as mean ± s.d. (*n* = 3 independently infected wells) for **b**. Scale bars, 100 μm. Source data

**Extended Data Fig. 3:**
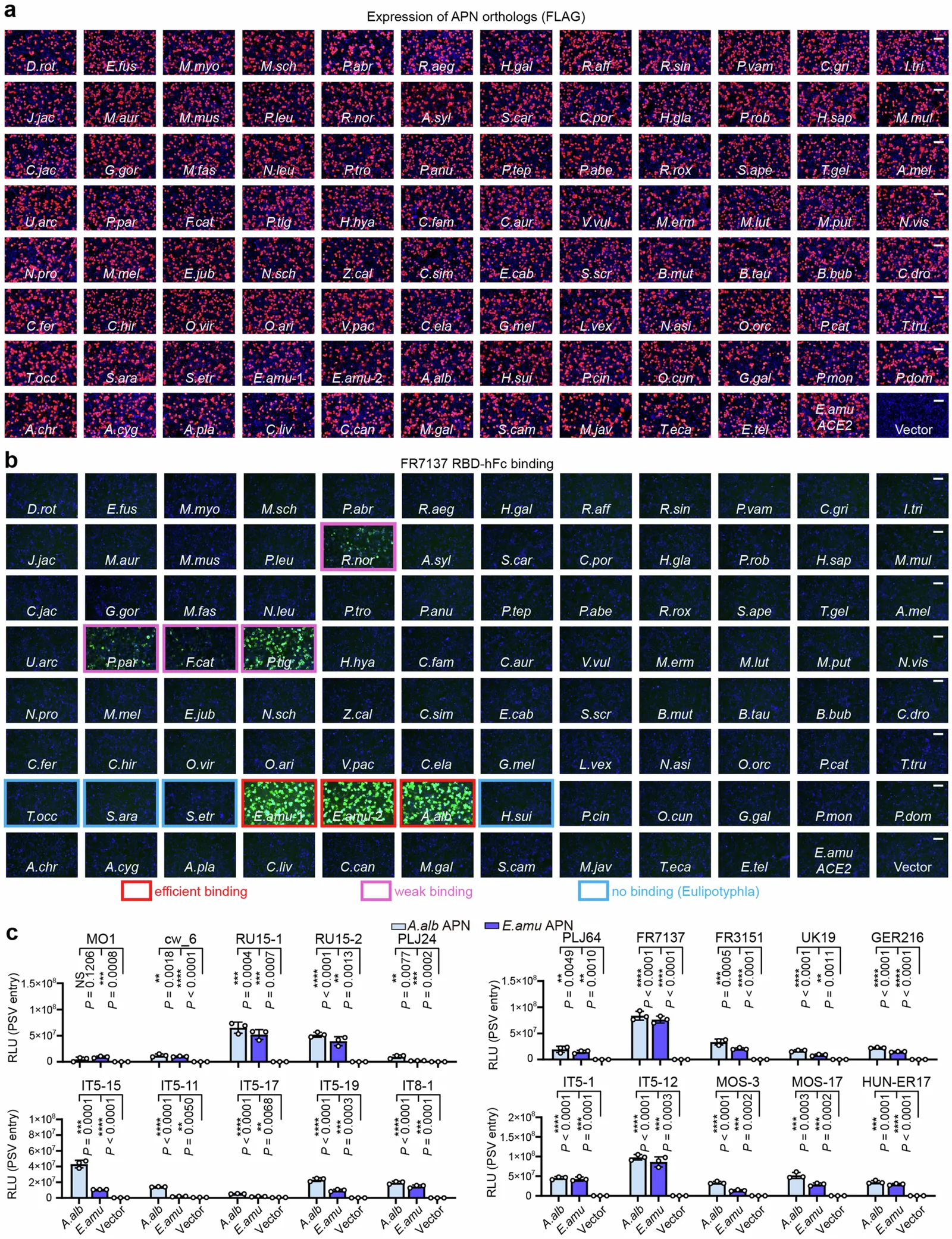
Expression and EriCoV RBD-binding efficiency of APN orthologues and pseudovirus entry mediated by two hedgehog APN orthologues. (**a**) Immunofluorescence of 93 FLAG-tagged APN orthologues (Supplementary Table 3) transiently expressed in HEK293T cells (red). (**b**) FR7137 RBD–hFc binding (green) to HEK293T cells transiently expressing the indicated APN orthologues. Data are representative of two independent experiments with similar results. (**c**) Entry of VSV-based pseudoviruses of selected EriCoV strains into HEK293T cells transiently expressing *A.alb* APN or *E.amu* APN. Pseudoviruses were pretreated with 1.25 mg m^−^^1^ trypsin. Mean ± s.d. and n = 3 independently infected wells for c. Statistical significance was determined using unpaired two-tailed *t*-tests. *P < 0.05, **P < 0.01, ***P < 0.001, and ****P < 0.0001, NS: not significant. Scale bars: 100 μm. Source data

To assess the spillover risk of EriCoVs, we evaluated the receptor function of 93 mammalian and avian APN orthologues, all with validated expression (Fig. 2a, Extended Data Fig. 3a and Supplementary Table 3). Overall, EriCoVs exhibited restricted APN tropism. European (RU15-1 and FR7137) and Asian (MO1) strains efficiently recognize *E.* *amurensis* APN and the closely related *Atelerix albiventris* APN from African pygmy hedgehogs. In contrast, APNs from shrews *Sorex araneus* and *Suncus etruscus*, also belonging to the Eulipotyphla, were not recognized. APNs from tigers (*Panthera tigris*), leopards (*Panthera pardus*) and domestic cats (*Felis catus*) supported detectable FR7137 and RU15-1 RBD binding, whereas rat (*Rattus norvegicus*) APN was recognized only by FR7137 (Extended Data Fig. 3a,b and Supplementary Fig. 2a,b). Consistently, APNs supporting FR7137 RBD binding also promoted pseudovirus entry (Fig. 2b). Similar to *E.* *amurensis* APN, *A.* *albiventris* APN efficiently supported entry of all 20 tested EriCoV strains (Extended Data Fig. 3c).

**Extended Data Fig. 4:**
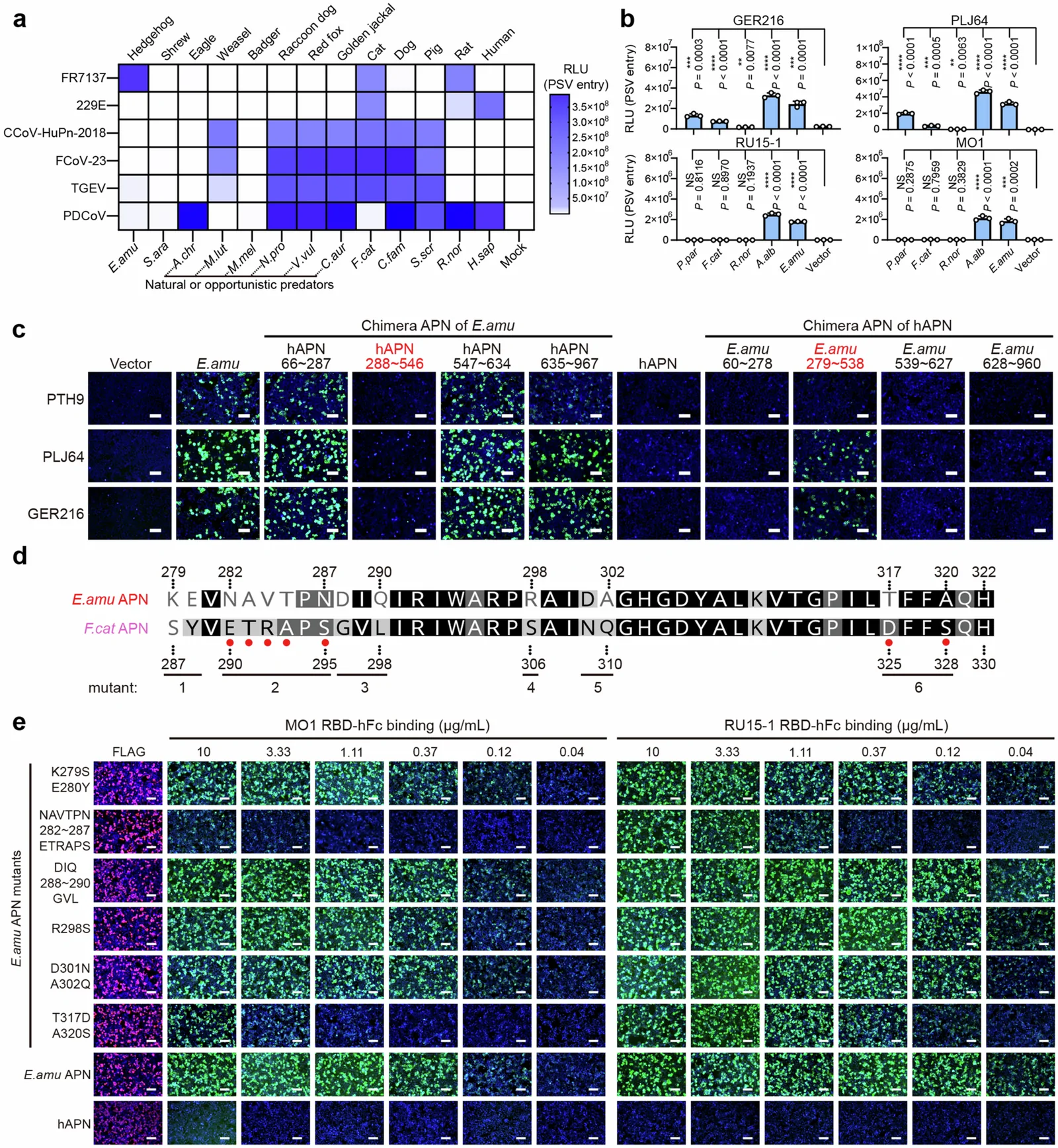
APN tropism APN-using coronaviruses and host tropism determinants of EriCoVs. (**a**) Heat map (means of 3 independently infected wells) of pseudovirus entry of APN-using coronaviruses in HEK293T cells transiently expressing APN orthologues from natural or opportunistic predators or species with overlapping habitats of hedgehogs. (**b**) Entry of GER216, PLJ64, RU15-1, and MO1 pseudoviruses into HEK293T cells transiently expressing indicated APN orthologues. (**c**) EriCoV RBD–hFc binding to HEK293T cells transiently expressing APN chimeras with domain swaps between human and *E.amu* APNs. Data are representative of two independent experiments with similar results. (**d**) Sequence alignment of *E.amu* APN and *F.cat* APN in the mapped region. Residues implicated in host range determination are indicated by red dots. (**e**) Binding (green) of MO1 and RU15-1 RBD–hFc to HEK293T cells transiently expressing *E.amu* APN mutants (red). Representative data are from a single immunofluorescence experiment. Recombinant proteins were diluted in SMM 293-TII Expression Medium at the indicated concentrations. Data are presented as mean for a, mean ± s.d. for b. Statistical significance was determined using unpaired two-tailed *t*-tests (n = 3 independently infected wells). *P < 0.05, **P < 0.01, ***P < 0.001, ****P < 0.0001, and NS: not significant. RLU: relative light units. Scale bars: 100 μm. PSV: pseudovirus. Source data

We further examined APNs from biologically relevant species with frequent environmental overlap with hedgehogs or that prey on them. Compared with other APN-using coronaviruses, EriCoVs consistently exhibit restricted orthologue tropism, as only felid and rat APNs supported limited FR7137 pseudovirus entry, whereas APNs from natural or opportunistic predators (for example, badgers^35^) did not (Extended Data Fig. 4a and Supplementary Fig. 3a). Expanded validation across additional EriCoV strains confirmed felid APN usage (Fig. 2c and Extended Data Fig. 4b).

To define determinants underlying this restriction, we constructed domain-swapped chimeras between human and *E.* *amurensis* APNs (Fig. 2d). Replacement of domain 2 in *E.* *amurensis* APN with the human counterpart abolished binding, whereas reciprocal substitution of *E.* *amurensis* domain 2 in human APN counterpart restored binding (Fig. 2e and Extended Data Fig. 4c). Fine mapping narrowed down the critical region to residues 288–331_hAPN_, among which β16 and α2 are more surface exposed; the latter contains a restrictive N319 glycosylation in human APN but not *E. amurensis* APN (Fig. 2f–h and Supplementary Fig. 3b–d). Logoplot analysis of the 93 APN orthologues revealed high variability in this region, with *E.* *amurensis* APN displaying less conservation around β16 and within α2 (Fig. 2i). Notably, the absence of the N319_hAPN_ glycosylation in felid APNs may partially explain their receptor compatibility. Additionally, substitution analysis based on *F. catus* APN identified residues within β16 (positions 282–287_E.amuAPN_) and α2 (T317_E.amuAPN_ and A320_E.amuAPN_) as critical for efficient binding (Extended Data Fig. 4d,e).

### Structural basis of *E.amurensis* APN recognition by EriCoV

**Fig. 3:**
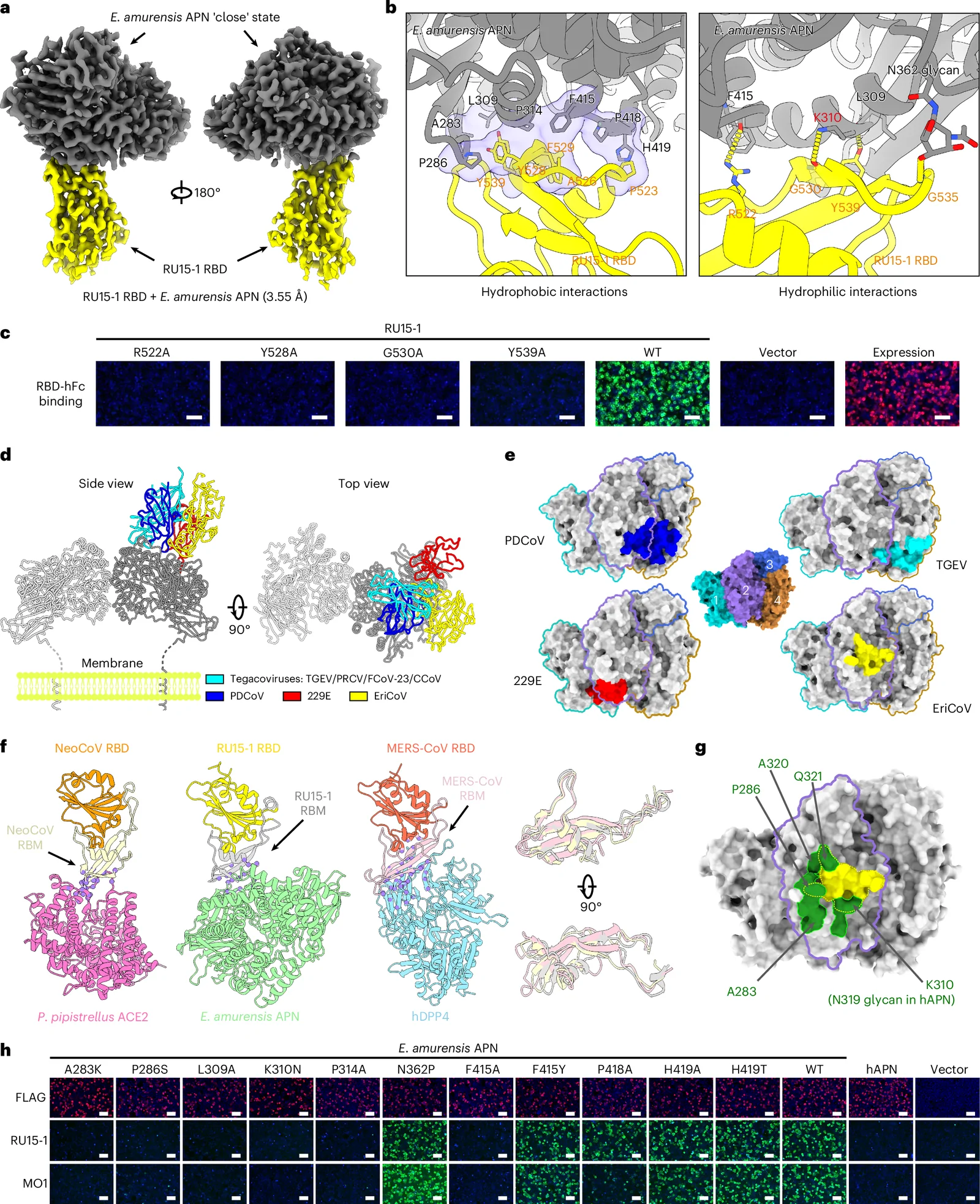
Structural basis of RU15-1 RBD recognition of *E.amurensis* APN. **a**, Cryo-EM structure (27OA) of the RU15-1 RBD (yellow) in complex with *E.* *amurensis* APN (grey). **b**, Detailed interactions at the RU15-1 RBD–*E.* *amurensis* APN interface. Key interacting residues and hydrogen bonds are rendered as sticks and yellow dashed lines, respectively. The hydrophobic pocket is shown as a semitransparent blue shadow. K310 corresponding to the N-glycosylation site in human APN is coloured red. **c**, Binding (green) of WT and mutant RU15-1 RBD–hFc to HEK293T cells transiently expressing *E.* *amurensis* APN (red). **d**, Four distinct APN-binding modes used by representative coronaviruses, shown in both side and top views. TGEV is shown as a representative of tegacoviruses. PDB IDs, TGEV (8Z27), PRCV (4F5C), FCoV-23 (9DAZ), CCoV-HuPn-2018 (7U0L), PDCoV (7VPP), HCoV-229E (8WDE) and EriCoV (RU15-1) (9XWA). Only human APN dimer (grey) is shown, for clarity. **e**, APN domains and corresponding RBD footprints are indicated (colour scheme is the same as in **d**). APN domains 1–4 are colour-coded in dark turquoise, medium purple, royal blue and dark goldenrod, respectively. **f**, Structural comparison of merbecovirus binding modes with three distinct receptors: NeoCoV–*Pipistrellus pipistrellus* ACE2 (7WPO), EriCoV–*E.* *amurensis* APN (9XWA) and MERS-CoV–hDPP4 (4KR0). Right: zoomed-in view showing the superimposed RBMs. The *P. pipistrellus* ACE2, *E.* *amurensis* APN and hDPP4 are coloured hot pink, light green and sky blue, respectively. NeoCoV, EriCoV and MERS-CoV RBDs are coloured dark orange, gold and tomato, respectively, with their corresponding RBMs highlighted in wheat, silver and light pink, respectively. **g**, Summary of the identified host-range determinants (green) and RU15-1 RBD binding interface (yellow) mapped onto *E.* *amurensis* APN surface. Yellow dashed outline indicates interacting residues critical for host range; purple outline indicates domain 2. **h**, RU15-1 and MO1 RBD–hFc binding (green) to HEK293T cells transiently expressing WT or mutant *E.* *amurensis* APN, assessed by immunofluorescence (red). Representative RBD-binding immunofluorescence images (green in **c**,**h**) are shown from two independent experiments with similar results. Scale bars, 100 μm.

**Extended Data Fig. 5:**
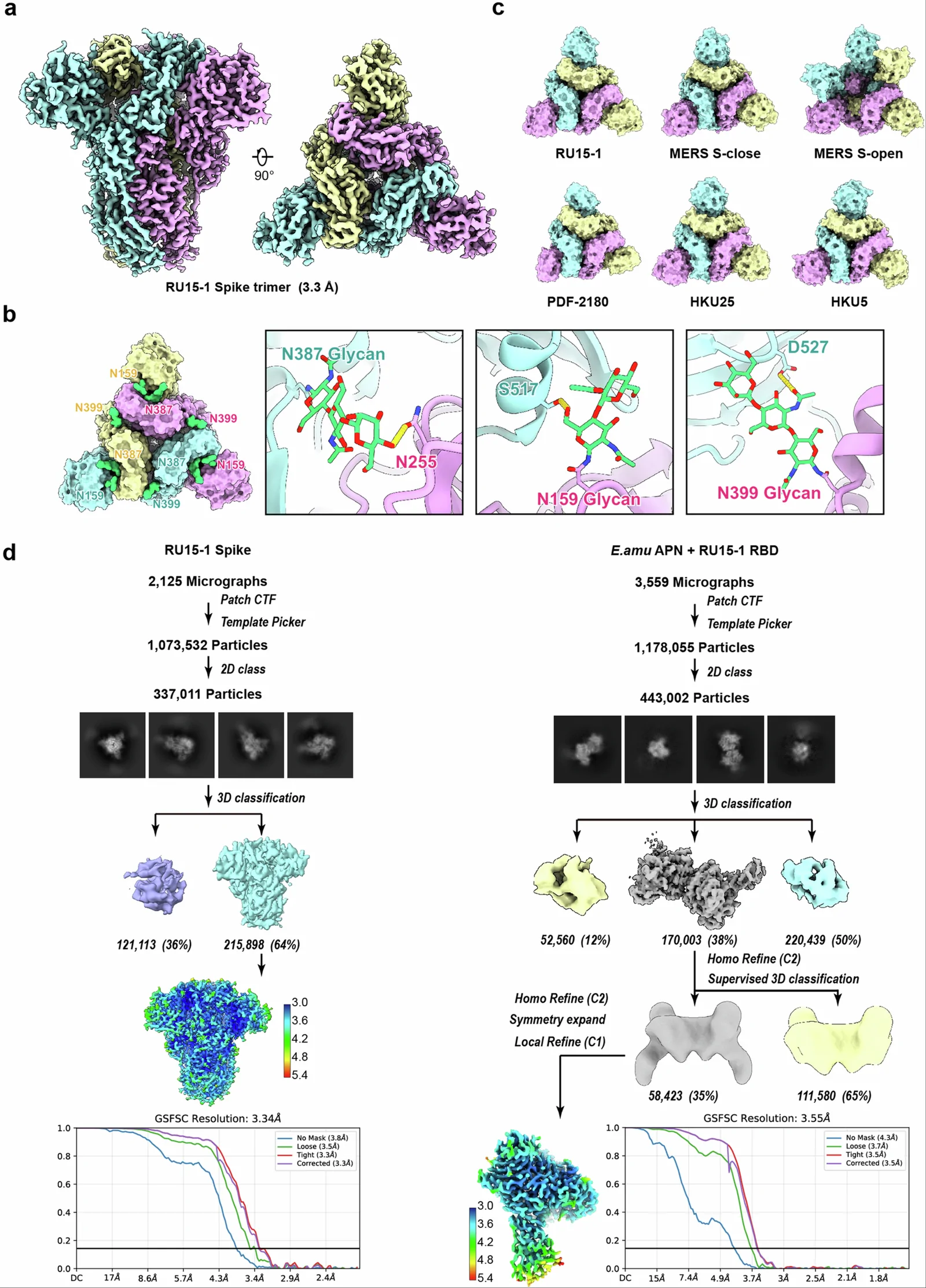
Conformations of the RU15-1 spike and Cryo-EM data processing workflow. (**a**) Cryo-EM structure of the RU15-1 spike trimer. Cryo-EM reconstruction of the RU15-1 spike trimer at 3.3 Å resolution shown in front (left) and top (right) views. The three protomers are colored cyan, purple, and yellow, respectively. (**b**) Structural representation of glycosylation sites on the RU15-1 spike. N-linked glycosylation sites are shown in green (left: cartoon representation; right: stick representation). The three protomers are depicted in cyan, purple, and yellow, respectively, and key residues are annotated. (**c**) Cryo-EM reconstructions showing the conformational states of spike trimers from representative merbecoviruses, including RU15-1, MERS-CoV (open and closed), PDF-2180, HKU25, and HKU5. The three protomers are colored cyan, purple, and yellow, respectively. (**d**) Flowcharts for structure determinations of the RU15-1 spike and RU15-1 RBD–*E.amu* APN complex. Gold-standard Fourier shell correlation (FSC) curves are shown to assess map quality and resolution. Local resolution assessments of cryo-EM maps using rainbow are shown.

To elucidate the structural basis of EriCoV receptor recognition, we determined cryogenic electron microscopy (cryo-EM) structures of EriCoV spikes and RBD–APN complexes. The RU15-1 apo spike adopts a closed, three-RBD-down conformation stabilized by glycan-mediated contacts between RBD glycans N387/N399 or N-terminal domain glycan N159 and neighbouring spike surfaces (Extended Data Fig. 5a,b). Similar glycan-mediated stabilization is observed in other merbecoviruses, including HKU5, HKU25 and PDF-2180 (Extended Data Fig. 5c). Considering the closed spike, we chose RU15-1 RBD and *E.* *amurensis* APN for receptor-complex analysis, which yielded 443,002 particles from 3,559 micrographs. Approximately 38% of particles represented the target complex, and symmetry expansion with local refinement yielded a 3.55-Å reconstruction of the RU15-1 RBD-bound APN monomer (Fig. 3a and Extended Data Fig. 5d).

RU15-1 residues P523, A526, Y528, F529 and Y539 contact APN residues A283, P286, L309, P314, F415, P418 and H419, forming an extensive hydrophobic interface. Additional hydrogen bonds are formed by RU15-1 Y539 and R522 with APN L309 and F415, respectively, whereas the backbone atoms of G530 and G535 interact with APN K310 and the N362 glycan. Notably, K310 in *E.* *amurensis* APN corresponds to a glycosylation site in human APN that may sterically hinder viral binding (Fig. 3b). Consistently, RU15-1 R522A, Y528A, G530A and Y539A mutations markedly reduced *E.* *amurensis* APN binding (Fig. 3c).

**Extended Data Fig. 6:**
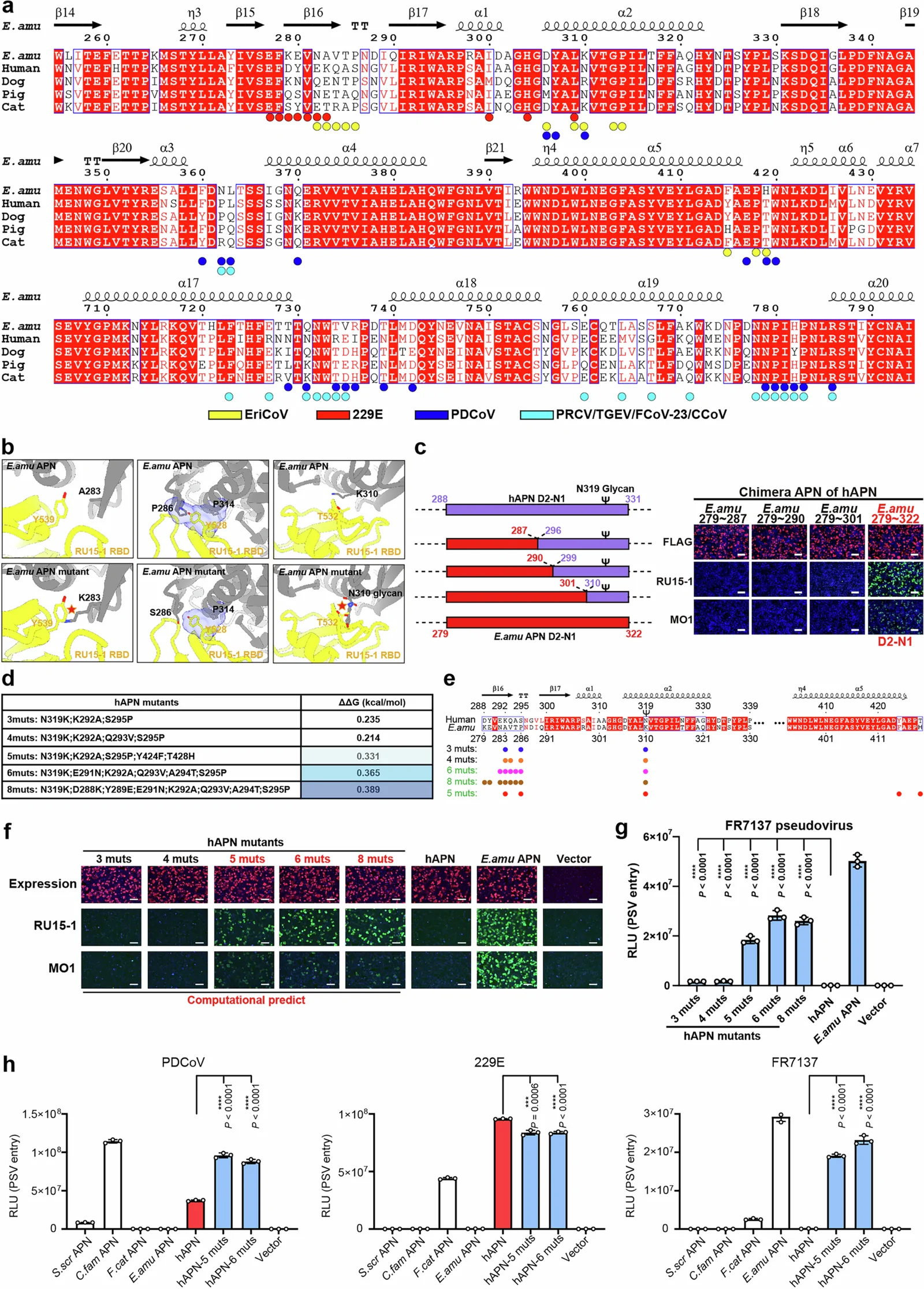
Prediction and validation of key determinants for EriCoV–APN interaction. (**a**) Multiple sequence alignment of APN orthologues from *Erinaceus amurensis* (E.amu), *Homo sapiens* (Human), *Canis lupus* (Dog), *Sus scrofa* (Pig), and *Felis catus* (cat). Residues within 4 Å of viral RBDs are indicated by colored circles: yellow for EriCoV, red for HCoV-229E, blue for PDCoV, and cyan for tegacoviruses (PRCV, TGEV, FCoV-23, and CCoV). (**b**) Structural basis of key *E.amu* APN mutations affecting RU15-1 binding. The upper panel showed the WT *E.amu* APN, and the lower panel displayed the corresponding residue-mutant *E.amu* APN. *E.amu* APN was colored in grey, and the RU15-1 RBD was shown in yellow. The hydrophobic pocket was indicated by a semi-transparent blue surface, hydrogen bonds were depicted as yellow dashed lines, and steric clashes were highlighted by red pentagrams. (**c**) Substitution of the D2-N1 region (residues 279-322) from *E.amu* APN enabled human APN (hAPN) to support RU15-1 and MO1 RBD binding. N-319 glycosylation in human APN is labeled with Ψ. (**d**) Predicted effects of human APN mutations on its binding affinity to RU15-1. Binding affinity changes were estimated by PPI2 with ΔΔG (kcal/mol) calculated as ΔG_wt - ΔG_mut. Positive ΔΔG values indicate enhanced binding affinity of the mutant human APN to RU15-1 relative to the wild-type complex. (**e-g**) Mutation combinations identified by structural analysis and PPI2 prediction (e), rendered human APN capable of supporting RU15-1 and MO1 RBD–hFc binding (f) and FR7137 pseudovirus entry (g). (**h**) Pseudovirus entry of APN-using coronaviruses in HEK293T cells transiently expressing indicated APN orthologues or indicated human APN mutants. Expression of WT and mutated APN was confirmed by FLAG-tag immunostaining (red) for c and f. Data are presented as mean ± s.d. for g and h. Data in panel **h** are representative of two independent pseudovirus entry experiments with similar results. Statistical significance was determined using unpaired two-tailed *t*-tests (n = 3 independently infected wells). ***P < 0.001 and ****P < 0.0001. Scale bars: 100 μm for c and f. Source data

Three APN-binding modes have been documented among alpha- and deltacoronaviruses. Tegacoviruses transmissible gastroenteritis virus (TGEV), porcine respiratory coronavirus (PRCV), canine coronavirus-human pneumonia-2018 (CCoV-HuPn-2018) and feline coronavirus-23 (FCoV-23) share one mode, whereas human coronavirus-229E (HCoV-229E) and porcine deltacoronavirus (PDCoV) adopt two additional modes with distinct RBD orientations and footprints^27,36–39^ (Fig. 3d and Extended Data Fig. 6a). EriCoVs, as betacoronaviruses, adopt a previously uncharacterized laterally shifted APN-binding mode, targeting a central region APN domain 2. In contrast, footprints of other modes involve not only domain 2 but also variable contributions from domain 1 or 4 (Fig. 3e and Extended Data Fig. 6a). This convergent APN usage resembles the various ACE2-binding strategies among coronaviruses^17,18,25,26,28,40^ (Fig. 3e). Merbecoviruses engage receptors through a shared RBM β-sheet surface, but RBM indels and sequence variation remodel the interface, driving receptor specificity (Fig. 3f).

Residues identified as determinants of receptor compatibility in the APN swap analysis (Fig. 2i) clustered within or near the RBD footprint, including *E.* *amurensis* APN A283, P286, K310, A320 and Q321 (Fig. 3g). Substitutions A_E.amu_283K_human_, P_E.amu_286S_human_ and K_E.amu_310N_human_ markedly reduced RU15-1 RBD binding (Fig. 3h), consistent with predictions of introducing steric clashes (A283K and K310N) or disrupting hydrophobic contacts (P286S) (Extended Data Fig. 6b). Additional L_E.amu_309A, P_E.amu_314A and F_E.amu_415A substitutions also impaired binding, supporting their roles in EriCoV recognition (Fig. 3h and Supplementary Fig. 4a). Consistently, replacement of human APN region 288–331 with the *E.* *amurensis* APN counterpart (residues 279–322) conferred RU15-1 RBD binding (Extended Data Fig. 6c). Combining structural analysis and PPI2 predictions, we further identified combinations of five to six substitutions that were sufficient to convert human APN into a functional EriCoV receptor (Extended Data Fig. 6d–g). These mutations did not substantially impair pseudovirus entry mediated by 229E and PDCoV spikes, consistent with their distinct binding interfaces (Extended Data Fig. 6h).

### Proteases facilitate EriCoV S-mediated entry

**Extended Data Fig. 7:**
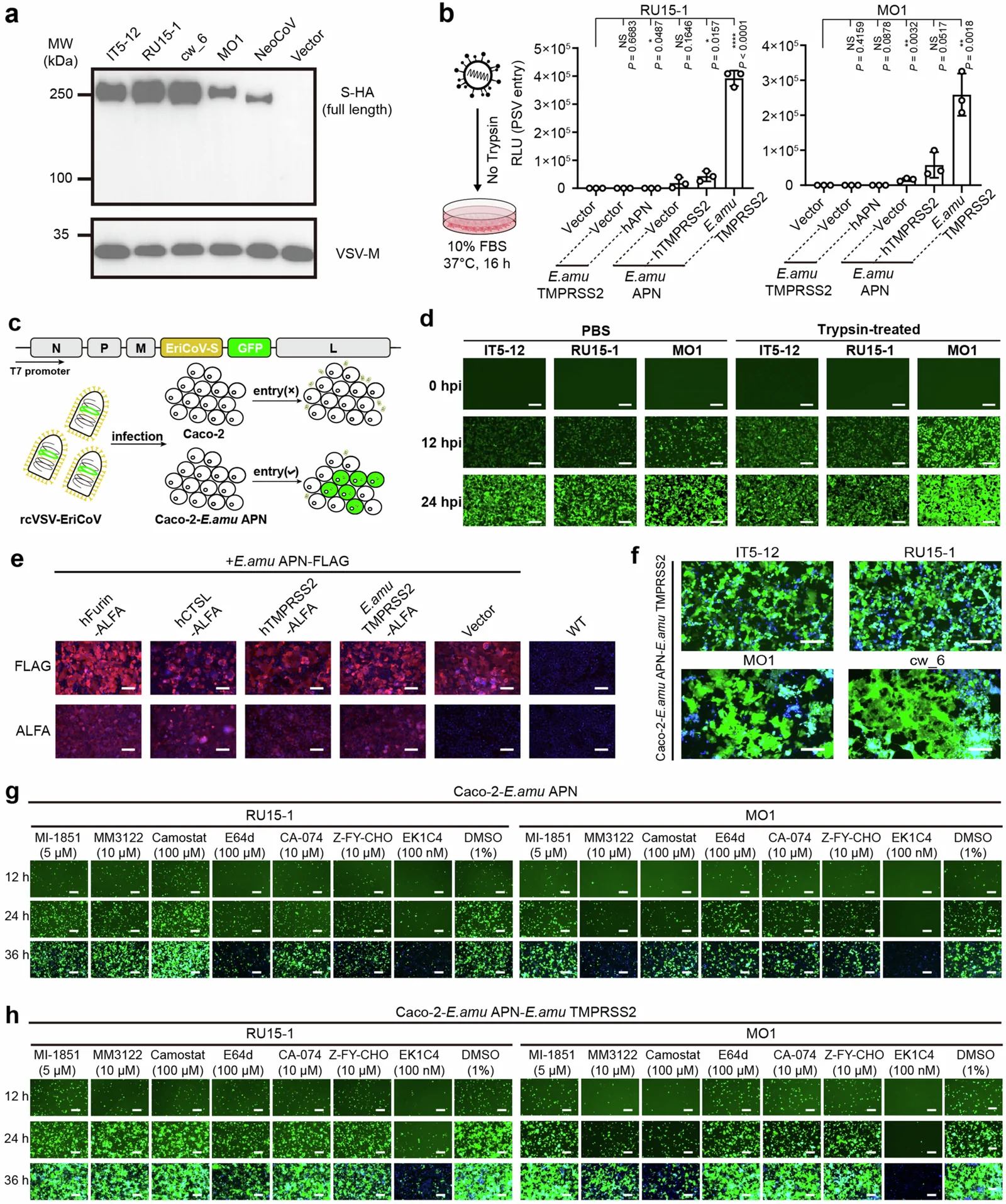
Analysis of the cellular protease usage by single-round and replication-competent EriCoV pseudoviruses. (**a**) Western blot analysis of EriCoV S glycoproteins in VSV pseudoviral particles detecting the C-terminally fused HA tag. VSV-M served as a virion normalization control. Data are representative of three independent experiments with similar results. (**b**) Entry of RU15-1 and MO1 pseudovirus into HEK293T cells transiently expressing the indicated human or hedgehog APN and TMPRSS2 orthologues. Flowcharts summarize the infection conditions without trypsin treatment. (**c**) Genetic organization and amplification workflow for rcVSV-S constructs encoding EriCoV S glycoproteins. (**d**) Propagation of rcVSV-RU15-1, rcVSV-IT5-12, and rcVSV-MO1 in Caco-2 cells stably expressing *E.amu* APN with or without the presence of 20 μg ml^−^^1^ TPCK-treated trypsin in 2% FBS during viral amplification. (**e**) Immunofluorescence validation of stable expression of *E.amu* APN (FLAG-tagged) and the indicated cellular proteases (ALFA-tagged) in Caco-2 cells. (**f**) Strain-specific syncytium formation induced by rcVSV-IT5-12, rcVSV-RU15-1, rcVSV-MO1, and rcVSV-cw_6 in Caco-2 cells stably co-expressing *E.amu* APN and *E.amu* TMPRSS2 at 48 h post-infection. (**g**) Amplification of rcVSV-RU15-1 (left), or rcVSV-MO1 (right) in Caco-2-*E.amu* APN cells upon the presence of indicated inhibitors without trypsin treatment. (**h**) Propagation of rcVSV-RU15-1 (left), and rcVSV-MO1 (right) in Caco-2 cells stably coexpressing *E.amu* APN and *E.amu* TMPRSS2 in the presence of the indicated inhibitors during viral amplification. Independent experiments were performed three times for panels **d** and **f**, and twice for panels **e**, **g**, and **h**, with similar results. Data are presented as mean ± s.d. for b. Statistical significance was determined using unpaired two-tailed *t*-tests (n = 3 independently infected wells). *P < 0.05, **P < 0.01, ***P < 0.001, ****P < 0.0001, and NS: not significant. Scale bars: 200 μm for d, g and h, 100 μm for e and f. Source data

Coronavirus spike activation commonly includes an initial S_1_/S_2_ cleavage and subsequent S_2_’ cleavage upon receptor engagement, either by extracellular or cellular proteases^41–44^. Analysis of the EriCoV spike in pseudoviruses revealed inefficient S_1_/S_2_ processing during biogenesis (Extended Data Fig. 7a), a feature common to enteric coronaviruses without a furin cleavage site^26,45^. Correspondingly, trypsin markedly enhanced pseudovirus entry and spike-mediated cell–cell fusion (Fig. 1j and Extended Data Fig. 2d).

**Fig. 4:**
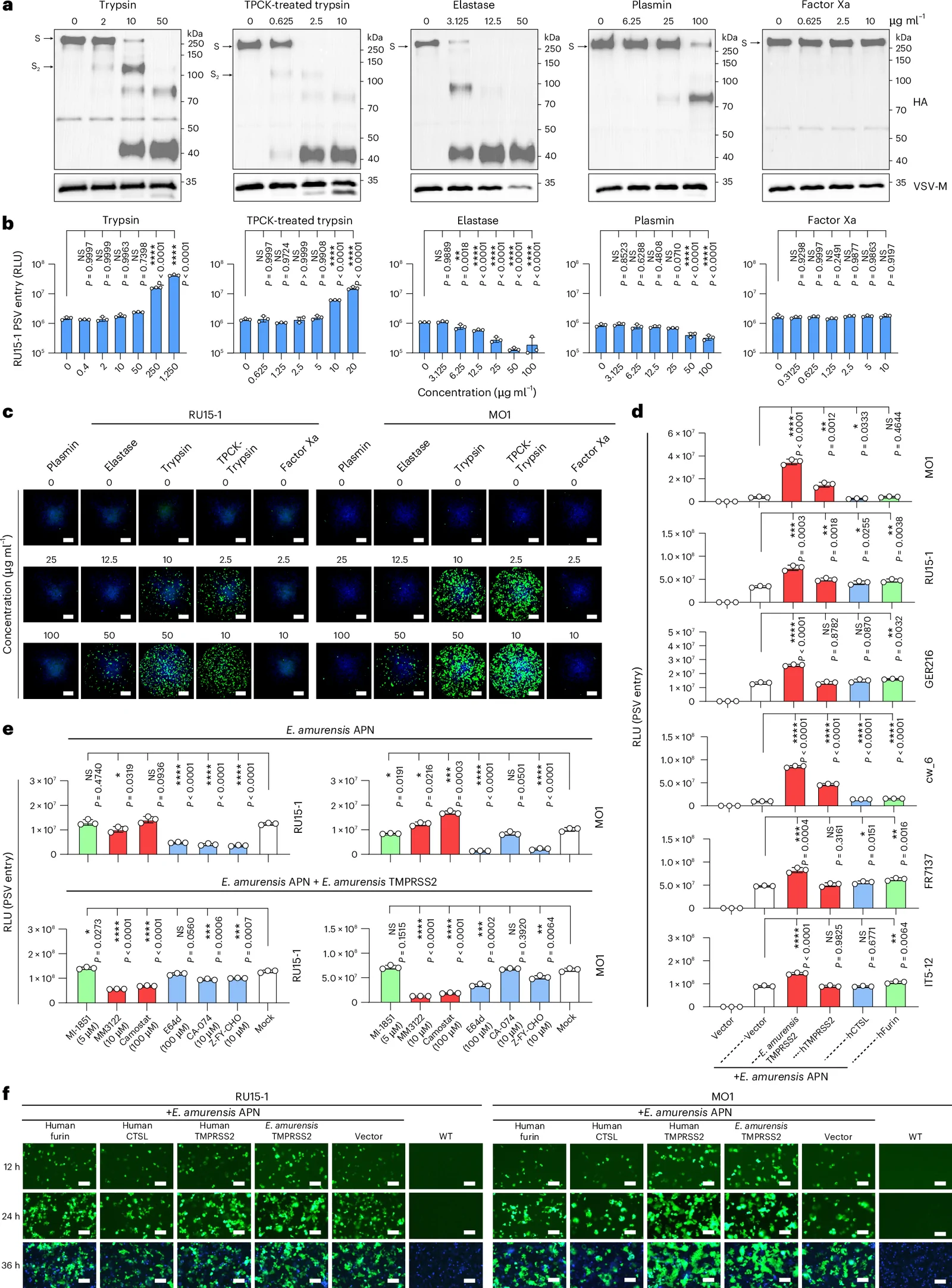
Characterization of protease-dependent EriCoV spike activation. **a**, Cleavage of RU15-1 S in VSV pseudoviruses following treatment with the indicated exogenous proteases (37 °C, 1 h), examined by western blot detecting the C-terminally fused hemagglutinin tag (HA) tags. VSV-M served as a virion normalization control run on different gels. The predicted molecular weights of full-length S and the S_1_/S_2_ cleavage product (S_2_) are indicated. **b**, VSV-based pseudovirus entry of RU15-1 in HEK293T cells transiently expressing *E.* *amurensis* APN. Pseudoviruses were pretreated with indicated proteases at 37 °C for 1 h. **c**, Cell–cell membrane fusion mediated by RU15-1-S and MO1-S in HEK293T cells transiently expressing *E.* *amurensis* APN treated with indicated concentrations of proteases at 37 °C for 30 min. **d**, Entry efficiency of selected EriCoV pseudoviruses in HEK293T cells transiently expressing *E.* *amurensis* APN alone or together with human furin (hFurin), human cathepsin L (hCTSL), human TMPRSS2 (hTMPRSS2) or hedgehog TMPRSS2 (*E.* *amurensis* TMPRSS2). **e**, RU15-1 and MO1 pseudovirus entry in HEK293T cells transiently expressing *E.* *amurensis* APN alone or together with *E.* *amurensis* TMPRSS2 in the presence of indicated protease inhibitors during infection. **f**, Amplification of rcVSV-EriCoV-S in Caco-2-*E.* *amurensis* APN cells stably expressing the indicated proteases. Pseudoviruses were pretreated with 1.25 mg ml^−1^ trypsin at 37 °C for 1 h for **d** and **e**. Data are presented as mean ± s.d. for **b**, **d** and **e**; data are representative of two experiments with similar results. Statistical significance was determined using unpaired two-tailed *t*-tests (*n* = 3 independently infected wells) for **d** and **e**; one-way analysis of variance followed by Dunnett’s test (*n* = 3 independently infected wells) for **b**. *P < 0.05, **P < 0.01, ***P < 0.001 and ****P < 0.0001. Scale bars, 5 mm (**c**), 100 μm (**f**). Source data

We first assessed a panel of exogenous proteases. Trypsin, or its *N*-tosyl-l-phenylalanyl chloromethyl ketone (TPCK)-treated version, efficiently cleaved RU15-1 spikes (Fig. 4a) and markedly enhanced pseudovirus entry (Fig. 4b) and cell–cell fusion (Fig. 4c). Elastase and plasmin, but not Factor Xa, also cleaved spike proteins but produced limited or inhibitory effects on entry at higher concentrations (Fig. 4a–c). These results indicate that EriCoVs prefer trypsin-like proteases and cleavage alone is insufficient for productive entry.

We then investigated the contribution of cellular proteases. In HEK293T-*E.* *amurensis* APN cells lacking endogenous TMPRSS2 expression^42^, co-expression of TMPRSS2, especially the hedgehog version, enhanced EriCoV pseudovirus entry, particularly for the Asian strains MO1 and cw_6 (Fig. 4d). Human APN remained non-functional even in the presence of *E.* *amurensis* TMPRSS2, indicating that TMPRSS2 acts as an accessory factor (Extended Data Fig. 7b). Similar strain-dependent enhancement was observed across a broader panel of 20 EriCoVs (Supplementary Fig. 5a). Protease inhibition experiments revealed flexible and context-dependent protease usage. In HEK293T cells transiently expressing *E.* *amurensis* APN, pseudovirus entry was sensitive to the cysteine protease inhibitor E64d^46^, as well as to the more selective cathepsin inhibitors CA-074 and Z-FY-CHO^47^. Upon *E.* *amurensis* TMPRSS2 co-expression, entry became partially sensitive to the serine protease inhibitor camostat^42^ and the more selective TMPRSS2 inhibitor MM3122^48^. In contrast, the furin inhibitor MI-1851^49^ had no detectable effect (Fig. 4e).

To evaluate multiround amplification, we generated replication-competent VSV (rcVSV) encoding IT5-12, RU15-1, MO1 or cw_6 spikes and examined their amplification in human colorectal cell line Caco-2 cells endogenously expressing TMPRSS2 (Extended Data Fig. 7c). Upon *E.* *amurensis* APN expression, amplification was readily detected and enhanced by trypsin (Extended Data Fig. 7d). Co-expression of *E.* *amurensis* TMPRSS2 further promoted viral spread, particularly for the Asian strains MO1 and cw_6, which induced more extensive syncytium formation than the European strains IT5-12 and RU15-1 (Extended Data Fig. 7e,f). Consistently, rcVSV-MO1 spreads more rapidly in Caco-2-*E.* *amurensis* APN cells co-expressing TMPRSS2 but not furin or cathepsin L (Fig. 4f).

Without *E.* *amurensis* TMPRSS2 overexpression, rcVSV-RU15-1 propagation remained primarily sensitive to E64d, CA-074 and Z-FY-CHO, whereas rcVSV-MO1 showed partial sensitivity to camostat and stronger inhibition by MM3122, indicating strain-specific reliance on endosomal versus cell-surface proteases (Extended Data Fig. 7g). Upon *E.* *amurensis* TMPRSS2 co-expression, rcVSV-RU15-1 remains partially sensitive to E64d, whereas MO1 showed increased sensitivity to camostat relative to the human TMPRSS2-selective MM3122 (Extended Data Fig. 7h). Notably, the pan-coronavirus fusion inhibitor EK1C4 peptide potently suppressed the viral amplification under all tested conditions^50^.

Together, these results indicate that EriCoV spike activation can proceed through multiple proteolytic pathways. Extracellular trypsin-like proteases provide highly efficient activation, whereas cellular proteases, including TMPRSS2 and endosomal cathepsins, contribute in a context-dependent and strain-specific manner.

### Neutralizing antibodies against EriCoV entry

**Fig. 5:**
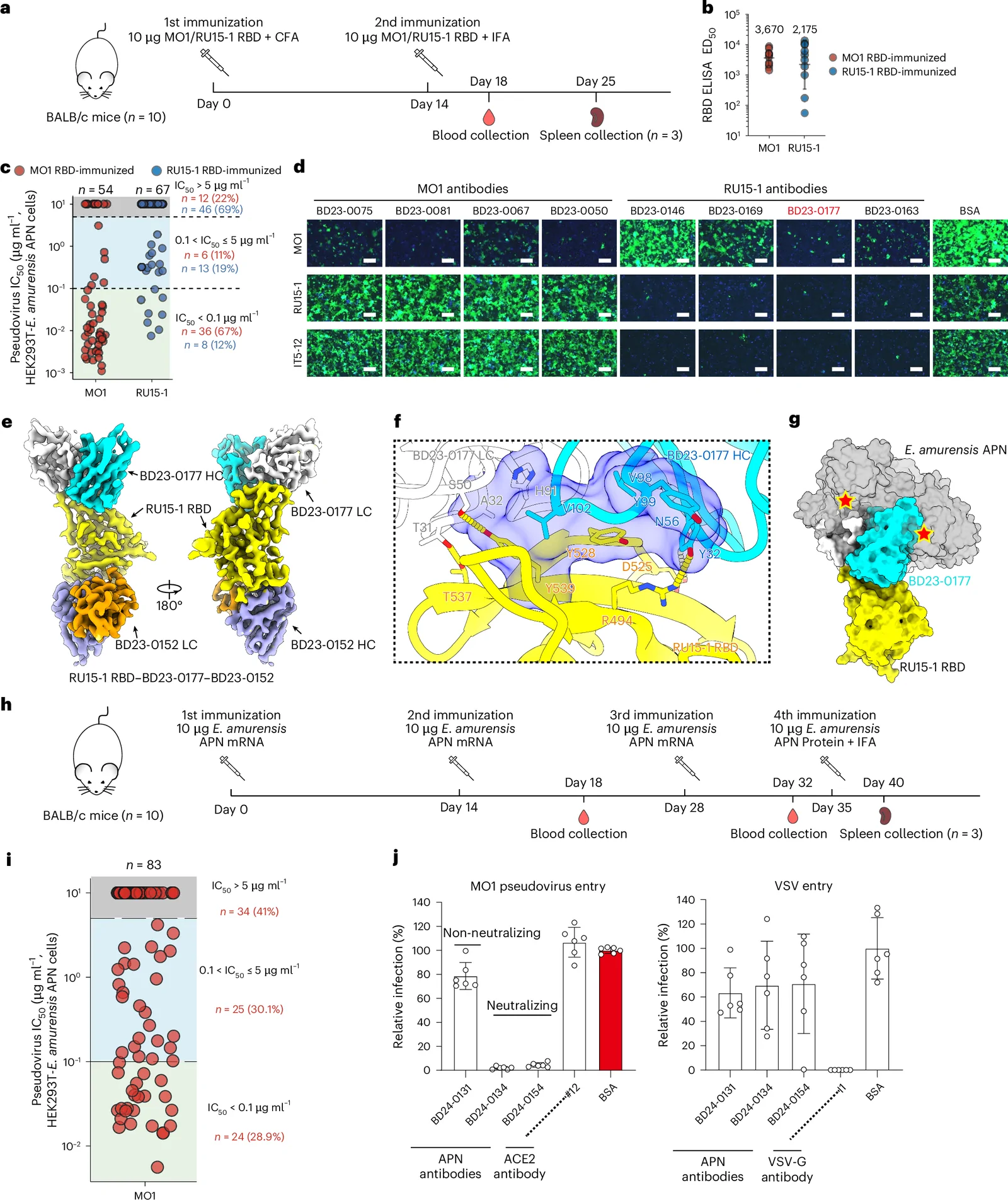
Neutralizing antibodies efficiently block EriCoV S-mediated entry. **a**, Schematic of EriCoV RBD immunization (*n* = 10 per group). CFA, complete Freund’s adjuvant; IFA, incomplete Freund’s adjuvant. **b**, ELISA analyses of serum samples from immunized mice. Median effective dose (ED_50_) values were calculated using four-parameter logistic regression. Data are presented as geometric mean titres with geometric standard deviations, and geometric mean titres are shown above each group. **c**, Pseudovirus IC_50_ values of antibodies derived from MO1 (red circles, *n* = 54) or RU15-1 (blue circles, *n* = 67) immunization groups, categorized by potency. Neutralization assays were performed in HEK293T-*E.* *amurensis* APN cells. **d**, Inhibition of rcVSV-EriCoV-S propagation by RU15-1- or MO1-targeting neutralizing antibodies (10 μg ml^−1^) in Caco-2 cells stably expressing the *E.* *amurensis* APN and *E.* *amurensis* TMPRSS2. The inhibition assays shown were representative of three independent experiments with similar results. **e**, The cryo-EM structures of the EriCoV RU15-1 RBD bound to BD23-0177 and BD23-0152 Fabs. **f**, The interaction details of the RU15-1 RBD–BD23-0177 interface. The hydrophobic pocket and hydrogen bonds are shown with coloured semitransparent blue and yellow dashed lines, respectively. **g**, Overlay of the cryo-EM complex structures of the RU15-1 RBD–*E.* *amurensis* APN and *E.* *amurensis* APN–BD23-0177 based on superposition of the RU15-1 RBD. Red stars indicate steric crash. **h**, Schematic of *E.* *amurensis* APN immunization (*n* = 10). **i**, Pseudovirus IC_50_ values of mAbs derived from *E.* *amurensis* APN-immunized mice, categorized by potency. MO1 neutralization assays were performed in HEK293T-*E.* *amurensis* APN cells. **j**, Primary cells derived from the kidney of African pygmy hedgehogs (*A.* *albiventris*) were tested with MO1 VSV pseudovirus entry in the presence of 10 μg ml^−1^ of indicated antibodies. VSV-G-based pseudovirus served as a virus control, which can be neutralized by an anti-VSV-G antibody (from the I1-hybridoma supernatant). Mean ± s.d. for **j** (*n* = 6 independently infected wells). BSA, 10 μg ml^−1^ for **d** and **j**. Scale bars, 200 μm. Source data

**Extended Data Fig. 8:**
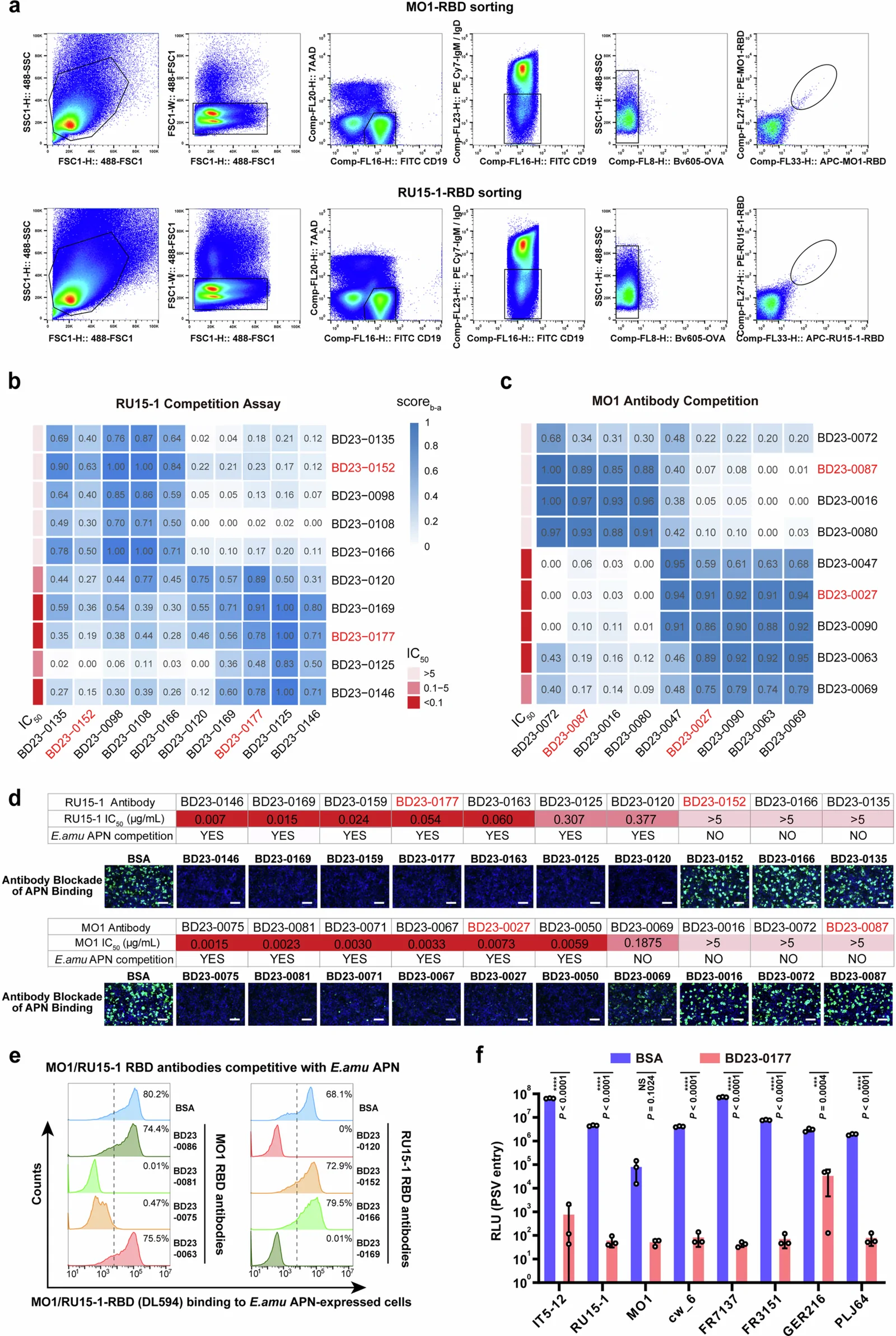
EriCoV RBD-specific antibodies efficiently inhibited the EriCoV pseudovirus entry. (**a**) Flow cytometry gating strategy for isolation of antigen-specific B cells. Splenic B cells were pooled from immunized BALB/c mice and stained for surface markers and antigen probes. The representative gating strategy is shown for MO1-RBD (top) and RU15-1-RBD (bottom). Lymphocytes were first identified based on forward scatter (FSC) and side scatter (SSC) profiles, followed by doublet exclusion. Live cells were gated by excluding 7-AAD^+^ events. CD19^+^ B cells were selected, and IgM^−^/IgD^−^ class-switched B cells were further enriched. OVA-negative (BV605^−^) cells were gated to exclude non-specific binding. Antigen-specific B cells were identified as double-positive for PE-conjugated SA-MO1-RBD or SA-RU15-1-RBD and APC-conjugated SA-MO1-RBD or SA-RU15-1-RBD, respectively. These antigen-binding B cells were subsequently sorted for downstream single-cell V(D)J sequencing and antibody cloning. (**b-c**) Heatmap for pair-wise SPR competition matrices of anti-RU15-1 RBD (b) and anti-MO1 RBD (c) mAbs, with darker blue indicating stronger competition. Scores < 0 and > 1 shown as 0 and 1, respectively. Corresponding IC_50_ of mAbs are represented as red bars on the left of each heatmap, with darker red indicating the highest neutralizing activity. (**d**) Blockage of MO1/RU15-1 RBD–hFc binding (green) to *E.amu* APN transiently expressed in HEK293T cells by indicated antibodies (10 μg ml^−^^1^) and analyzed by immunofluorescence. Antibody IC_50_ values derived from a subset of the experimental data in Fig. 5c. Data are representative of two independent blockage experiments with similar results. (**e**) Blockage of MO1 (left)/ RU15-1 (right) RBD–hFc binding to *E.amu* APN transiently expressed in HEK293T cells by indicated antibodies (10 μg ml^−1^) and analyzed by flow cytometry. (**f**) Neutralization of EriCoV pseudovirus entry by indicated antibodies in HEK293T cells transiently expressing *E.amu* APN. BSA: Bovine serum albumin, 10 μg ml^−1^. Data are presented as mean ± s.d. for f. Statistical significance was determined using unpaired two-tailed *t*-tests (n = 3 independently infected wells). *P < 0.05, **P < 0.01, ***P < 0.001, ****P < 0.0001, and NS: not significant. Scale bars: 100 μm. Source data

To develop EriCoV-specific neutralizing antibodies, mice were immunized with MO1 and RU15-1 RBDs (Fig. 5a), which share 79% sequence identity. Enzyme-linked immunosorbent assay (ELISA) confirmed robust responses after two immunizations (Fig. 5b). Single B cells were sorted from mice with the highest titre for antibody discovery (Extended Data Fig. 8a), yielding 54 MO1-reactive and 67 RU15-1-reactive monoclonal antibodies (Supplementary Table 4). Neutralizing evaluation revealed potent activities among 36 of 54 (67%) MO1-targeting and 8 of 67 (12%) RU15-1-targeting antibodies (Fig. 5c).

To define the relationship between epitope and neutralization, antibodies were subjected to SPR-based epitope binning. They segregated into two distinct epitope bins, only one of which correlated with potent pseudovirus neutralization (Extended Data Fig. 8b,c). APN blockade assays using representative antibodies demonstrated a close correlation between neutralization and inhibition of recombinant RBD–hFc binding to *E.* *amurensis* APN (Extended Data Fig. 8d,e). Eight potent neutralizing antibodies were further validated against rcVSV-MO1, rcVSV-RU15-1 or rcVSV-IT5-12 amplification. Two RU15-1 antibodies, BD23-0177 and BD23-0163, additionally neutralized MO1 (Fig. 5d). BD23-0177 displayed broad neutralization potential, efficiently blocking entry of various EriCoV strains (Extended Data Fig. 8f).

**Extended Data Fig. 9:**
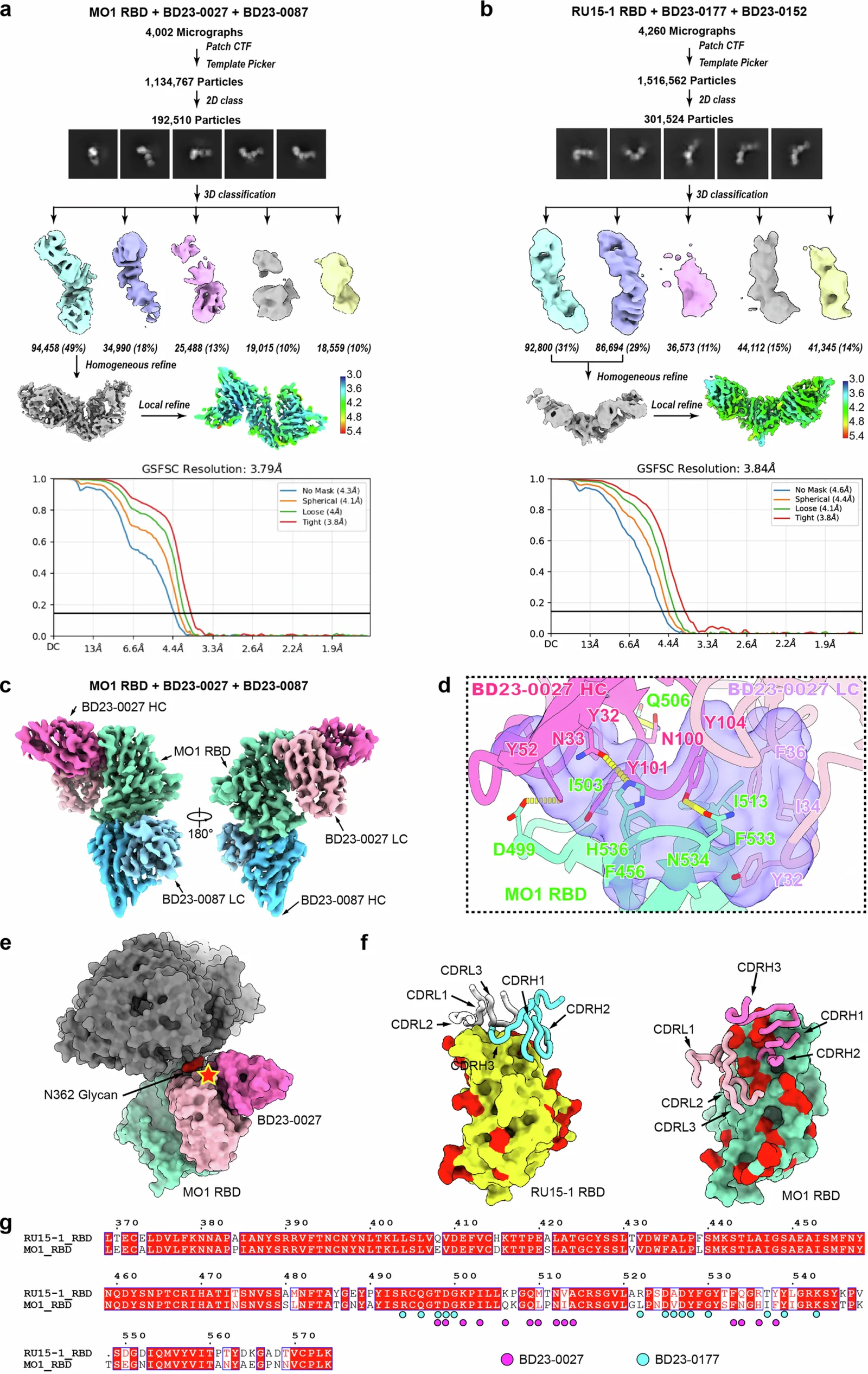
Cryo-EM analysis of the neutralizing mechanism of EriCoV RBD-specific antibodies. (**a-b**) Flowcharts for structure determinations of MO1 RBD bound to BD23-0087 and BD23-0027 (a), and RU15-1 RBD bound to BD23-0177 and BD23-0152 complex (b). Gold-standard Fourier shell correlation (FSC) curves are shown to assess map quality and resolution. Local resolution assessments of cryo-EM maps using rainbow are shown. (**c**) The overall structures of the MO1 RBD bound to BD23-0027 and BD23-0087. MO1 RBD is shown in medium aquamarine. The heavy chain and light chain of BD23-0027 are colored hot pink and light pink, respectively. Those of BD23-0087 are deep sky blue and light sky blue. (**d**) Interaction interface between MO1 RBD and BD23-0027. Key residues are rendered as sticks. The hydrophobic pocket and hydrogen bonds are colored semi-transparent blue and yellow dashed lines, respectively. (**e**) The structural basis of the BD23-0027 neutralization. The structures are shown as surface, and steric clashes are highlighted by red pentagrams. The *E.amu* APN, MO1 RBDs, and Fabs are colored as described previously. (**f**) Detailed interactions between the CDRs of BD23-0177 and BD23-0027 with the RU15-1 RBD (left) and MO1 RBD (right), respectively. Close-up views show the interaction interfaces, with the RU15-1 and MO1 RBDs displayed in surface representation and the antibody CDRs shown in cartoon representation. The RU15-1 and MO1 RBDs are colored yellow and medium aquamarine, respectively. Residues that differ between RU15-1 and MO1 RBDs are highlighted in red. The colors of the antibody heavy and light chains are consistent with those described previously. (**g**) Sequence alignment of RU15-1 and MO1 RBDs. Residues within 4 Å of RU15-1 RBD bound to BD23-0177 and MO1 RBD bound to BD23-0027 are indicated by cyan circles and magenta circles, respectively.

To define the neutralization mechanisms, we structurally characterized BD23-0177/BD23-0152 and BD23-0027/BD23-0087 as representative RU15-1 and MO1 monoclonal antibodies (mAbs), with reconstructions determined at 3.84 and 3.79 Å, respectively (Extended Data Fig. 9a,b). BD23-0177 recognizes the RU15-1 RBM tip, with residues T537, Y539, R494 and D525 positioned near the antibody interface and potentially contributing to polar interactions. In addition, heavy-chain residues Y32/Y99/V98/V102, light-chain residues H91/A32 and RU15-1 Y528/Y539 form a hydrophobic interface that likely contributes to RU15-1 recognition, thereby sterically blocking APN engagement (Fig. 5e–g and Supplementary Table 5). By contrast, BD23-0027 targets a rear-left epitope on the MO1 RBD tip, likely neutralizing MO1 by clashing with the *E.* *amurensis* APN N362 glycan (Extended Data Fig. 9c–e). Notably, BD23-0177 recognizes a relatively conserved RU15-1/MO1 RBD epitope, accounting for its broader neutralization (Extended Data Fig. 9f,g).

**Extended Data Fig. 10:**
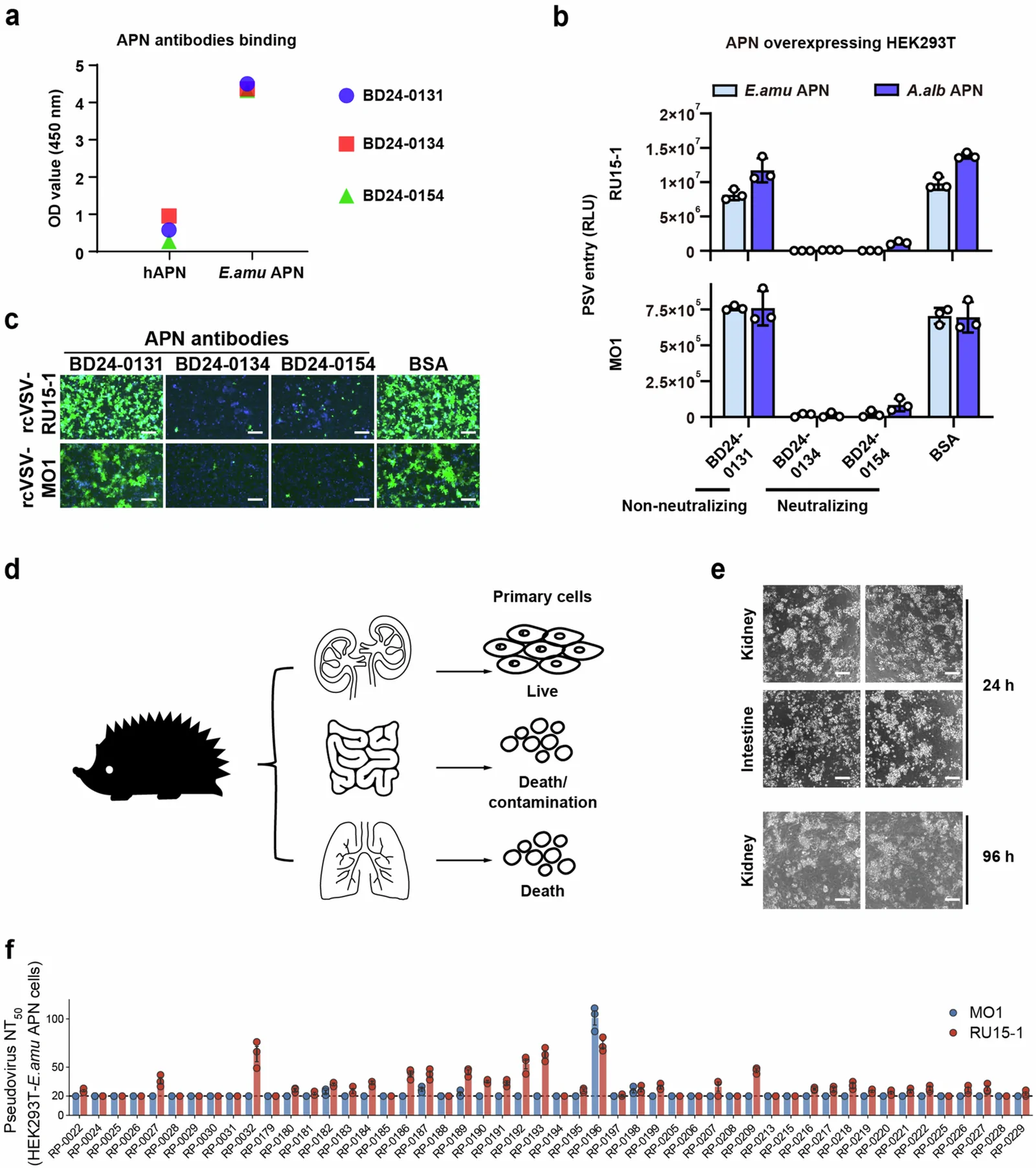
Antibodies block EriCoV pseudovirus entry in APN overexpressing human cell lines and hedgehog primary cells. (**a**) ELISA analysis of the binding efficiency of three indicated *E.amu* APN-targeting antibodies to immobilized human APN or *E.amu* APN. (**b**) Entry of RU15-1 and MO1 pseudovirus in HEK293T cells transiently expressing *E.amu* or *A.alb* APN in the presence of 10 μg ml^−1^ neutralizing (BD24-0134, and BD24-0154) or non-neutralizing (BD24-0131) antibodies. Mean ± s.d. (n = 3 independently infected wells). BSA: 10 μg ml^−1^ Bovine serum albumin for b. (**c**) Neutralizing activity of *E.amu* APN antibodies (10 μg ml^−1^) against rcVSV-RU15-1 or rcVSV-MO1 in Caco-2 cells overexpressing *E.amu* APN and *E.amu* TMPRSS2. Data are representative of two independent experiments with similar results. (**d-e**) Primary cells derived from the kidney and intestine of the African pygmy hedgehogs (*Atelerix albiventris, A.alb*). (**f**) 50% Neutralization titer (NT_50_) of plasma samples from random human donors against MO1 and RU15-1 pseudoviruses, determined using HEK293T-*E.amu* APN cells. Dashed lines indicate the assay baseline. mean  ± s.e.m. (n = 3 independent experiments). Scale bars: 200 μm for c, 100 μm for e. Source data

To investigate APN-targeting antibodies, mice were immunized with *E.* *amurensis* APN mRNA, and APN-specific single B cells were sorted after boosting (Fig. 5h). In total, 83 antibodies were obtained (Supplementary Table 4), over half of which strongly neutralized EriCoV pseudovirus entry (Fig. 5i). Two neutralizing antibodies (BD24-0134 and BD24-0154) and one non-neutralizing antibody (BD24-0131) were selected. BD24-0134 and BD24-0154 do not recognize human APN and blocked RU15-1 and MO1 entry and propagation in cells expressing *E.* *amurensis* or African pygmy hedgehog (*A.* *albiventris*) APNs (Extended Data Fig. 10a–c). As Amur hedgehog cells were unavailable, primary African pygmy hedgehog kidney cells were tested. EriCoV, but not VSV-G, pseudovirus entry was specifically inhibited by the neutralizing *E.* *amurensis* APN antibodies, confirming that endogenous APN is essential for viral entry (Fig. 5j and Extended Data Fig. 10d,e).

Together, these neutralizing antibodies represent promising countermeasures for outbreak preparedness, given the negligible cross-neutralizing activity elicited by prior SARS-CoV-2 infection or vaccination (Extended Data Fig. 10f and Supplementary Table 6).

## Discussion

Coronaviruses circulating in wildlife continue to pose a threat of future pandemics. EriCoVs, relatives of MERS-CoV, have been known for over a decade and are now recognized as genetically diverse and widely distributed^11^. Unlike bats, hedgehogs frequently interact with humans as urban wildlife or pets^51^. However, the knowledge gap of EriCoV receptor usage and entry mechanism has limited assessment of spillover risk and pathogenesis and the development of countermeasures.

DPP4 was initially identified as a MERS-CoV receptor^20^ and long considered the canonical receptor for merbecoviruses. However, the recent discovery of ACE2 usage by NeoCoV and several additional merbecovirus lineages revealed substantial diversity of receptor usage within this subgenus. Our study, together with a recent independent study by Jin et al.^52^, identified APN as a functional receptor for EriCoVs, unexpectedly expanding APN usage to the betacoronavirus genus, providing convergent evidence that EriCoVs have evolved a distinct APN-binding strategy.

Prior to this work, three APN-binding modes had been described among alpha- and deltacoronaviruses. Our findings add another, structurally distinct APN-binding mode, highlighting the remarkable plasticity of coronavirus RBDs in adapting to diverse receptor surfaces^26^. Furthermore, our study defines host-range determinants, explores protease requirements for entry and characterizes neutralizing antibodies, providing a complementary perspective to the work by Jin et al.^52^.

Intriguingly, the three merbecovirus receptors—ACE2, DPP4 and APN—are all ectopeptidases expressing on both epithelial surfaces of the respiratory and gastrointestinal tracts. The repeated exploitation of ectopeptidases may reflect common evolutionary advantages, such as accessibility, tissue distribution and favourable structural properties for virus–receptor interactions^53^.

Despite structural and sequence homology between NeoCoV and EriCoV, their distinct receptor usage implies receptor switching during their evolutionary divergence. The mechanisms underlying this transition remain unclear. Recombination, RBM insertions/deletions and antigenic drift are likely contributors^31,54^. These findings also illustrate the limitations of predicting coronavirus receptor usage solely from sequence similarity or structural modelling.

Consistent with the study by Jin et al.^52^, our results show that EriCoVs are highly adapted to hedgehog APN and display restricted receptor tropism. Outside hedgehogs, only a limited number of APN orthologues—most notably from felids—support detectable receptor function. This restriction is governed by a small number of residues in APN domain 2, including glycosylation, suggesting that current EriCoVs face substantial barriers to cross-species transmission. Although our data suggest a relatively low spillover risk through natural hedgehog predators, such as badgers, limited compatibility with felid APN indicates that modest changes at the receptor-binding interface could lower the host barrier. In addition, hedgehogs can harbour other coronaviruses, such as *Alphacoronavirus miniopteri* (BtCoV-HKU8)^11^, raising the possibility of future recombination events that could drive EriCoV evolution. Therefore, continued surveillance of the presence and viral sequence changes in hedgehogs, felids and other ecologically relevant species will be critical for mitigating future spillover risks.

Beyond receptor recognition, proteolytic activation represents an additional critical determinant of EriCoV entry. Extracellular soluble proteases, such as trypsin, may play a major role in spike activation, whereas TMPRSS2 and endosomal cathepsins contribute in a context-dependent and strain-specific manner. This flexibility resembles that observed in several other coronaviruses and suggests that multiple entry routes may be available depending on host species and tissue environment^36,44^. The strong dependence on trypsin-like proteases is consistent with the predominantly enteric distribution and supports a potential faecal–oral transmission route^45^, whereas adaptation to other proteases, such as TMPRSS2, could facilitate shifts in tissue tropism and pathogenicity^55^. Nevertheless, these observations should be interpreted with caution as the experimental systems used may not fully recapitulate authentic infection.

Overall, our findings expand the receptor repertoire of merbecoviruses and provide a structural and functional framework for risk assessment and countermeasure development for EriCoVs that could potentially pose a threat to domestic animals or humans.

## Methods

### Ethical statement

All mouse-immunization-related experiments were conducted in accordance with protocols approved by the Animal Welfare Ethics Committee of HFK Biologics with an approval number of HFK-AP-20250313. All hedgehog-related experiments were authorized by the Institutional Animal Care and Use Committee (IACUC) of the Institute of Laboratory Animal Science (ILAS), Chinese Academy of Medical Sciences (CAMS) & Peking Union Medical College (PUMC), with an approval number of BLL25006. All procedures were conducted in strict compliance with institutional and national ethical guidelines for animal welfare.

Plasma samples for neutralization assays were collected from a random population of plasma donors at Beijing Ditan Hospital, Capital Medical University. All participants provided written informed consent before sample collection. Participants received reimbursement for transportation expenses related to blood sample collection. The study was approved by the Ethics Committee of Beijing Ditan Hospital, Capital Medical University with an approval number of DTEC-KY2024-112-01. Demographic data for the plasma donors are presented in Supplementary Table 6.

### Biosafety statement

All viral entry and amplification assays in this study were performed with VSV pseudoviruses or rcVSVs expressing coronavirus spike proteins (rcVSV-S). The experiments involving rcVSV-S were authorized by the Biosafety Committee of the State Key Laboratory of Virology and Biosafety, Wuhan University and conducted according to standard operating procedures in a Biosafety Level 2 laboratory with an approval number of GC2025-006. No authentic coronavirus was used in this study.

### Cell lines and culture conditions

HEK293T (CRL-3216), Caco-2 (HTB-37), BHK-21 (CCL-10) and I1-Hybridoma (CRL-2700) cells were obtained from the American Type Culture Collection. HEK293T, BHK-21 and Caco-2 cells were cultured in Dulbecco’s Modified Eagle Medium (DMEM; Monad) supplemented with 10% fetal bovine serum (FBS; Excell), 1% penicillin-streptomycin (Thermo Fisher Scientific) at 37 °C in a humidified incubator with 5% CO_2_. The I1-Hybridoma cell line, which produces a neutralizing antibody against VSV glycoprotein (anti-VSV-G monoclonal antibody, I1), was maintained in Minimum Essential Medium (Gibco) with Earle’s balanced salts, 2.0 mM L-glutamine (Gibco) and 10% FBS. HEK293T or Caco-2 stable cell lines overexpressing various receptors were generated using lentivirus transduction and then selected and maintained in growth medium with puromycin (1 μg ml^−1^; Yuanye, China, S17055) or Zeocin (100 μg ml^−1^; Invivogen, ant-zn-1).

### Receptor sequence retrieval and assembly

The nucleic acid sequences of *E.* *amurensis* ACE2, *E.* *amurensis* DPP4, *E.* *amurensis* APN, *E.* *amurensis* TMPRSS2, *Hylomys suillus* APN and *A.* *albiventris* APN were assembled from RNA-seq raw data derived from *Erinaceus amurensis* Kidney (Genome Sequence Archive (GSA) database, GSA accession ID: CRX555648), *E.* *amurensis* lymph (Sequence Read Archive (SRA) accession: SRX13651754), *Hylomys suillus* muscle/liver tissue (SRA accession: SRR6053059) or *A.* *albiventris* kidney (GSA accession ID: CRX911825). Briefly, raw next-generation sequencing reads were downloaded from the GSA and SRA databases and processed using Cutadapt (v2.4) to remove adapters and discard reads shorter than 30 base pairs. De novo assembly was performed with MEGAHIT (v1.2.9), using a minimum contig length of 100 bp. Resulting final contigs were analysed using Geneious (2023.0.1) and were subjected to Geneious workflows for mapping reads to the indicated reference sequences under default parameters.

### Plasmids and vectors

Plasmids for expressing coronavirus receptors or their derivatives were constructed by inserting human codon-optimized coding sequences obtained through commercial synthesis into the lentiviral transfer vector (pLVX-EF1α-Puro, Genewiz) with C-terminally fused 3× FLAG tags (DYKDHD-G-DYKDHD-I-DYKDDDDK)^17^. Human furin, human cathepsin L, human TMPRSS2 and *E.* *amurensis* TMPRSS2 expressing plasmids were constructed to pLVX-EF1α-Zeocin (clone based on pLVX-EF1α-Puro with the resistance gene replaced with Zeocin) with C-terminally fused ALFA tag (PSRLEEELRRRLTE)^56^. DNA fragments for cloning chimera or mutants were generated by overlap extension polymerase chain reaction (PCR) or gene synthesis and verified by commercial DNA sequencing.

For pseudovirus production, human codon-optimized spike sequences of 229E (AAK32191.1), FCoV-23 (obtained from a previous study^57^), CCoV-HuPn-2018 (QVL91811.1), TGEV (ABG89335.1), PDCoV (QWE80500.1), MO1 (UMO75628.1), F6 (QGA70692.1), cw_6 (QOQ34381.1), IT8-1 (QRN68024.1), IT5-19 (QRN68031.1), IT5-17 (QRN68055.1), IT5-1 (QRN68048.1), IT5-11 (QRN68066.1), IT5-12 (QRN68078.1), IT5-15 (QRN68101.1), GER216 (AGX27810.1), GER174 (YP009513010), UK19 (QCC20713.1), PTH9 (XAJ09624.1), RU15-1 (XCN28775.1), RU15-2 (XCN28786.1), PLJ24 (XAJ09723.1), PLJ64 (XAJ09735.1), FR7137 (XNJ65105.1), FR3151 (XNJ65093.1), MOS-3 (XQP08644.1), MOS-17 (XQP08624.1), HUN-ER17 (YBK88845.1), NeoCoV (AGY29650.2) and MERS-CoV (YP_009047204.1) were cloned into the pCAGGS vector with 13–15 C-terminal residues replaced by an HA tag (YPYDVPDYA) to facilitate S incorporation^58^.

Plasmids expressing recombinant proteins, including coronavirus antigens, and soluble viral receptor proteins are described as follows. For coronavirus RBD–hFc fusion proteins, plasmids were constructed by inserting RBD coding sequences from RU15-1 (residues 366–574), MO1 (residues 366–575), PTH9 (residues 367–576), PLJ64 (residues 365–574), GER216 (residues 365–574), PLJ24 (residues 365–574), FR7137 (residues 362–571), FR3151 (residues 363–572), UK19 (residues 364–573), IT8-1 (residues 365–573), IT5-1 (residues 365–573), IT5-12 (residues 365–573), IT5-15 (residues 366–575), IT5-17 (residues 366–575), IT5-19 (residues 366–575), cw_6 (residues 366–575), F6 (residues 366–575) or 229E (residues 293–434) into the pCAGGS vector containing an N-terminal CD5 secretion signal peptide (MPMGSLQPLATLYLLGMLVASVL) and C-terminal hFc-Twin-Strep-3× FLAG tags (WSHPQFEKGGGSGGGSGGSAWSHPQFEK-GGGRSDYKDHDGDYKDHDIDYKDDDDK) for purification and detection. For expressing soluble viral receptor proteins, plasmids were constructed by inserting ectodomain coding sequences from *E.* *amurensis* APN (residues 60–960) or human APN (residues 66–967) into the pCAGGS vector containing an N-terminal CD5 secretion signal peptide (MPMGSLQPLATLYLLGMLVASVL) and C-terminal 8× His tags for purification.

For cryo-EM and BLI assays, DNA sequences encoding *E.* *amurensis* APN protein (residues 60–960) and RU15-1 RBD (residues 366–574; GenBank: XCN28775.1) were obtained from the National Center for Biotechnology Information (NCBI). An N-terminal CD5 signal peptide and a C-terminal His-tag were added to both constructs via PCR. The DNA sequence encoding the full-length RU15-1 spike protein (residues 1–1262; GenBank: XCN28775.1) was obtained from NCBI. A C-terminal T4 fibritin trimerization motif (foldon), followed by an HRV 3C protease cleavage site and a Twin-Strep tag, were added via PCR. These constructs were cloned into the pCAGGS mammalian expression vectors.

Importantly, no stabilizing mutations (such as proline substitutions) were introduced in the spike constructs. Therefore, the predominance of the closed conformation observed in our cryo-EM structure is unlikely to be an artefact of engineered stabilization, but rather reflects an intrinsic conformational property of the RU15-1 spike under the experimental conditions.

### Primary hedgehog cells

An African pygmy hedgehog (*A.* *albiventris*, 12 months of age, female) was purchased from a licensed commercial pet supplier in Beijing, China, on 11 October 2025. The animal was outbred, commercially bred individuals with the standard WT coat colouration. Before the experiment, the animal was clinically healthy and tested negative for common pathogens of this species, including *Salmonella* spp., dermatophytes, ectoparasites and intestinal parasites. All primary cells used in this study were derived from a single individual. For animal anaesthesia, tiletamine–zolazepam (Virbac) was administered intramuscularly at a dose of 15 mg per kg body weight before euthanasia. According to the product label and relevant veterinary advice, a pilot experiment was conducted using 10 mg per kg (the lower limit of this range) as the starting dose^59^. The dose was then titrated according to the depth of anaesthesia, the animals’ stress responses and the operational requirements of tissue collection, and a final dose of 15 mg per kg was established for the formal experiments. Following confirmation that the animal had achieved a stable, deep surgical plane of anaesthesia, terminal exsanguination was performed to complete euthanasia, followed by surgical dissection and tissue collection. All procedures were conducted in strict compliance with institutional laboratory animal care and use guidelines and applicable animal welfare standards.

The primary kidney cells were isolated from one animal using the Multi Tissue Dissociation Kit 1 (Miltenyi, 130-110-201) following the manufacturer’s instructions. Briefly, the tissue pieces were placed in RPMI 1640, and appropriate amounts of enzymes A, D and R were added sequentially. The tissue was then cut into small fragments (2–4 mm) with scissors and incubated at 37 °C with rotation for 30 min. After thorough trituration, the suspension was passed through a 70-µm cell strainer. The filtrate was collected and centrifuged (300*g*, 7 min), and the supernatant was discarded. Finally, 1 ml of PBS (0.04% bovine serum albumin (BSA)) was added, the cells were gently resuspended, and cell viability was assessed with Trypan blue staining. Cells were attached in collagen-coated 96-well plates and cultured in a growth medium (DMEM-F-12 medium supplemented with 1× insulin-transferrin-selenium, 1× Minimum Essential Medium nonessential amino acids, 0.1 μM hydrocortisone, 0.005 μg ml^−1^ epidermal growth factor, 100 IU ml^−1^ penicillin, 100 μg ml^−1^ streptomycin and 10% FBS) at 37 °C before infection. Bright-field images of primary hedgehog cells were acquired using a Nikon ECLIPSE Ti2 microscope.

### Protein expression and purification

Proteins for binding, pull-down or flow cytometry assays were produced in HEK293T cells via transient transfection, following the manufacturer’s guidelines. Supernatants were collected every 48 h after transfection. Twin-Strep-tagged proteins were enriched using Strep-Tactin XT 4Flow high-capacity resin (IBA, 2-5030-002), washed and eluted with buffer BXT (100 mM Tris/HCl, pH 8.0, 150 mM NaCl, 1 mM EDTA, 50 mM biotin). Eluted proteins were concentrated, buffer-exchanged into PBS by ultrafiltration, aliquoted and stored at −80 °C.

Protein expression and purification were conducted following the established method for cryo-EM or BLI analysis^60,61^. Briefly, the plasmids of these proteins were transiently transfected into suspension-grown HEK293F maintained at 37 °C with 8% CO_2_, rotating at 130 rpm. Supernatants were collected 72 h after transfection, concentrated and purified sequentially by Ni-NTA affinity chromatography and size-exclusion chromatography using a Superdex 200 Increase 10/300 GL column (GE Healthcare) equilibrated with PBS buffer.

### ELISA

ELISA plates were precoated with RBD protein from either strain MO1 or RU15-1 at a concentration of 1 μg ml^−1^ in 0.05 M coating buffer (Solarbio, C1055) and incubated overnight at 4 °C. After washing, plates were blocked, followed by the addition of 100 μl serially diluted mouse serum to each well and incubation at room temperature for 30 min. Plates were then washed and incubated with peroxidase-conjugated AffiniPure goat anti-mouse IgG (H + L) (Jackson ImmunoResearch, 115-035-003, 1:4,000) at 0.25 μg ml^−1^ for 30 min at room temperature. The reaction was developed using tetramethylbenzidine substrate (Solarbio, PR1200) and stopped by the addition of 2 M H_2_SO_4_. Absorbance was measured at 450 nm using a microplate reader (Thermo Scientific, Multiskan Fc).

### Animal immunization and antibody screening

Thirty female BALB/c mice aged 6–8 weeks were used in the experiments. Mice were housed under specific pathogen-free conditions with a 12-h light/dark cycle, an ambient temperature of 20–26 °C and a relative humidity of 40–70%. Animals were group-housed (five mice per cage) and had ad libitum access to sterilized chow and water throughout the study period. Mice were immunized according to the schedule outlined in Fig. 5a. Specifically, mice in the MO1 and RU15-1 RBD protein groups were immunized via subcutaneous injection with 10 µg of purified RBD protein from either the MO1 strain or RU15-1 strain, each formulated with Freund’s adjuvant. The primary immunization was administered with complete Freund’s adjuvant, followed by a booster injection formulated with incomplete Freund’s adjuvant 14 days later. Blood samples were collected 4 days after the second immunization.

Mice in the *E.* *amurensis* APN group were immunized via intramuscular injection with 10 µg of *E.* *amurensis* APN mRNA on day 0, day 14 and day 28, followed by a booster injection with 10 µg of purified *E.* *amurensis* APN protein formulated with incomplete Freund’s adjuvant on day 35. Blood samples were collected 4 days after the second immunization and 4 days after the third immunization. Serum titres were determined using ELISA.

Spleens from euthanized mice were minced in RPMI 1640 medium supplemented with 5% FBS, filtered through a 100-μm mesh and treated with 1× red blood cell lysis buffer (eBioscience, catalogue no. 00-4333-57). After centrifugation (350*g*, 8 min), cells were resuspended in staining buffer (PBS + 2% FBS) and adjusted to a final concentration of 1 × 10^7^ cells ml^−1^. Recombinant biotinylated RBD proteins (MO1 or RU15-1 strain) or *E.* *amurensis* APN proteins were incubated with streptavidin-conjugated fluorophores (PE and APC, BioLegend) on ice for at least 30 min. Then H_2_O was added to adjust volume, and the complexes were centrifuged at 14,000*g* for 10 min at 4 °C to remove aggregates. Before staining, splenocytes were incubated with TruStain FcX (BioLegend) for 10 min on ice in the dark to block Fc receptors. Antigen probes and fluorescently conjugated antibodies (FITC-CD19 antibody, BioLegend, catalogue no. 115506, 1:500; PE/Cyanine7-IgD antibody, BioLegend, catalogue no. 406514, 1:500; PE/Cyanine7-IgM antibody, BioLegend, catalogue no. 405720, 1:500) were then added, and cells were incubated on ice for 30 min in the dark. After staining, cells were washed twice with staining buffer (400*g*, 5–8 min) and resuspended in cold buffer. 7-AAD (eBioscience, catalogue no. 00-6993-50) was added 10 min before flow cytometry to exclude dead cells. For cell sorting, antigen-specific B cells were defined as 7-AAD^−^, CD19^+^, IgM^−^/IgD^−^, OVA^−^ and either MO1 RBD^+^, RU15-1 RBD^+^ or *E.* *amurensis* APN^+^, depending on the immunization group. Single B cells were sorted using a MoFlo Astrios EQ Cell Sorter (Beckman Coulter) directly into 96-well PCR plates.

Sorted single B cells were lysed in buffer containing RNase inhibitor, 0.095% Triton X-100, Oligo dT primer and dNTPs, followed by cDNA synthesis using the Vazyme R302 kit. Variable regions of heavy chains and light chains (lambda/kappa) were amplified via two-round PCR. After verification by 1% agarose gel electrophoresis and magnetic bead purification, the variable region amplicons were set aside. Separately, the human IgG1 constant region was cloned into pCMV3 vectors using homologous recombination with the ClonExpress II One Step Cloning Kit (Vazyme, catalogue no. C112-02) at 37 °C for 30 min. Variable regions were recombined into the pCMV3 vectors using the same homologous recombination method. Recombinant plasmids were transformed into DH5α competent cells (Tsingke, catalogue no. TSC-C01), activated at 37 °C and plated for selection. Plasmid DNA of expanded cultures was extracted (CWBIO CW2105) after being verified by the alignment results of Sanger sequencing. Protein production was carried out in Expi293F cells. Cotransfection was performed by mixing the antibody plasmids with polyethylenimine (Yeasen, catalogue no. 40816ES03) in 0.9% NaCl solution before adding to the cell culture. Cells were maintained under standard culture conditions. After 24 h, 1 ml of nutrient supplement (OPM Biosciences, catalogue no. F081918-001) was added per culture bottle, with subsequent feedings every 48 h during a 6–10 day culturing period. For antibody purification, supernatant containing the monoclonal antibodies was obtained by centrifugation (3,000*g*, 10 min). After incubation of Protein A magnetic beads (GenScript, L00695) with the supernatant for 2 h, automated purification was achieved by using a KingFisher instrument (Thermo Fisher). Antibody concentration was measured with a NanoDrop (Thermo Fisher, catalogue no. 840-317400), and purity was assessed by sodium dodecyl sulfate–polyacrylamide gel electrophoresis (SDS–PAGE) (LabLead, P42015). Screening of monoclonal antibodies was performed using a pseudovirus neutralization assay. The antibody sequences are listed in Supplementary Table 4.

### SPR competitive binding assay

Antibodies were assessed using the Biacore 8K system (GE Healthcare). For competitive binding experiments, His-tagged MO1 or RU15-1 RBD proteins were immobilized on anti-His sensor chips (Cytiva) at a concentration of 1.35 μg ml^−1^ for 1 min. Pairs of antibodies (37.5 μg ml^−1^ each) were then sequentially injected over the chip surface, with each injection lasting 2 min. The chip surface was regenerated with 1.5 M glycine. Competition between antibodies was quantified using the following score: score_*b−a*_ = 1 − response_*b*_/response^′^_*b*_, where response_*b*_ represents the response units (RU) when antibody *b* was injected as the second antibody following antibody *a* and response′_*b*_ represents the mean RU when antibody *b* was injected as the first antibody.

### Antigen-hFc live-cell binding assay

HEK293T cells transiently expressing distinct receptors or orthologous APNs were incubated 48 h after transfection with recombinant RBD–hFc proteins (2 µg ml^−1^ diluted in SMM 293-TII Expression Medium) for 30 min at 37 °C (5% CO_2_). Cells were gently washed once with Hanks’ Balanced Salt Solution (HBSS) and then stained for 1 h at 37 °C with Alexa Fluor 488-conjugated goat anti-human IgG (Thermo Fisher Scientific, A11013, 1:1,000) and Hoechst 33342 (Thermo Fisher Scientific, 62249, 1:2000) nuclear counterstain (1:10,000 in HBSS). Both reagents were prepared in HBSS containing 1% BSA. After staining, cells were washed twice with HBSS and imaged. Fluorescence microscopy used an MI52-N system (Mshot). Relative fluorescence intensities were quantified using a Varioskan LUX multimode plate reader (Thermo Scientific).

For experiments of RBD-specific antibody competition, recombinant RBD–hFc proteins (2 µg ml^−1^) and specific RBD-targeting antibodies (10 µg ml^−1^) were mixed in 50 µl SMM 293-TII Expression Medium for 1 h at 37 °C and then applied to HEK293T cells transiently expressing *E.* *amurensis* APN to evaluate RBD–hFc binding efficiency. RBD–hFc was subsequently stained with Alexa Fluor 488-conjugated goat anti-human IgG (Thermo Fisher Scientific, A11013, 1:1,000) for live-cell immunofluorescence and goat anti-human Fc conjugated with DL594 (Thermo Fisher Scientific, catalogue no. SA5-10136, 1:1,000) for flow cytometry.

### Flow cytometry

To analyse RBD–hFc binding by flow cytometry, HEK293T cells transiently expressing the indicated APN orthologues were detached with 5 mM EDTA/PBS 24 h after transfection. For antibody–APN competition assays, RU15-1 or MO1 RBD–hFc proteins were pretreated with indicated antibodies (10 μg ml^−1^) at 37 °C for 1 h. The detached cells were incubated with RU15-1 or MO1 RBD–hFc proteins (2 µg ml^−1^) at 4 °C for 1 h. After two PBS washes, cells were stained for 1 h at 4 °C using DyLight 594-conjugated goat anti-human IgG antibodies (Thermo Fisher Scientific, catalogue no. SA5-10136, 1:1,000) to detect RBD binding. Following two additional PBS washes, 15,000 live cells for RBD–hFc binding assays or 10,000 live cells for antibody–APN competition assays (gated based on forward scatter and side scatter) were analysed on a CytoFLEX flow cytometer (Beckman) (Supplementary Fig. 6).

### EriCoV spike cleavage assay with exogenous proteases

Pseudovirus-containing supernatants were concentrated 20-fold through a 30% sucrose cushion (30% sucrose, 15 mM Tris-HCl, 100 mM NaCl, 0.5 mM EDTA) by centrifugation at 20,000*g* for 1 h at 4 °C. Pellets were resuspended in DMEM and incubated overnight at 4 °C for sufficient resolubilization. Pseudoviruses were then digested with the indicated concentrations of exogenous proteases including trypsin (Sigma-Aldrich, catalogue no. T4549), TPCK-treated trypsin (Sigma-Aldrich, catalogue no. T8802), elastase (Sigma-Aldrich, catalogue no. E7885), plasmin (Sigma-Aldrich, catalogue no. P1867) and factor Xa (NEB, catalogue no. P8010S) at 37 °C for 1 h. Reactions were terminated by the addition of 1× SDS loading buffer and boiling at 100 °C for 15 min. Samples were subsequently subjected to SDS–PAGE under reducing conditions and immunoblotted with a mouse anti-HA antibody to detect spike cleavage. VSV-M served as a virion normalization control.

### Western blot

Western blotting was performed to assess S incorporation into VSV pseudoviruses. Pseudovirus-containing supernatants were concentrated through a 30% sucrose cushion by centrifugation at 20,000*g* for 1 h at 4 °C. Pellets were resuspended in 1× SDS loading buffer, boiled at 95 °C for 30 min and subjected to SDS–PAGE and immunoblotting. VSV-M served as a virion normalization control.

To evaluate expression of different EriCoV spike or APN orthologues, HEK293T cells were transfected for 24 h, lysed in 1% Triton X-100 at 4 °C for 10 min and clarified by centrifugation at 12,000*g* for 5 min. Supernatants were mixed with 5× loading buffer and analysed by western blot. Membranes were blocked with 5% milk in PBS supplemented with 0.02% Tween-20 (PBST) for 1 h at room temperature, incubated overnight at 4 °C with anti-HA (MBL, catalogue no. M180-3, 1:5,000), anti-VSV-M (Kerafast, catalogue no. EB0011, 1:10,000), anti-β-tubulin (Immunoway, catalogue no. YM3030, 1:10,000), anti-β-actin (Yeasen, catalogue no. 30101ES50, 1:10,000) or anti-FLAG (Sigma-Aldrich, catalogue no. F1804, 1:10,000) and then probed with goat anti-mouse (Jackson, catalogue no. 115-035-003, 1:10,000) IgG secondary antibody for 1 h at room temperature. β-actin or β-tubulin served as a loading control. After four washes in PBST, signals were visualized using the Omni-ECL Femto Light Chemiluminescence Kit (EpiZyme, catalogue no. SQ201) and imaged on a ChemiDoc MP system (Bio-Rad).

### Immunofluorescence assay

Immunofluorescence assays were performed to assess the expression levels of APN orthologues with C-terminal fused 3× FLAG tags and cellular proteases with C-terminal fused ALFA tag. Cells expressing the receptors were seeded in the 96-well plates. Specifically, the transfected cells were fixed and permeabilized with 100% methanol for 10 min at room temperature. Then the cells were incubated with a mouse antibody M2 (Sigma-Aldrich, catalogue no. F1804, 1:1,000) or anti-ALFA antibodies (3 µg ml^−1^, in-house generated) diluted in HBSS/1% BSA for 1 h at 37 °C. After one HBSS wash, the cells were incubated with Alexa Fluor 594-conjugated goat anti-mouse IgG (Thermo Fisher Scientific, A32742, 1:1,000) or DyLight 594-conjugated goat anti-human IgG (Thermo Fisher Scientific, catalogue no. SA5-10136, 1:1,000) secondary antibody diluted in 1% BSA/PBS for one hour at 37 °C. The images were captured with a fluorescence microscope (Mshot, catalogue no. MI52-N) after the nucleus was stained blue with Hoechst 33342 reagent (1:2,000 in HBSS).

### Pull-down assay

For pull-down assays, recombinant viral RBD–hFc-Strep-FLAG and soluble *E.* *amurensis* APN-His or hAPN-His ectodomain proteins were expressed in eukaryotic cells and purified by affinity chromatography. Viral RBD–hFc-Strep-FLAG protein (1 μg ml^−1^) was incubated with 30 μl suspended Mag-Strep beads in 500 μl PBS at 4 °C for 1 h. Beads were washed three times with cold PBS and then incubated with 2 μg of *E.* *amurensis* APN-His or hAPN-His protein in 1 mL PBS at 4 °C for an additional hour. After washing three times with cold PBS, beads were resuspended in PBS, denatured in 1× SDS loading buffer and boiled for 20 min. Samples were analysed by SDS–PAGE followed by western blotting.

### BLI binding assay

Binding affinities were measured using BLI on an Octet RED96 instrument (Molecular Devices), following a protocol similar to that described above^62,63^. Briefly, recombinant MO1 and RU15-1 RBD-His proteins (50 μg ml^−1^) were immobilized onto Ni-NTA biosensors (ForteBio) for 300 s. Following loading, the biosensors were transferred to kinetics buffer (PBST) for 60 s to remove unbound proteins. The sensors were then immersed in PBST containing serial dilutions (0–10 μM) of soluble *E.* *amurensis* APN ectodomain (twin-Strep-tag) for 120 s to monitor the association phase, followed by a 120 s dissociation phase in PBST. Data acquisition and analysis were performed using Octet Acquisition 9.0 and Octet Analysis 12.2.0.20 software (ForteBio), respectively.

### Cell–cell fusion assays

A cell–cell fusion assay based on dual-split proteins was performed in HEK293T cells stably expressing *E.* *amurensis* APN, which were transiently transfected with spike and reporter gene expressing vectors by Lipofectamine 2000 (Biosharp, BL623E) following the manufacturer’s instructions. Two groups of cells were prepared to assess the S-mediated membrane fusion promoted by receptor interaction. Group A cells were transfected with plasmids expressing S and rLucN(1–155)-sfGFP1–7(1–157), and group B cells received plasmids encoding S and sfGFP8–11(158–231)-rLuc(156–311). At 12 h after transfection, both groups of cells were trypsinized, mixed and seeded into a 96-well plate at 8 × 10^4^ cells per well. At 24 h after transfection, the cells were washed twice with DMEM and then incubated with DMEM containing the indicated concentrations of Trypsin, from porcine pancreas (Sigma-Aldrich, catalogue no. T4549) or other exogenous protease for 30 min at 37 °C. Clumped cells remained viable and adherent to plates despite high trypsin concentrations. Protease activity was then quenched by replacing the medium with DMEM supplemented with 10% FBS and Hoechst 33342 (1:5,000) for nuclear staining. Syncytia formation with green fluorescence was assessed 1.5 h post medium change, and images were captured by an ELISpot reader (S6 Universal M2).

### Pseudovirus production

Pseudoviruses carrying coronavirus S glycoproteins were generated using a VSV-based pseudotyping system^17^. Briefly, HEK293T cells were transfected with plasmids encoding the S proteins using Lipofectamine 2000 (Biosharp, BL623E). After 24 h, the cells were infected with VSV-ΔG-fLuc-GFP (1 × 10^6^ 50% tissue culture infectious dose (TCID_50_) ml^−1^) in DMEM supplemented with 8 μg ml^−1^ polybrene and incubated for 2 h at 37 °C on a shaker. The inoculum was then replaced with fresh DMEM or SMM 293-TII Expression Medium (Sino Biological, catalogue no. M293TII) containing an anti-VSV-G monoclonal antibody (purified from I1-hybridoma supernatant, American Type Culture Collection, 1 μg ml^−1^) to neutralize residual input virus. After another 24 h, the supernatant containing pseudoviruses was collected, clarified by centrifugation at 13,523*g* for 5 min at 4 °C and stored at −80 °C until use.

### Single-round pseudovirus entry and inhibition assay

Single-round EriCoV pseudovirus entry assays were performed in HEK293T cells transiently or stably expressing various receptors. Cells were seeded in 96-well plates at 3 × 10^4^ cells per well and infected with pseudoviruses (1.5 × 10^5^ TCID_50_ in 100 μl) in the presence of the I1 anti-VSV-G neutralizing antibody to minimize background.

For proteolytic activation, pseudoviruses were pretreated with indicated concentrations of exogenous proteases for 1 h at 37 °C. Exogenous proteases included plasmin (Sigma-Aldrich, catalogue no. P1867), elastase (Sigma-Aldrich, catalogue no. E7885), trypsin (Sigma-Aldrich, catalogue no. T4549), TPCK-treated trypsin (Sigma-Aldrich, catalogue no. T8802) and factor Xa (NEB, catalogue no. P8010S). Then proteolytic cleavage was quenched with 10% FBS. For untreated controls, PBS was added instead.

For pseudovirus entry or entry inhibition assays, cells were incubated with 1.5 × 10^5^ TCID_50_ of pseudovirus at 100 μl per well. The incubation allowed for attachment and viral entry with or without the indicated concentrations of antibodies or other inhibitors. In some cases, Camostat (MedChemExpress, catalogue no. HY-13512), E64d (MedChemExpress, catalogue no. HY-100229), Z-FY-CHO (MedChemExpress, catalogue no. HY-128140), CA-074 (MedChemExpress, catalogue no. HY-103350), EK1C4, MM3122 (MedChemExpress, catalogue no. HY-148072) and MI-1851 (MedChemExpress, catalogue no. HY-150737) were used for entry inhibition. DMSO was used as vehicle control. Inoculated plates were centrifuged at 4 °C at 1,000*g* for 1 h. Then, 16–20 h after infection, intracellular luciferase activity was measured using the Bright-Glo Luciferase Assay System (Promega, catalogue no. E2620) on a Thermo Varioskan LUX or GloMax 20/20 Luminometer (Promega). For pseudovirus entry assays in primary hedgehog cells, luciferase activity was measured using the Bright-Lite Luciferase Assay Buffer (Vazyme, catalogue no. DD1209-03-AB) on a microplate spectrophotometer (PerkinElmer, catalogue no. HH3400).

Monoclonal antibodies or plasma samples were serially diluted in DMEM (Hyclone, catalogue no. SH30243.01) and incubated with pseudovirus in 96-well plates at 37 °C under 5% CO_2_ for 1 h. Trypsin-digested HEK293T-*E.* *amurensis* APN cells were then seeded into the plates and cultured for 24 h. After half the supernatant was discarded, reconstituted lyophilized Bright-Lite Luciferase Assay Substrate was mixed with Bright-Lite Luciferase Assay Buffer (Vazyme, catalogue no. DD1209-03-AB) before being added to the wells for incubation in the dark. Luminescence intensity was measured using a microplate spectrophotometer (PerkinElmer, model HH3400). The 50% inhibitory concentration (IC_50_) and the 50% neutralization titre were calculated via four-parameter logistic regression using PRISM software (version 9.0.1).

### rcVSV-CoV amplification and inhibition assay

Experiments involving replication-competent VSV-S (rcVSV-S) were approved by the Biosafety Committee of the State Key Laboratory of Virology and Biosafety of Wuhan University and performed under Biosafety Level 2 conditions. Plasmids for rescuing rcVSV-S expressing IT5-12, RU15-1, MO1 or cw_6 spike (S) glycoproteins were constructed by substituting the firefly luciferase (fLuc) coding sequence with coronavirus S gene sequences in the pVSV-ΔG-fLuc-GFP vector backbone^53^. In these recombinant viruses, the VSV-G gene was replaced with genomically encoded S, with a GFP reporter expression cassette for visualization. rcVSVs expressing IT5-12, RU15-1, MO1 or cw_6 S were generated via reverse genetics following a modified published protocol. Briefly, BHK-21 cells seeded at 80% confluence in six-well plates were infected with recombinant vaccinia virus expressing T7 RNA polymerase (vvT7 provided by M. Chen’s laboratory, Hubei University) at a multiplicity of infection of 5 for 45 min at 37 °C. Following infection, cells were transfected with pVSV-ΔG-GFP-S plasmids and helper plasmids (mass ratio: 5:3:5:8:1 for pVSV-ΔG-GFP-S:pBS-N:pBS-P:pBS-G:pBS-L). Supernatants containing rcVSV-S (designated P0) were collected 48 h after transfection, filtered (0.22 μm) to eliminate residual vvT7 and used to infect Caco-2 cells pretransfected with VSV-G for production of passage 1 (P1) viruses. To generate passage 2 (P2) viral particles carrying IT5-12, RU15-1, MO1 or cw_6 S glycoproteins, P1 viruses were used to infect Caco-2 cells stably expressing *E.* *amurensis* APN without ectopic VSV-G expression and in the presence of anti-VSV-G (I1-hybridoma supernatant).

For viral amplification or amplification inhibition assays, 3 × 10^4^ trypsinized Caco-2 cells expressing relevant APNs were seeded in 96-well plates and incubated with rcVSV-CoV (1 × 10^5^ TCID_50_ per 100 μl) in DMEM supplemented with 2% FBS, with or without 20 μg ml^−1^ TPCK-treated trypsin. Nuclei were stained with Hoechst 33342 (1:10,000 in HBSS) for 30 min at 37 °C. Fluorescence images were acquired at designated time points after infection using an MI52-N fluorescence microscope (Mshot).

For protease inhibition assays, Caco-2-*E.* *amurensis* APN or Caco-2-*E.* *amurensis* APN-*E.* *amurensis* TMPRSS2 cells were seeded in 96-well plates at a density of 3 × 10^4^ cells per well. After 12 h, cells were incubated with endogenous protease inhibitors Camostat (MedChemExpress, catalogue no. HY-13512), E64d (MedChemExpress, catalogue no. HY-100229), Z-FY-CHO (MedChemExpress, catalogue no. HY-128140), CA-074 (MedChemExpress, catalogue no. HY-103350), EK1C4, MM3122 (MedChemExpress, catalogue no. HY-148072) and MI-1851 (MedChemExpress, catalogue no. HY-150737) at indicated concentrations for 30 min at 37 °C. Subsequently, rcVSV-CoV (1 × 10^5^ TCID_50_ per 100 μl) was added to the target cells in DMEM supplemented with 10% FBS. After 36 h, nuclei were stained with Hoechst 33342 (1:10,000 in HBSS) for 30 min at 37 °C, and fluorescence images were acquired using an MI52-N fluorescence microscope (Mshot).

For antibody neutralization assays, Caco-2-*E.* *amurensis* APN-*E.* *amurensis* TMPRSS2 cells were seeded in 96-well plates at a density of 5 × 10^4^ cells per well. Twelve hours later, rcVSV-CoV was incubated with the indicated concentrations of antibodies in DMEM for 1 h at 37 °C and then added to the target cells in DMEM containing 10% FBS. At the indicated time after infection, nuclei were stained with Hoechst 33342 (1:10,000 in HBSS) for 30 min at 37 °C, and fluorescence images were acquired using a fluorescence microscope (MI52-N).

### Cryo-EM sample preparation and data collection

Cryo-EM samples were prepared by mixing the respective protein components at defined molar ratios. For the *E.* *amurensis* APN–RU15-1 RBD complex, proteins were combined at a 1:3 molar ratio. For the RU15-1 RBD–BD23-0177 Fab–BD23-0152 antigen-binding fragment (Fab) and MO1 RBD – BD23-0027 Fab–BD23-0087 Fab complexes, the components were mixed at 1:1.2:1.2 molar ratios, respectively. The RU15-1 spike sample was adjusted to a final concentration of 1 mg ml^−1^. All mixtures were incubated at 4 °C overnight before grid preparation, following the same procedure used for previous cryo-EM sample preparations^64^. Each complex was subjected to size-exclusion chromatography to ensure sample homogeneity. The subsequent mixture of RBD and *E.* *amurensis* APN required the addition of amastatin to a final concentration of 1 mM (ref. ^65^). The samples were applied to glow-discharged holey carbon-coated gold grids (C-flat, 300 mesh, 1.2/1.3, Protochips), blotted for 7 s without blotting force under 100% relative humidity and plunge-frozen in liquid ethane using a Vitrobot (FEI).

Cryo-EM data were collected on an FEI Titan Krios transmission electron microscope operated at 300 kV. Videos were recorded using a K3 Summit direct electron detector in counting mode, with each video consisting of 32 frames (0.2 s per frame) and a total electron dose of 60 e^−^ per Å^2^. Automated data acquisition was performed using SerialEM. For the *E.* *amurensis* APN–RU15-1 RBD, the RU15-1 RBD–BD23-0177 Fab–BD23-0152 Fab and MO1 RBD–BD23-0027 Fab–BD23-0087 Fab complexes, data were acquired at a nominal magnification of ×165,000, corresponding to a calibrated pixel size of 0.82 Å. For RU15-1 spike, data were acquired at a nominal magnification of ×29,000, corresponding to a calibrated pixel size of 1.07 Å. The defocus range was set between −1.2 and −2.0 μm.

### Cryo-EM data processing, model fitting and refinement

A total of 2,125, 3,559, 4,260 and 4,002 micrographs were collected for the RU15-1 spike, *E.* *amurensis* APN–RU15-1 RBD, RU15-1 RBD–BD23-0177–BD23-0152 and MO1 RBD–BD23-0027–BD23-0087 complexes, respectively. Beam-induced motion correction was performed using MotionCor2, and the defocus values of each micrograph were estimated with Gctf^66^. A total of 1,073,532, 1,178,055, 1,516,562 and 1,134,767 particles were extracted from the corresponding datasets. Based on the two-dimensional class averages, 215,898, 170,003, 179,494 and 129,849 particles were selected for final reconstruction in cryoSPARC^67^.

During ab initio reconstruction, no symmetry was imposed, which resulted in multiple initial models representing potential conformational states of the complexes. For homogeneous refinement, symmetries of C3, C2, C1 and C1 were applied to the RU15-1 spike, *E.* *amurensis* APN–RU15-1 RBD, RU15-1 RBD–BD23-0177–BD23-0152 and MO1 RBD–BD23-0027–BD23-0087 datasets, respectively. The best models were subsequently subjected to non-uniform refinement in cryoSPARC to generate the final cryo-EM density maps. For the *E.* *amurensis* APN–RU15-1 RBD complex, to further improve the local resolution at the interaction interface between *E.* *amurensis* APN and RU15-1 RBD, symmetry expansion followed by local refinement (C1) was performed, yielding a high-resolution structure of the *E.* *amurensis* APN monomer in complex with RU15-1 RBD.

The final resolution of each structure was estimated using the gold-standard Fourier shell correlation criterion (threshold = 0.143) and local resolution was evaluated using ResMap^68–70^. A detailed summary of the image processing workflow is provided in Supplementary Information and Extended Data Figs. 5d and 9a,b, and the statistics are listed in Supplementary Table 7.

The atomic models^71^ of all complexes were generated by fitting structures (predicted by AlphaFold3) into the cryo-EM densities described above by Chimera^72^, followed by manual adjustment and correction according to the protein sequences and densities in Coot^73^, as well as real space refinement using Phenix^74^. Local agreement between the atomic model and the cryo-EM map were assessed using the DAQ-score method (https://daqdb.kiharalab.org/)^75^. Details of the refinement statistics of the complexes are summarized in Supplementary Table 7.

### Bioinformatic and structural analysis

A dataset of 27 unique EriCoV S glycoproteins was gathered via BLAST searches of the NCBI database using the HKU31-F6 S protein as a reference sequence. A total of 93 orthologous APN protein sequences from mammals and birds were either directly annotated or assembled from whole-genome sequences or RNA-seq raw data obtained from the NCBI or GSA databases. The whole genome sequences, RBD (corresponding to NeoCoV residues 380–585), and S protein amino acid sequences of EriCoV, and APN amino acid sequences used for phylogenetic analysis were aligned using MAFFT (v7.526) (ref. ^76^), and the RBM sequence alignments in Extended Data Fig. 1e were aligned via MAFFT with manual adjustments to optimize indel positioning. Phylogenetic trees were constructed using IQ-TREE (version 3.0.1) (ref. ^77^) under the maximum-likelihood model with 1,000 bootstrap replicates. Evolutionary model selection was performed using ModelFinder Plus in IQ-TREE with Bayesian information criterion optimization. The best-fit models implemented were GTR+R5+F for the whole genomes, WAG+G4 for the RBD and spike amino acid sequences, and Q.plant+R5+F for the APN amino acid sequences. The resulting trees were visualized and rendered using iTOL (https://itol.embl.de/). Pairwise sequence identities were calculated in Geneious Prime (version 2023.0.4) (https://www.geneious.com/) following MAFFT-DASH alignment. Experimentally resolved structures from Protein Data Bank (PDB) used for structural analysis in this study included MERS-CoV RBD (4KQZ), HKU4-1 RBD (4QZV), HKU5-19s RBD (9D32), NeoCoV RBD (7WPO>), EriCoV-GER216 RBD (9JMG), MOW15-22 RBD (9C6O), HCoV-229E RBD-hAPN complex (6U7E), PDCoV RBD-pAPN complex (7VPQ) and PRCV RBD-pAPN complex (4F5C). Structural visualization and analysis were performed using ChimeraX (v1.7.1).

### mCSM-PPI2 analysis of human APN mutations affecting binding affinity

To evaluate the impact of human APN mutations on its interaction with the RU15-1 RBD, we computationally predicted the changes in binding free energy (ΔΔ*G*) induced by multiple amino acid substitutions. Specifically, the PPI2 prediction was performed using the mCSM-PPI2 web server (https://biosig.lab.uq.edu.au/mcsm_ppi2/), which predicts the effects of mutations on protein–protein interactions based on graph-based structural signatures^78^. The structure of the hAPN–RU15-1 complex was used as the template for all calculations after removal of solvent molecules and glycosylation site. In brief, the experimentally determined structure of the WT protein complex was used as input (in PDB format), and mutations were defined based on residue positions corresponding to the structural model. The predicted effects of mutations on binding affinity were evaluated in terms of changes in binding free energy (ΔΔ*G*), as implemented in the mCSM-PPI2 framework.

For each designed mutation, PPI2 was applied to estimate the binding free energy change (ΔΔ*G*), calculated as ΔΔ*G* = Δ*G*_wt_ − Δ*G*_mut_, where Δ*G*_wt_ and Δ*G*_mut_ represent the predicted binding free energy values of the WT and mutant complexes, respectively. Positive ΔΔ*G* values indicate a predicted enhancement of binding affinity, and negative values correspond to reduced affinity relative to the WT.

### Quantification and statistical analysis

Most experiments related to pseudovirus infection were conducted 2–3 times with *n* = 3 independently infected wells, as indicated in the figure legends. Representative results are shown. Data are presented by mean, mean ± s.d. or mean ± standard error of the mean (s.e.m.), as indicated in the corresponding figure legends. Unpaired two-tailed *t*-tests were conducted for most statistical analyses for two independent groups, and one-way analysis of variance followed by Dunnett’s test was used in Fig. 4b using GraphPad Prism 8.0.2. *P* < 0.05 was considered significant. *P < 0.05, **P < 0.01, ***P < 0.001 and ****P < 0.0001, NS: not significant. No data were excluded for data analysis. Human serum samples were collected from randomly selected donors. Randomization was not applicable to other experiments, as experimental groups and conditions were predefined according to the study design. Data collection and analysis were not performed blind to the conditions of the experiments. Data distribution was assumed to be normal, but this was not formally tested. No statistical methods were used to predetermine sample sizes, but our sample sizes are similar to those reported in previous publications^26,30,79^.

### Reporting summary

Further information on research design is available in the Nature Portfolio Reporting Summary linked to this article.

## Supplementary information

Supplementary Information Supplementary Figs. 1–6 and source data for Supplementary Figs. 1a,b, 3 and 4.

Reporting Summary

Peer Review File

Supplementary Table 1 EriCoV S sequences.

Supplementary Table 2 Hedgehog receptor sequences.

Supplementary Table 3 APN orthologues sequences in this study.

Supplementary Table 4 The antibody sequences of RU15-1 RBD, MO1 RBD, and *E.amu* APN.

Supplementary Table 5 List of interacting residues between Fabs and RU15-1 or MO1 RBDs.

Supplementary Table 6 Demographic data for the plasma donors.

Supplementary Table 7 Cryo-EM data collection and atomic models refinement statistics.

Supplementary Data 1 Source data for Supplementary Fig. 5.

Supplementary Data 2 Source data for Supplementary Fig. 6.

## Source data

Source Data Fig. 1 Statistical source data.

Source Data Fig. 1 Unprocessed western blots.

Source Data Fig. 2 Statistical source data.

Source Data Fig. 4 Statistical source data.

Source Data Fig. 4 Unprocessed western blots.

Source Data Fig. 5 Statistical source data.

Source Data Extended Data Fig. 3 Statistical source data.

Source Data Extended Data Fig. 4 Statistical source data.

Source Data Extended Data Fig. 6 Statistical source data.

Source Data Extended Data Fig. 7 Statistical source data.

Source Data Extended Data Fig. 7 Unprocessed western blots.

Source Data Extended Data Fig. 8 Statistical source data.

Source Data Extended Data Fig. 10 Statistical source data.

## Data Availability

The cryo-EM maps and models have been deposited to the Electron Microscopy Data Bank and PDB with accession numbers EMD-67300/PDB 9XVR (global refinement of the RU15-1 spike trimer), EMD-81298/PDB 27OA (focused reconstruction of the *E.* *amurensis* APN in complex with RU15-1 RBD), EMD-67330/PDB 9XWA (global refinement of the *E.* *amurensis* APN in complex with RU15-1 RBD), EMD-67338/PDB 9XWN (global refinement of the RU15-1 RBD–BD23-0177 Fab–BD23-0152 Fab complex) and EMD-67337/PDB 9XWM (global refinement of the MO1 RBD–BD23-0027 Fab–BD23-0087 Fab complex). The 27 EriCoV S sequences, 6 receptor sequences from hedgehog (*E.* *amurensis* ACE2, *E.* *amurensis* DPP4, *E.* *amurensis* APN, *E.* *amurensis* TMPRSS2, *H. suillus* APN and *A.* *albiventris* APN) and 93 APN-coding sequences are provided in the Methods and Supplementary Tables 1–7. All other data supporting the findings of this study are available with the article and Supplementary Information. Source data are provided with this paper.

## Code availability

This study did not generate any custom computer code.
