# Supplementary material for: Convergent use of aminopeptidase N receptor by hedgehog merbecoviruses
Source: Nat Microbiol. 2026 Sep 16;11(10):2821–38. doi: 10.1038/s41564-026-02478-1 (PMC13716839; doi:10.1038/s41564-026-02478-1)
Supplement: Supplementary file 2 [file 41564_2026_2478_MOESM17_ESM.pdf]

Source data from Fig. 4a

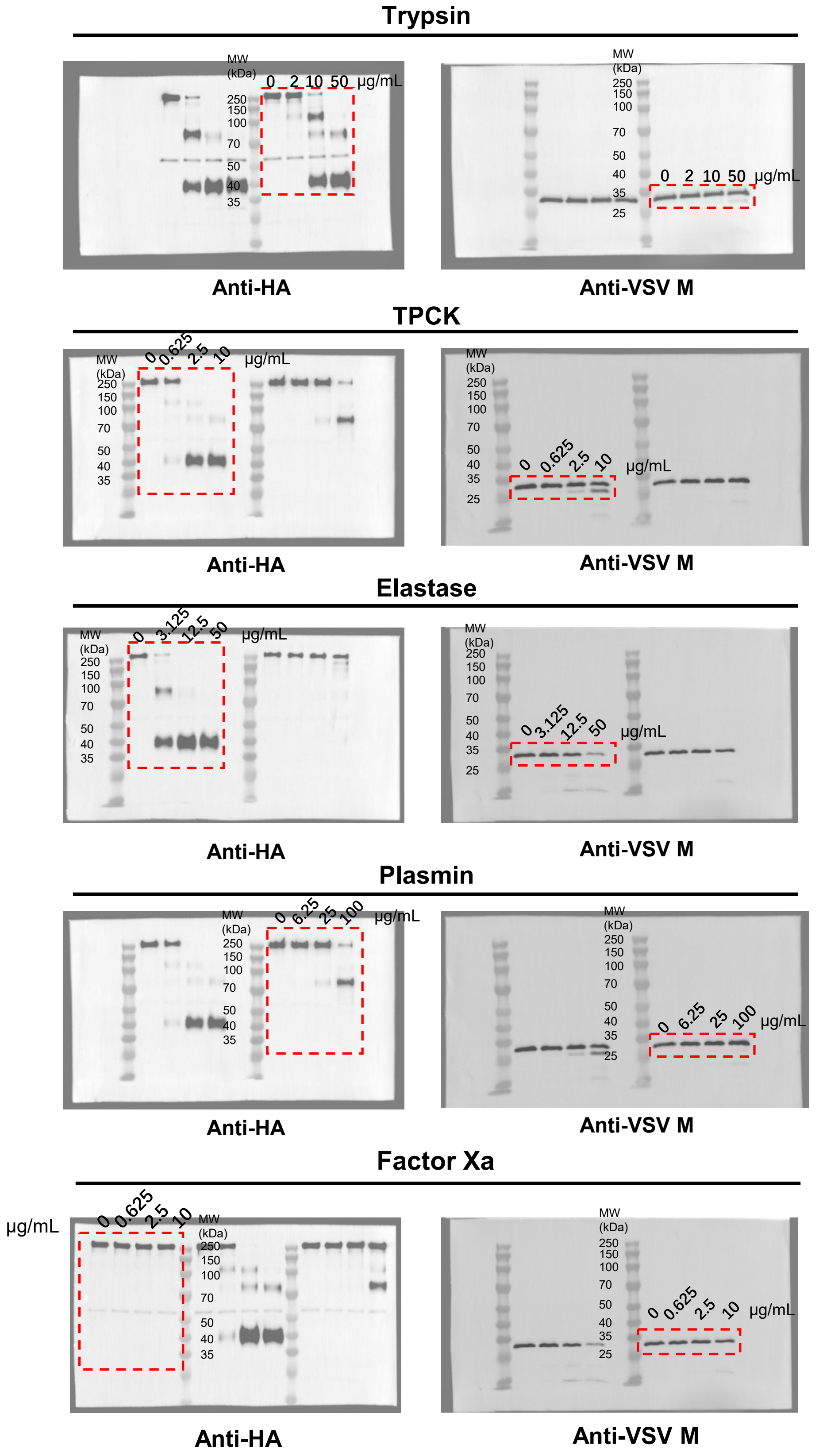

Source data for Fig. 4a | Uncropped immunoblots from Fig. 4a. Uncropped and unprocessed scans of the protein blots used in Fig. 4a. Molecular weight markers, detecting antibodies, and the corresponding Figures were indicated.
