# Supplementary material for: Convergent use of aminopeptidase N receptor by hedgehog merbecoviruses
Source: Nat Microbiol. 2026 Sep 16;11(10):2821–38. doi: 10.1038/s41564-026-02478-1 (PMC13716839; doi:10.1038/s41564-026-02478-1)
Supplement: Supplementary file 3 [file 41564_2026_2478_MOESM1_ESM.pdf]

nature microbiology

Supplementary information

https://doi.org/10.1038/s41564-026-02478-1

Convergent use of aminopeptidase N receptor by hedgehog merbecoviruses

In the format provided by the authors and unedited

Supplementary information for

Convergent use of Aminopeptidase N receptor by hedgehog merbecoviruses

Peng Liu[1,#], Qianhui Zhu[2,3,#], Jingyi Liu[4,5,7,#], Zhixing Ma[1,#], Guocui Mou[8,#], Yuanling Yu[5], Chen Liu[1], Chengbao Ma[1], Junyu Si[1], Xiao Yang[1], Meiling Huang[1], Yanchu Liu[1], Yehui Sun[1], Xiao Yu[1], Yucheng Sun[1], Tianci Liu[2,3], Lingling Yu[5], Qianran Wang[5], Honghao Ma[5], Qi Lv[8], Jing Li[1], Linlin Bao[8,*], Yunlong Cao[4,5,6,*], Xiangxi Wang[2,3,*], Huan Yan[1,*]

Supplementary Fig. 1 | Western blot analysis of the expression of EriCoV spike and package of VSV pseudovirions.
Supplementary Fig. 2 | Binding of EriCoV RBD-hFc to HEK293T cells transiently expressing APN orthologues.
Supplementary Fig. 3 | Western blot analysis of the expression of the APN orthologues and mutants.
Supplementary Fig. 4 | Western blot analysis of the expression of the APN mutants.
Supplementary Fig. 5 | *E.amu* TMPRSS2 can promote the infection of EriCoV pseudoviruses in HEK293T transiently expressing *E.amu* APN.
Supplementary Fig. 6 | Gating strategies for representative flow cytometry analyses.

Source data for Supplementary Fig. 1a
Source data for Supplementary Fig. 1b
Source data for Supplementary Fig. 3
Source data for Supplementary Fig. 4

Excel file for Supplementary Table 1
Excel file for Supplementary Table 2
Excel file for Supplementary Table 3
Excel file for Supplementary Table 4
Excel file for Supplementary Table 5
Excel file for Supplementary Table 6
Excel file for Supplementary Table 7

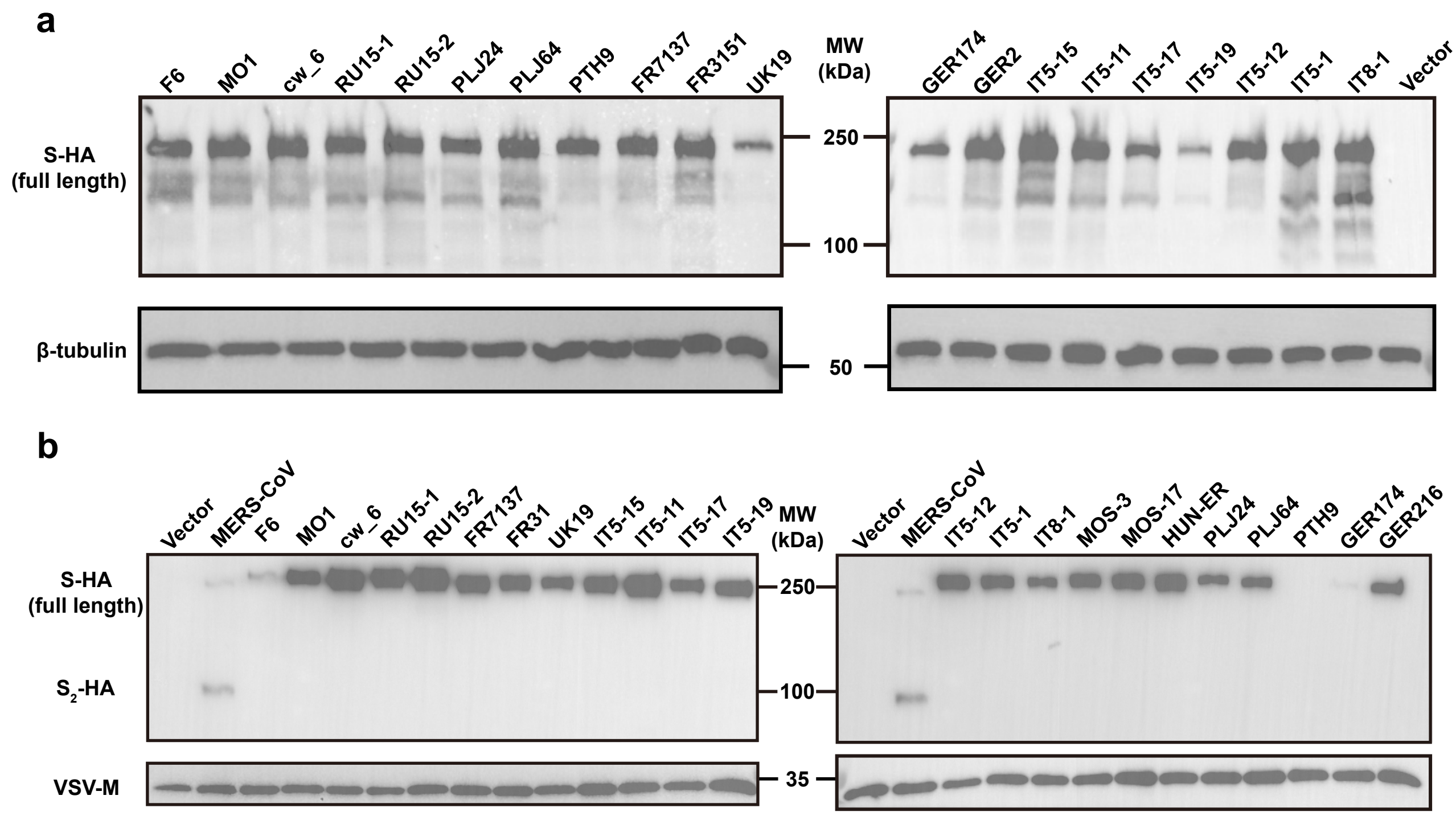

Supplementary Fig. 1 | Western blot analysis of the expression of EriCoV spike and package of VSV pseudovirions. (**a**) Western blot analysis of indicated EriCoV S glycoprotein expression in HEK293T cells for Extended Data 2d. β-tubulin served as a loading control. (**b**) Detection of EriCoV S glycoprotein in VSV pseudovirions for Fig. 1j via Western blot using an anti-HA antibody targeting the C-terminally fused HA tag. VSV-M served as a virion normalization control.

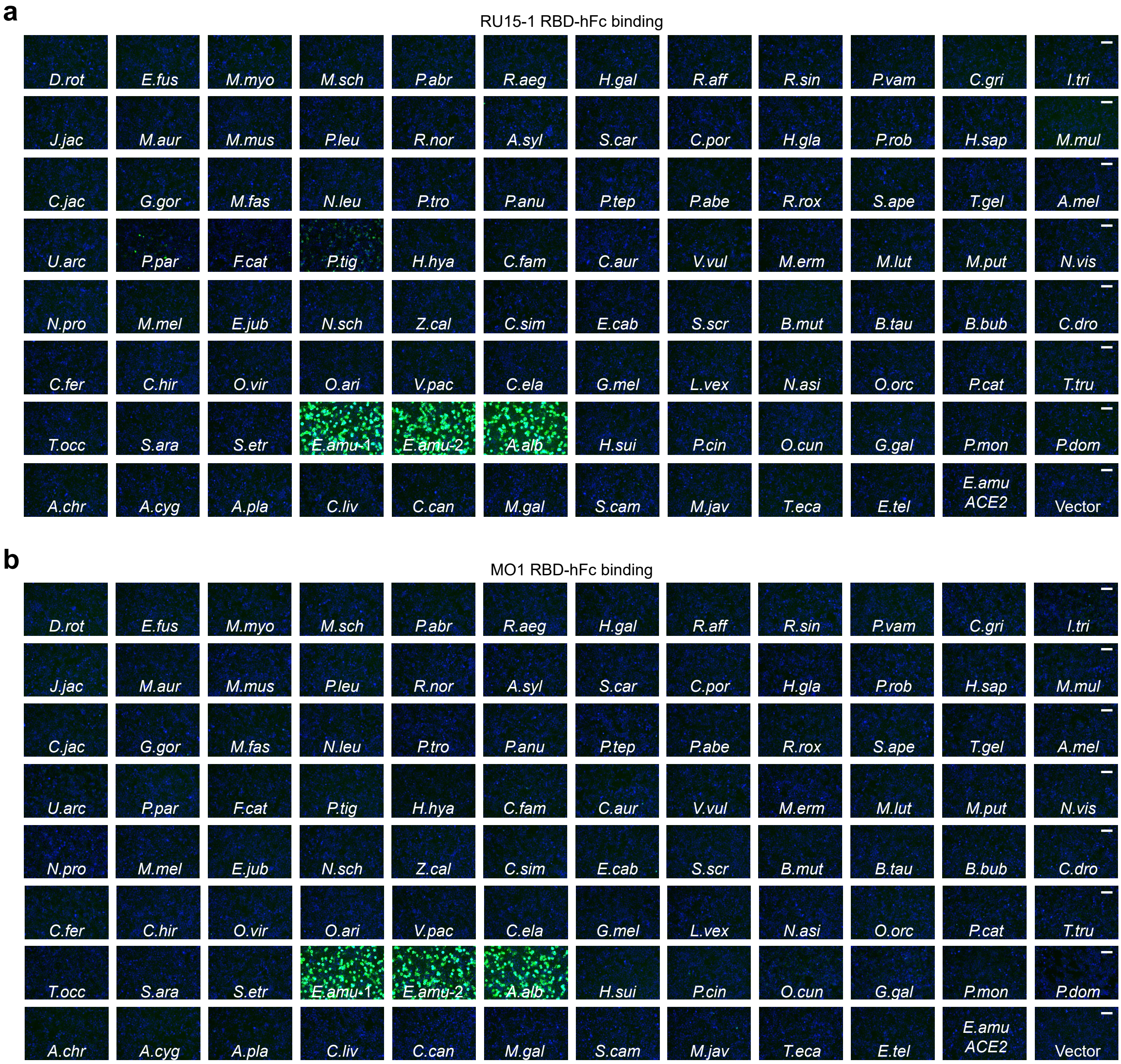

Supplementary Fig. 2 | Binding of EriCoV RBD-hFc to HEK293T cells transiently expressing APN orthologues. (**a, b**) Binding of RU15-1 (a) or MO1 (b) RBD-hFc to HEK293T cells transiently expressing the indicated APN orthologues, assessed by live-cell immunofluorescence using an anti-hFc antibody (green). The immunofluorescence assays shown were representative of two independent experiments. Scale bars: 100 μm.

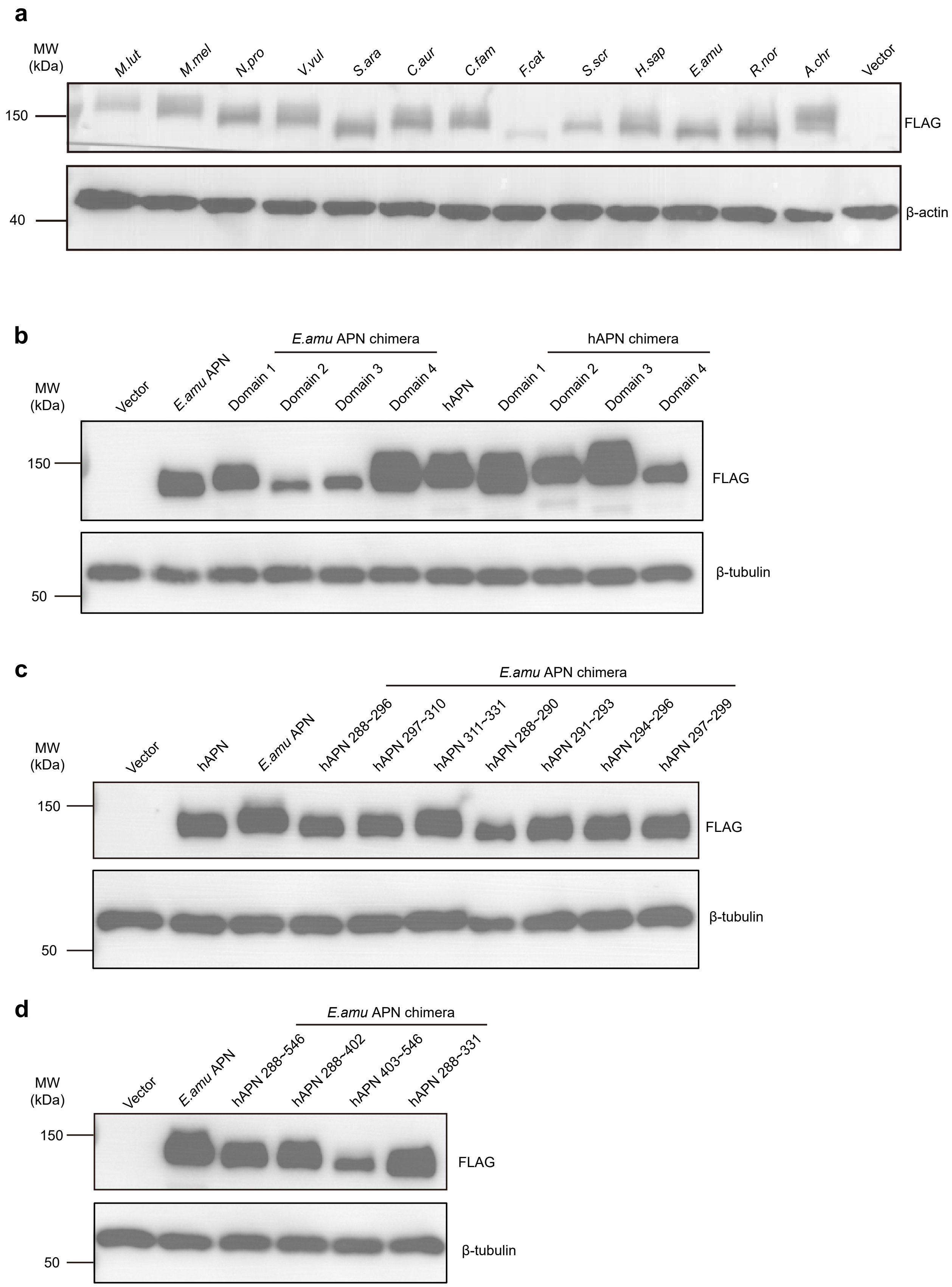

Supplementary Fig. 3 | Western blot analysis of the expression of the APN orthologues and mutants. (**a**) Western blot analysis of indicated APN orthologues in Extended Data Fig. 3c expression in HEK293T cells. β-actin served as a loading control. (**b-d**) Western blot validation of the expression of chimeric APN constructs of Fig. 2e, 2g, and 2h by detecting the C-terminally fused FLAG tags. β-tubulin served as a loading control.

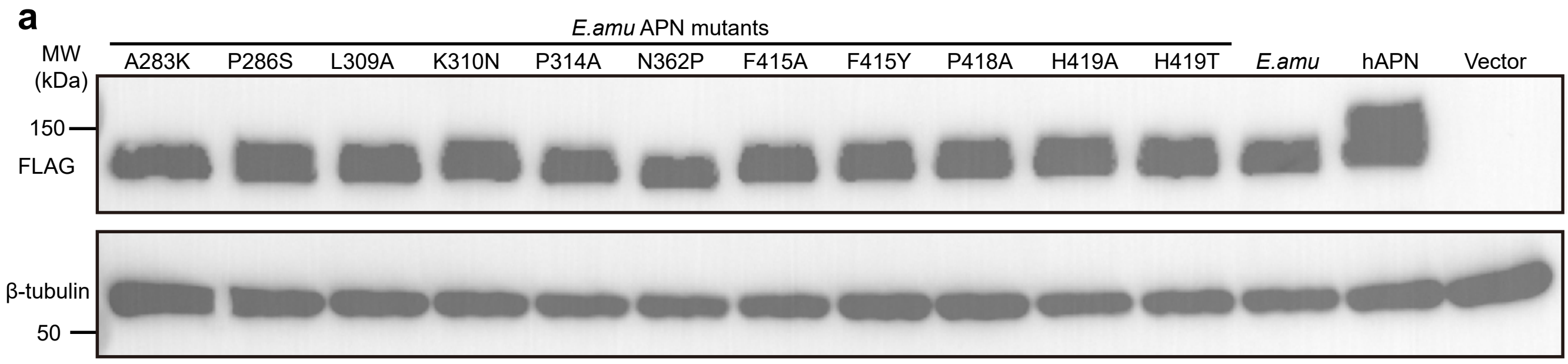

Supplementary Fig. 4 | Western blot analysis of the expression of the APN mutants. (**a**) Western blot validation of the expression of *E.amu* APN mutants constructs of Fig. 3h by detecting the C-terminally fused FLAG tags. β-tubulin served as a loading control.

a

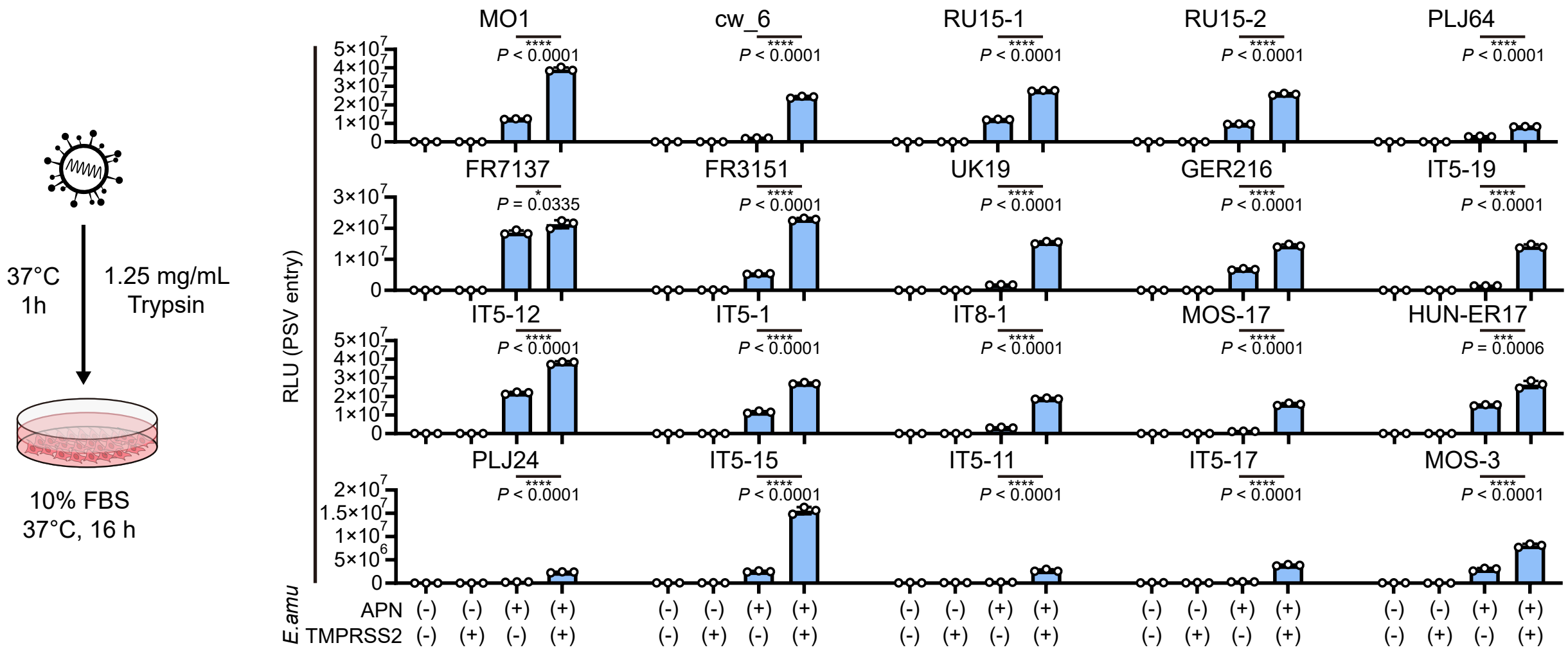

Supplementary Fig. 5 | *E.amu* TMPRSS2 can promote the infection of EriCoV pseudoviruses in HEK293T transiently expressing *E.amu* APN. (**a**) Pseudovirus entry efficiency of 20 EriCoV strains into HEK293T cells transiently expressing the *E.amu* APN, *E.amu* TMPRSS2, or both. Pseudoviruses were pretreated with 1.25 mg/mL trypsin, and infection was performed in the presence of 10% FBS. Statistical significance was determined using unpaired two-tailed *t*-tests (n = 3 independently infected wells). *: P<0.05, **: P<0.01, ***: P<0.001, and ****: P<0.0001, NS: not significant.

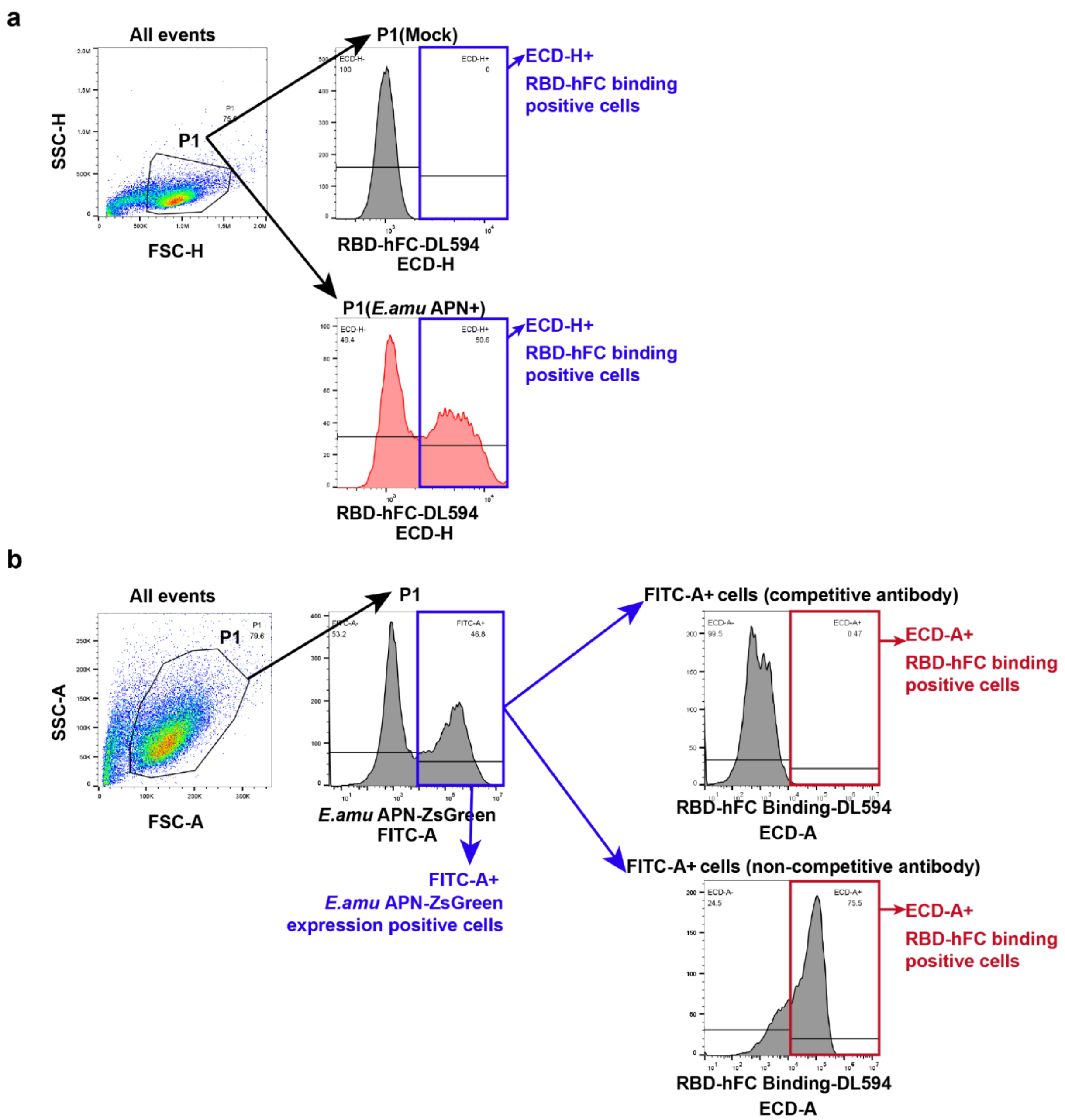

Supplementary Fig. 6 | Gating strategies for representative flow cytometry analyses. (**a**) Gating strategy for analysis of EriCoV RBD-hFc binding to HEK293T cells expressing APN orthologues in Fig. 1f. Gates were applied sequentially to exclude debris and dead cells based on FSC/SSC profiles and to identify RBD-binding cells (ECD-A+), with threshold set based on the histogram of mock controls (HEK293T transfected with vector plasmids). (**b**) Gating strategy for analysis of EriCoV RBD-hFc binding to *E.amu* APN expressing HEK293T cells in the presence of 10 μg/mL indicated antibodies in Extended Data Fig. 8e. After exclusion of debris and dead cells by FSC/SSC gating, *E.amu* APN-zsGreen-positive cells were selected as FITC-A+, and RBD-binding cells (ECD-A+) were quantified within the FITC-A+ population. Positive gates for *E.amu* APN-zsGreen-positive cells were defined using mock-transfected HEK293T cells.

Source data for Supplementary Fig. 1a

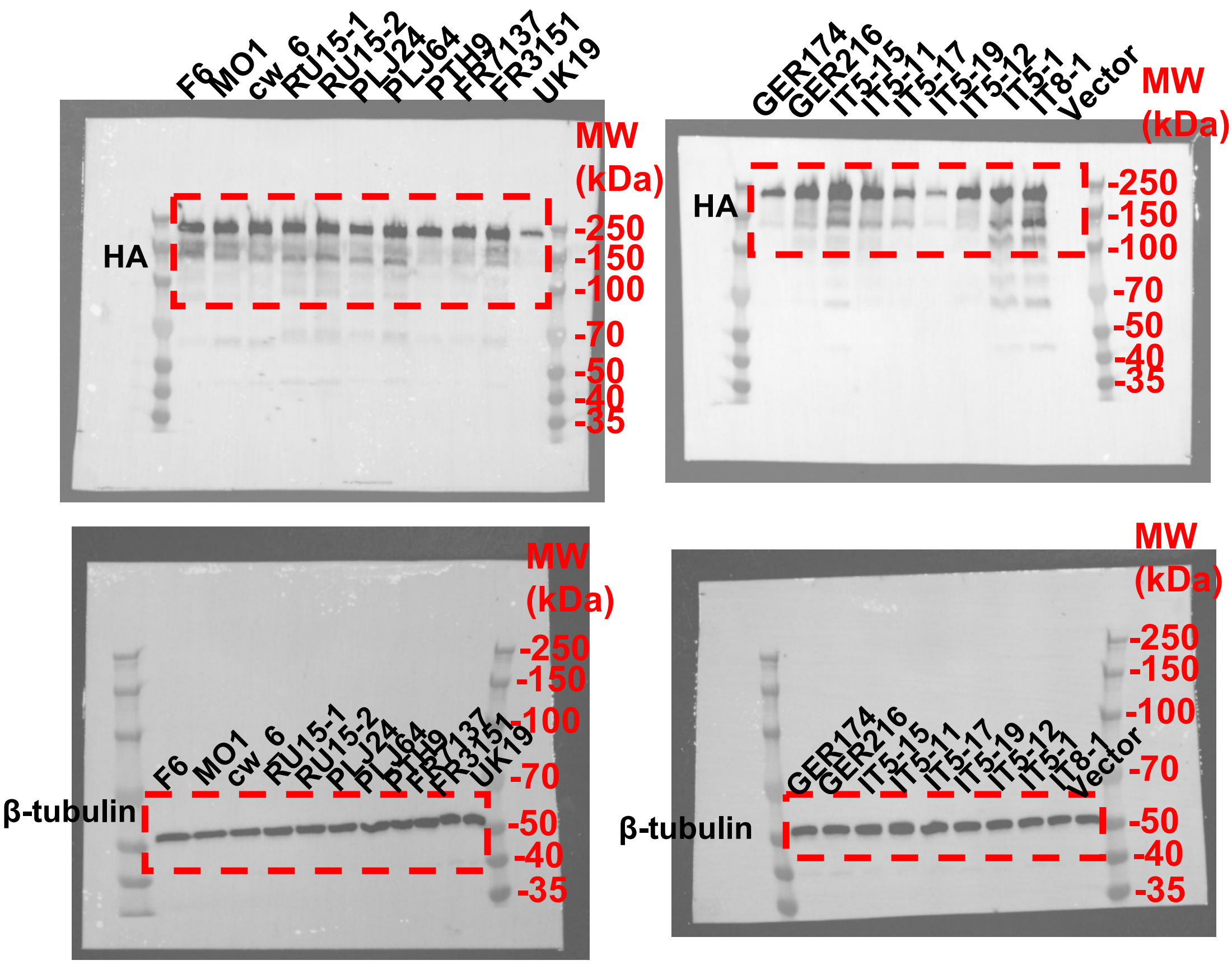

Source data for Supplementary Fig. 1a | Uncropped immunoblots from Supplementary Fig. 1a. Uncropped and unprocessed scans of the protein blots used in Supplementary Fig. 1a. Molecular weight markers, detecting antibodies, and the corresponding Figures were indicated.

Source data for Supplementary Fig. 1b

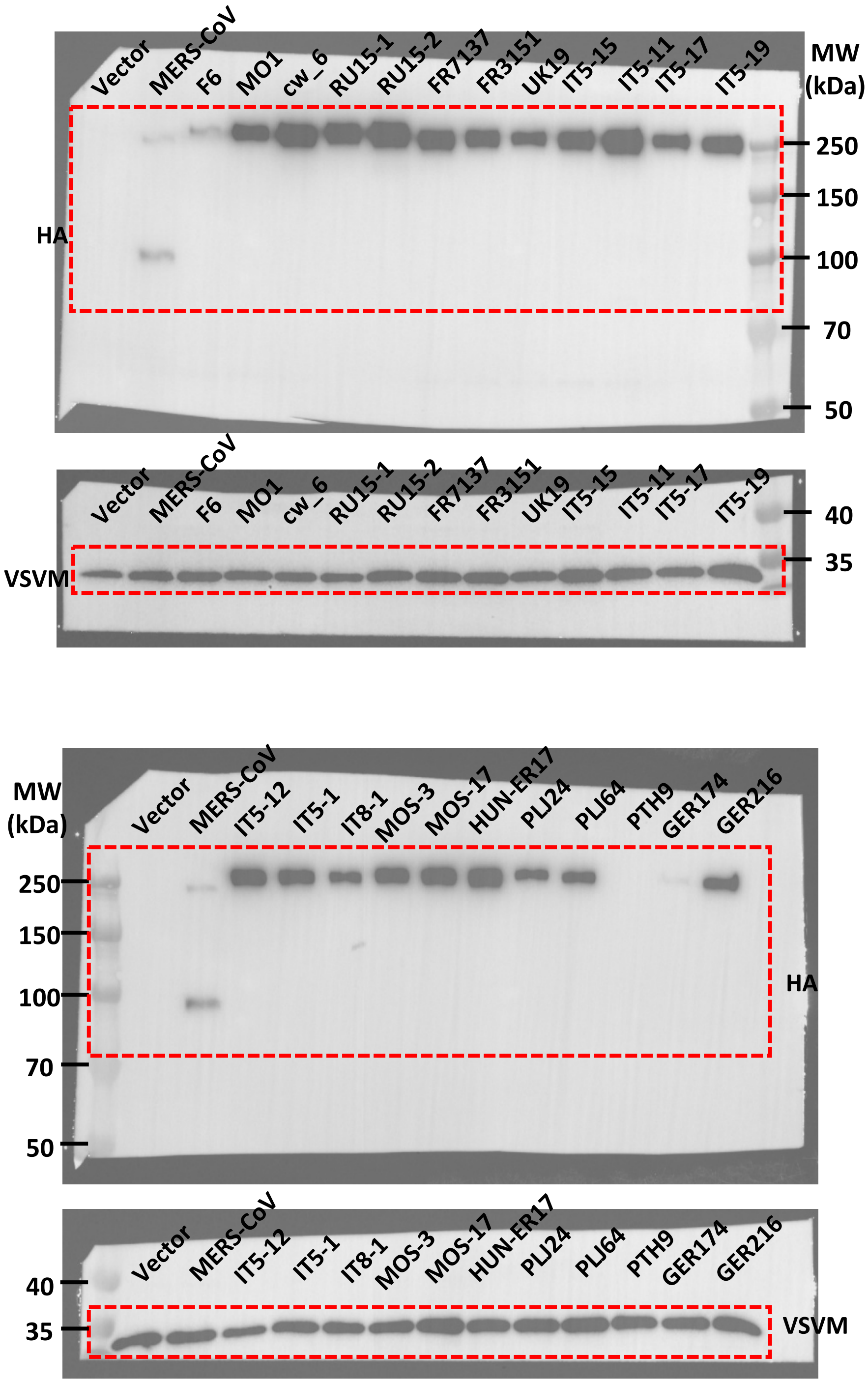

Source data for Supplementary Fig. 1b | Uncropped immunoblots from Supplementary Fig. 1b. Uncropped and unprocessed scans of the protein blots used in Supplementary Fig. 1b. Molecular weight markers, detecting antibodies, and the corresponding Figures were indicated.

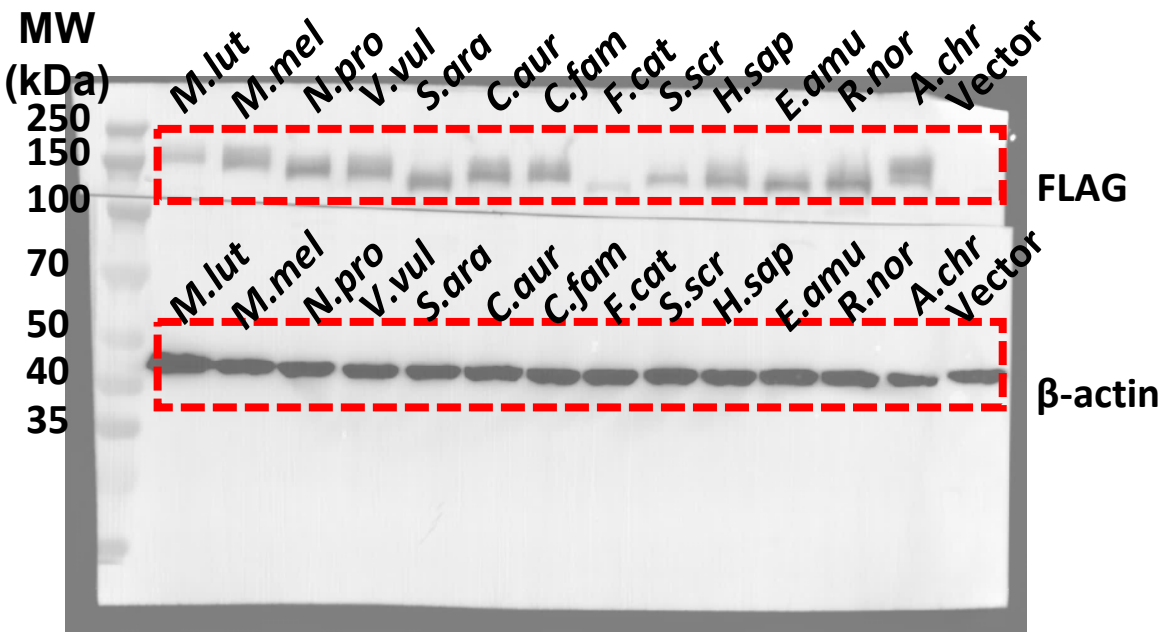

Source data for Supplementary Fig. 3a

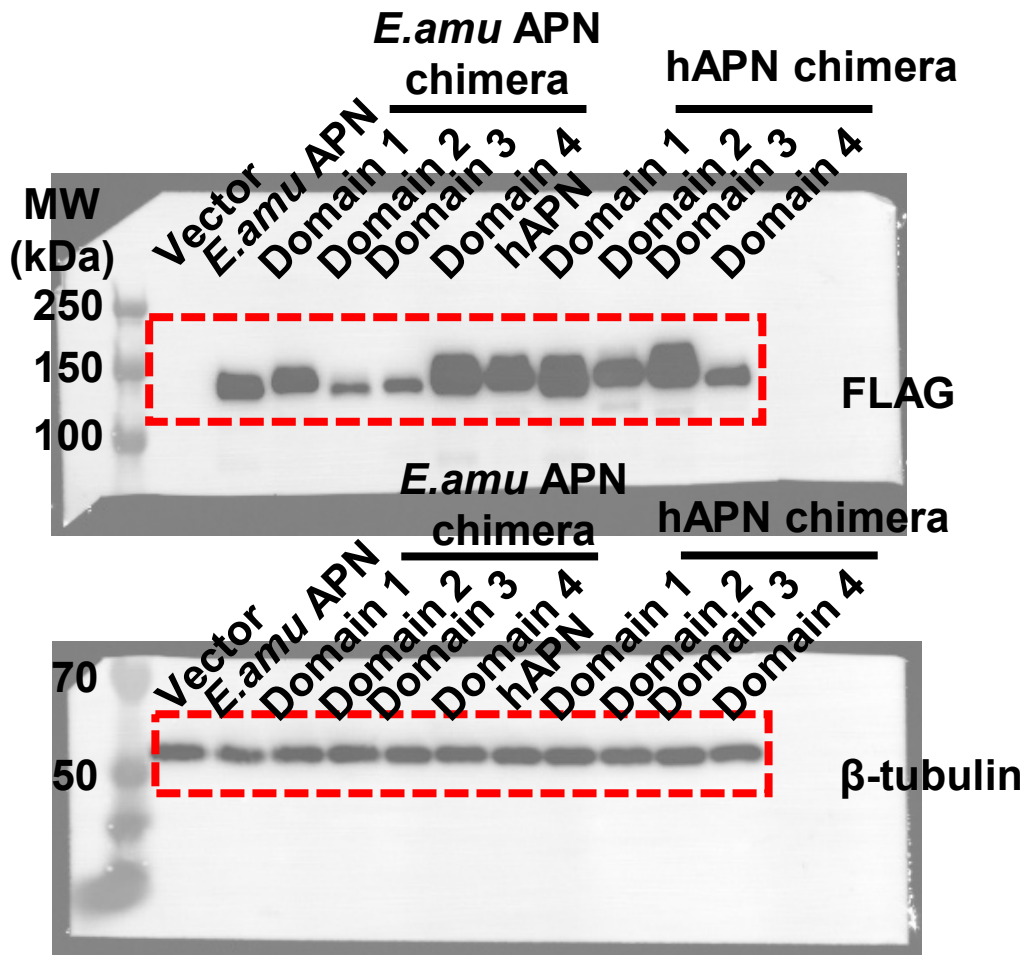

Source data for Supplementary Fig. 3b

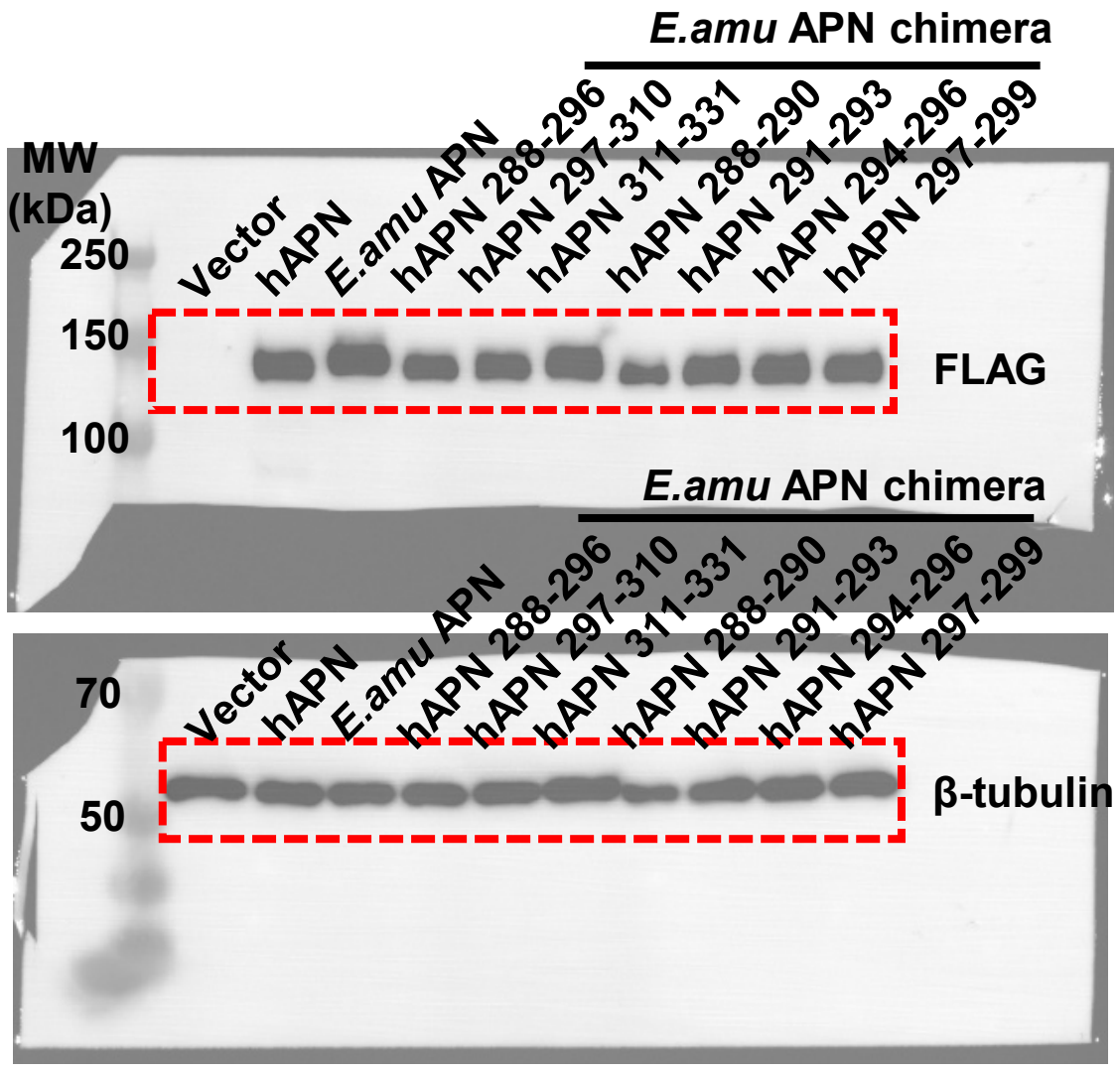

Source data for Supplementary Fig. 3c

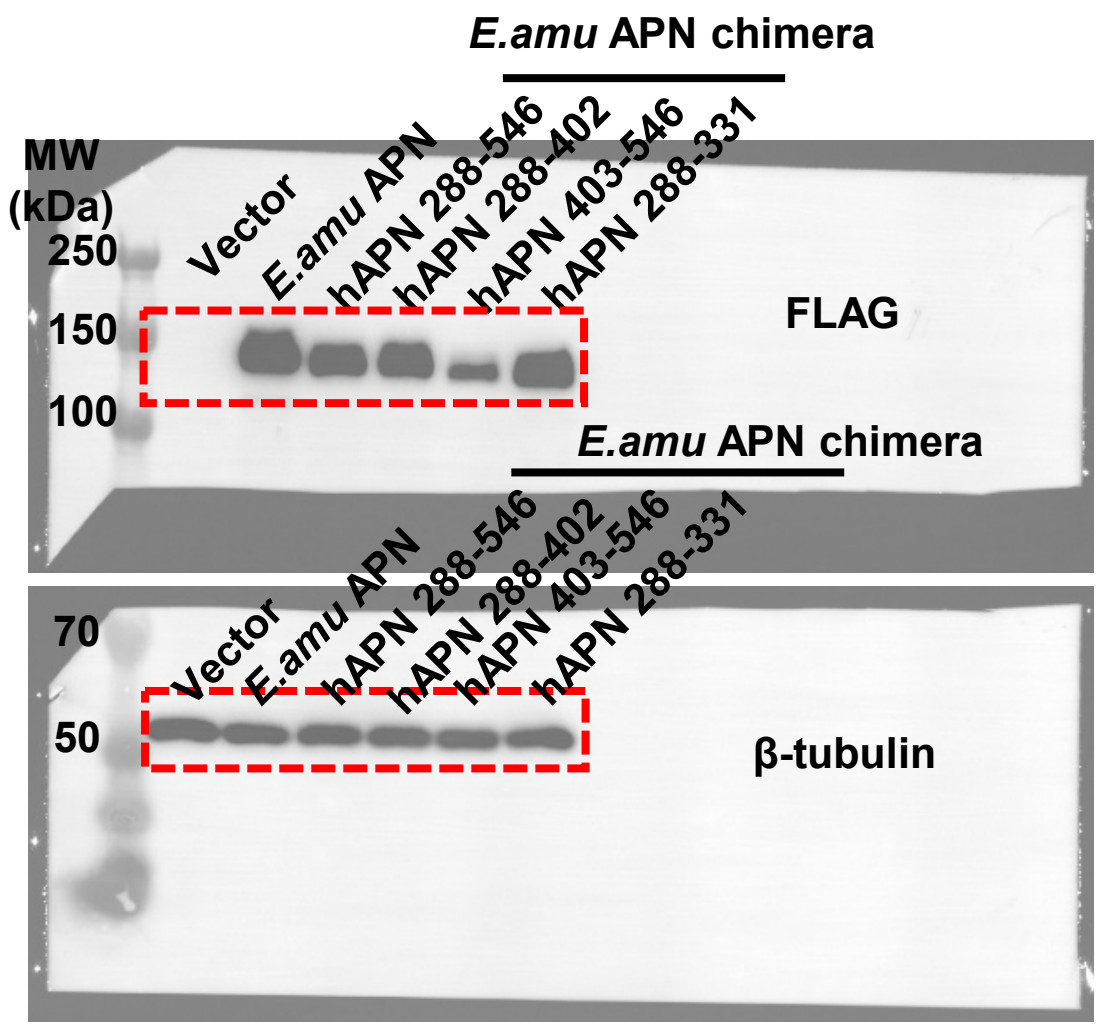

Source data for Supplementary Fig. 3d

Source data for Supplementary Fig. 3 | Uncropped immunoblots from Supplementary Fig. 3. Uncropped and unprocessed scans of the protein blots used in Supplementary Fig. 3. Molecular weight markers, detecting antibodies, and the corresponding Figures were indicated.

Source data for Supplementary Fig. 4

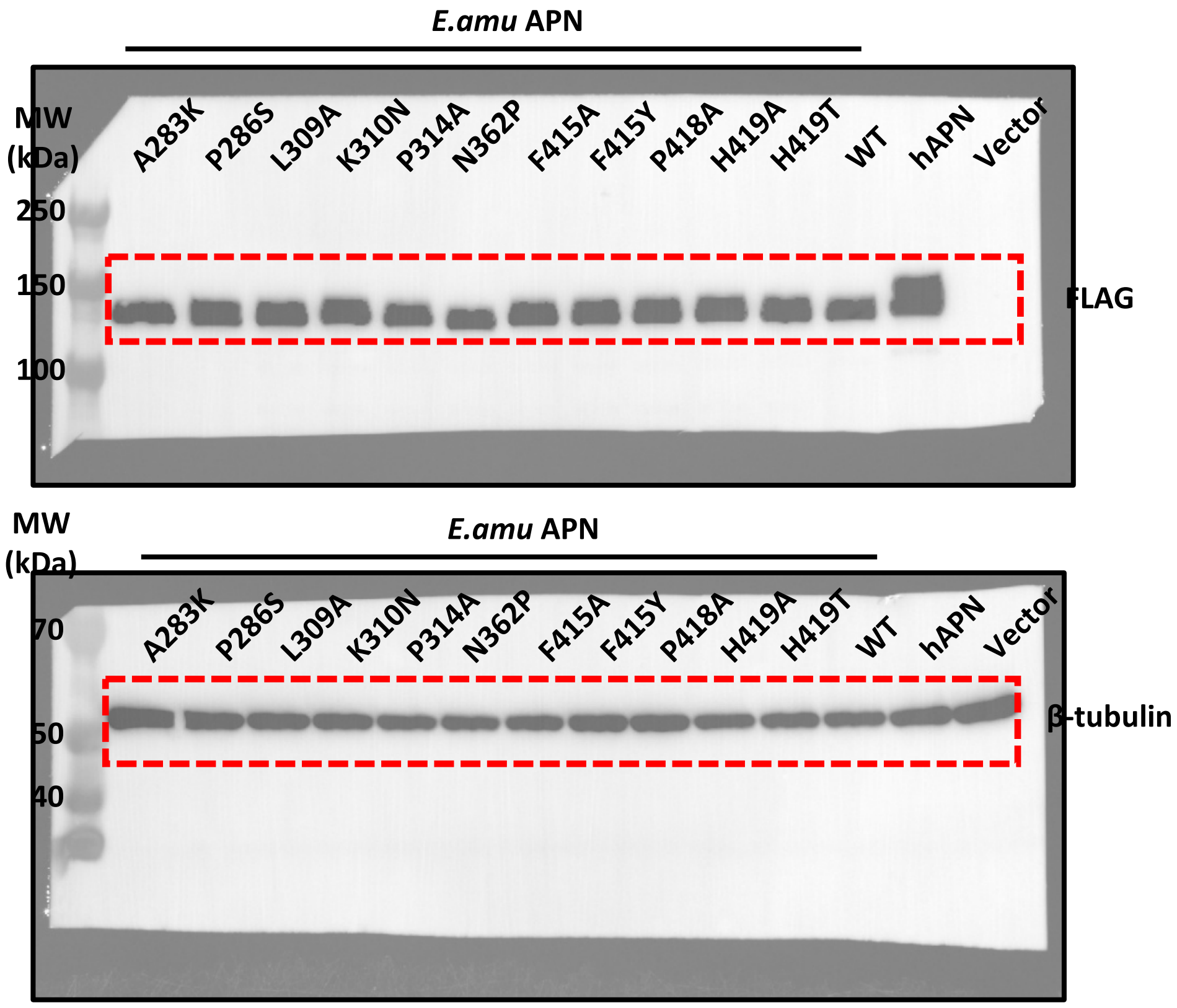

Source data for Supplementary Fig. 4 | Uncropped immunoblots from Supplementary Fig. 4. Uncropped and unprocessed scans of the protein blots used in Supplementary Fig. 4. Molecular weight markers, detecting antibodies, and the corresponding Figures were indicated.
