# Supplementary material for: Convergent use of aminopeptidase N receptor by hedgehog merbecoviruses
Source: Nat Microbiol. 2026 Sep 16;11(10):2821–38. doi: 10.1038/s41564-026-02478-1 (PMC13716839; doi:10.1038/s41564-026-02478-1)
Supplement: Supplementary file 4 [file 41564_2026_2478_MOESM23_ESM.pdf]

Source data from Extended Data Fig. 7a

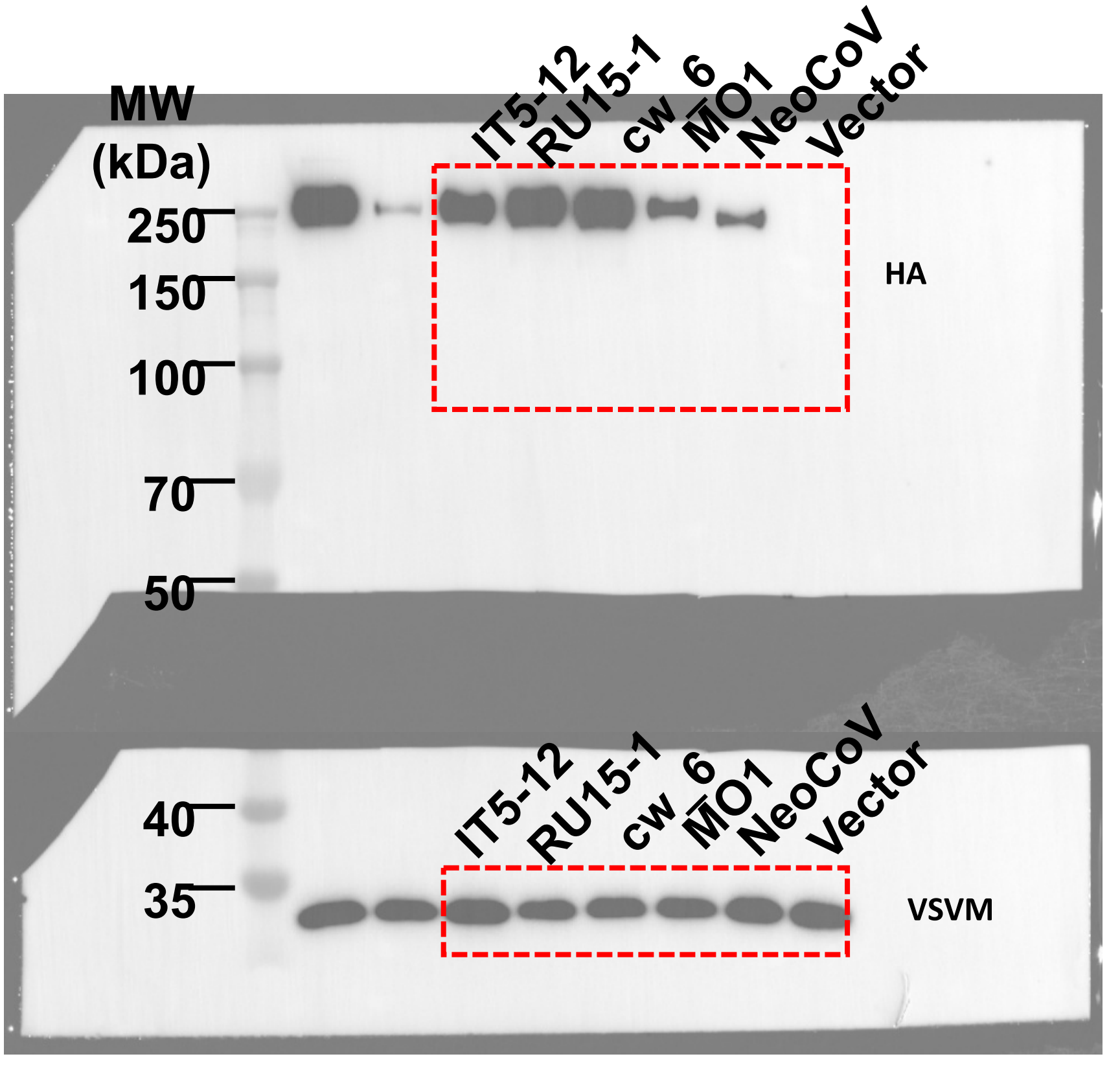

Source data for Extended Data Fig. 7a | Uncropped immunoblots from Extended Data Fig. 7a. Uncropped and unprocessed scans of the protein blots used in Extended Data Fig. 7a. Molecular weight markers, detecting antibodies, and the corresponding Figures were indicated.
