# Supplementary material for: Convergent use of aminopeptidase N receptor by hedgehog merbecoviruses
Source: Nat Microbiol. 2026 Sep 16;11(10):2821–38. doi: 10.1038/s41564-026-02478-1 (PMC13716839; doi:10.1038/s41564-026-02478-1)
Supplement: Supplementary file 5 [file 41564_2026_2478_MOESM2_ESM.pdf]

nature portfolio

Corresponding author(s): Huan Yan

Last updated by author(s): Sep 1, 2026

Reporting Summary

Nature Portfolio wishes to improve the reproducibility of the work that we publish. This form provides structure for consistency and transparency in reporting. For further information on Nature Portfolio policies, see our Editorial Policies and the Editorial Policy Checklist.

Statistics

For all statistical analyses, confirm that the following items are present in the figure legend, table legend, main text, or Methods section.

n/a	Confirmed	
☐	☒	The exact sample size (*n*) for each experimental group/condition, given as a discrete number and unit of measurement
☐	☒	A statement on whether measurements were taken from distinct samples or whether the same sample was measured repeatedly
☐	☒	The statistical test(s) used AND whether they are one- or two-sided<br>*Only common tests should be described solely by name; describe more complex techniques in the Methods section.*
☒	☐	A description of all covariates tested
☒	☐	A description of any assumptions or corrections, such as tests of normality and adjustment for multiple comparisons
☐	☒	A full description of the statistical parameters including central tendency (e.g. means) or other basic estimates (e.g. regression coefficient) AND variation (e.g. standard deviation) or associated estimates of uncertainty (e.g. confidence intervals)
☐	☒	For null hypothesis testing, the test statistic (e.g. *F*, *t*, *r*) with confidence intervals, effect sizes, degrees of freedom and *P* value noted<br>*Give P values as exact values whenever suitable.*
☒	☐	For Bayesian analysis, information on the choice of priors and Markov chain Monte Carlo settings
☒	☐	For hierarchical and complex designs, identification of the appropriate level for tests and full reporting of outcomes
☒	☐	Estimates of effect sizes (e.g. Cohen's *d*, Pearson's *r*), indicating how they were calculated

Our web collection on statistics for biologists contains articles on many of the points above.

Software and code

Policy information about availability of computer code

Data collection	The western blot data are collected by ChemiDoc MP Imaging System (Bio-Rad). Luciferase activity was measured using GloMax® 20/20 Luminometer (Promega, United States), Varioskan LUX Multi-well Luminometer (Thermo Fisher), or a microplate spectrophotometer (PerkinElmer, HH3400). Flow cytometry data were generated using the CytoFLEX Flow Cytometer (Beckman Coulter, United States). BLI assays were conducted on an Octet RED96 instrument (Molecular Devices, United States). Fluorescent images were captured with either the MI52-N fluorescence microscope (Mshot). Bright-field images of primary hedgehog cells were acquired using a Nikon ECLIPSE Ti2 microscope. For Cryo-electron microscopy analysis of E.amu APN–RU15-1 RBD complex, RU15-1 RBD+BD23-0177 Fab+BD23-0152 Fab complexes, and MO1 RBD+BD23-0027 Fab+BD23-0087 Fab complexes related structures, micrographs were collected at 300 kV using an FEI Titan Krios transmission electron micrscope (Thermo Fisher), equipped with a K2 direct detector (Gatan, Pleasanton, CA) with a Gatan Quantum GIF energy filter. For Cryo-electron microscopy analysis of RU15-1 Spike trimer structure, equipped with a K3 direct detector (Gatan, Pleasanton, CA). Absorbance was measured at 450 nm using a microplate reader (Thermo Scientific, Multiskan Fc). Antibodies were assessed using the Biacore 8K system (GE Healthcare). Single B cells were sorted using a MoFlo Astrios EQ Cell Sorter (Beckman Coulter) directly into 96-well PCR plates.
Data analysis	GraphPad Prism (version 8.0.2) was used for all statistical analyses. Image Lab (version 5.2.1) was utilized to analyze Western blot data. FlowJo (version 10) was employed for the analysis of flow cytometry data. Protein and nucleic acid sequence alignment was performed using the Mafft (version v7.526) and Geneious Prim (2023.0.4). Phylogenetic analysis was performed using the IQ-Tree (version 3.0.1). Protein structures were presented and analyzed using UCSF ChimeraX (version 1.7.1). Octet Data Analysis software (version 12.2.0.20) was used for the analysis of BLI data. Sequence Read Archive (SRA) database and processed using Cutadapt (version 2.4) to remove adapters. De novo assembly was performed with MEGAHIT (version 1.2.9). Final contigs were analyzed using Geneious Prim (version 2023.0.4) and were subjected to Geneious workflows for mapping reads to the indicated reference sequences. Particles-selected, and final cryo-EM density generation were done using cryoSPARC (version 4.6.2). The final resolution of each structure was estimated using the gold-standard Fourier

shell correlation (FSC), and local resolution estimation was using cryoSPARC (version 4.6.2). The atomic model of all complexes were generated by fitting structures (predicted by AlphaFold3) into the cryo-EM densities described above by Chimera (version 1.16), followed by manual adjustment and correction according to the protein sequences and densities in Coot (version 0.9.8.93), as well as real space refinement using Phenix (version 1.20). Figures were generated using UCSF ChimeraX (version 1.5.0.0). Structure-based binding affinity predictions were performed using PPI2 (http://biosig.unimelb.edu.au/mcsm_ppi2/). Local agreement between the atomic model and the cryo-EM map were assessed using the DAQ-score method (https://daqdb.kiharalab.org/).

For manuscripts utilizing custom algorithms or software that are central to the research but not yet described in published literature, software must be made available to editors and reviewers. We strongly encourage code deposition in a community repository (e.g. GitHub). See the Nature Portfolio guidelines for submitting code & software for further information.

Data

Policy information about availability of data

All manuscripts must include a data availability statement. This statement should provide the following information, where applicable:

- Accession codes, unique identifiers, or web links for publicly available datasets
- A description of any restrictions on data availability
- For clinical datasets or third party data, please ensure that the statement adheres to our policy

The cryo-EM maps and models have been deposited to the Electron Microscopy Data Bank and Protein Data Bank (PDB) with accession numbers EMD-67300/PDB ID 9XVR (global refinement of the RU15-1 spike trimer), EMD-81298/PDB ID 27OA (focused reconstruction of the E.amu APN in complex with RU15-1 RBD), EMD-67330/PDB ID 9XWA (global refinement of the E.amu APN in complex with RU15-1 RBD), EMD-67338/PDB ID 9XWN (global refinement of the RU15-1 RBD +BD23-0177 Fab + BD23-0152 Fab complex), and EMD-67337/PDB ID 9XWM (global refinement of the MO1 RBD+BD23-0027 Fab+BD23-0087 Fab complex).

The 27 EriCoV S sequences, 6 receptor sequences from hedgehog (E.amu ACE2, E.amu DPP4, E.amu APN, E.amu TMPRSS2, H.sui APN, and A.alb APN), and 93 APN-coding sequences are provided in the Methods and Supplementary Tables. All other data supporting the findings of this study are available with the Article and the Supplementary Information. Source data are provided with this paper.

Research involving human participants, their data, or biological material

Policy information about studies with human participants or human data. See also policy information about sex, gender (identity/presentation), and sexual orientation and race, ethnicity and racism.

Reporting on sex and gender	The sex and gender of participants are not relevant to the objective of this study, which aims to determine whether randomly sampled convalescent COVID-19 plasma contains immune activity capable of neutralizing hedgehog merbecoviruses. We utilized available sera from 50 individuals who had recovered from SARS-CoV-2 infection.
Reporting on race, ethnicity, or other socially relevant groupings	All sera samples were collected from individuals of East Asia (Chinese) background.
Population characteristics	Convalescent plasma samples were randomly sampled from 50 individuals who had previously recovered from SARS-CoV-2 infection. The cohort represents a heterogeneous convalescent population with varying infection times, numbers of infection episodes, and diverse COVID-19 vaccination histories. The median age of the donors was 47.5 years, with 36% male and 64% female. The gender, age, vaccination status, infection time, and sampling time were listed in Supplementary Table 6.
Recruitment	The volunteers were recruited by Beijing Ditan Hospital, Capital Medical University. All participants provided written informed consent before sample collection. The convalescent individuals were randomly sampled without applying additional selection criteria regarding their prior infection episodes or vaccination histories. Participants received reimbursement for transportation expenses related to blood sample collection.
Ethics oversight	Ethical approval for the plasma collection was granted by the Ethics Committee of Beijing Ditan Hospital, Capital Medical University, with approval number DTEC-KY2024-112-01. All participants provided written informed consent prior to sample collection.

Note that full information on the approval of the study protocol must also be provided in the manuscript.

Field-specific reporting

Please select the one below that is the best fit for your research. If you are not sure, read the appropriate sections before making your selection.

☒ Life sciences ☐ Behavioural & social sciences ☐ Ecological, evolutionary & environmental sciences

For a reference copy of the document with all sections, see nature.com/documents/nr-reporting-summary-flat.pdf

Life sciences study design

All studies must disclose on these points even when the disclosure is negative.

Sample size

We assessed the capacity of 93 mammalian and avian aminopeptidase N (APN) orthologs to mediate viral binding and entry. APN sequences were retrieved from the NCBI GenBank database by querying for 'aminopeptidase N' and 'APN'. For species without reference genomes—including the Asian hedgehog (Erinaceus amurensis), African hedgehog (Atelerix albiventris) and hairy hedgehog (Hylomys suillus)—sequences were assembled de novo from transcriptomic data. The final panel encompassed 10 Chiroptera, 12 Rodentia, 13 Primates, 15 Carnivora, 3 Pinnipedia, 2 Perissodactyla, 11 Artiodactyla, 6 Cetacea, 6 Eulipotyphla, 10 Aves, 2 Afrotheria, and one species each from Diprotodontia, Lagomorpha and Pholidota. This broad taxonomic coverage provides a comprehensive view of the cross-species transmission potential.

In this study, twenty-seven merbecoviruses of hedgehog were obtained from the NCBI GenBank database by querying for 'hedgehog coronavirus', 'HKU31' and 'Erinaceus coronavirus'. This dataset comprised all publicly available hedgehog coronavirus genome publicly available at the time of submission, capturing substantial phylogenetic diversity among EriCoV lineages. This comprehensive sampling enabled us to assess the broad utilization of hedgehog APN across diverse hedgehog coronaviruses.

In serum neutralization assays, we used 50 SARS-CoV-2 convalescent plasma, to demonstrate the critical and specificity neutralizing activity of these serum against EriCoVs. The convalescent plasma samples were randomly sampled from 50 individuals who had previously recovered from SARS-CoV-2 infection.

No statistical methods were used to pre-determine sample sizes. No sample size calculation was performed, the rationale for these sample sizes were sufficient is described above and are similar to those reported in previous publications (PMID: 39922191,41168429, 35714668).

Data exclusions

There is no data excluded from the analysis.

Replication

The majority of experiments were conducted 2 to 4 times, with 2 to 6 replicates (most are triplicated).

Randomization

Human serum samples were collected from randomly selected donors. Randomization was not applicable to other experiments, as experimental groups and conditions were predefined according to the study design.

Blinding

Data collection and analysis were not performed blind to the conditions of the experiments.

Reporting for specific materials, systems and methods

We require information from authors about some types of materials, experimental systems and methods used in many studies. Here, indicate whether each material, system or method listed is relevant to your study. If you are not sure if a list item applies to your research, read the appropriate section before selecting a response.

Materials & experimental systems

n/a	Involved in the study	
☐	☒	Antibodies
☐	☒	Eukaryotic cell lines
☒	☐	Palaeontology and archaeology
☐	☒	Animals and other organisms
☒	☐	Clinical data
☒	☐	Dual use research of concern
☒	☐	Plants

Methods

n/a	Involved in the study	
☒	☐	ChIP-seq
☐	☒	Flow cytometry
☒	☐	MRI-based neuroimaging

Antibodies

Antibodies used

Neutralizing Antibodies:
Anti-VSV G Mouse monoclonal Antibody(I1), I1-hybridoma supernatant from the I1-Hybridoma mouse cell line (ATCC: CRL-2700, Neutralizing, 1 μg/mL);
Anti-ACE2 Human monoclonal Antibody(#12) (Neutralizing, 10 μg/mL);

Primary antibodies:
Anti-Flag Mouse Sigma, F1804/clone M2 (Cat: F1804-1MG, Sigma-Aldrich) (WB, 1:10,000; IFA,1:1,000);
Anti-HA-tag mAb (Cat: M180-3, MBL) (IFA,WB, 1:5,000);
Anti-ALFA-tag Nanobody-hFc-recombinant protein(IFA,3 μg/mL);
β-Tubulin Monoclonal Antibody(5G3) (Cat: YM3030, Immunoway) (WB,1:10,000);
Anti-VSV-M [23H12] Antibody (Cat: EB0011, kerafast) (WB,1:10,000);
Anti-His tag Antibody, Mouse Monoclonal (Cat: 105327-MM02T, SinoBiological) (WB, 10,000).
β-actin, Mouse Monoclonal Antibody (Cat: 30101ES50, YEASEN) (WB, 1:10,000)

Secondary antibodies:
Alexa Fluor 594-conjugated goat anti-Mouse IgG, Goat, Thermo Fisher Scientific, A32742 (IFA, 1:1,000);

DyLight 594-conjugated goat anti-Human lgG, Goat, Thermo Fisher Scientific, SA5-10136 (IFA, 1:1,000);
Alexa Fluor 488-conjugated goat anti-Human lgG, Goat, Thermo Fisher Scientific, A11013 (IFA,1:1,000);
Alexa Fluor 488-conjugated goat anti-Mouse lgG, Goat, Thermo Fisher Scientific, A32723 (IFA,1:1,000);
FITC-conjugated goat anti-Mouse lgG, Goat, Thermo Fisher Scientific, A16079 (IFA,1:1,000);
Goat anti-mouse lgG(H+L)HRP, Goat, JACKSON, 115-035-003 (WB, 1:10,000);

The enriched B cells staining antibodies:
FITC anti-mouse CD19 Antibody, Mouse, 6D5, BioLegend, 115506 (FAC, 1:500);
PE/ Cyanine7 anti-mouse IgD Antibody, Mouse, 11-26c.2a, BioLegend, 405720 (FAC, 1:500);
PE/ Cyanine7 anti-mouse IgM Antibody, Mouse, RMM-1, BioLegend, 406514 (FAC, 1:500).
All monoclonal antibodies were generated by amplifying variable regions from single B cells via RT-PCR and cloned into appropriate expression plasmids (pCMV3-CH, pCMV3-CL, or pCMV3-CK) containing human or mouse IgG1 constant regions, followed by expression in CHO cells.

Validation

Anti-Flag Mouse Sigma, F1804/clone M2 (Cat: F1804-1MG, Sigma-Aldrich). Vendor validated highly sensitive and specific detection of FLAG fusion proteins by WB/IP/IHC/IF/ICC. Optimized for single banded detection of FLAG fusion proteins in mammalian, plant, and bacterial expression systems. https://www.sigmaaldrich.cn/CN/en/product/sigma/f1804
Anti-HA-tag mAb (Cat: M180-3, MBL). Vendor validated application for WB/IP/FCM/FCM. https://www.mblbio.com/bio/g/dtl/A/?pcd=M180-3
Anti-ALFA-tag Nanobody-hFc-recombinant protein(IFA, 3 μg/mL), in-house generated. A nanobody (NbALFA) binding ALFA-tagged proteins from native or fixed specimen with low picomolar affinity. That was verified in this paper: DOI: 10.1038/s41467-019-12301-7.
β-Tubulin Monoclonal Antibody(5G3)(Cat: YM3030, Immunoway). Vendor validated application for WB/IF/IHC. Reactive with B-Tubulin from different species (Human/Rat/Mouse/Mk/Dg/Ch/Hamster/Rabbit/sheep). http://www.bionewway.com/CHome/22/YM3030
β-actin, Mouse Monoclonal Antibody (Cat: 30101ES50, YEASEN). Vendor validated application for WB. Reactive with β-actin from different species (Human/Mouse/Monkey/Goat/Rat/Hamster/...). https://www.yeasen.com/products/detail/877?bingpc=kw
Anti-VSV-M [23H12] Antibody (Cat: EB0011, kerafast). Vendor validated application for high sensitivity of VSV-M for WB detection. https://www.kerafast.com/productgroup/1011/anti-vsv-m-23h12-antibody?ProductID=445
Anti-His tag Antibody, Mouse Monoclonal (Cat: 105327-MM02T, SinoBiological). Vendor validated application for high sensitivity of His-Taq for ELISA detection. https://www.sinobiological.com/antibodies/his-tag-105327-mm02t

Goat anti-human IgG (H+L)-HRP (JACKSON, 109-035-003): Based on immunoelectrophoresis and/or ELISA, the antibody reacts with the whole molecule human IgG molecule. It also reacts with the light chains of other human immunoglobulins. No antibody reactivity was detected against non-immunoglobulin serum proteins. The antibody may cross-react with immunoglobulins from other species.
PE/Cyanine7 anti-mouse IgM Antibody (BioLegend, Cat. No. 406514) was validated by successful staining and flow cytometry (FC) analysis according to the manufacturer's website https://www.biolegend.com/en-us/products/pe-cyanine7-anti-mouse-igm-6935 and previous publication: Erickson JJ, et al. 2022. Nature. 606:769.
PE/ Cyanine7 anti-mouse IgD Antibody (BioLegend, 405720) was validated by successful staining and flow cytometry (FC) analysis according to the manufacturer's website https://www.biolegend.com/en-us/products/pe-cyanine7-anti-mouse-igd-8398 and previous publication: Melzi E, et al. 2022. Immunity. 55:2168.
FITC anti-mouse CD19 Antibody (BioLegend, 115506) was validated by successful staining and flow cytometry (FC) analysis according to the manufacturer's website https://www.biolegend.com/en-us/products/fitc-anti-mouse-cd19-antibody-1528 and previous publication: Ramakrishna C, et al. 2019. Nat Commun. 10:2153.

Antibody #12, an antibody that recognizes human ACE2 and can cross-recognize hedgehog ACE2, has been validated by previous experiments conducted in Xiangxi Wang laboratory, and the relevant data are currently being compiled for publication.

In this study, we generated a total of 204 antibodies (54 targeting HKU31-MO1-RBD, 67 targeting RU15-1-RBD, and 83 targeting E.amu APN). All antibodies were expressed in CHO cells using mouse IgG1 constant regions. The binding of each antibody to its corresponding antigen was validated by ELISA, and its neutralization activity was confirmed using VSV-based pseudotyped virus assays.

All other antibodies are validated by the vendors.

Eukaryotic cell lines

Policy information about cell lines and Sex and Gender in Research

Cell line source(s)

HEK293T (CRL-3216), Caco2 (HTB-37) , BHK-21 (CCL-10), and I1-Hybridoma (CRL-2700) were purchased from ATCC.

Authentication

Not authenticated.

Mycoplasma contamination

Cell lines used in this study were not tested for mycoplasma contaminatlon.

Commonly misidentified lines
(See ICLAC register)

No commonly misidentified line was used.

Animals and other research organisms

Policy information about studies involving animals; ARRIVE guidelines recommended for reporting animal research, and Sex and Gender in Research

Laboratory animals	For animal immunization, thirty female BALB/c mice aged 6-8 weeks were used in the experiments. Mice were immunized with RBD proteins or APN mRNA for 2-3 times, then spleens were harvested for single-B-cell sorting and subsequent antibody characterization. Mice were housed under specific pathogen-free (SPF) conditions with a 12-h light/dark cycle, an ambient temperature of 20–26°C, and a relative humidity of 40–70%. Animals were group-housed (five mice per cage) and had ad libitum access to sterilized chow and water throughout the study period.<br><br>For primary hedgehog cell isolation, African pygmy hedgehog (Atelerix albiventris, 12 months of age, female) was purchased from a licensed commercial pet supplier in Beijing, China, on 11 October 2025. The animal was outbred, commercially bred individuals with the standard wild-type coat coloration. Prior to the experiment, the animal was clinically healthy and tested negative for common pathogens of this species, including Salmonella spp., dermatophytes, ectoparasites, and intestinal parasites. All primary cells used in this study were derived from a single individual. For animal anesthesia, tiletamine-zolazepam (Virbac) was administered intramuscularly at a dose of 15 mg/kg body weight as anesthesia prior to euthanasia. According to the product label and relevant veterinary advice, a pilot experiment was conducted using 10 mg/kg (the lower limit of this range) as the starting dose. The dose was then titrated according to the depth of anesthesia, the animals' stress responses, and the operational requirements of tissue collection, and a final dose of 15 mg/kg was established for the formal experiments. Following confirmation that the animal had achieved a stable, deep surgical plane of anesthesia, terminal exsanguination was performed to complete euthanasia, followed by surgical dissection and tissue collection. All procedures were conducted in strict compliance with institutional laboratory animal care and use guidelines and applicable animal welfare standards. No hedgehog housing was involved in this study.
Wild animals	This study did not involve wild animals.
Reporting on sex	The sex of laboratory animals is not relevant to the objective of this study. Only female BALB/c mice are used for immunization in the study because the target of this study (immunization of antigens and expression of antibodies) should not be significantly affected by sex. Both male and female BALB/c mice are used in the related studies. Only a female african pygmy hedgehog was used for primary cells isolation in the study.
Field-collected samples	This study did not involve samples collected from the field.
Ethics oversight	All mouse immunization-related experiments were conducted in accordance with protocols approved by the Animal Welfare Ethics Committee of HFK Biologics with an approval number of HFK-AP-20250313. All hedgehog related experiments were authorized by the Institutional Animal Care and Use Committee (IACUC) of the Institute of Laboratory Animal Science (ILAS), Chinese Academy of Medical Sciences (CAMS) & Peking Union Medical College (PUMC), with an approval number of BLL25006. All procedures were conducted in strict compliance with institutional and national ethical guidelines for animal welfare.

Note that full information on the approval of the study protocol must also be provided in the manuscript.

Plants

Seed stocks	n/a
Novel plant genotypes	n/a
Authentication	n/a

Flow Cytometry

Plots

Confirm that:

- [x] The axis labels state the marker and fluorochrome used (e.g. CD4-FITC).
- [x] The axis scales are clearly visible. Include numbers along axes only for bottom left plot of group (a 'group' is an analysis of identical markers).
- [ ] All plots are contour plots with outliers or pseudocolor plots.
- [x] A numerical value for number of cells or percentage (with statistics) is provided.

Methodology

Sample preparation	To analyze RBD–hFc binding by flow cytometry, HEK293T cells transiently expressing the indicated APN orthologues were detached with 5 mM EDTA/PBS 24 hours post-transfection. For antibody–APN competition assays, RU15-1 or MO1 RBD–hFc proteins were pretreated with indicated antibodies (10 µg/mL) at 37°C for 1 hour. The detached cells were incubated with RU15-1 or MO1 RBD–hFc proteins (2 µg/mL) at 4°C for 1 hour. After two PBS washes, cells were stained for 1 hour at 4°C using DyLight 594-conjugated goat anti-human IgG antibodies (Thermo Fisher Scientific; SA5-10136) to detect RBD binding. Following two additional PBS washes, 15,000 live cells for RBD–hFc binding assays, or 10,000 live cells for antibody–APN competition assays (gated based on SSC/FSC) were analyzed on a CytoFLEX flow cytometer (Beckman).<br><br>For single B cells sorting, spleens from euthanized mice were minced in RPMI 1640 medium supplemented with 5% FBS, filtered through a 100 µm mesh, and treated with 1 × RBC Lysis Buffer (eBioscience, cat. no. 00-4333-57). After centrifugation (350 × g, 8min), cells were resuspended in staining buffer (PBS + 2% FBS) and adjusted to a final concentration of 1×10^7 cells/mL. Recombinant biotinylated RBD proteins (MO1 or RU15-1 strain) or E.amu APN proteins were incubated with streptavidin-conjugated fluorophores (PE and APC; BioLegend) on ice for at least 30 minutes. Then H_2O was added to adjust volume, and the complexes were centrifuged at 14,000 × g for 10minutes at 4°C to remove aggregates. Before staining, splenocytes were incubated with TruStain FcX™ (BioLegend) for 10minutes on ice in the dark to block Fc receptors. Antigen probes and fluorescently conjugated antibodies were then added, and cells were incubated on ice for 30minutes in the dark. After staining, cells were washed twice with staining buffer (400 × g, 5–8 minutes) and resuspended in cold buffer. 7-AAD (eBioscience, cat. no. 00-6993-50) was added 10 minutes before flow cytometry to exclude dead cells.
Instrument	CytoFLEX Flow Cytometer (Beckman Coulter,United States). Single B cells were sorted using a MoFlo Astrios EQ Cell Sorter (Beckman Coulter) directly into 96-well PCR plates.
Software	Summit 6.0 (Beckman Coulter) for cell sorting; FlowJo version 10 for data analysis.
Cell population abundance	For RBD-hFc binding assays, 15,000 events in the gated area were analyzed for all samples; for antibody–APN competition assays, 10,000 events in the gated area were analyzed for all samples. Detailed abundance data that were used for sorting are shown in the Supplementary Information.
Gating strategy	For RBD-hFc binding assays, 20,000-50,000 total events were recorded for each sample. Gating was initially performed to exclude cell debris and dead cells (FSC-H/SSC-H) for subsequent analysis (P1). Gating thresholds were established using histogram profiles of mock controls, which consisted of HEK293T cells transfected with empty vector plasmids or non-relevant receptor plasmids. Subsequently, 15,000 events within the P1 population were recorded for binding efficiency analysis (positive for DL594). For antibody–APN competition assays, Secondary gating, based on ZsGreen-positive cells, was then conducted to distinguish cells expressing detectable hedgehog APN (P2). Gating thresholds were established using histogram profiles of mock, which consisted of HEK293T cells transfected with empty vector plasmids. Subsequently, 10,000 events within the P2 population were recorded for antibody–APN competition analysis (positive for DL594). The representative gating strategy is demonstrated in Supplementary Fig. 6.<br><br>For cell sorting, antigen-specific B cells were defined as 7-AAD$^-$, FITC-CD19$^+$, PE Cy7-IgM$^-$/IgD$^-$, Bv605-OVA$^-$, and either MO1 RBD$^+$, RU15-1 RBD$^+$, or E.amu APN$^+$, depending on the immunization group. The representative gating strategy is demonstrated in Extended Data Fig. 8a.

☒ Tick this box to confirm that a figure exemplifying the gating strategy is provided in the Supplementary Information.
