# Supplementary material for: Convergent use of aminopeptidase N receptor by hedgehog merbecoviruses
Source: Nat Microbiol. 2026 Sep 16;11(10):2821–38. doi: 10.1038/s41564-026-02478-1 (PMC13716839; doi:10.1038/s41564-026-02478-1)
Supplement: Supplementary file 6 [file 41564_2026_2478_MOESM3_ESM.pdf]

nature portfolio

Peer Review File

Convergent use of Aminopeptidase N receptor by hedgehog merbecoviruses

Corresponding Author: Professor Huan Yan

Version 0:

Decision Letter:

5th February 2026

Dear Professor Yan

Thank you for submitting your Article entitled "Convergent utilization of APN receptor by hedgehog merbecoviruses" for consideration in Nature Microbiology. I'm writing to let you know that we have decided to send your manuscript for peer review, however we cannot do so in its present form.

Therefore, we would kindly ask you to please provide us with structural maps and model files for evaluation by the referees. You can provide a GDrive link or use the figshare repository.
You can use the figshare integration for that purpose (via the ‘Research Data Deposition’ tab when resubmitting manuscript). Please note that the pdb. files should be clearly labelled "for manuscript peer review", currently several files are NOT for manuscript review.

Data are stored privately until a manuscript decision is reached and you can edit/withdraw them up to this point: you retain rights and control over your data. **Datasets up to 50GB can be submitted via the manuscript submission system.** However, upload time and capacity is dependent on internet connection. If you have a larger dataset you would like to share, this would have to be managed outside the integrated application and additional storage fees may apply (see figshare’s large data service, figshare). Contact research.data@springernature.com for more details.

In addition, we are trying to improve the quality of methods and statistics reporting in our papers. To that end, we are now asking authors to complete a reporting summary that collects information on experimental design and reagents.

The Reporting Summary can be found here https://www.nature.com/documents/nr-reporting-summary.pdf)

Please complete the relevant form and upload it with the resubmission. Please note that this form is a dynamic ‘smart pdf’ and must therefore be downloaded and completed in Adobe Reader. We will then flatten it for ease of use by the reviewers. If you would like to reference the guidance text as you complete the template, please access these flattened version at www.nature.com/authors/policies/availability.html

Please be aware of our guidelines on digital image standards.

Note that you are not required to revise your paper to include the information provided in the reporting summary.

Once we receive these documents and review them to ensure that all requested information is provided, we will proceed to send your paper for review. If you have questions or anticipate delays, please let me know as soon as possible.

Please use the link below to submit a revised paper:

Link Redacted

We hope to receive the manuscript and the requested files within 48 hours. Please just let us know should you need more time for resubmission.

Many thanks and best wishes,

Version 1:

Reviewer comments:

Reviewer #1

(Remarks to the Author)
Liu and colleagues report that European and Asian hedgehog merbecoviruses (EriCoVs) use hedgehog aminopeptidase N (APN) as their functional receptor, with a narrow APN ortholog tropism restricted to hedgehogs and some felids, and rely on hedgehog TMPRSS2 for spike activation and fusion. The authors used comparative genomics, a 139-species APN ortholog panel, cryo-EM of RBD–APN and antibody–RBD complexes, and pseudovirus/rcVSV entry and spread assays to define the molecular determinants of receptor usage, host range and entry routes. They also generated neutralizing antibodies against both EriCoV RBD and hedgehog APN that block EriCoV entry in APN-expressing human cells and primary hedgehog cells. This integration of genomics, structural virology, receptor biology, and antibody engineering is a clear strength and supports a translational angle for pandemic preparedness. This is an impressive work, adding APN to the repertoire of betacoronavirus receptors, previously governed by DPP4 and ACE2. The authors also demonstrate a novel APN-binding mode distinct from that of 229E. I don't have any major concerns.
Main comments
1. These results are in line with a similar work by Jin et al, available at BioRxiv. This other study should be discussed more precisely, highlighting the common results.
2. The conversion of hAPN into an EriCoV receptor via 5–6 mutations is interesting; adding functional data on how these mutations affect 229E usage of APN would clarify whether gain of EriCoV binding trades off against other APN–CoV interactions. This should be at least further discussed.

Reviewer #2

(Remarks to the Author)
The authors have produced a comprehensive, logical, and easy to follow stream of experiments assessing the receptor binding of EriCoVs.
They identify hedgehog APN (also known as ANPEP) as the receptor primary receptor for the virus. In a reasonably comprehensive analysis they test the ability of other species' APN able to mediate fusion or infection of pseudovirus.

They find trypsin, in the full-lengths version to have signficant impact on fusion and infection. They also do a limited analysis of proteolytic activation, i.e. limited to TMPRSS2 overexpression experiments and broad-range protease or lipopeptide fusion inhibitors. This experiment is the weakest one in the manuscript. Effect of TMPRSS2 is marginal and not brought into context of a pseudovirus (VSV) that normally enters through Clathrin-mediated endocytosis also due to its size and shape. I have suggested further experiments in the respective section and also would urge to tune down the TMPRSS2 wording in the summary section as trypsin also is highly potent to activate this virus.

Like many of these papers, the authors have a very strong focus on human spillover but negate to look at the immediate biology of infetion in the target species and transmission to species that interact closely. I have highlighte this in the section relating to proteolytic activation but also relating to APN species binding. Looking at hedgehog predator species for example would have been interesting. The latter experiments would be interesting to be performed but not exxential. The proteolytic activation experiments, especially when heading the respective section as such, should be added.

One more minor thing I would like to see are western blots on the mutant APN proteins to show not only that the tag can be stained in a cell but that the protein is not degraded. This would be supplementary data and is indicated further down with respect to the relative figures.

Graphical abstract:
I was wondering what made the authors chose to highlight PRCV specifically for the APN binding, whereas this is binding to the Alphacoronavirus Suis species in general as they highlight for all the EriCoVs in general. At least it should be broader than PRCV since PRCV is a deletion mutant of TGEV, which is evolutionary “parental”. If we are going down that path, it should even be CCoV as an evolutionary ancestral strain. However, I think αCoV Suis would be most applicable as the binding modes are highly similar.

Summary:
As highlighted later in the respective section, I believe TMPRSS2 functionality here is overinterpreted and should be worded more cautiously here. Just because PM fusion is enhanced, doesn't necessarily mean it's biologically relevant. Especially when using pseudovirus systems based on a different virus family as well.

Introduction
Section Line 63fff: Please also highlight what the biology is of EriCoVs in hedgehogs. What is known about their impact in the species itself; what is the infection and transmission route if known?

Line 76: "cross species transmission potential"

Results
Line 182: It would suggest clarifying the experiment for 2a a bit – "supported detectable binding of Fc-tagged RBD protein (Fig. 2a)". Quantification here could have been nice i.e. only positive receptor expressing cells show binding staining but it is pretty black and white so that I don't think this is essential.
Similarly, in the figure legend, I presume binding was also confirmed with immunofluorescence staining? Please add this and also compare with Figure 3 legend and make sure the same experiments have at least similar and independently comprehensible legends. In 3 for example you highlight RBD-Fc is green, not in 1.

Line 183: This is the first introduction of the pseudoviruses in the main body of text. Pseudoviruses can be made on a variety of backgrounds that impact entry pathways. Therefore, please introduce that this is a VSV-based pseudovirus used in these experiments and for all the following experiments. I would urge to add this information to all the figure legends where pseudoviruses are used, i.e. VSV-pseudovirus mentioned at least once in the legend to each figure where the authors used pseudoviruses.

Line 185: Interesting that the authors chose 229E RBD rather than any of the arguably more closely related APN-binding coronaviruses. Maybe highlight that this reciprocal experiment is limited by adding a line; No other APN-binding coronaviruses were tested in this experiment. (Just to remind the reader immediately that there are other APN-binding coronaviruses that may bind to hedgehog APN. In case I forget this later, worth discussing this in the discussion as well that recombination events could be reciprocal as well, i.e. hedgehogs being infected by a virus from a more closely interacting species, for example badgers, natural predators of hedgehogs, who recently were shown to carry an alphacoronavirus; 10.1016/j.meegid.2023.105406).

Line 189: I would suggest that the authors use the word peptidase NOT protease activity. Whilst technically proteolytic, it's a term not quite appropriate in the common use of protease activity.

Lines 192 fff and figure 2 h & i: I like the figures and the results, clearly indicating a possible and likely enteric infection route of this virus (would have been interesting to see whether pepsin has similar activity). Nonetheless, I would just highlight either in the results or the figure legend some notes about cell health in the face of such high levels of trypsin. The black shades in the background show significant clumping up of cells; I am actually surprised that

Ortholog specificity and host tropism determinants
Line 234: suggest adding "APNs from shrews Sorex … "

Line 247: suggest "all tested species of ERiCoVs"

Line 249: suggest "the GER216, FR7137, and PLJ64 strains [or strain spikes] are also capable …"

Figure 3: For experiments related to the chimera figures, 3f, j, and i; I would like the authors to show a western blot with these chimeras in the supplementary data, just to show these proteins do not show signs of misfolding and degradation. Whilst these two species show about 76% sequence identity, some of the areas are significantly different that this must be a concern.
It is a real shame that the authors didn't include biologically important species in this experiment as well, i.e. main predator species of hedgehogs, such as badger species. That would be an important assessment for risk of cross species transmission.

Line 262: highlight which species contains the glycosylation site.

Line 267: I think the statement in 267 is too definite. Given the authors haven't done further experiments with recombinants of F. Cat or P. Par they cannot say this "likely explains their receptor compatibility." I would say "could be an explanation for their receptor compatibility." Furthermore, in relation to figure k: How did the authors decide on critical residues involved in host range determination? I cannot quite follow the thought process here, for example, I kind of understand the first stretch of 6 amino acids, though this has not been experimentally validated, e.g. swapping this section into feline APN may be very insightful? In the latter region, 326 I can again not follow. There is a clear difference in hedgehog but why this may be determining I don't understand. Do the boxes here reflect the alpha2 and beta 16 regions at all? More rationale and explanation in the results section is needed here.

Note on both structural sections; can you ensure it is clear which amino acid residues you are numbering in the different sections? I presume in the "Ortholog specificity and host tropism determinants" it is always the human aa residue number you are highlighting whereas it is or possibly should be the hedgehog, i.e. E. amu residue in the "Structure basis of E.amu APN recognition by EriCoV" section.

Structure basis of E.amu APN recognition by EriCoV
Pretty straightforward structural analysis chapter. Similar to my previous note on figure 3, it would be good to also see western blots on the mutant protein expression.

For the experiment relating to figure 4h, I refer back to multiple ones of my previous comments; this section remains hard to follow since the explanation and choice of residues related to figure 3k were not made very clear. Some of these residues are now highlighted in figure 4b, which makes the non-verbalised rationale clear now based on structural information. The slight issue here is that in figure 3k, residues are numbered with the human residue numbers whereas now the hedgehog residues are used. It may be good for the sequence alignment in 3k to display both so that the link to figure 4h would be clear. I would also suggest somewhere around line 360 to refer back to figure 3k and you must highlight that these aa changes were based on mutations to the human aa residue in that alignment position. Easiest if referring to figure 3k.

EriCoV spike proteolytic activation

I will repeat myself further down in this section as I have only just added this general note. However, the authors have put a very generic title on this section yet have done a fraction of what they promise. What they have primarily looked at is functional potential of TMPRSS2 in EriCoVs but not broader proteolytic activation studies. If one looks at proteolytic activation in MERS-CoV, the virus is very amenable to a broad range of proteolytic activation (10.1073/pnas.1407087111). For a thorough study, and what is really warranted her, the authors need to look at a broader spectrum of proteolytic activation. They already have all the general assays in place; it would really only take an expansion of figure 5a, or broader fusion assay tests. I think this section is lacking and needs extension beyond TMPRSS2. Furthermore, the reasonably marginal effect that TMPRSS2 produces does not warrant the absolute statement of TMPRSS2 needs in the abstract and needs to be tuned down or extended once further proteolytic activation studies have been performed.

Line 413: The facilitation of S protein activation is not always merely a facilitator for S protein activation but can also be, for many coronaviruses, be a requirement determined by the macroenvironment for tropism of infection and stability. Many of the enteric tropism coronaviruses rely on the stability from a non or not completely proteolytically processed spike protein. Activation by macroenvironment proteases, such as pepsin or trypsin in the small intestine, or microenvironment proteases in the endocytic vesicular system are crucial for successful infection of many coronaviruses. For all intents and purposes, what we know about merbecoviruses is that they are enteric in hedgehogs so this is important to remember when discussing proteolytic processing.

Lines 414ff: This comment links to the previous comment; the authors like to make the link back to another merbecovirus here – understandably. However, “inefficient” particle processing during particle biogenesis is completely normal for some coronaviruses. In particular the ones entering through an oro-fecal route, e.g. PEDV, are not proteolytically processed upon assembly and release. It is only upon trypsin treatment that PEDV for example gets processed (e.g. 10.1128/JVI.00297-14). Just as a cautionary sentence here as the focus seems to be on plasma membrane mediated fusion for this entire section whilst working with a) a pseudovirus only and b) in a very artificial cell and macroenvironment system for this virus.

Line 424: I would reword that to: “functions as an accessory factor only to the APN receptor.”

Figure 5 and line 427ff: I understand the general rationale the authors employ here basically using trypsin as a generic proteolytic activation tool to enhance entry of this specific coronavirus. What seems to be neglected in the general context is that trypsin may very well be the true protease that may activate EriCoVs in vivo. They do their best to find alternative proteases but for example, don’t show what happens to EriCoV spikes upon protease treatment in figure 5a. To be truly comprehensive looking at what the section title suggests I would urge the authors to perform proteolytic activation assays with common proteases including cathepsins, trypsin, pepsin, plasmin, PC1, Xa, etc. Think about papers like the one by Millet & Whittaker on MERS (10.1073/pnas.1407087111) and 10.1038/s41586-025-09155-z, extended data figure 5.

Please also bare in mind that for some of the enteric coronaviruses trypsin does not merely function as a pre-activator but viruses very much require presence of trypsin during entry (e.g. PEDV, 10.1128/JVI.00297-14). Please caveat this in the biological context also in relation to low level infection that is observed.

Line 432fff: E64d is a cys protease inhibitor and whilst it inhibits cathepsins, it also inhibits a broad array of other cellular proteases. Please clarify this here – e.g. the cys-protease inhibitor E64d, used here as a proxy to assess potential Cathepsin involvement. Similarly, Camostat inhibits a variety of serine proteases, including TMPRSS2, but also trypsin, plasmin, Xa, and others. These are not specific assays and must be caveated as such. If a broader proteolytic activation panel would be performed for 5a, then maybe broader conclusions could be reached here. Camostat is also cell permeable so it cannot be assumed it only acts on surface proteases.
Furthermore, maybe expand that EK1C4 (eventually in line 453) is really a lipopeptide fusion inhibitor that can act on PM and endocytic fusion inhibition.

Line 438: Any rationale why the authors now moved to CaCo-2 cells? Human enteric cell line? (also not a fully differentiated cell line so it can be polymorphic). Also please specify that “can be boosted with trypsin pre-treatment of the pseudovirus” unless in this case it is treatment during infection?

Figure 5f, g and associated results section 436 fff: I do only partially agree with the author’s interpretation here. The fact that trypsin treatment boosts infection (significantly) and TMPRSS2 presence at the surface boosts infection slightly – 3-5x – does not support a strong involvement of TMPRSS2. In my opinion, it supports a conclusion that proteolytic activation upon receptor binding may be required for successful infection. A role that can be partially met by an OVEREXPRESSION of TMPRSS2 but may also be met by a macroenvironmental protease in vivo (i.e. trypsin, pepsin, etc). The only thing I conclude from the experiment in figure 5 is that TMPRSS2 is marginally enhancing infection here but that other proteases, similar to MERS but also similar to other (possibly enteric) coronaviruses, are playing a role as the E64d treatment and camostat treatment show. Namely, in the absence of TMPRSS2 overexpression (figure 5g), infection is far stronger with Camostat treatment than it ever is in the presence of TMPRSS2 with Camostat treatment. This actually indicates that TMPRSS2 may be having negative effects on infection rather than aiding it. And that surface fusion is possibly not a successful infection route for the pseudovirus. But please bare in mind this is a system based on VSV, which generally goes through clathrin-mediated endocytic uptake and this may not be as relevant for the actual EriCoV.

Neutralizing antibodies against EriCoV
I don’t have much to say on this experiment. Monoclonal antibody therapies have shown limited long-term efficacy in preventing spread but can aid individual patients and could possibly help in a small localised outbreak scenario.

Discussion
The discussion section is short and I would like to see a bit more discussion of the risk of cross-species spillover. Discussion of the binding pocket and what the risks are of spillover in marginally susceptible species, such as felines and rats. As highlighted

earlier in the respective section, it is a shame that the authors did not assess the susceptibility of natural predators of hedgehogs, such as badgers in the biological context.

Similarly, once proteolytic activation has been studied a bit more broadly, I would like to see some discussion on the route of transmission and infection (based on the biological

Reviewer #3

(Remarks to the Author)
Liu et al. demonstrate that European and Asian hedgehog coronaviruses (EriCoVs) utilize hedgehog aminopeptidase N (APN) as a physiological receptor. Using a broad panel of EriCoV spike proteins and APN-expressing cells, combined with cryo-EM structural analysis, the authors provide a comprehensive study. These analyses are excellent and impressive, offering clear evidence for receptor characterization. However, I have the following comments to address.

Major Comments
1. The authors found that R. norvegicus (R.nor) APN did not support RU15-1 or MO1 binding (via Fc-fusion RBD proteins) and only exhibited detectable binding with the FR7137 RBD. However, the constructs for the Fc-fusion proteins appear inconsistent across species; some include the entire S1 domain, while others include only the RBD. The S1 domain alone can be particularly unstable, and the exposure of hydrophobic interfaces (originally the S2 interface) might facilitate non-specific binding. The authors should clearly state these differences in the expressed domains and discuss how this species specificity relates to amino acid substitutions in both the APNs and the RBD/S1 domains, based on their cryo-EM structures.
2. Details regarding the recombinant Spike constructs used for cryo-EM are missing. The authors should clarify whether they introduced stabilizing mutations, such as prolines. If such mutations were used, they might account for the dominance of the "closed" conformation.
3. The PPI2 prediction is neither cited nor fully described. It is unclear which tool was used and whether the prediction is reliable.

Minor Comments
1. A figure showing an amino acid sequence comparison of APNs and EriCoVs, including a binding counter/matrix, would greatly assist the reader's understanding.
2. Lines 255-256: The sentence "Substituting domain 2 of E. amu APN with its human counterpart abolished binding, while substituting E. amu Domain 2 with human APN counterpart restored binding..." is confusing. Please clarify the direction of these substitutions.
3. The inconsistent use of "hAPN/human APN" and "hTMPRSS2/human TMPRSS2" may be confusing. Since proteins from various species are compared throughout the manuscript, I suggest unifying the terminology to "human APN" and "human TMPRSS2" for clarity.
4. Lines 310-311: To avoid confusion with the "closed" state of the Spike protein (even though only the RBD was used), the phrase "...ensuring that all E. amu APN particles adopted a 'closed' conformation" should perhaps be revised to "...ensuring that all E. amu APN molecules in the particles adopted a 'closed' conformation."
5. Figure 3g: Two shifted peaks are observed for WT E. amu APN with MO1-RBD, whereas only one is observed with RU15.1. The authors should provide a comment on this observation.
6. Figure 4 (panels a, b, or c?), its legend, and the related text (lines 319-330): The authors should indicate or mention K310, which corresponds to the N-glycosylation site in human APN.
7. Figure 4f: For a clearer comparison of the receptor-binding interfaces, the RBDs in all complexes should be displayed at the same angle.

Decision Letter:

20th March 2026

*Please ensure you delete the link to your author homepage in this e-mail if you wish to forward it to your co-authors.

Dear Professor Yan,

Thank you for your patience while your manuscript "Convergent utilization of APN receptor by hedgehog merbecoviruses" was under peer-review at Nature Microbiology. It has now been seen by 3 referees, whose expertise and comments you will find at the end of this email. Although they find your work of some potential interest, they have raised a number of concerns that will need to be addressed before we can consider publication of the work in Nature Microbiology.

Therefore, we recommend that you provide additional clarifications, explanations, and quantitation as asked by the referees for various different aspects of the work. We feel that proteolytic activation assays with some common proteases asked by R2 is not an absolute requirement. Should further experimental data allow you to address these criticisms, we would be happy to look at a revised manuscript with a detailed point-by-point response to the referee comments.

We are committed to providing a fair and constructive peer-review process. Please do not hesitate to contact us if there are specific requests from the reviewers that you believe are technically impossible or unlikely to yield a meaningful outcome.

Please use the link below to submit a revised paper:

Link Redacted

Note: This url links to your confidential homepage and associated information about manuscripts you may have submitted or be reviewing for us. If you wish to forward this e-mail to co-authors, please delete this link to your homepage first.

Given the extremely competitive nature of the field, we would urge that you revise and resubmit the manuscript as quickly as possible. Therefore, we would be grateful if you could indicate an estimated timeline by responding to this email.

Looking forward to hearing from you.

Best wishes,

****
Reviewer Expertise:

Referee #1: Coronaviruses, virus entry
Referee #2: Coronaviruses, virus entry
Referee #3: CryoEM and virology

Reviewer Comments:

Reviewer #1 (Remarks to the Author):

Liu and colleagues report that European and Asian hedgehog merbecoviruses (EriCoVs) use hedgehog aminopeptidase N (APN) as their functional receptor, with a narrow APN ortholog tropism restricted to hedgehogs and some felids, and rely on hedgehog TMPRSS2 for spike activation and fusion. The authors used comparative genomics, a 139-species APN ortholog panel, cryo-EM of RBD–APN and antibody–RBD complexes, and pseudovirus/rcVSV entry and spread assays to define the molecular determinants of receptor usage, host range and entry routes. They also generated neutralizing antibodies against both EriCoV RBD and hedgehog APN that block EriCoV entry in APN-expressing human cells and primary hedgehog cells. This integration of genomics, structural virology, receptor biology, and antibody engineering is a clear strength and supports a translational angle for pandemic preparedness. This is an impressive work, adding APN to the repertoire of betacoronavirus receptors, previously governed by DPP4 and ACE2. The authors also demonstrate a novel APN-binding mode distinct from that of 229E. I don't have any major concerns.
Main comments
1. These results are in line with a similar work by Jin et al, available at BioRxiv. This other study should be discussed more precisely, highlighting the common results.
2. The conversion of hAPN into an EriCoV receptor via 5–6 mutations is interesting; adding functional data on how these mutations affect 229E usage of APN would clarify whether gain of EriCoV binding trades off against other APN–CoV interactions. This should be at least further discussed.

Reviewer #2 (Remarks to the Author):

The authors have produced a comprehensive, logical, and easy to follow stream of experiments assessing the receptor binding of EriCoVs.
They identify hedgehog APN (also known as ANPEP) as the receptor primary receptor for the virus. In a reasonably comprehensive analysis they test the ability of other species' APN able to mediate fusion or infection of pseudovirus.

They find trypsin, in the full-lengths version to have signficant impact on fusion and infection. They also do a limited analysis of proteolytic activation, i.e. limited to TMPRSS2 overexpression experiments and broad-range protease or lipopeptide fusion inhibitors. This experiment is the weakest one in the manuscript. Effect of TMPRSS2 is marginal and not brought into context of a pseudovirus (VSV) that normally enters through Clathrin-mediated endocytosis also due to its size and shape. I have suggested further experiments in the respective section and also would urge to tune down the TMPRSS2 wording in the summary section as trypsin also is highly potent to activate this virus.

Like many of these papers, the authors have a very strong focus on human spillover but negate to look at the immediate biology of infetion in the target species and transmission to species that interact closely. I have highlighte this in the section relating to proteolytic activation but also relating to APN species binding. Looking at hedgehog predator species for example would have been interesting. The latter experiments would be interesting to be performed but not exxential. The proteolytic activation experiments, especially when heading the respective section as such, should be added.

One more minor thing I would like to see are western blots on the mutant APN proteins to show not only that the tag can be stained in a cell but that the protein is not degraded. This would be supplementary data and is indicated further down with respect to the relative figures.

Graphical abstract:
I was wondering what made the authors chose to highlight PRCV specifically for the APN binding, whereas this is binding to the Alphacoronavirus Suis species in general as they highlight for all the EriCoVs in general. At least it should be broader than PRCV since PRCV is a deletion mutant of TGEV, which is evolutionary “parental”. If we are going down that path, it should even be CCoV as an evolutionary ancestral strain. However, I think αCoV Suis would be most applicable as the binding modes are

highly similar.

Summary:
As highlighted later in the respective section, I believe TMPRSS2 functionality here is overinterpreted and should be worded more cautiously here. Just because PM fusion is enhanced, doesn't necessarily mean it's biologically relevant. Especially when using pseudovirus systems based on a different virus family as well.

Introduction
Section Line 63fff: Please also highlight what the biology is of EriCoVs in hedgehogs. What is known about their impact in the species itself; what is the infection and transmission route if known?

Line 76: "cross species transmission potential"

Results
Line 182: It would suggest clarifying the experiment for 2a a bit – "supported detectable binding of Fc-tagged RBD protein (Fig. 2a)". Quantification here could have been nice i.e. only positive receptor expressing cells show binding staining but it is pretty black and white so that I don't think this is essential.
Similarly, in the figure legend, I presume binding was also confirmed with immunofluorescence staining? Please add this and also compare with Figure 3 legend and make sure the same experiments have at least similar and independently comprehensible legends. In 3 for example you highlight RBD-Fc is green, not in 1.

Line 183: This is the first introduction of the pseudoviruses in the main body of text. Pseudoviruses can be made on a variety of backgrounds that impact entry pathways. Therefore, please introduce that this is a VSV-based pseudovirus used in these experiments and for all the following experiments. I would urge to add this information to all the figure legends where pseudoviruses are used, i.e. VSV-pseudovirus mentioned at least once in the legend to each figure where the authors used pseudoviruses.

Line 185: Interesting that the authors chose 229E RBD rather than any of the arguably more closely related APN-binding coronaviruses. Maybe highlight that this reciprocal experiment is limited by adding a line; No other APN-binding coronaviruses were tested in this experiment. (Just to remind the reader immediately that there are other APN-binding coronaviruses that may bind to hedgehog APN. In case I forget this later, worth discussing this in the discussion as well that recombination events could be reciprocal as well, i.e. hedgehogs being infected by a virus from a more closely interacting species, for example badgers, natural predators of hedgehogs, who recently were shown to carry an alphacoronavirus; 10.1016/j.meegid.2023.105406).

Line 189: I would suggest that the authors use the word peptidase NOT protease activity. Whilst technically proteolytic, it's a term not quite appropriate in the common use of protease activity.

Lines 192 fff and figure 2 h & i: I like the figures and the results, clearly indicating a possible and likely enteric infection route of this virus (would have been interesting to see whether pepsin has similar activity). Nonetheless, I would just highlight either in the results or the figure legend some notes about cell health in the face of such high levels of trypsin. The black shades in the background show significant clumping up of cells; I am actually surprised that

Ortholog specificity and host tropism determinants
Line 234: suggest adding "APNs from shrews Sorex … "

Line 247: suggest "all tested species of ERiCoVs"

Line 249: suggest "the GER216, FR7137, and PLJ64 strains [or strain spikes] are also capable …"

Figure 3: For experiments related to the chimera figures, 3f, j, and i; I would like the authors to show a western blot with these chimeras in the supplementary data, just to show these proteins do not show signs of misfolding and degradation. Whilst these two species show about 76% sequence identity, some of the areas are significantly different that this must be a concern.
It is a real shame that the authors didn't include biologically important species in this experiment as well, i.e. main predator species of hedgehogs, such as badger species. That would be an important assessment for risk of cross species transmission.

Line 262: highlight which species contains the glycosylation site.

Line 267: I think the statement in 267 is too definite. Given the authors haven't done further experiments with recombinants of F. Cat or P. Par they cannot say this "likely explains their receptor compatibility." I would say "could be an explanation for their receptor compatibility." Furthermore, in relation to figure k: How did the authors decide on critical residues involved in host range determination? I cannot quite follow the thought process here, for example, I kind of understand the first stretch of 6 amino acids, though this has not been experimentally validated, e.g. swapping this section into feline APN may be very insightful? In the latter region, 326 I can again not follow. There is a clear difference in hedgehog but why this may be determining I don't understand. Do the boxes here reflect the alpha2 and beta 16 regions at all? More rationale and explanation in the results section is needed here.

Note on both structural sections; can you ensure it is clear which amino acid residues you are numbering in the different sections? I presume in the "Ortholog specificity and host tropism determinants" it is always the human aa residue number you are highlighting whereas it is or possibly should be the hedgehog, i.e. E. amu residue in the "Structure basis of E.amu APN

recognition by EriCoV" section.

Structure basis of E.amu APN recognition by EriCoV
Pretty straightforward structural analysis chapter. Similar to my previous note on figure 3, it would be good to also see western blots on the mutant protein expression.

For the experiment relating to figure 4h, I refer back to multiple ones of my previous comments; this section remains hard to follow since the explanation and choice of residues related to figure 3k were not made very clear. Some of these residues are now highlighted in figure 4b, which makes the non-verbalised rationale clear now based on structural information. The slight issue here is that in figure 3k, residues are numbered with the human residue numbers whereas now the hedgehog residues are used. It may be good for the sequence alignment in 3k to display both so that the link to figure 4h would be clear. I would also suggest somewhere around line 360 to refer back to figure 3k and you must highlight that these aa changes were based on mutations to the human aa residue in that alignment position. Easiest if referring to figure 3k.

EriCoV spike proteolytic activation
I will repeat myself further down in this section as I have only just added this general note. However, the authors have put a very generic title on this section yet have done a fraction of what they promise. What they have primarily looked at is functional potential of TMPRSS2 in EriCoVs but not broader proteolytic activation studies. If one looks at proteolytic activation in MERS-CoV, the virus is very amenable to a broad range of proteolytic activation (10.1073/pnas.1407087111). For a thorough study, and what is really warranted her, the authors need to look at a broader spectrum of proteolytic activation. They already have all the general assays in place; it would really only take an expansion of figure 5a, or broader fusion assay tests. I think this section is lacking and needs extension beyond TMPRSS2. Furthermore, the reasonably marginal effect that TMPRSS2 produces does not warrant the absolute statement of TMPRSS2 needs in the abstract and needs to be tuned down or extended once further proteolytic activation studies have been performed.

Line 413: The facilitation of S protein activation is not always merely a facilitator for S protein activation but can also be, for many coronaviruses, be a requirement determined by the macroenvironment for tropism of infection and stability. Many of the enteric tropism coronaviruses rely on the stability from a non or not completely proteolytically processed spike protein. Activation by macroenvironment proteases, such as pepsin or trypsin in the small intestine, or microenvironment proteases in the endocytic vesicular system are crucial for successful infection of many coronaviruses. For all intents and purposes, what we know about merbecoviruses is that they are enteric in hedgehogs so this is important to remember when discussing proteolytic processing.

Lines 414ff: This comment links to the previous comment; the authors like to make the link back to another merbecovirus here – understandably. However, "inefficient" particle processing during particle biogenesis is completely normal for some coronaviruses. In particular the ones entering through an oro-fecal route, e.g. PEDV, are not proteolytically processed upon assembly and release. It is only upon trypsin treatment that PEDV for example gets processed (e.g. 10.1128/JVI.00297-14). Just as a cautionary sentence here as the focus seems to be on plasma membrane mediated fusion for this entire section whilst working with a) a pseudovirus only and b) in a very artificial cell and macroenvironment system for this virus.

Line 424: I would reword that to: "functions as an accessory factor only to the APN receptor."

Figure 5 and line 427ff: I understand the general rationale the authors employ here basically using trypsin as a generic proteolytic activation tool to enhance entry of this specific coronavirus. What seems to be neglected in the general context is that trypsin may very well be the true protease that may activate EriCoVs in vivo. They do their best to find alternative proteases but for example, don't show what happens to EriCoV spikes upon protease treatment in figure 5a. To be truly comprehensive looking at what the section title suggests I would urge the authors to perform proteolytic activation assays with common proteases including cathepsins, trypsin, pepsin, plasmin, PC1, Xa, etc. Think about papers like the one by Millet & Whittaker on MERS (10.1073/pnas.1407087111) and 10.1038/s41586-025-09155-z, extended data figure 5.

Please also bare in mind that for some of the enteric coronaviruses trypsin does not merely function as a pre-activator but viruses very much require presence of trypsin during entry (e.g. PEDV, 10.1128/JVI.00297-14). Please caveat this in the biological context also in relation to low level infection that is observed.

Line 432fff: E64d is a cys protease inhibitor and whilst it inhibits cathepsins, it also inhibits a broad array of other cellular proteases. Please clarify this here – e.g. the cys-protease inhibitor E64d, used here as a proxy to assess potential Cathepsin involvement. Similarly, Camostat inhibits a variety of serine proteases, including TMPRSS2, but also trypsin, plasmin, Xa, and others. These are not specific assays and must be caveated as such. If a broader proteolytic activation panel would be performed for 5a, then maybe broader conclusions could be reached here. Camostat is also cell permeable so it cannot be assumed it only acts on surface proteases.
Furthermore, maybe expand that EK1C4 (eventually in line 453) is really a lipopeptide fusion inhibitor that can act on PM and endocytic fusion inhibition.

Line 438: Any rationale why the authors now moved to CaCo-2 cells? Human enteric cell line? (also not a fully differentiated cell line so it can be polymorphic). Also please specify that "can be boosted with trypsin pre-treatment of the pseudovirus" unless in this case it is treatment during infection?

Figure 5f, g and associated results section 436 fff: I do only partially agree with the author's interpretation here. The fact that trypsin treatment boosts infection (significantly) and TMPRSS2 presence at the surface boosts infection slightly – 3-5x – does not support a strong involvement of TMPRSS2. In my opinion, it supports a conclusion that proteolytic activation upon receptor binding may be required for successful infection. A role that can be partially met by an OVEREXPRESSION of TMPRSS2 but may also be met by a macroenvironmental protease in vivo (i.e. trypsin, pepsin, etc). The only thing I conclude from the

experiment in figure 5 is that TMPRSS2 is marginally enhancing infection here but that other proteases, similar to MERS but also similar to other (possibly enteric) coronaviruses, are playing a role as the E64d treatment and camostat treatment show. Namely, in the absence of TMPRSS2 overexpression (figure 5g), infection is far stronger with Camostat treatment than it ever is in the presence of TMPRSS2 with Camostat treatment. This actually indicates that TMPRSS2 may be having negative effects on infection rather than aiding it. And that surface fusion is possibly not a successful infection route for the pseudovirus. But please bare in mind this is a system based on VSV, which generally goes through clathrin-mediated endocytic uptake and this may not be as relevant for the actual EriCoV.

Neutralizing antibodies against EriCoV
I don't have much to say on this experiment. Monoclonal antibody therapies have shown limited long-term efficacy in preventing spread but can aid individual patients and could possibly help in a small localised outbreak scenario.

Discussion
The discussion section is short and I would like to see a bit more discussion of the risk of cross-species spillover. Discussion of the binding pocket and what the risks are of spillover in marginally susceptible species, such as felines and rats. As highlighted earlier in the respective section, it is a shame that the authors did not assess the susceptibility of natural predators of hedgehogs, such as badgers in the biological context.

Similarly, once proteolytic activation has been studied a bit more broadly, I would like to see some discussion on the route of transmission and infection (based on the biological

Reviewer #3 (Remarks to the Author):

Liu et al. demonstrate that European and Asian hedgehog coronaviruses (EriCoVs) utilize hedgehog aminopeptidase N (APN) as a physiological receptor. Using a broad panel of EriCoV spike proteins and APN-expressing cells, combined with cryo-EM structural analysis, the authors provide a comprehensive study. These analyses are excellent and impressive, offering clear evidence for receptor characterization. However, I have the following comments to address.

Major Comments
1. The authors found that R. norvegicus (R.nor) APN did not support RU15-1 or MO1 binding (via Fc-fusion RBD proteins) and only exhibited detectable binding with the FR7137 RBD. However, the constructs for the Fc-fusion proteins appear inconsistent across species; some include the entire S1 domain, while others include only the RBD. The S1 domain alone can be particularly unstable, and the exposure of hydrophobic interfaces (originally the S2 interface) might facilitate non-specific binding. The authors should clearly state these differences in the expressed domains and discuss how this species specificity relates to amino acid substitutions in both the APNs and the RBD/S1 domains, based on their cryo-EM structures.
2. Details regarding the recombinant Spike constructs used for cryo-EM are missing. The authors should clarify whether they introduced stabilizing mutations, such as prolines. If such mutations were used, they might account for the dominance of the "closed" conformation.
3. The PPI2 prediction is neither cited nor fully described. It is unclear which tool was used and whether the prediction is reliable.

Minor Comments
1. A figure showing an amino acid sequence comparison of APNs and EriCoVs, including a binding counter/matrix, would greatly assist the reader's understanding.
2. Lines 255-256: The sentence "Substituting domain 2 of E. amu APN with its human counterpart abolished binding, while substituting E. amu Domain 2 with human APN counterpart restored binding..." is confusing. Please clarify the direction of these substitutions.
3. The inconsistent use of "hAPN/human APN" and "hTMPRSS2/human TMPRSS2" may be confusing. Since proteins from various species are compared throughout the manuscript, I suggest unifying the terminology to "human APN" and "human TMPRSS2" for clarity.
4. Lines 310-311: To avoid confusion with the "closed" state of the Spike protein (even though only the RBD was used), the phrase "...ensuring that all E. amu APN particles adopted a 'closed' conformation" should perhaps be revised to "...ensuring that all E. amu APN molecules in the particles adopted a 'closed' conformation."
5. Figure 3g: Two shifted peaks are observed for WT E. amu APN with MO1-RBD, whereas only one is observed with RU15.1. The authors should provide a comment on this observation.
6. Figure 4 (panels a, b, or c?), its legend, and the related text (lines 319-330): The authors should indicate or mention K310, which corresponds to the N-glycosylation site in human APN.
7. Figure 4f: For a clearer comparison of the receptor-binding interfaces, the RBDs in all complexes should be displayed at the same angle.

****

Version 2:

Reviewer comments:

Reviewer #1

(Remarks to the Author)
the authors have addressed my concerns

Reviewer #2

(Remarks to the Author)
Dear authors,

I can only give back my sincere thanks for what is a very thorough follow-up and response to my feedback. This was already a very good manuscript and as a I highlighted, comprehensive and easy to follow; it is now even more so.

I think this manuscript can now stand very proud as a great example of one health research of infectious agents where often we don't have a full virus but equally conducting thorough and safe in vitro work are vital for risk assessment and biological understanding.

I strongly recommend this manuscript for acceptance.

Reviewer #3

(Remarks to the Author)
I would like to thank the authors for their detailed responses to my previous comments. I apologize for introducing these structure-related concerns at this stage, as they should have been raised during the initial review. However, upon further inspection of the deposited data, I believe the following points require clarification or correction to ensure the highest quality of the manuscript:

Based on the PDB validation reports, I have concerns regarding the following points:
1) PDB IDs: 9XW6 and 9XWA (Validation reports of PDB)
While the interface with the RBD is at a reasonable resolution and the model building appears generally plausible, the modeling of the RBD itself seems somewhat overstrained or physically unnatural (as reflected in the Validation Reports, pp. 39–40 (9XW6) and pp. 54–55 (9XWA), respectively).
2) PDB ID: 9XWM
There are several critical indicators of potential issues with data processing and model quality (Extended Data Fig. 12a and Validation report of PDB):
• Unnatural FSC Curve: The FSC curve exhibits an unnatural profile, dropping abruptly and unusually smoothly from a flat plateau.
• High Clashscore: The clashscore is exceptionally high (19), which raises concerns about the stereochemical quality of the model.
• Signs of Overfitting: The presence of radial map artifacts suggests potential overfitting, which is further supported by the notably low Q-scores in the outer regions of the molecule.
The authors might had better check DAQ sore of Prof. Kihara's laboratory of Purdue University (https://daqdb.kiharalab.org/) to evaluate the structures and should address these concerns or correct the models accordingly.

Extended Data Fig. 7
Please use the yellow "dotted" lines for interactions.

Decision Letter:

26th May 2026

*Please ensure you delete the link to your author homepage in this e-mail if you wish to forward it to your co-authors.

Dear Professor Yan,

Thank you for your patience while your manuscript "Convergent utilization of APN receptor by hedgehog merbecoviruses" was under peer-review at Nature Microbiology. It has now been seen by 3 referees, whose expertise and comments you will find at the end of this email. You will see from their comments below that while they find your work of interest, some important points are raised. We are very interested in the possibility of publishing your study in Nature Microbiology, but would like to consider your response to these concerns in the form of a revised manuscript before we make a final decision on publication.

In particular, you will see that referee #1 raises concerns related to your structure. The rest referees' reports are clear and the remaining issues should be straightforward to address.

We are committed to providing a fair and constructive peer-review process. Do not hesitate to contact us if there are specific requests from the reviewers that you believe are technically impossible or unlikely to yield a meaningful outcome.

If you have not done so already please begin to revise your manuscript so that it conforms to our Article format instructions at http://www.nature.com/nmicrobiol/info/final-submission/

The usual length limit for a Nature Microbiology Article is six display items (figures or tables) and 3,000 words. We have some flexibility, and can allow a revised manuscript at 3,500 words, but please consider this a firm upper limit. There is a trade-off of ~250 words per display item, so if you need more space, you could move a Figure or Table to Supplementary Information.

Some reduction could be achieved by focusing any introductory material and moving it to the start of your opening 'bold' paragraph, whose function is to outline the background to your work, describe in a sentence your new observations, and explain your main conclusions. The discussion should also be limited. Methods should be described in a separate section following the discussion, we do not place a word limit on Methods.

Nature Microbiology titles should give a sense of the main new findings of a manuscript, and should not contain punctuation. Please keep in mind that we strongly discourage active verbs in titles, and that they should ideally fit within 90 characters each (including spaces).

We strongly support public availability of data. Please place the data used in your paper into a public data repository, if one exists, or alternatively, present the data as Source Data or Supplementary Information. If data can only be shared on request, please explain why in your Data Availability Statement, and also in the correspondence with your editor. For some data types, deposition in a public repository is mandatory - more information on our data deposition policies and available repositories can be found at https://www.nature.com/nature-research/editorial-policies/reporting-standards#availability-of-data.

Please include a data availability statement as a separate section after Methods but before references, under the heading "Data Availability". This section should inform readers about the availability of the data used to support the conclusions of your study. This information includes accession codes to public repositories (data banks for protein, DNA or RNA sequences, microarray, proteomics data etc…), references to source data published alongside the paper, unique identifiers such as URLs to data repository entries, or data set DOIs, and any other statement about data availability. At a minimum, you should include the following statement: "The data that support the findings of this study are available from the corresponding author upon request", mentioning any restrictions on availability. If DOIs are provided, we also strongly encourage including these in the Reference list (authors, title, publisher (repository name), identifier, year). For more guidance on how to write this section please see: http://www.nature.com/authors/policies/data/data-availability-statements-data-citations.pdf

To improve the accessibility of your paper to readers from other research areas, please pay particular attention to the wording of the paper's opening bold paragraph, which serves both as an introduction and as a brief, non-technical summary in about 150 words. If, however, you require one or two extra sentences to explain your work clearly, please include them even if the paragraph is over-length as a result. The opening paragraph should not contain references. Because scientists from other sub-disciplines will be interested in your results and their implications, it is important to explain essential but specialised terms concisely. We suggest you show your summary paragraph to colleagues in other fields to uncover any problematic concepts.

If your paper is accepted for publication, we will edit your display items electronically so they conform to our house style and will reproduce clearly in print. If necessary, we will re-size figures to fit single or double column width. If your figures contain several parts, the parts should form a neat rectangle when assembled. Choosing the right electronic format at this stage will speed up the processing of your paper and give the best possible results in print. We would like the figures to be supplied as vector files - EPS, PDF, AI or postscript (PS) file formats (not raster or bitmap files), preferably generated with vector-graphics software (Adobe Illustrator for example). Please try to ensure that all figures are non-flattened and fully editable. All images should be at least 300 dpi resolution (when figures are scaled to approximately the size that they are to be printed at) and in RGB colour format. Please do not submit Jpeg or flattened TIFF files. Please see also 'Guidelines for Electronic Submission of Figures' at the end of this letter for further detail.

Figure legends must provide a brief description of the figure and the symbols used, within 350 words, including definitions of any error bars employed in the figures.

When submitting the revised version of your manuscript, please pay close attention to our href="https://www.nature.com/nature-research/editorial-policies/image-integrity">Digital Image Integrity Guidelines. and to the following points below:

-- that unprocessed scans are clearly labelled and match the gels and western blots presented in figures.
-- that control panels for gels and western blots are appropriately described as loading on sample processing controls
-- all images in the paper are checked for duplication of panels and for splicing of gel lanes.

EXTENDED DATA FIGURES

When re-submitting your manuscript, please ensure that any supplementary figures and tables that are crucial to the manuscript's conclusions are converted into Extended Data figures and tables to increase visibility of these data. Extended Data figures and tables are online-only (present in the online PDF and full-text HTML versions of the paper), peer-reviewed display items that provide essential background to the article but are not included in the main article due to space constraints. A maximum of ten Extended Data display items (figures and tables) is permitted.

Finally, please ensure that you retain unprocessed data and metadata files after publication, ideally archiving data in perpetuity, as these may be requested during the peer review and production process or after publication if any issues arise.

Please include a statement before the acknowledgements naming the author to whom correspondence and requests for materials should be addressed.

Finally, we require authors to include a statement of their individual contributions to the paper -- such as experimental work, project planning, data analysis, etc. -- immediately after the acknowledgements. The statement should be short, and refer to authors by their initials. For details please see the Authorship section of our joint Editorial policies at http://www.nature.com/authors/editorial_policies/authorship.html

When revising your paper:

* include a point-by-point response to any editorial suggestions and to our referees. Please include your response to the editorial suggestions in your cover letter, and please upload your response to the referees as a separate document.

* ensure it complies with our format requirements for Letters as set out in our guide to authors at www.nature.com/nmicrobiol/info/gta/

* state in a cover note the length of the text, methods and legends; the number of references; number and estimated final size of figures and tables

* resubmit electronically if possible using the link below to access your home page:

Link Redacted

*This url links to your confidential homepage and associated information about manuscripts you may have submitted or be reviewing for us. If you wish to forward this e-mail to co-authors, please delete this link to your homepage first.

Please ensure that all correspondence is marked with your Nature Microbiology reference number in the subject line.

Nature Microbiology is committed to improving transparency in authorship. As part of our efforts in this direction, we are now requesting that all authors identified as ‘corresponding author’ on published papers create and link their Open Researcher and Contributor Identifier (ORCID) with their account on the Manuscript Tracking System (MTS), prior to acceptance. This applies to primary research papers only. ORCID helps the scientific community achieve unambiguous attribution of all scholarly contributions. You can create and link your ORCID from the home page of the MTS by clicking on ‘Modify my Springer Nature account’. For more information please visit please visit www.springernature.com/orcid.

We hope to receive your revised paper within three weeks. If you cannot send it within this time, please let us know.

We look forward to hearing from you soon.

Yours sincerely,

****

Reviewer Expertise:

Referee #1: virus entry, virology
Referee #2: coronaviruses
Referee #3: structural biology

Reviewers Comments:

Reviewer #1 (Remarks to the Author):

the authors have addressed my concerns

Reviewer #2 (Remarks to the Author):

Dear authors,

I can only give back my sincere thanks for what is a very thorough follow-up and response to my feedback. This was already a very good manuscript and as a I highlighted, comprehensive and easy to follow; it is now even more so.

I think this manuscript can now stand very proud as a great example of one health research of infectious agents where often we don't have a full virus but equally conducting thorough and safe in vitro work are vital for risk assessment and biological understanding.

I strongly recommend this manuscript for acceptance.

Reviewer #3 (Remarks to the Author):

I would like to thank the authors for their detailed responses to my previous comments. I apologize for introducing these structure-related concerns at this stage, as they should have been raised during the initial review. However, upon further inspection of the deposited data, I believe the following points require clarification or correction to ensure the highest quality of the manuscript:

Based on the PDB validation reports, I have concerns regarding the following points:
1) PDB IDs: 9XW6 and 9XWA (Validation reports of PDB)
While the interface with the RBD is at a reasonable resolution and the model building appears generally plausible, the modeling of the RBD itself seems somewhat overstrained or physically unnatural (as reflected in the Validation Reports, pp. 39–40 (9XW6) and pp. 54–55 (9XWA), respectively).
2) PDB ID: 9XWM
There are several critical indicators of potential issues with data processing and model quality (Extended Data Fig. 12a and Validation report of PDB):
• Unnatural FSC Curve: The FSC curve exhibits an unnatural profile, dropping abruptly and unusually smoothly from a flat plateau.
• High Clashscore: The clashscore is exceptionally high (19), which raises concerns about the stereochemical quality of the model.
• Signs of Overfitting: The presence of radial map artifacts suggests potential overfitting, which is further supported by the notably low Q-scores in the outer regions of the molecule.
The authors might had better check DAQ sore of Prof. Kihara's laboratory of Purdue University (https://daqdb.kiharalab.org/) to evaluate the structures and should address these concerns or correct the models accordingly.

Extended Data Fig. 7
Please use the yellow "dotted" lines for interactions.

****

Version 3:

Reviewer comments:

Reviewer #3

(Remarks to the Author)
I would like to thank the authors for their detailed response and for providing the updated information and models regarding their cryo-EM analysis.

General Comments: The revised models and statistics look improved; the global FSC resolution has been adjusted to a more realistic value (from 2.0 Å to over 3.x Å), and the density maps for the RBD-receptor binding interface—the primary focus of this study—look reasonable.

However, there are still several noticeable flaws in the detailed model building. At the current resolution, discussing specific side-chain orientations and detailed interactions is at the baseline threshold of feasibility. Since it is difficult to verify every single detail of the coordinates, the authors must thoroughly re-examine and further refine the models under their own responsibility.

The following specific points regarding model quality and map processing should be addressed:

Glycan and Model Quality: There are prominent discrepancies in the model building (please see the examples, the attached pdf file of maps). For example, specifically, there are regions where clear glycan densities are visible but no models have been built. Conversely, in other areas, models have been placed where no corresponding density exists in the map. The authors need to carefully inspect the entire structure and correct these modeling errors.

Map Sharpening and B-factors: All submitted maps appear to be sharpened, and they show signs of over-sharpening (e.g., discontinuous/broken densities). The authors should inspect the unsharpened maps for verification. Furthermore, rather than relying solely on the default sharpened maps automatically generated by the CryoSPARC refinement job, it is highly recommended to run independent sharpening jobs. The authors should test multiple B-factor values to find the optimal sharpening level that balances detail and continuity.

Mask Soft Padding Width: The mask soft padding used in the analysis should be re-evaluated. According to the official CryoSPARC guide, a soft padding width of 5×(current resolution/pixel size) is generally recommended. While this width may be narrowed for smaller targets, the authors should first test a width of approximately 23 Å derived from this standard formula to

ensure appropriate masking and prevent potential artifacts. (Reference: https://guide.cryosparc.com/processing-data/tutorials-and-case-studies/mask-selection-and-generation-in-ucsf-chimera)

Decision Letter:

1st July 2026

*Please ensure you delete the link to your author homepage in this e-mail if you wish to forward it to your co-authors.

Dear Huan,

Thank you for your patience while your manuscript "Convergent utilization of APN receptor by hedgehog merbecoviruses" was under peer-review at Nature Microbiology. It has now been seen by 1 referees, whose expertise and comments you will find at the end of this email. You will see from their comments below that while they find your work of interest, some important points are raised. We are very interested in the possibility of publishing your study in Nature Microbiology, but would like to consider your response to these concerns in the form of a revised manuscript before we make a final decision on publication.

In particular, you will see that this referee requests to further improve the cryoEM models. The examples mentioned by this referee can be found in the attachment. In addition, we would kindly ask you to seperately upload your extended data files (figures and table) when resubmitting your revised manuscript.

We are committed to providing a fair and constructive peer-review process. Do not hesitate to contact us if there are specific requests from the reviewers that you believe are technically impossible or unlikely to yield a meaningful outcome.

If you have not done so already please begin to revise your manuscript so that it conforms to our Article format instructions at http://www.nature.com/nmicrobiol/info/final-submission/

The usual length limit for a Nature Microbiology Article is six display items (figures or tables) and 3,000 words. We have some flexibility, and can allow a revised manuscript at 3,500 words, but please consider this a firm upper limit. There is a trade-off of ~250 words per display item, so if you need more space, you could move a Figure or Table to Supplementary Information.

Some reduction could be achieved by focusing any introductory material and moving it to the start of your opening ‘bold’ paragraph, whose function is to outline the background to your work, describe in a sentence your new observations, and explain your main conclusions. The discussion should also be limited. Methods should be described in a separate section following the discussion, we do not place a word limit on Methods.

Nature Microbiology titles should give a sense of the main new findings of a manuscript, and should not contain punctuation. Please keep in mind that we strongly discourage active verbs in titles, and that they should ideally fit within 90 characters each (including spaces).

We strongly support public availability of data. Please place the data used in your paper into a public data repository, if one exists, or alternatively, present the data as Source Data or Supplementary Information. If data can only be shared on request, please explain why in your Data Availability Statement, and also in the correspondence with your editor. For some data types, deposition in a public repository is mandatory - more information on our data deposition policies and available repositories can be found at https://www.nature.com/nature-research/editorial-policies/reporting-standards#availability-of-data.

Please include a data availability statement as a separate section after Methods but before references, under the heading "Data Availability”. This section should inform readers about the availability of the data used to support the conclusions of your study. This information includes accession codes to public repositories (data banks for protein, DNA or RNA sequences, microarray, proteomics data etc…), references to source data published alongside the paper, unique identifiers such as URLs to data repository entries, or data set DOIs, and any other statement about data availability. At a minimum, you should include the following statement: “The data that support the findings of this study are available from the corresponding author upon request”, mentioning any restrictions on availability. If DOIs are provided, we also strongly encourage including these in the Reference list (authors, title, publisher (repository name), identifier, year). For more guidance on how to write this section please see: http://www.nature.com/authors/policies/data/data-availability-statements-data-citations.pdf

To improve the accessibility of your paper to readers from other research areas, please pay particular attention to the wording of the paper’s opening bold paragraph, which serves both as an introduction and as a brief, non-technical summary in about 150 words. If, however, you require one or two extra sentences to explain your work clearly, please include them even if the paragraph is over-length as a result. The opening paragraph should not contain references. Because scientists from other sub-disciplines will be interested in your results and their implications, it is important to explain essential but specialised terms concisely. We suggest you show your summary paragraph to colleagues in other fields to uncover any problematic concepts.

If your paper is accepted for publication, we will edit your display items electronically so they conform to our house style and will reproduce clearly in print. If necessary, we will re-size figures to fit single or double column width. If your figures contain several parts, the parts should form a neat rectangle when assembled. Choosing the right electronic format at this stage will speed up the processing of your paper and give the best possible results in print. We would like the figures to be supplied as vector files - EPS, PDF, AI or postscript (PS) file formats (not raster or bitmap files), preferably generated with vector-graphics software

(Adobe Illustrator for example). Please try to ensure that all figures are non-flattened and fully editable. All images should be at least 300 dpi resolution (when figures are scaled to approximately the size that they are to be printed at) and in RGB colour format. Please do not submit Jpeg or flattened TIFF files. Please see also 'Guidelines for Electronic Submission of Figures' at the end of this letter for further detail.

Figure legends must provide a brief description of the figure and the symbols used, within 350 words, including definitions of any error bars employed in the figures.

When submitting the revised version of your manuscript, please pay close attention to our href="https://www.nature.com/nature-research/editorial-policies/image-integrity">Digital Image Integrity Guidelines. and to the following points below:

-- that unprocessed scans are clearly labelled and match the gels and western blots presented in figures.
-- that control panels for gels and western blots are appropriately described as loading on sample processing controls
-- all images in the paper are checked for duplication of panels and for splicing of gel lanes.

EXTENDED DATA FIGURES

When re-submitting your manuscript, please ensure that any supplementary figures and tables that are crucial to the manuscript's conclusions are converted into Extended Data figures and tables to increase visibility of these data. Extended Data figures and tables are online-only (present in the online PDF and full-text HTML versions of the paper), peer-reviewed display items that provide essential background to the article but are not included in the main article due to space constraints. A maximum of ten Extended Data display items (figures and tables) is permitted.

Finally, please ensure that you retain unprocessed data and metadata files after publication, ideally archiving data in perpetuity, as these may be requested during the peer review and production process or after publication if any issues arise.

Please include a statement before the acknowledgements naming the author to whom correspondence and requests for materials should be addressed.

Finally, we require authors to include a statement of their individual contributions to the paper -- such as experimental work, project planning, data analysis, etc. -- immediately after the acknowledgements. The statement should be short, and refer to authors by their initials. For details please see the Authorship section of our joint Editorial policies at http://www.nature.com/authors/editorial_policies/authorship.html

When revising your paper:

* include a point-by-point response to any editorial suggestions and to our referees. Please include your response to the editorial suggestions in your cover letter, and please upload your response to the referees as a separate document.

* ensure it complies with our format requirements for Letters as set out in our guide to authors at www.nature.com/nmicrobiol/info/gta/

* state in a cover note the length of the text, methods and legends; the number of references; number and estimated final size of figures and tables

* resubmit electronically if possible using the link below to access your home page:

Link Redacted

*This url links to your confidential homepage and associated information about manuscripts you may have submitted or be reviewing for us. If you wish to forward this e-mail to co-authors, please delete this link to your homepage first.

Please ensure that all correspondence is marked with your Nature Microbiology reference number in the subject line.

Nature Microbiology is committed to improving transparency in authorship. As part of our efforts in this direction, we are now requesting that all authors identified as 'corresponding author' on published papers create and link their Open Researcher and Contributor Identifier (ORCID) with their account on the Manuscript Tracking System (MTS), prior to acceptance. This applies to primary research papers only. ORCID helps the scientific community achieve unambiguous attribution of all scholarly contributions. You can create and link your ORCID from the home page of the MTS by clicking on 'Modify my Springer Nature account'. For more information please visit please visit www.springernature.com/orcid.

We hope to receive your revised paper within one weeks. If you cannot send it within this time, please let us know.

We look forward to hearing from you soon.

Yours sincerely,

****

Reviewer Expertise:

Referee #3: cryoEM

Reviewers Comments:

Reviewer #3 (Remarks to the Author):

I would like to thank the authors for their detailed response and for providing the updated information and models regarding their cryo-EM analysis.

General Comments: The revised models and statistics look improved; the global FSC resolution has been adjusted to a more realistic value (from 2.0 Å to over 3.x Å), and the density maps for the RBD-receptor binding interface—the primary focus of this study—look reasonable.

However, there are still several noticeable flaws in the detailed model building. At the current resolution, discussing specific side-chain orientations and detailed interactions is at the baseline threshold of feasibility. Since it is difficult to verify every single detail of the coordinates, the authors must thoroughly re-examine and further refine the models under their own responsibility.

The following specific points regarding model quality and map processing should be addressed:

Glycan and Model Quality: There are prominent discrepancies in the model building (please see the examples, the attached pdf file of maps). For example, specifically, there are regions where clear glycan densities are visible but no models have been built. Conversely, in other areas, models have been placed where no corresponding density exists in the map. The authors need to carefully inspect the entire structure and correct these modeling errors.

Map Sharpening and B-factors: All submitted maps appear to be sharpened, and they show signs of over-sharpening (e.g., discontinuous/broken densities). The authors should inspect the unsharpened maps for verification. Furthermore, rather than relying solely on the default sharpened maps automatically generated by the CryoSPARC refinement job, it is highly recommended to run independent sharpening jobs. The authors should test multiple B-factor values to find the optimal sharpening level that balances detail and continuity.

Mask Soft Padding Width: The mask soft padding used in the analysis should be re-evaluated. According to the official CryoSPARC guide, a soft padding width of 5×(current resolution/pixel size) is generally recommended. While this width may be narrowed for smaller targets, the authors should first test a width of approximately 23 Å derived from this standard formula to ensure appropriate masking and prevent potential artifacts. (Reference: https://guide.cryosparc.com/processing-data/tutorials-and-case-studies/mask-selection-and-generation-in-ucsf-chimera)

****

Version 4:

Reviewer comments:

Reviewer #3

(Remarks to the Author)
Thank you very much for your hard work and detailed response to address all the comments. I highly appreciate your thorough efforts in improving the manuscript and the models.

Since all the points have been successfully addressed, please make sure to deposit and update the refined models in the PDB accordingly.

Decision Letter:

Our ref: NMICROBIOL-26010366D

13th July 2026

Dear Dr. Yan,

Thank you for submitting your revised manuscript "Convergent utilization of APN receptor by hedgehog merbecoviruses" (NMICROBIOL-26010366D). It has now been seen by the original referees and their comments are below. The reviewers find that the paper has improved in revision, and therefore we'll be happy in principle to publish it in Nature Microbiology, pending minor revisions to satisfy the referees' final requests and to comply with our editorial and formatting guidelines.

If the current version of your manuscript is in a PDF format, please email us a copy of the file in an editable format (Microsoft Word or LaTex)-- we can not proceed with PDFs at this stage.

We are now performing detailed checks on your paper and will send you a checklist detailing our editorial and formatting requirements in a couple of weeks. Please do not upload the final materials and make any revisions until you receive this additional information from us.

Thank you again for your interest in Nature Microbiology Please do not hesitate to contact me if you have any questions.

Sincerely,

Reviewer #3 (Remarks to the Author):

Thank you very much for your hard work and detailed response to address all the comments. I highly appreciate your thorough efforts in improving the manuscript and the models.

Since all the points have been successfully addressed, please make sure to deposit and update the refined models in the PDB accordingly.

INTERNAL USE ONLY AIP modules required: ["Life Science"]

Version 5:

Decision Letter:

7th August 2026

Dear Huan,

I am delighted to accept your Article "Convergent use of Aminopeptidase N receptor by hedgehog merbecoviruses" for publication in Nature Microbiology. Thank you for having chosen to submit your work to us and many congratulations.

Over the next few weeks, your paper will be copyedited to ensure that it conforms to Nature Microbiology style. We look particularly carefully at the titles of all papers to ensure that they are relatively brief and understandable.

Once your paper is typeset, you will receive an email with a link to choose the appropriate publishing options for your paper and our Author Services team will be in touch regarding any additional information that may be required. Once your paper has been scheduled for online publication, the Nature press office will be in touch to confirm the details.

You may wish to make your media relations office aware of your accepted publication, in case they consider it appropriate to organize some internal or external publicity. Once your paper has been scheduled you will receive an email confirming the publication details. This is normally 3-4 working days in advance of publication. If you need additional notice of the date and time of publication, please let the production team know when you receive the proof of your article to ensure there is sufficient time to coordinate. Further information on our embargo policies can be found here:
https://www.nature.com/authors/policies/embargo.html

After the grant of rights is completed, you will receive a link to your electronic proof via email with a request to make any corrections within 48 hours. If, when you receive your proof, you cannot meet this deadline, please inform us at rjsproduction@springernature.com immediately. You will not receive your proofs until the publishing agreement has been received through our system

Due to the importance of these deadlines, we ask you please us know now whether you will be difficult to contact over the next month. If this is the case, we ask you provide us with the contact information (email, phone and fax) of someone who will be able to check the proofs on your behalf, and who will be available to address any last-minute problems.

Acceptance of your manuscript is conditional on all authors' agreement with our publication policies (see https://www.nature.com/nmicrobiol/editorial-policies). In particular your manuscript must not be published elsewhere.

Authors may need to take specific actions to achieve compliance with funder and institutional open access mandates. If

your research is supported by a funder that requires immediate open access (e.g. according to Plan S principles or the NIH public access policy) then you should select the gold OA route, and we will direct you to the compliant route where possible. Because authors warrant under our subscription licensing terms that they haven't committed to licensing any version of their article under a licence inconsistent with the terms of our agreement – including the applicable embargo period – publication under the subscription model isn't suitable for authors whose funders require no embargo.

If you have any questions about our publishing options, costs, Open Access requirements, or our legal forms, please contact ASJournals@springernature.com

An online order form for reprints of your paper is available at https://www.nature.com/reprints/author-reprints.html. All co-authors, authors' institutions and authors' funding agencies can order reprints using the form appropriate to their geographical region.

We welcome the submission of potential cover material (including a short caption of around 40 words) related to your manuscript; suggestions should be sent to Nature Microbiology as electronic files (the image should be 300 dpi at 210 x 297 mm in either TIFF or JPEG format). Please note that such pictures should be selected more for their aesthetic appeal than for their scientific content, and that colour images work better than black and white or grayscale images. Please do not try to design a cover with the Nature Microbiology logo etc., and please do not submit composites of images related to your work. I am sure you will understand that we cannot make any promise as to whether any of your suggestions might be selected for the cover of the journal.

You can now use a single sign-on for all your accounts, view the status of all your manuscript submissions and reviews, access usage statistics for your published articles and download a record of your refereeing activity for the Nature journals.

To assist our authors in disseminating their research to the broader community, our SharedIt initiative provides you with a unique shareable link that will allow anyone (with or without a subscription) to read the published article. Recipients of the link with a subscription will also be able to download and print the PDF.

As soon as your article is published, you will receive an automated email with your shareable link.

Congrats again to you and your co-authors! I am looking forward to seeing your paper published.

With kind regards,

P.S. Click on the following link if you would like to recommend Nature Microbiology to your librarian http://www.nature.com/subscriptions/recommend.html#forms

** Visit the Springer Nature Editorial and Publishing website at www.springernature.com/editorial-and-publishing-jobs for more information about our career opportunities. If you have any questions please click here.**

Open Access This Peer Review File is licensed under a Creative Commons Attribution 4.0 International License, which permits use, sharing, adaptation, distribution and reproduction in any medium or format, as long as you give appropriate credit to the original author(s) and the source, provide a link to the Creative Commons license, and indicate if changes were made.
In cases where reviewers are anonymous, credit should be given to 'Anonymous Referee' and the source.
The images or other third party material in this Peer Review File are included in the article's Creative Commons license, unless indicated otherwise in a credit line to the material. If material is not included in the article's Creative Commons license and your intended use is not permitted by statutory regulation or exceeds the permitted use, you will need to obtain permission directly from the copyright holder.
To view a copy of this license, visit https://creativecommons.org/licenses/by/4.0/

Point-by-Point Response letter for Nature Microbiology

Manuscript title: Convergent utilization of APN receptor by hedgehog merbecoviruses

Corresponding authors: **Huan Yan**, Xiangxi Wang, Yunlong Cao, Linlin Bao

Manuscript ID: NMICROBIOL-26010366B

Dear reviewers:

We thank the reviewers for their careful evaluation of our work and for their constructive comments. We have addressed all the points raised, which have helped us further strengthen the manuscript.

Below, we provide a detailed point-by-point response (**blue**) to each comment (**black**) and indicate the corresponding changes (**red**) in the revised manuscript. Besides, we also uploaded a tracked changes version of the revised manuscript with changes and additions marked in **red**.

Thanks again for their time and consideration.

Yours sincerely,

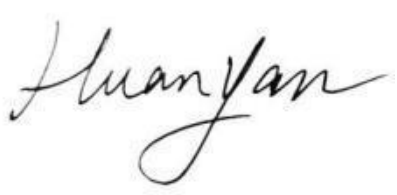

Huan Yan

On behalf of all co-authors

Reviewers' Comments:

Reviewer #1 (Remarks to the Author):

Liu and colleagues report that European and Asian hedgehog merbecoviruses (EriCoVs) use hedgehog aminopeptidase N (APN) as their functional receptor, with a narrow APN ortholog tropism restricted to hedgehogs and some felids, and rely on hedgehog TMPRSS2 for spike activation and fusion. The authors used comparative genomics, a 139-species APN ortholog panel, cryo-EM of RBD–APN and antibody–RBD complexes, and pseudovirus/rcVSV entry and spread assays to define the molecular determinants of receptor usage, host range and entry routes. They also generated neutralizing antibodies against both EriCoV RBD and hedgehog APN that block EriCoV entry in APN-expressing human cells and primary hedgehog cells. This integration of genomics, structural virology, receptor biology, and antibody engineering is a clear strength and supports a translational angle for pandemic preparedness. This is an **impressive** work, adding APN to the repertoire of betacoronavirus receptors, previously governed by DPP4 and ACE2. The authors also demonstrate a novel APN-binding mode distinct from that of 229E. **I don't have any major concerns.**

Response: We sincerely thank the reviewer for the positive assessment of our work. We are particularly grateful for the recognition of the novelty and significance of this finding. We are pleased that the reviewer have no major concerns about this study and considers this study a valuable contribution to the field.

Main comments

1. These results are in line with a similar work by Jin et al, available at BioRxiv. This other study should be discussed more precisely, highlighting the common results.

Response: We thank the reviewer for mentioning the relevance of the recent study by Jin *et al*. We were aware of this parallel study and agree that a more explicit comparison is important for clarity.

In the revised manuscript, we have expanded the Discussion to more thoroughly compare the two studies, highlighting convergent findings, including the identification of APN as a functional receptor for EriCoVs, the overall restricted host range, and the new binding mode. In addition, we also clarified the complementary aspects between the two studies. While both studies identify APN usage, our work evaluated a border panel of species, defines residue-level host range determinants, systematically examines protease usage, and characterizes neutralizing antibodies.

Updated Discussion

Page 27, lines 695–698:

This finding is consistent with a recent parallel study by Jin et al., which independently also identified APN as an entry receptor for EriCoVs and reported a similarly restricted host range. Together, the two studies provide convergent evidence that EriCoVs have evolved a distinct

APN-binding strategy.

Lines 706–709:

At the same time, our study extends these observations by defining residue-level host range determinants, exploring protease requirements for entry, and characterizing neutralizing antibodies, thereby providing a complementary perspective to the work by Jin et al.

Lines 726–728:

Consistent with the study by Jin et al, Our results show that EriCoVs are highly adapted to hedgehog APN orthologs, only a limited number of orthologs—most notably from felids and, to a lesser extent, rodents—support suboptimal receptor function.

2. The conversion of hAPN into an EriCoV receptor via 5–6 mutations is interesting; adding functional data on how these mutations affect 229E usage of APN would clarify whether gain of EriCoV binding trades off against other APN–CoV interactions. This should be at least further discussed.

Response: We thank the reviewer for this insightful suggestion. To address this point, we have experimentally evaluated the impact of the identified mutations on receptor usage of not just 229E but also several other APN-using coronaviruses, including TGEV, CCoV-HuPn-2018, FCoV-23, and PDCoV.

In line with the prior study (10.1016/j.cell.2022.05.019.), our functional results show WT human APN can be utilized by 229E, PDCoV, but not TGEV, CCoV or FCoV-23, while hedgehog APN is specifically recognized by EriCoVs. We found that the introduced 5 or 6 mutations do not significantly impair 229E entry and to a extent enhance PDCoV entry (cf. **Reviewer Fig. R1** below). These findings suggest that acquisition of EriCoV receptor functionality by human APN can occur without a substantial functional trade-off with other APN-dependent coronaviruses. This observation is consistent with the distinct binding interface observed in our structural analyses, indicating that APN can accommodate multiple coronavirus binding modes with limited cross interference.

These findings provide additional insight into the strain specificity of APN usage and have now been incorporated into the revised manuscript (**Extended Data Fig. 9f**).

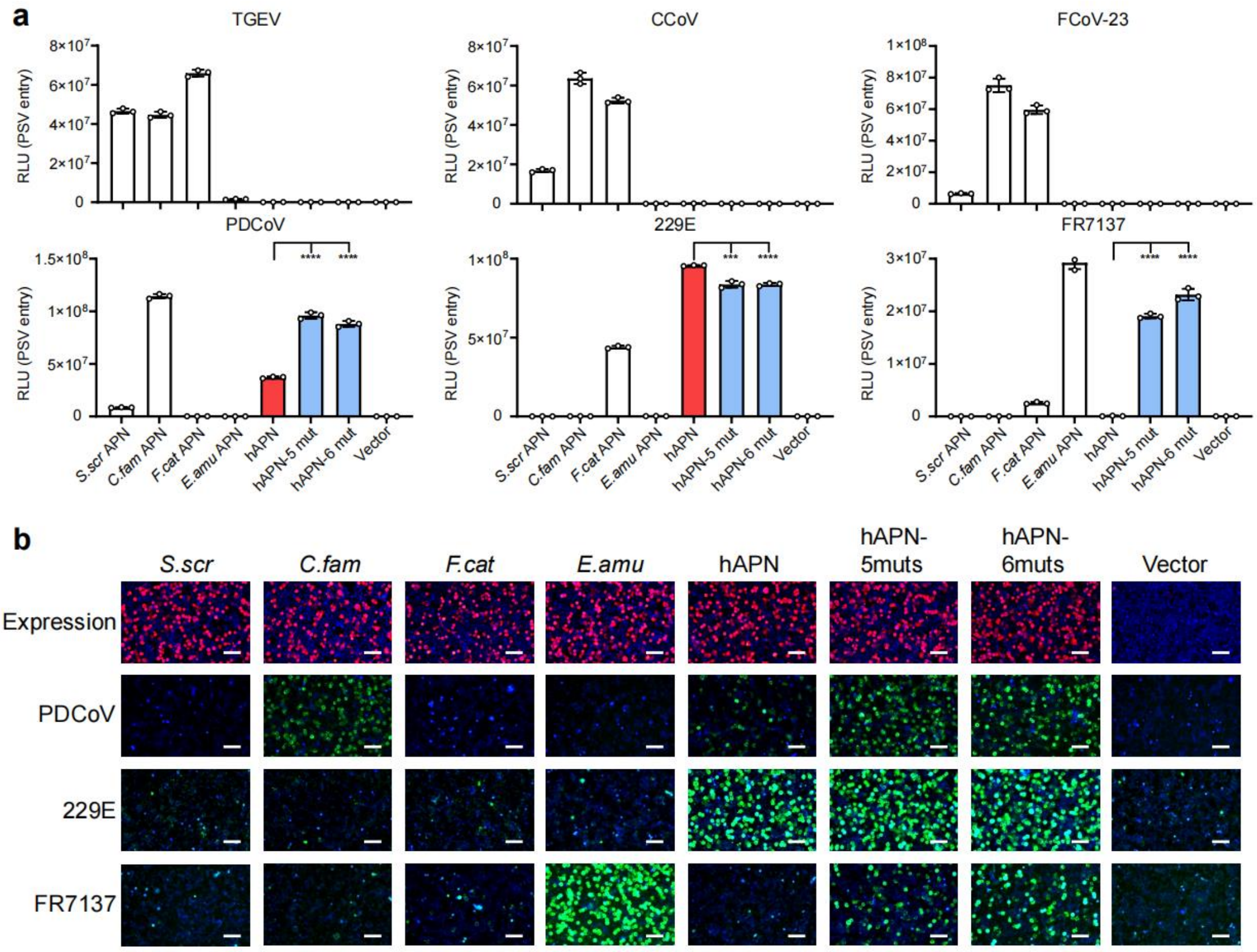

For reviewers, Fig. R1 (partially used in Extended Data Fig. 9f) | Receptor recognition of representative APN-using coronaviruses across indicated APN orthologs and hAPN mutants. (**a**) Entry of VSV-based pseudoviruses carrying APN-using coronavirus spikes into HEK293T cells transiently expressing the indicated APN orthologs or hAPN mutants. (**b**) Binding efficiency of PDCoV, 229E, and FR7137 RBD–hFc proteins to HEK293T cells transiently expressing the indicated APN orthologs or hAPN mutants. Statistical significance was determined by unpaired two-tailed t tests (n = 3 independently infected wells). *: $P<0.05$, **: $P<0.01$, ***: $P<0.005$, and ****: $P<0.001$, NS: not significant. RLU: relative light units. Scale bars: 100 µm.

Result section

Page 16, lines 409–412:

Importantly, these mutations did not significantly impair pseudovirus entry mediated by 229E and PDCoV spikes, indicating that acquisition of EriCoV receptor activity does not compromise other APN–coronavirus interactions, consistent with their distinct binding interfaces (**Extended Data Fig. 9f**).

Reviewer #2 (Remarks to the Author):

Generally summary:

We sincerely thank the reviewer for this exceptionally thorough, rigorous, and insightful evaluation of our manuscript. We particularly appreciate the emphasis on proteolytic activation, biological context, and ecological relevance, which has greatly improved the depth, clarity, and balance of our study.

In response to these comments, we have undertaken substantial experimental and editorial revisions.

(1) Expanded protease analyses. We have substantially expanded the analysis of proteolytic activation by incorporating a wider panel of extracellular and cellular proteases, along with complementary biochemical, pseudovirus entry, and cell-cell fusion assays (**Fig. 5 and Extended Data Fig. 10**).

(2) Refined interpretation of TMPRSS2. We have carefully re-evaluated and moderated our interpretation of TMPRSS2. It is now presented as a context-dependent and strain-specific contributor rather than a key factor required for entry.

(3) Improved biological and ecological relevance. We refined and expanded our APN ortholog panel to include a broader and less redundant set of species, with particular emphasis on ecologically relevant hosts, including potential predators and species in close contact with hedgehogs. In addition, new sequence swap and mapping analyses further define host range determinants.

(4) Expanded discussion of transmission and evolution. We have substantially expanded the discussion to better address ecological and evolutionary implications, including potential transmission routes, spillover risk, and recombination scenarios.

(5) Improved clarity and consistency. To enhance readability, we standardized residue annotation across the manuscript by consistently indicating species origin and aligning sequence, structural, and functional analyses.

Below, we provide a detailed point-by-point response to each comment. We believe that the revised manuscript now addresses the reviewer's major concerns and provides a more balanced and biologically grounded interpretation of our findings.

Thank you again for your insightful and constructive feedback.

Sincerely,

On behalf of all authors

Point-by-point response:

The authors have produced a **comprehensive, logical, and easy to follow stream** of experiments assessing the receptor binding of EriCoVs.

They identify hedgehog APN (also known as ANPEP) as the receptor primary receptor for the virus. In a reasonably comprehensive analysis they test the ability of other species' APN able to mediate fusion or infection of pseudovirus.

Response: We thank the reviewer for this positive assessment of our work. We are pleased that the overall experimental design, logical flow, and integration of multiple approaches are well appreciated.

They find trypsin, in the full-lengths version to have signficant impact on fusion and infection. They also do a limited analysis of proteolytic activation, i.e. limited to TMPRSS2 overexpression experiments and broad-range protease or lipopeptide fusion inhibitors. This experiment is the weakest one in the manuscript. Effect of TMPRSS2 is marginal and not brought into context of a pseudovirus (VSV) that normally enters through Clathrin-mediated endocytosis also due to its size and shape. I have suggested further experiments in the respective section and also would urge to tune down the TMPRSS2 wording in the summary section as trypsin also is highly potent to activate this virus.

Response: We thank the reviewer for this critical and constructive comment. We agree that the original analysis of proteolytic activation was limited and that the role of TMPRSS2 was overinterpreted relative to the data. In the revised manuscript, we have substantially expanded and reorganized this section.

Specifically, we have:

(1) **Expanded the panel of exogenous proteases**, including pepsin, plasmin, elastase, and factor Xa, and systematically evaluated their effects on spike cleavage, cell–cell fusion, and pseudovirus entry (revised **Fig. 5a-c**);

(2) **Assessed the contribution of cell-surface and endosomal proteases** in both HEK293T cells (lacking endogenous TMPRSS2) and Caco-2 cells (with endogenous TMPRSS2), including TMPRSS2 (hedgehog and human), cathepsins (CTSB/L), and furin, using both overexpression and inhibitor-based approaches, and assessed their roles in pseudovirus entry and rcVSV-S amplification (revised **Fig. 5d-f and Extended Data Fig. 10f-h**);

(3) **Evaluated the strain-specific TMPRSS2 usage** across a panel of 20 EriCoV strains (revised **Extended Data Fig. 10c**).

These data consistently show that: (a) macroenvironmental trypsin is the most efficient activator of EriCoV spike; (b) TMPRSS2 provides only a modest, strain-dependent enhancement under overexpression conditions; (c) cathepsin-dependent endocytic entry

represents an important alternative route, particularly in the absence of surface cystine proteases.

Accordingly, we have substantially revised our interpretation throughout the manuscript. We have toned down all statements regarding TMPRSS2 in the Abstract, Results, and Discussion, and it is now described as a context-dependent accessory factor rather than a primary determinant of entry. We have also explicitly discussed the limitations of the VSV-based pseudovirus system.

Like many of these papers, the authors have a very strong focus on human spillover but negate to look at the immediate biology of infetion in the target species and transmission to species that interact closely. I have highlighted this in the section relating to proteolytic activation but also relating to APN species binding. Looking at hedgehog predator species for example would have been interesting.

Response: We thank the reviewer for emphasizing the importance of ecological context and the need to consider the biology and ecology of relevant host species.

In response, we expanded and refined our APN ortholog panel to include species that are ecologically relevant to hedgehogs, including potential predators (e.g., badgers, foxes, golden jackals, and eagles) as well as other mammals with potential close interactions (e.g., shrews, raccoon dogs, and weasels). These analyses show that APNs from these species do not support EriCoV entry, with the exception of felids (cat, leopard, and tiger), which exhibit only marginal receptor functionality. This is consistent with the overall narrow host range observed (revised **Fig. 3d**).

We further investigated the structural basis of this restriction and identified steric constraints at the receptor interface, including glycan-associated barriers, that likely limit cross-species compatibility (**Reviewer Figures below**).

Finally, we have expanded the Discussion to better integrate these findings in an ecological context, including predator–prey interactions, potential recombination scenarios, and their implications for cross-species transmission.

The latter experiments would be interesting to be performed but not essential. The **proteolytic activation experiments**, especially when heading the respective section as such, should be added.

Response: We agree with the reviewer that a more thorough analysis of proteolytic activation is necessary. In response, we have performed additional experiments to systematically assess the roles of both extracellular and cellular proteases in EriCoV entry, resulting in a substantial revision of this section.

We have also revised the section title from "**EriCoV spike proteolytic activation**" to "**Proteases facilitate EriCoV S-mediated entry**" to more accurately reflect the scope of

the updated data and to avoid overinterpretation.

One more minor thing I would like to see are western blots on the mutant APN proteins to show not only that the tag can be stained in a cell but that the protein is not degraded. This would be supplementary data and is indicated further down with respect to the relative figures.

Response: As suggested, we have now included western blot validation for mutant and chimeric APN constructs in **Extended Data Fig. 5 and Fig.4i** (see below), confirming the comparable expression levels and the absence of misfolding or instability.

Graphical abstract:

I was wondering what made the authors chose to highlight PRCV specifically for the APN binding, whereas this is binding to the Alphacoronavirus Suis species in general as they highlight for all the EriCoVs in general. At least it should be broader than PRCV since PRCV is a deletion mutant of TGEV, which is evolutionary “parental”. If we are going down that path, it should even be CCoV as an evolutionary ancestral strain. However, I think αCoV Suis would be most applicable as the binding modes are highly similar.

Response: We agree that PRCV is not the most representative strain in this case (maybe TGEV is better). We originally selected PRCV due to its availability of structural data with its host (TGEV structure is based on dog APN); however, to better reflect the broader biological context, we have revised the figure to refer to αCoV Suis as suggested. We also remove TMPRSS2 to avoid potential over-interpretations.

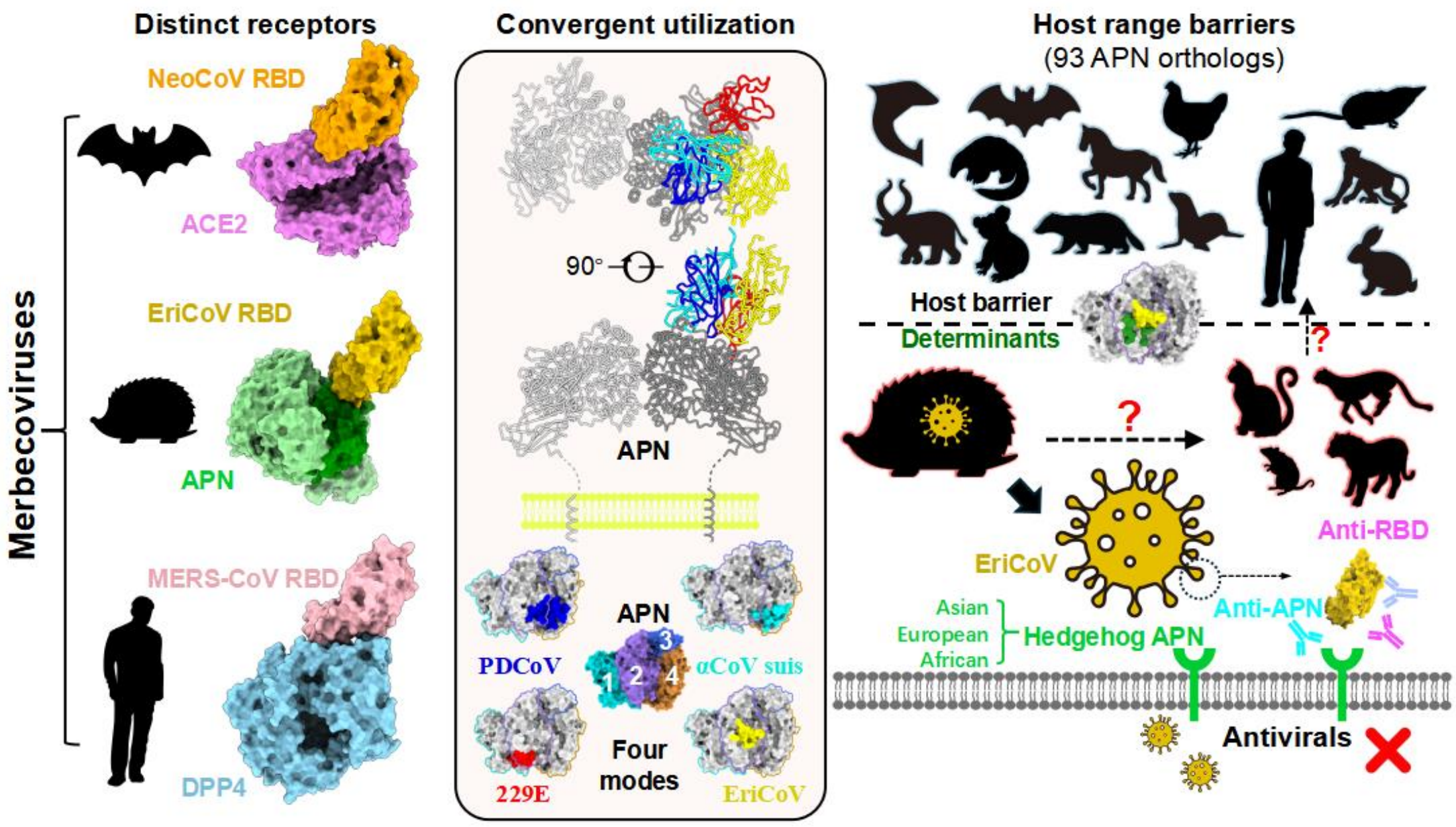

Summary:

As highlighted later in the respective section, I believe TMPRSS2 functionality here is overinterpreted and should be worded more cautiously here. Just because PM fusion is

enhanced, doesn't necessarily mean it's biologically relevant. Especially when using pseudovirus systems based on a different virus family as well.

Response: We thank the reviewer for emphasizing the need to moderate the interpretation of TMPRSS2 in the summary. We have revised the relevant sections to clarify that, while TMPRSS2 can enhance spike-mediated fusion under experimental conditions, its biological relevance remains to be established. We now explicitly present TMPRSS2 usage as a potential, but not dominant, factor, particularly in the context of possible tropism expansion rather than native infection biology. Therefore, we no longer highlight TMPRSS2 in the abstract.

Page 2, lines 37–39:

From: Functional assays further demonstrate that transmembrane serine protease 2 (TMPRSS2) enhances spike activation and promotes plasma membrane fusion.

To: Functional assays suggest that proteolytic activation of EriCoV Spikes can be facilitated by multiple proteases in a context- and strain-dependent manner.

Introduction

Section Line 63fff: Please also highlight what the biology is of EriCoVs in hedgehogs. What is known about their impact in the species itself; what is the infection and transmission route if known?

Response: In the Introduction, we have expanded the description of EriCoV biology in hedgehogs, noting that most detections derive from fecal or rectal samples, consistent with an enteric tropism. We have also incorporated additional introduction of their impact on the health of the host and the potential route of oral-fecal transmission.

Introduction

Page 3, lines 65–81:

In 2013, a novel merbecovirus species (*Betacoronavirus erinacei*, or EriCoV/ErinCoV) was discovered in fecal samples from European hedgehogs (*Erinaceus europaeus*, *E.eur*) in Germany, ***with high viral load in lower gastrointestinal tract***. A related Asian lineage (HKU31) was later found in Amur hedgehogs (*Erinaceus amurensis*, *E.amu*) in China. To date, EriCoVs have been reported exclusively in hedgehogs with high prevalence across a broad geographic range across Euroasia, including Germany, France, United Kingdom, Italy, Poland, Portugal, Russia, Hungary, and China. ***However, so far, no successful isolation or culture of authentic viruses has been reported.*** Among them, all Asian EriCoVs were sampled in *E.amu* hedgehogs, while nearly all European EriCoVs were found in *E.eur* hedgehogs, except for one strain found in *Erinaceus roumanicus* (*E.rou*) hedgehogs and six strains from Moscow annotated as *Erinaceus sp.*. ***Most genomic sequences were amplified from fecal or rectal samples, consistent with enteric tropism and a***

likely fecal-oral transmission route, though several studies found no obvious correlation between RNA viral load and animal health status. Despite their broad geographic distribution and genetic diversity, EriCoVs remain poorly understood in terms of pathogenicity and cross-species ***transmission*** potential.

Line 76: "cross species transmission potential"

Response: Updated, thanks.

Results

Line 182: It would suggest clarifying the experiment for 2a a bit – "supported detectable binding of Fc-tagged RBD protein (Fig. 2a)". Quantification here could have been nice i.e. only positive receptor expressing cells show binding staining but it is pretty black and white so that I don't think this is essential. Similarly, in the figure legend, I presume binding was also confirmed with immunofluorescence staining? Please add this and also compare with Figure 3 legend and make sure the same experiments have at least similar and independently comprehensible legends. In 3 for example you highlight RBD-Fc is green, not in 1.

Response: We thank the reviewer for the suggestions regarding figure quality and experimental description. We have replaced **Fig. 2a** with more clean data with remarkably low non-specific background, clarified the description of the binding assay, and ensured that all relevant figure legends explicitly describe the use of live-cell immunofluorescence based detection. We have also updated legend formats across figures to ensure consistency and clarity.

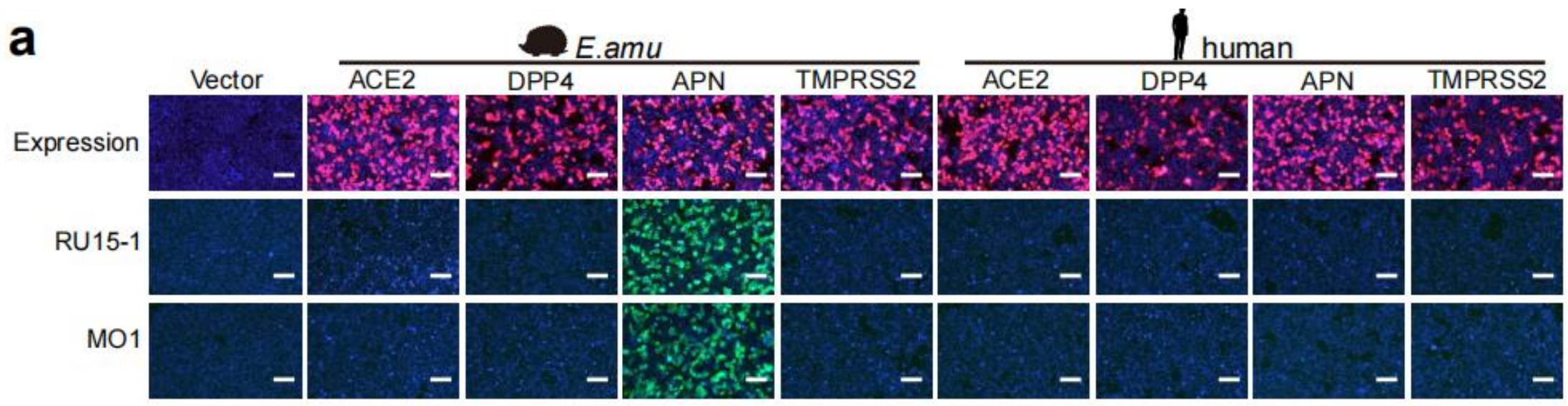

Revised Fig. 2a | Binding efficiency of EriCoV RBD-hFc to hedgehog or human receptors. Transient expression of *E. amurensis* (*E.amu*) APN, but not *E.amu* ACE2, DPP4, TMPRSS2, nor any tested human receptor counterparts, supported EriCoV RU15-1 and MO1 RBD human IgG1 Fc domain (hFc) fusion protein (RBD–hFc) binding (green) assessed by live-cell immunofluorescence using an anti-hFc antibody. Receptor expression was confirmed by immunofluorescence staining: FLAG-tagged ACE2, DPP4, and APN, and ALFA-tagged TMPRSS2 (red). Scale bars: 100 μm.

Page 10, lines 213–215:

Figure legends:

From: supported EriCoV RU15-1 and MO1 RBD human IgG1 Fc domain (hFc) fusion protein (RBD-hFc) binding (a).

To: supported EriCoV RU15-1 and MO1 RBD human IgG1 Fc domain (hFc) fusion protein (RBD-hFc) ***binding (green) assessed by live cell immunofluorescence using an anti-hFc antibody*** (a).

From: IT5-12 RBD-hFc binding (f)
To: IT5-12 RBD-hFc binding ***(green)*** (f)

From: assessed by immunofluorescence using an anti-hFc antibody.
To: assessed by **live cell** immunofluorescence using an anti-hFc antibody.

Note: relevant legends were also updated as suggested.

Line 183: This is the first introduction of the pseudoviruses in the main body of text. Pseudoviruses can be made on a variety of backgrounds that impact entry pathways. Therefore, please introduce that this is a VSV-based pseudovirus used in these experiments and for all the following experiments. I would urge to add this information to all the figure legends where pseudoviruses are used, i.e. VSV-pseudovirus mentioned at least once in the legend to each figure where the authors used pseudoviruses.
Response: We thank the reviewer for this important point. We have now clarified at the first mention in the main text that all pseudovirus experiments are based on a VSV pseudovirus system and have ensured that this information is consistently included in all relevant legends.

Page 8, lines 190–191:
From: supported detectable RBD binding (Fig. 2a) and RU15-1 and MO1 pseudovirus entry.

To: supported detectable RBD binding (Fig. 2a) and RU15-1 and MO1 ~~pseudovirus~~ entry evaluated by ***Vesicular Stomatitis Virus (VSV)-based pseudovirus***.

Note: relevant legends were also updated as suggested.

Line 185: Interesting that the authors chose 229E RBD rather than any of the arguably more closely related APN-binding coronaviruses. Maybe highlight that this reciprocal experiment is limited by adding a line; No other APN-binding coronaviruses were tested in this experiment. (Just to remind the reader immediately that there are other APN-binding coronaviruses that may bind to hedgehog APN. In case I forget this later, worth discussing this in the discussion as well that recombination events could be reciprocal as well, i.e. hedgehogs being infected by a virus from a more closely interacting species, for example **badgers**, natural predators of hedgehogs, who recently were shown to carry an alphacoronavirus; 10.1016/j.meegid.2023.105406).

Response: We thank the reviewer for this insightful comment. We selected 229E because it is a well-established APN-using human coronavirus and, to our knowledge, no other more related strain within betacoronavirus genus is known to use APN. To address this point more comprehensively, we have included additional APN-binding coronaviruses

and test their ability to use APN orthologs or mutants (as suggested by reviewer 1). We found most tested well-studied APN-using viruses (229E, CCoV-Hu-2018, FCoV-23, PDCoV) were almost unable to recognize hedgehog APN (cf. **Reviewer Fig. R2** below). In light of this, 229E remains a good choice for this experiment and we did not extend the pull-down assay to additional APN-binding coronaviruses, and instead **included human APN as a positive control** for 229E binding (**revised Fig. 1e**).

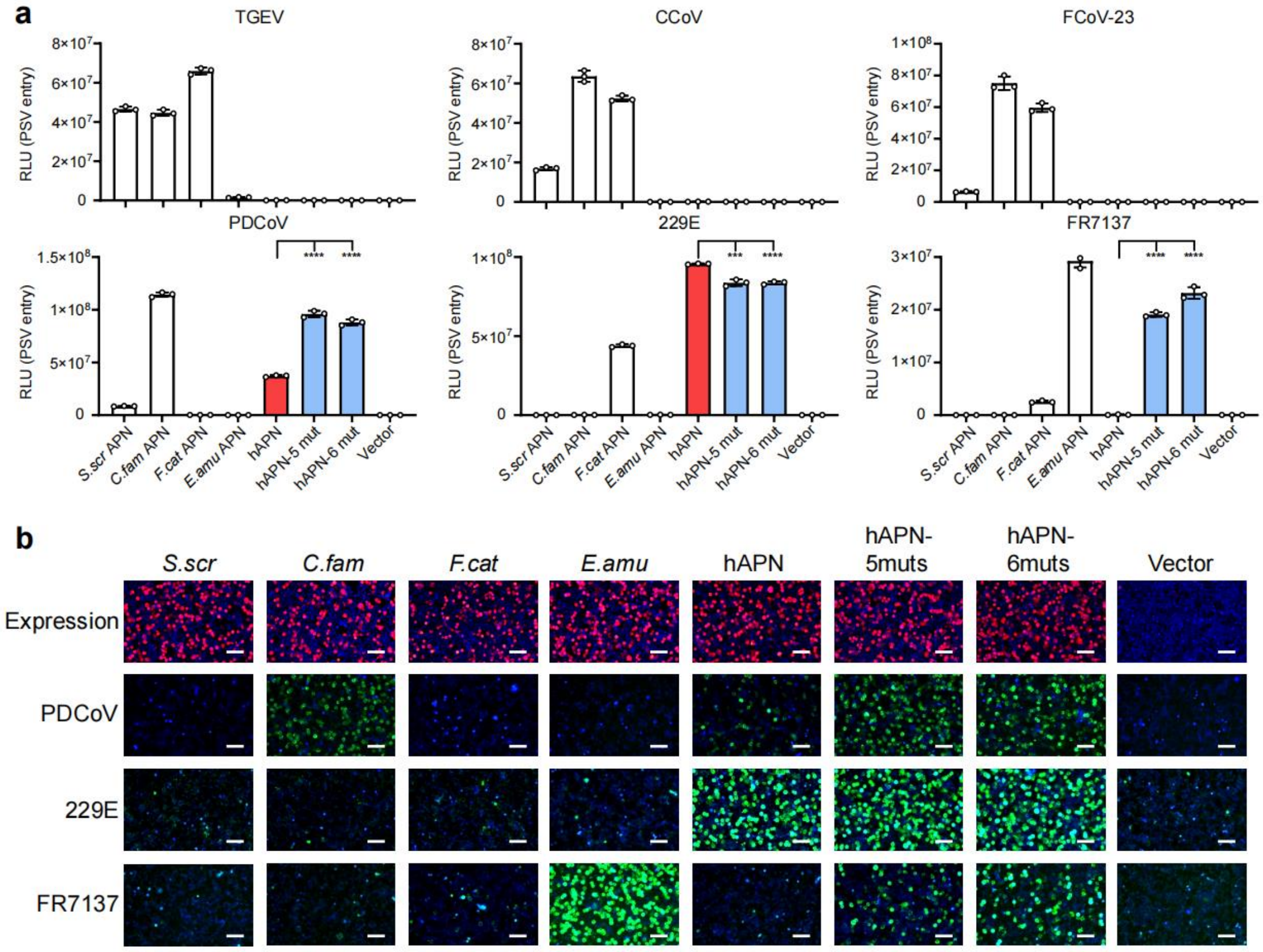

For Reviewers, Fig. R2. | Pseudovirus entry and RBD binding of APN-using coronaviruses in HEK293T cells transiently expressing indicated APN orthologs or indicated human APN mutants (**a**) VSV-based pseudoviruses entry of APN-using coronaviruses in HEK293T cells transiently expressing APN orthologs and mutant hAPN. (**b**) Binding of PDCoV, 229E, and FR7137 RBD-hFc to HEK293T transiently expressing APN orthologs and mutant hAPN. Statistical significance was determined by unpaired two-tailed *t* tests (n = 3 independently infected wells). *: $P<0.05$, **: $P<0.01$, ***: $P<0.005$, and ****: $P<0.001$. RLU: relative light units. Scale bars: 100 μm.

Note: hAPN-5mut and hAPN-6mut were also included here to address comments by Reviewer 1

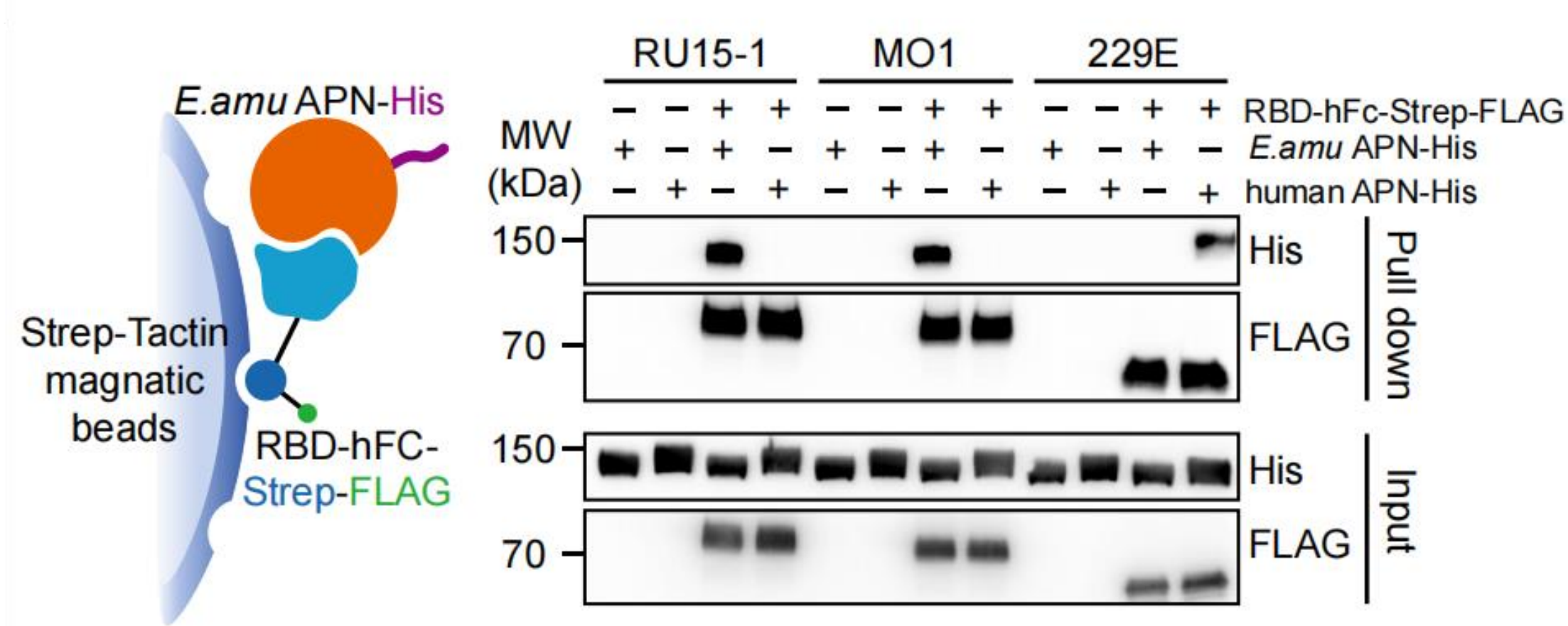

Revised Figures 1e | Pull-down assay of RBD-hFc-Strep-FLAG interacting with APN-His. Western blot shows specific interaction of soluble *E.amu* APN ectodomain (His-tagged) with Strep-Tactin-immobilized RU15-1 or MO1 RBD–hFc-Strep-FLAG fusion proteins, with 229E RBD as a negative control.

We have noticed the paper you mentioned (10.1016/j.meegid.2023.105406). Interestingly, new findings indicate this virus found in badgers is probably a now genus (ε) or even subfamily of coronavirus with segmented genome and unknown receptor (10.1016/j.cell.2025.08.016).

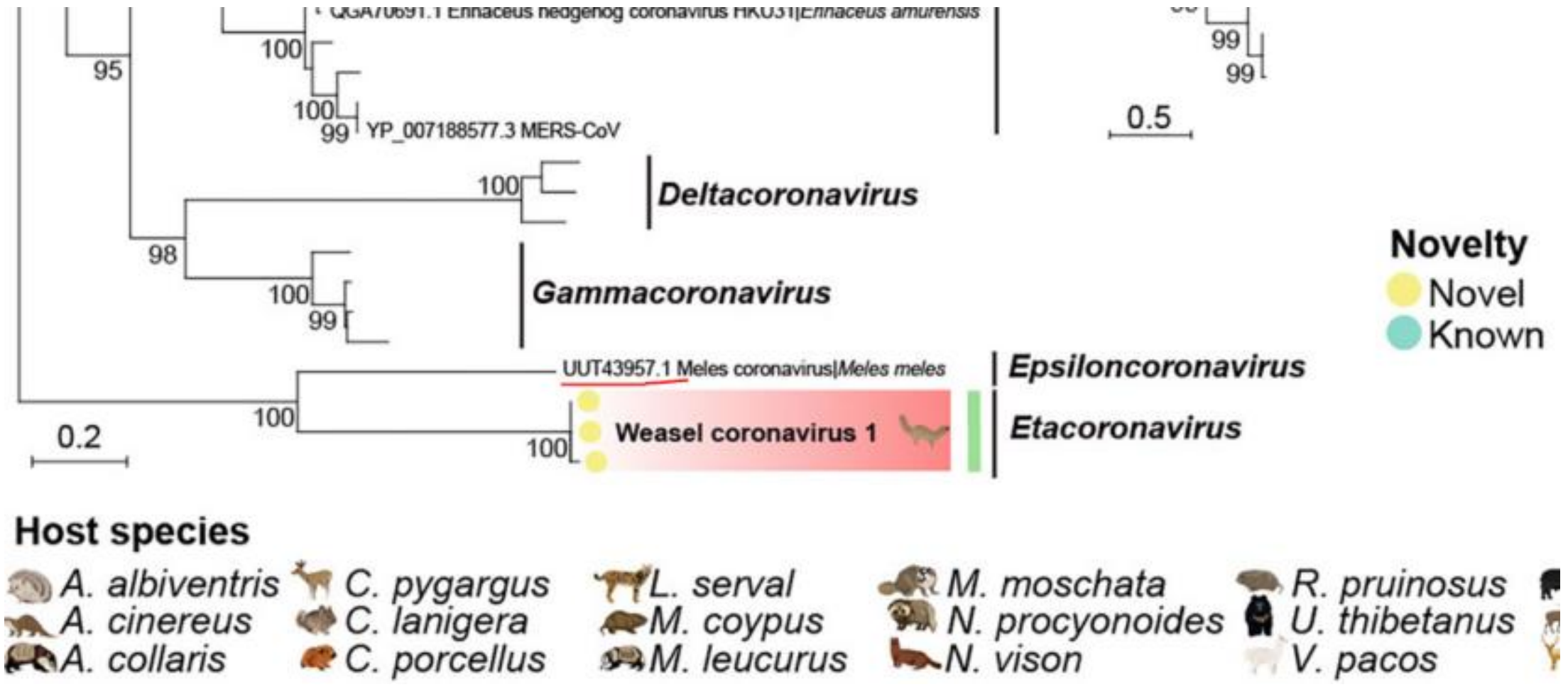

Cell 2025;10.1016/j.cell.2025.08.016

We also appreciate the reviewer's suggestion regarding ecological context and reciprocal recombination. We have now expanded the Discussion to address the possibility of virus exchange between hedgehogs and closely interacting species, including predators. In this context, we note that hedgehogs have been reported to harbor HKU8-related alphacoronaviruses (10.1016/j.onehlt.2024.100940), suggesting potential opportunities for co-infection. However, the likelihood of recombination across distant coronavirus lineages remains uncertain and may be constrained by substantial genetic barriers.

Updates in Discussion

Page 28, lines 741–748:

It is also important to consider that spillover events could be reciprocal and recombination could occur. For example, natural predators of hedgehogs, ***such as badgers, which have been reported to harbor coronaviruses***, may represent potential interfaces for cross-species transmission or recombination. Although most coronavirus found in hedgehogs belongs to the betacoronavirus EriCoV lineage, hedgehogs themselves could be coinfected by other coronaviruses, which may further shape EriCoV evolution. ***For example, alphacoronavirus belonging to the Alphacoronavirus miniopteri (BtCoV-HKU8) has been recently identified in hedgehogs in Portugal***.

Line 189: I would suggest that the authors use the word peptidase NOT protease activity. Whilst technically proteolytic, it's a term not quite appropriate in the common use of protease activity.

Response: We thank the reviewer for this precise terminology suggestion and updated as suggested.

Page 8, lines 198:

From: indicating that receptor function is independent of ***protease*** activity

To: indicating that receptor function is independent of ***peptidase*** activity

Lines 192 fff and figure 2 h & i: I like the figures and the results, clearly indicating a possible and likely enteric infection route of this virus (would have been interesting to see whether pepsin has similar activity). Nonetheless, I would just highlight either in the results or the figure legend some notes about cell health in the face of such high levels of trypsin. The black shades in the background show significant clumping up of cells; I am actually surprised that

Response: We thank the reviewer for this helpful comment. We agree that the strong trypsin dependence for efficient spike activation is consistent with a likely enteric tropism of EriCoVs, and we have now made this point more explicit in the revised manuscript.

As suggested, we also evaluated pepsin. Under our indicated experimental conditions, pepsin did not promote spike cleavage or enhance pseudovirus entry (cf. **Reviewer Fig. R3** below). This is likely due to its strict requirement for highly acidic pH (typically <4), which is incompatible with the neutral pH conditions of our cell line-based assays. Therefore, pepsin was not included our protease panel for functional assays. These observations are consistent with previous reports showing that pepsin can not boost infection and low pH resulted in the reduction of coronavirus infectivity (doi: 10.1128/jvi.00645-24; doi: 10.1136/gutjnl-2021-326624). We have therefore not included pepsin in the main functional protease panel.

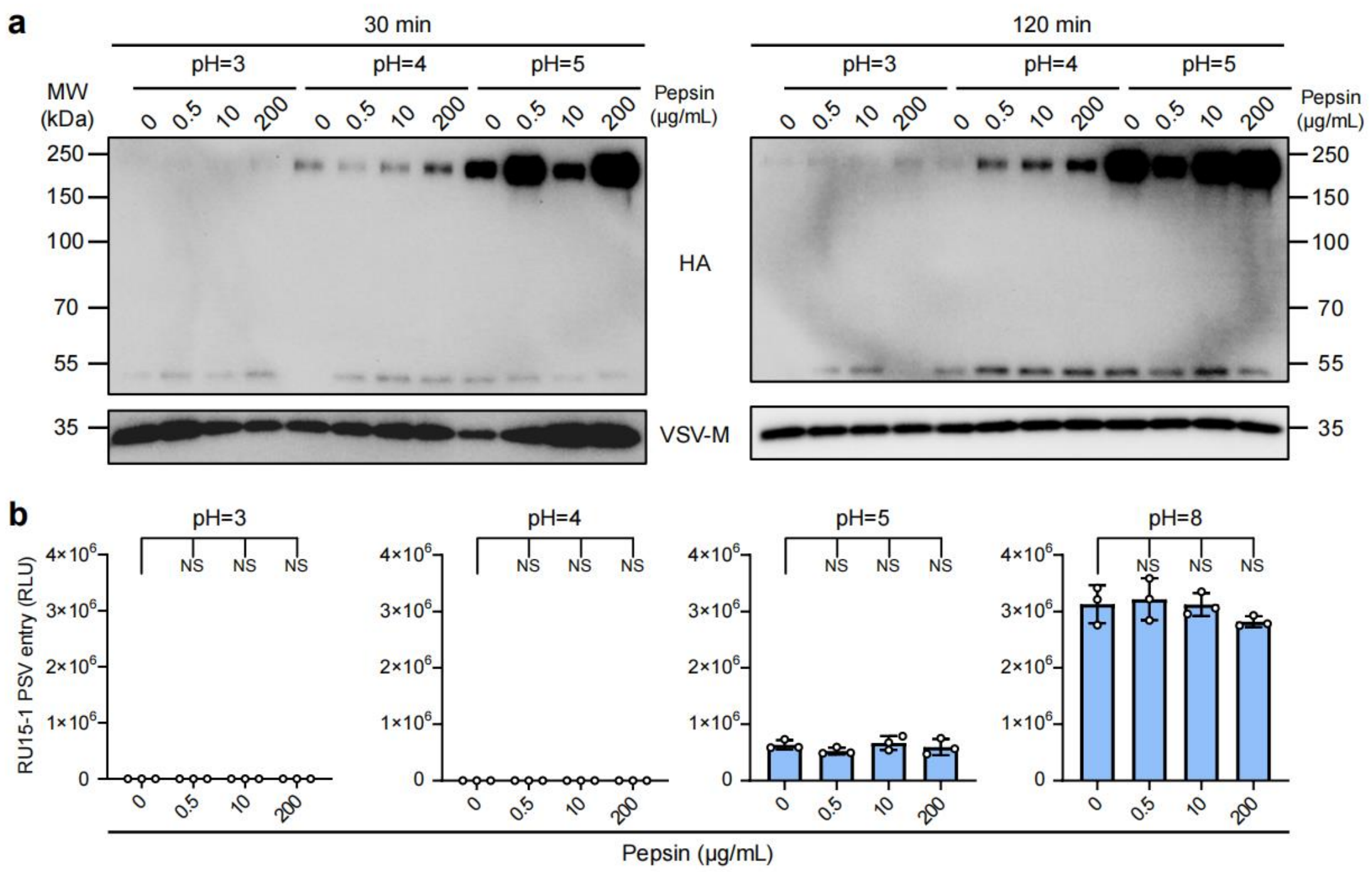

For reviewers, Fig. R3 | Effect of pepsin treatment for EriCoV S cleavage and pseudovirus entry. (**a**) Detection of RU15-1 S glycoprotein cleavage via Western blot using an anti-HA antibody targeting the C-terminally fused HA tag. Concentrated pseudoviruses were treated with pepsin at varying concentrations under different pH (adjusted by citric acid-disodium citrate buffer) at 37°C for 30 minutes or 120 minutes. VSV-M served as a loading control. (**b**) Pseudovirus entry of RU15-1 in HEK293T transiently expressing *E.amu* APN. Pseudoviruses were pretreated with pepsin for 1 h at 37°C. Statistical significance was determined by one-way ANOVA followed by Dunnett' s test (n = 3 independently infected wells). *: $P<0.05$, **: $P<0.01$, ***: $P<0.005$, and ****: $P<0.001$, NS: not significant. RLU: relative light units. PSV: pseudovirus.

Regarding cell health, we have now added clarifications in the figure legends to indicate that although some cell rounding and clustering are observed, cells remained attached and viable under the conditions used (cf. **Reviewer Fig. R4** below). Importantly, these morphological changes did not affect the interpretation of the entry and fusion assays, which were consistently reproducible across independent experiments.

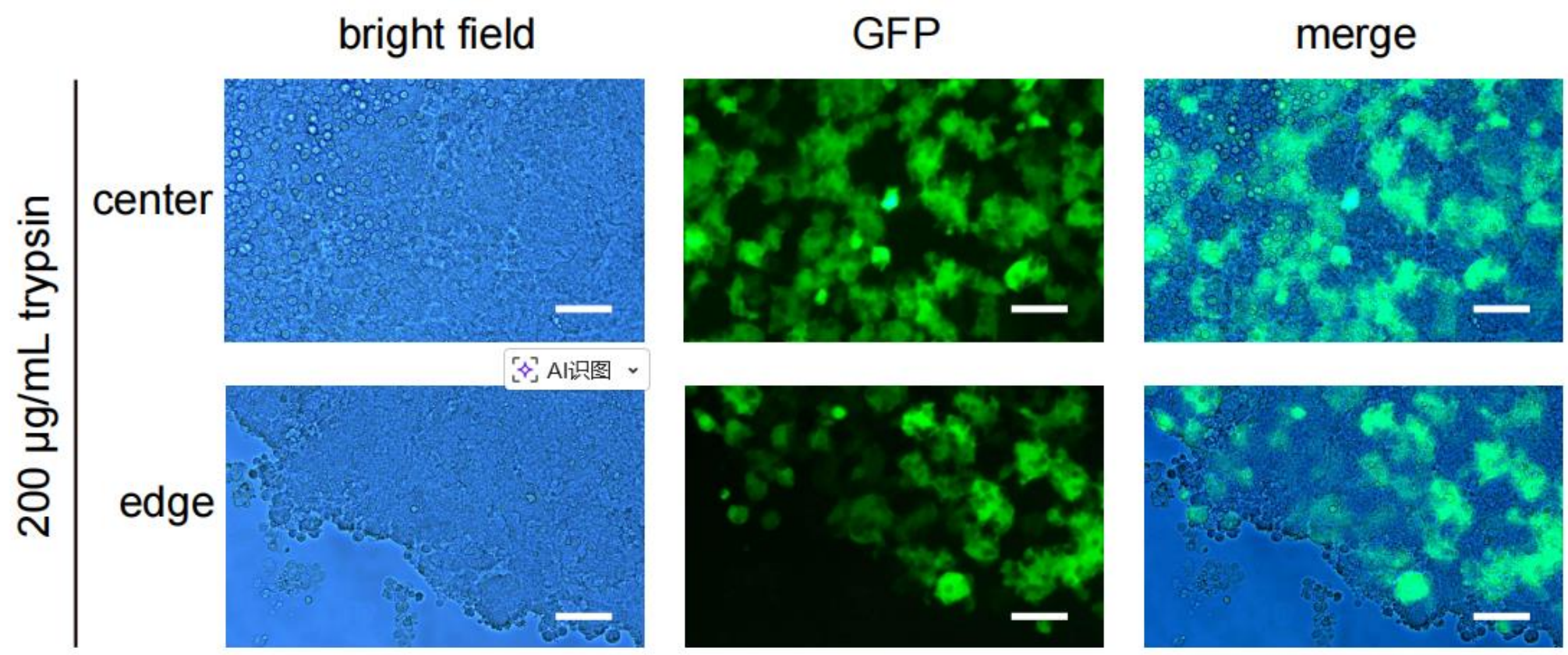

For reviewers, Fig. R4 | Cell morphology and fluorescence intensity in S-mediated cell-cell membrane fusion assay. HEK293T cells were treated with 200 µg/mL trypsin at 37°C for 30 minutes, trypsin activity was then quenched by adding the medium with DMEM supplemented with 10% FBS. Images were captured 1.5 hours post medium change. Scale bars: 100 µm.

Page 10, lines 228–231:

Legends for Fig. 2h:

Trypsin activity was then quenched by adding the medium with DMEM supplemented with 10% FBS. Images were captured 1.5 hours post medium change. Clumped cells remained viable and adherent to plates despite high trypsin concentrations.

Ortholog specificity and host tropism determinants

Line 234: suggest adding “APNs from shrews Sorex … “

Response: Updated as suggested, thanks.

Page 11, lines 250–251:

From: APNs from Sorex

To: APNs from ***shrews*** Sorex

Line 247: suggest “all tested species of ERiCoVs”

Response: Thank you, we amended it to “20 tested EriCoV strains”.

Page 11, lines 259:

From: can support efficient binding of all tested ***species***.

To: supporting entry of all 20 tested ***EriCoV strains***.

Line 249: suggest “the GER216, FR7137, and PLJ64 strains [or strain spikes] are also capable …”

Response: Thanks, we have added the word “strains”.

Page 11, lines 267–269:

From:The GER216, FR7137, and PLJ64 are...

To: Expanded validation across additional EriCoV strains confirmed that these orthologs support suboptimal RBD binding and pseudovirus entry, particularly for ***European strains***.

Figure 3: For experiments related to the chimera figures, 3f, j, and i; I would like the authors to show a western blot with these chimeras in the supplementary data, just to show these proteins do not show signs of misfolding and degradation. Whilst these two species show about 76% sequence identity, some of the areas are significantly different that this must be a concern.

It is a real shame that the authors didn't include biologically important species in this experiment as well, i.e. main predator species of hedgehogs, such as badger species. That would be an important assessment for risk of cross species transmission.

Response: Thank you for your suggestion related to expression validation and biologically important species, we have addressed both points:

(1) Western blot validation of the chimeric APN constructs has been added (updated **Extended Data Fig. 5**), confirming stable expression without misfolding or degradation.

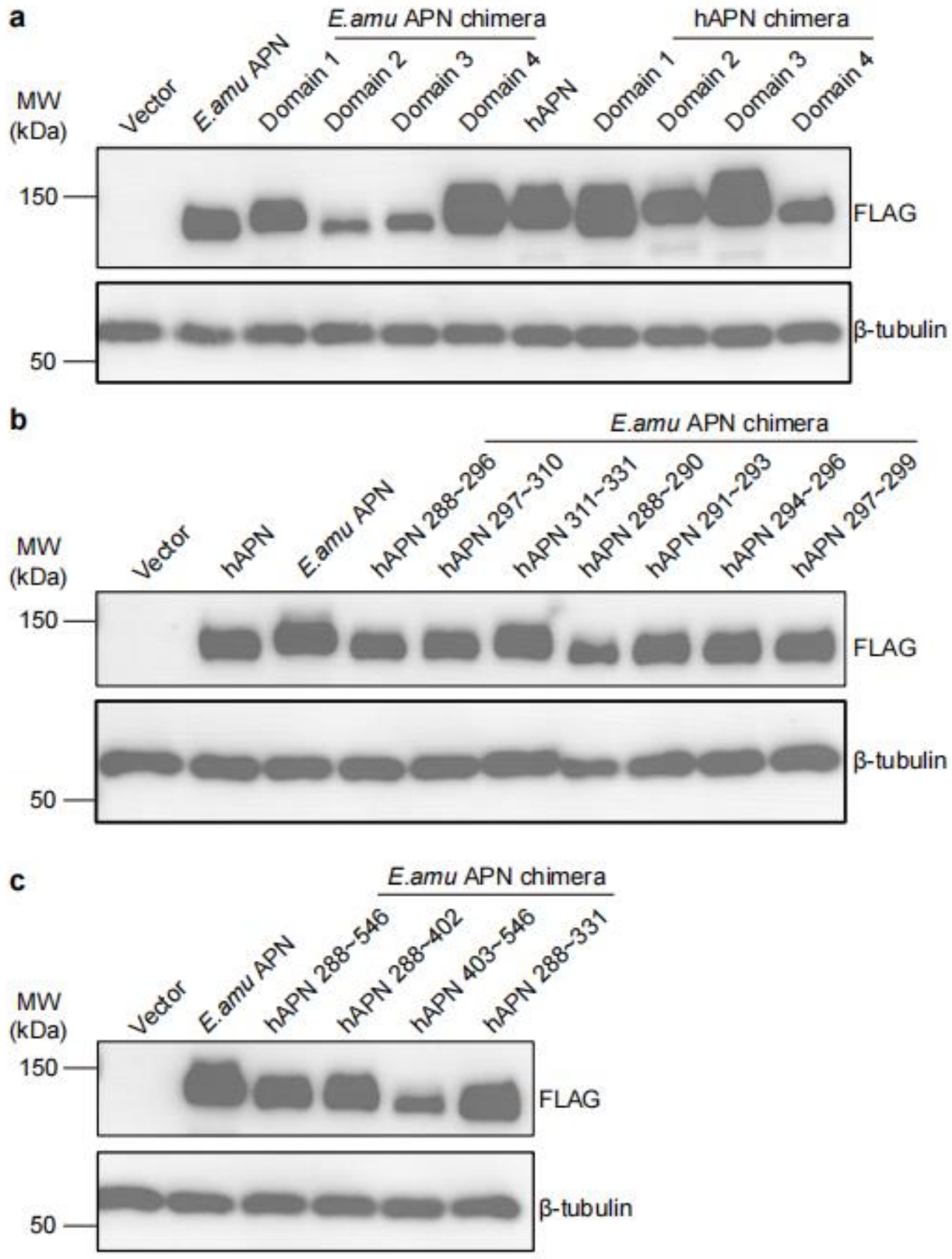

Extended Data Fig. 5 | Expression efficiency of *E.amu* APN and human APN chimera. (a-c) Western blot validation of the expression of chimeric APN constructs of Fig. 3g, 3i, and 3j by detecting the C-terminally fused FLAG tags. β-tubulin served as a loading control.

(2) In response to biologically important species, we have expanded our analysis to

incorporate APN orthologs from a broader panel of species with ecological relevance to hedgehogs, including natural predators (e.g., badgers), opportunistic predators (e.g., Red fox, golden jackals, and weasels), as well as other interacting mammals (revised **Fig. 3d**). Our results show that, among the species tested, only felid APNs (cat, tiger, and leopard) support FR7137 EriCoV pseudovirus entry, whereas APNs from badgers and other predators/potential predators do not. RU15-1 and MO1 displayed narrower APN tropism in RBD binding assays, further supporting a restricted host range (revised **Extended Data Fig. 3**). Structural analysis suggests that this lack of compatibility is associated with unfavorable residues at the receptor interface, including glycan-associated steric constraints. For example, the presence of an interface glycosylation in the badger APN (*M.mel*) may interfere with EriCoV binding (cf. **Reviewer Fig. R5** below).

We also discussed these results and their implications in the revised discussion section.

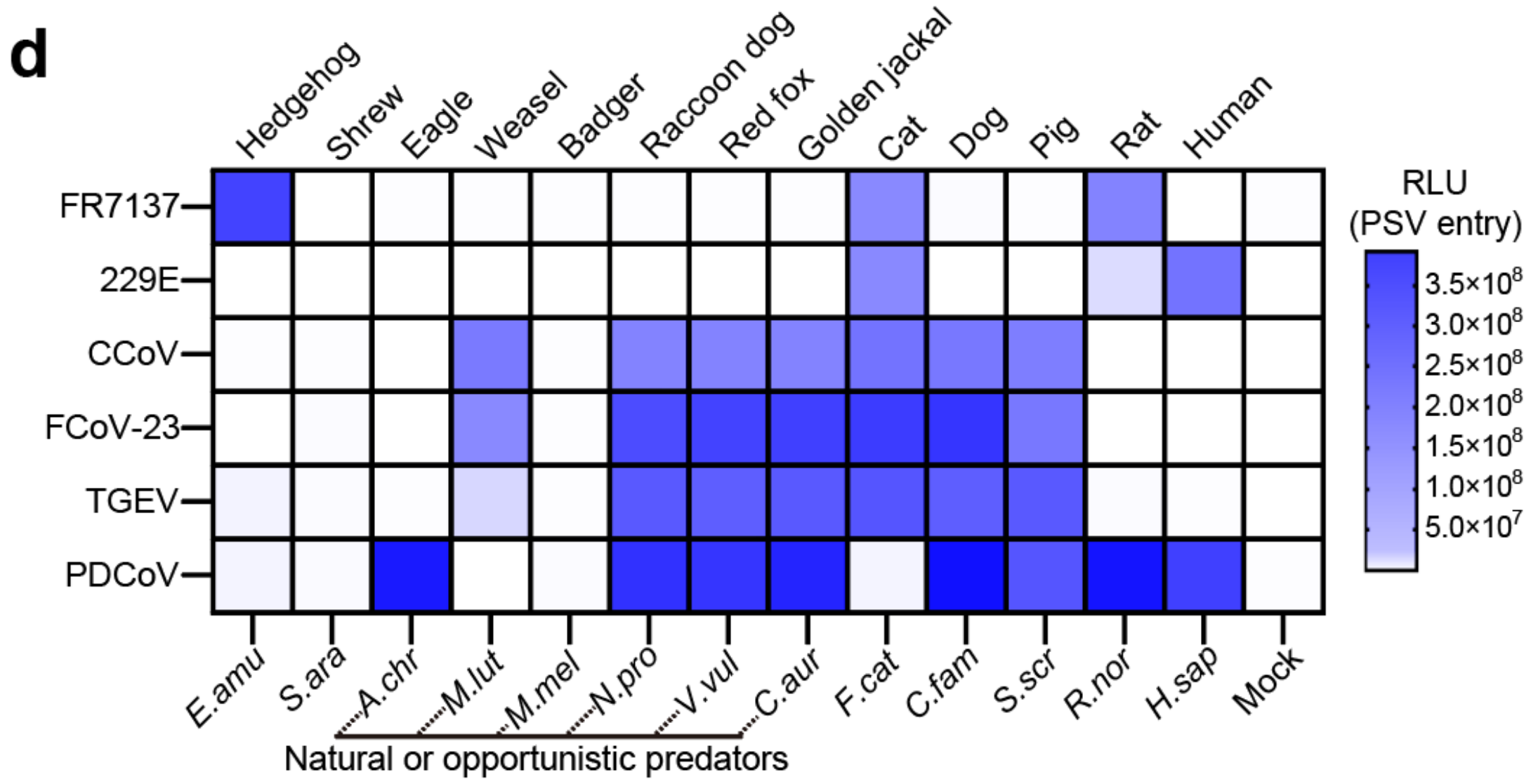

Fig. 3d | Coronavirus ortholog tropism of APN from species biologically or ecologically related to hedgehogs. Heat map of pseudovirus entry of APN-using coronaviruses in HEK293T expressing APN orthologs including potential predators or species with overlapping habitats of hedgehogs. Pseudoviruses were pretreated with trypsin. Data are presented as the Mean of 3 independently infected wells.

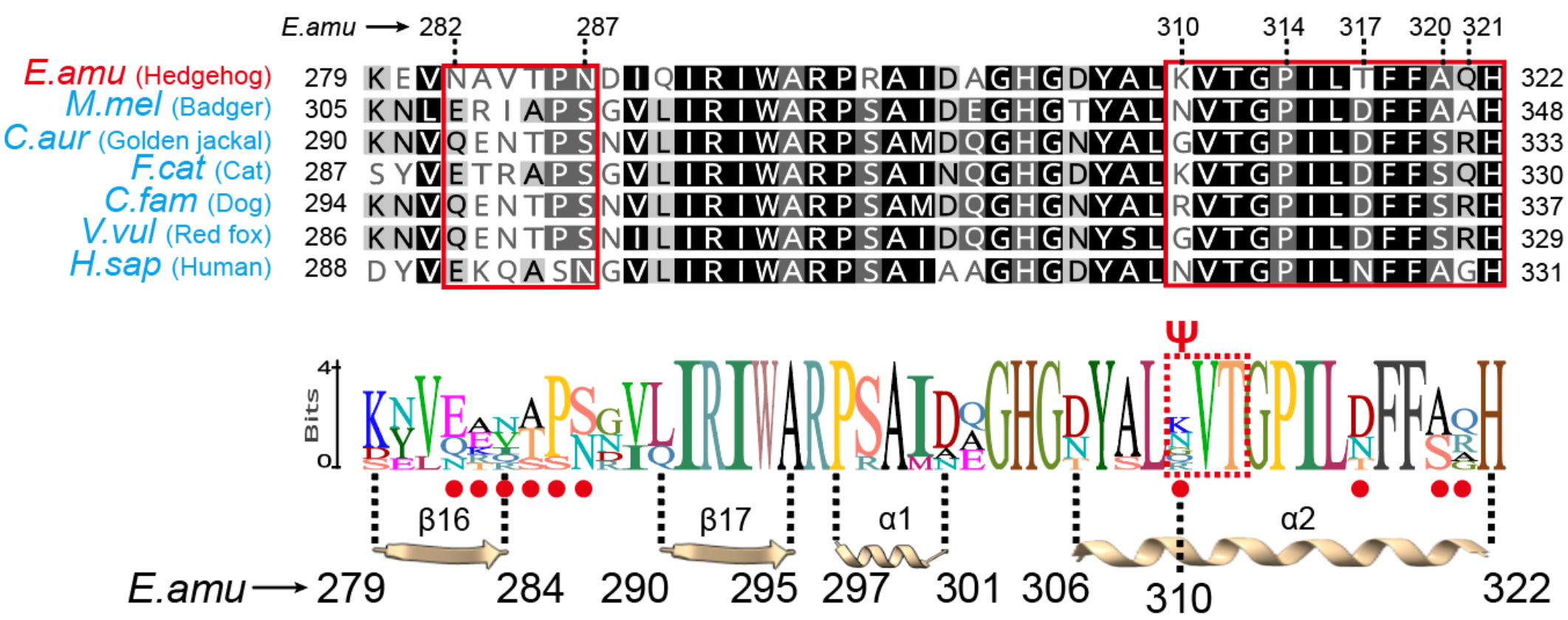

For reviewers, Fig. R5 | Sequence alignment of selected natural/opportunistic

predator-related APN orthologs and logo plot displaying the polymorphisms across 93 APN orthologs. Key secondary structures and predicted residues involved in host range determination according to data of Fig. 3i-j and Extended Data Fig.6 were marked with red dots. Red boxes: predicted hot-spot regions contributing to host range determination.

Updates in Results:

Page 11, lines 264–266:

Compared with other APN-using coronaviruses, EriCoVs consistently exhibit restricted receptor usage, as only felid and rat APN orthologs support limited FR7137 pseudovirus entry, whereas APNs from natural or opportunistic predators (e.g., badgers) are not compatible.

Updates in Discussion:

Page 28, lines 730–736:

This suggests that, under current conditions, EriCoVs face a substantial barrier to cross-species transmission. While current data suggest a relatively low risk of spillover through natural predators of hedgehogs, such as badgers, as their APNs are resistant to EriCoV recognition (Fig. 3d). However, the detectable, albeit weak, compatibility with felid APN for several European strains indicates that relatively minor changes at the receptor-binding interface—either in APN or the viral RBD—could lower the host barrier.

Page 28, lines 743–744:

For example, badgers have been reported to harbor coronaviruses, may represent potential interfaces for cross-species transmission or recombination.

Line 262: highlight which species contains the glycosylation site.

Response: We have clarified in the text which species contain the glycosylation site.

Page 11, lines 281–282:

From: and the latter contains an N319 glycosylation (Fig. 3h-j).

To: and the latter contains an N319 glycosylation ***in human APN, but not in E.amu APN*** (Fig. 3h-j and Extended Data Fig. 5a-c).

Line 267: I think the statement in 267 is too definite. Given the authors haven't done further experiments with recombinants of F. Cat or P. Par they cannot say this "likely explains their receptor compatibility." I would say "could be an explanation for their receptor compatibility."

Response: OK, we have update it to "could be an explanation for their receptor compatibility", as suggested. We also conducted swap experiments based on F.cat APN (**Extended Data Fig. 6**).

Page 12, lines 288–289:

From: likely explains their receptor compatibility.

To: could be an explanation for their receptor compatibility.

Furthermore, in relation to figure k: How did the authors decide on critical residues involved in host range determination? I cannot quite follow the thought process here, for example, I kind of understand the first stretch of 6 amino acids, though this has not been experimentally validated, e.g. swapping this section into **feline APN** may be very insightful? In the latter region, 326 I can again not follow. There is a clear difference in hedgehog but why this may be determining I don't understand. Do the boxes here reflect the alpha2 and beta 16 regions at all? More rationale and explanation in the results section is needed here.

Response: We apologize for the lack of clarity and agree that the rationale for residue selection was not sufficiently explained in the original version. We have now revised both the Results section and related legend to clearly describe how these residues were predicted and validated.

The initial prediction of the critical residues of the "first stretch of 6 amino acids" in the β16 region and other key residues within the α2 region was based on sequence alignment, sequence logo analysis, structural considerations such as surface exposure, which is preliminary validated by *E.amu* APN and human APN swap analysis in **Figure 3i,j**.

To directly address the reviewer's suggestion and experimentally validate these predictions, we performed sequence swap experiments between hedgehog (*E.amu*) APN and feline (*F.cat*) APN, both of which lack the restrictive $N319_{hAPN}$ glycosylation. Specifically, we generated seven *E.amu* APN mutants in which divergent regions between the two receptors were replaced with the corresponding feline sequences. These experiments demonstrated that **mutant 2** with substitutions in the β16 (the 6-aa divergence) and **mutant 6** with substitutions in the α2 region ($T_{E.amu}317D_{F.cat}$ and $A_{E.amu}320S_{F.cat}$) showed reduced RBD binding, whereas mutations in other regions had minimal effects (revised **Extended Data Fig. 6**). These results provide direct functional evidence that residues surrounding β16 and α2 are critical determinants of receptor compatibility. These regions are located in the boxed segments highlighted in revised **Fig. 3k** and are located near the RBD–APN interface in the structural analysis (**Fig. 4g**).

The previously mentioned position 326 in human APN ($D317_{E.amuAPN}$) is highly variable, and we predict is as a key residue for host range determinants. We agree with the reviewer that our original interpretation in the figure legend was too speculative. In the revised manuscript, we have toned down this claim and instead emphasize that mutations in the α2 ($T_{E.amu}317D_{F.cat}$ and $A_{E.amu}320S_{F.cat}$) collectively contribute to host range determination, as supported by the reduced binding observed in the corresponding mutants.

Finally, we clarify that the red boxes in Fig. 3k indicate regions predicted to contribute to host range determination, rather than strictly corresponding to specific secondary structure elements such as β16 or α2. This has now been explicitly stated in the figure

legend.

Together, these revisions provide a clearer and experimentally supported rationale for the identification of host range determinants.

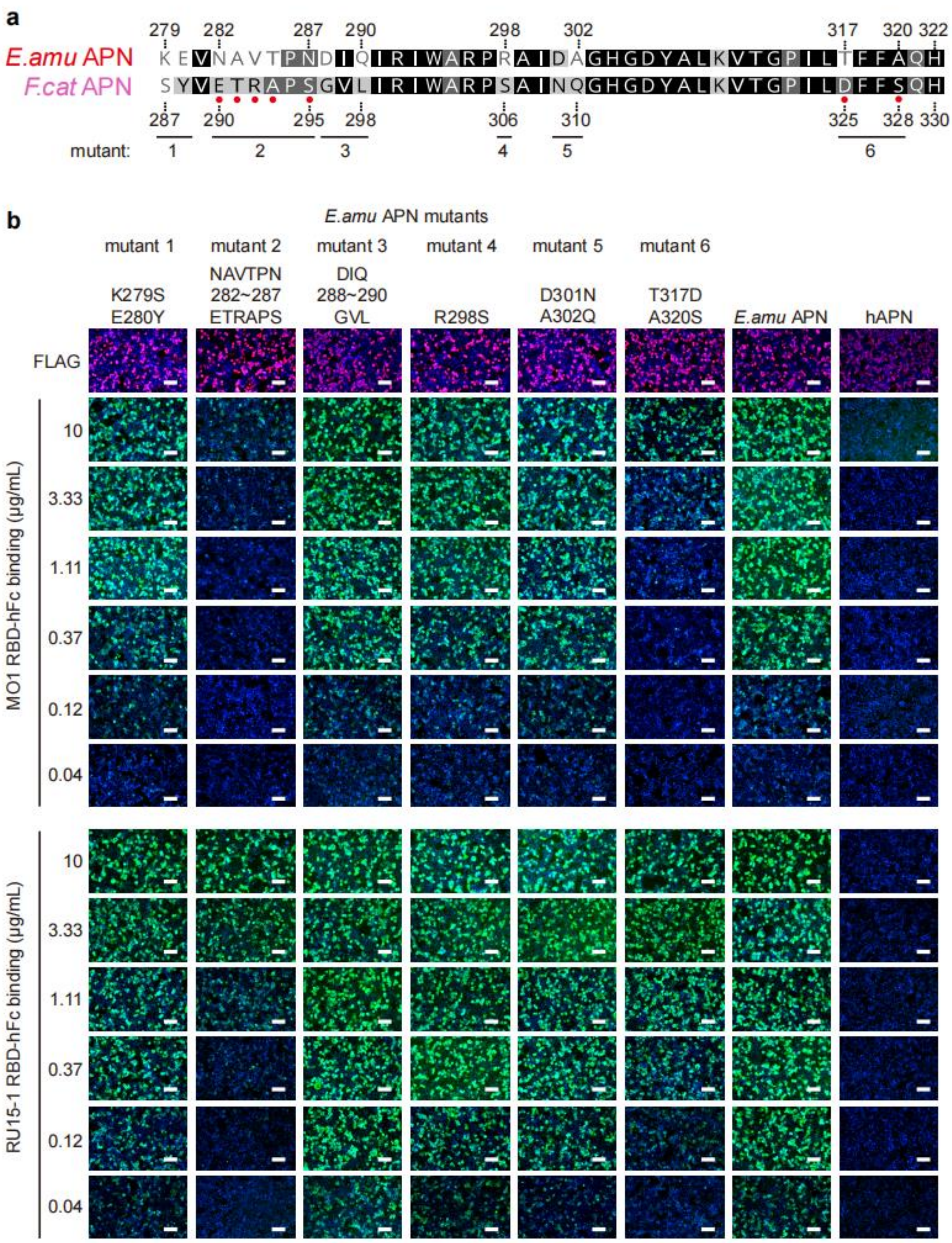

Revised Extended Data Fig. 6 | Fine mapping of the host range determinants in domain 2 by substituting divergent residues of *E.amu* APN with *F.cat* APN counterparts. (**a**) Sequence alignment highlighting amino acid differences between *E.amu* APN and *F.cat* APN within the mapped region. Residues implicated in host range determination are indicated by red dots. (**b**) Binding efficiency of MO1 and RU15-1 RBD–hFc to HEK293T transiently expressing *E.amu*

APN mutants. Recombinant proteins were diluted in SMM 293-TII Expression Medium at the indicated concentrations. Scale bars: 100 µm.

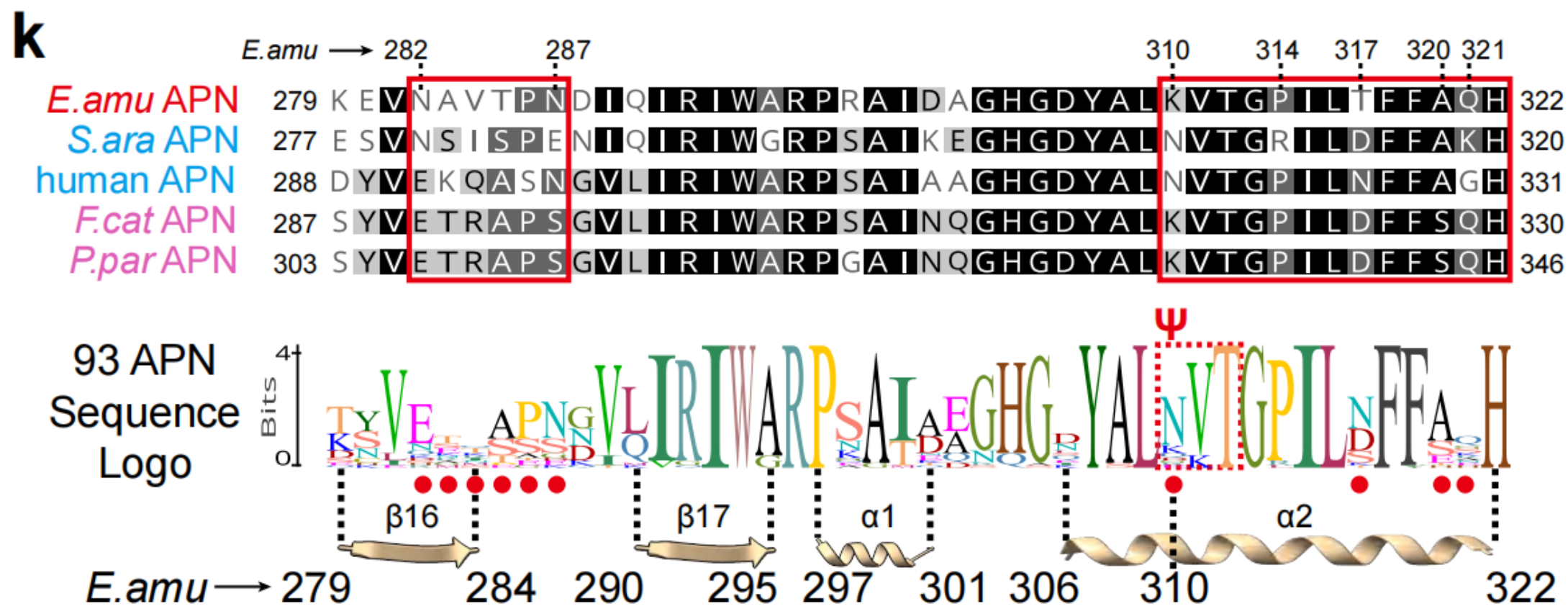

Fig. 3k | Sequence alignment of selected APN orthologs and logo plot displaying the polymorphisms across 93 APN orthologs. Sequence alignment of selected APN orthologs and logo plot displaying the polymorphisms across 93 APN orthologs. Key secondary structures and predicted residues involved in host range determination according to data of panel 3i-j and Extended Data Fig.6 were marked with red dots. Red boxes: predicted hot-spot regions contributing to host range determination. N319$_{hAPN}$ glycosylation sequon is indicated with red dashed box, and the modification site marked with Ψ.

Updated Result section

Page 12, lines 289–294:

Consistent with this, substitution of E.amu APN residues with the corresponding residues in F.cat APN identified critical residues within β16 (positions 282–287$_{E.amuAPN}$) and α2 (T317$_{E.amuAPN}$ and A320$_{E.amuAPN}$) that are required for high affinity binding with RU15-1 and MO1 RBDs (Extended Data Fig. 6a-b). Collectively, these results define several structurally constrained molecular determinants that underlie the restricted host tropism of EriCoVs.

Updated Figure 3 legend

Page 14, lines 316–319:

From: Key secondary structures and ***critical*** residues involved in host range determination were marked with red dots.

To: Key secondary structures and ***predicted*** residues involved in host range determination ***according to data of panel 3j-i and Extended Data Fig.6*** were marked with red dots. ***Red boxes: predicted hot-spot regions contributing to host range determination.***

Note on both structural sections; can you ensure it is clear which amino acid residues you are numbering in the different sections? I presume in the "Ortholog specificity and host tropism determinants" it is always the human aa residue number you are highlighting whereas it is or possibly should be the hedgehog, i.e. E. amu residue in the "Structure

basis of E.amu APN recognition by EriCoV" section.

Response: We thank the reviewer for pointing out this important issue and apologize for previous ambiguity. In the revised manuscript, we have standardized residue annotation across all structural sections and figures to ensure clarity and consistency.

Specifically, we now explicitly indicate the species origin of each residue using subscript notation directly following the amino acid and position (e.g., $N319_{hAPN}$ and $T_{E.amu}317D_{F.cat}$). This notation is applied consistently throughout the throughout both the "Ortholog specificity and host tropism determinants" and "Structural basis of *E.amu* APN recognition" sections and in all related figures.

This revision ensures that residue numbering is unambiguous and directly comparable across different sections.

Note: relevant residue numbering were updated as described.

Structure basis of E.amu APN recognition by EriCoV

Pretty straightforward structural analysis chapter. Similar to my previous note on figure 3, it would be good to also see western blots on the mutant protein expression.

Response: We have expanded the mutagenesis analysis in **Fig. 4h** and added western blot validation for the mutants in the structural section, as suggested (revised **Fig. 4i**).

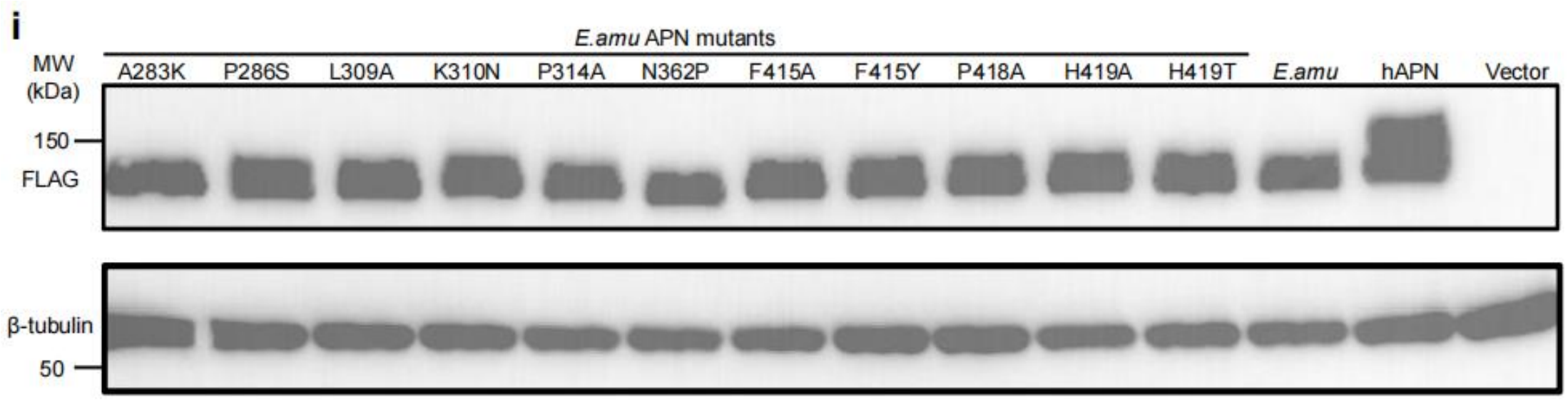

Fig. 4i | Expression efficiency of *E.amu* APN mutants. Western blot analysis and *E.amu* APN mutants (FLAG) expression levels in HEK293T cells. β-tubulin served as a loading control.

For the experiment relating to figure 4h, I refer back to multiple ones of my previous comments; this section remains hard to follow since the explanation and choice of residues related to. Some of these residues are now highlighted in figure 4b, which makes the non-verbalised rationale clear now based on structural information. The slight issue here is that in figure 3k, residues are numbered with the human residue numbers whereas now the hedgehog residues are used. It may be good for the sequence alignment in 3k to display both so that the link to figure 4h would be clear. I would also suggest somewhere around line 360 to refer back to figure 3k and you must highlight that these aa changes were based on mutations to the human aa residue in that alignment position. Easiest if referring to figure 3k.

Response: We thank the reviewer for this constructive suggestion and agree that clearer

linkage between the sequence analysis, structural mapping, and mutational validation is essential for readability.

To address this, we have revised **Fig. 3k** (see above) to improve residue annotation and cross-referencing. Specifically, the alignment now displays residue numbering corresponding to each species, while we consistently adopt *E.amu* APN residue numbering in both Fig. 3k and Fig. 4h to align with the structural analysis. This enable more direct comparison between the sequence variation and structurally defined interface residues. In addition, we now explicitly refer back to Fig. 3k in the Results section to make the rationale easy to follow.

In addition, we have expanded the mutagenesis analysis in **Fig. 4h** to include all key *E.amu* APN residues identified at the RBD interface in Fig. 4b. Mutations at several of these positions significantly reduce binding and pseudovirus entry, providing direct functional validation of the structural predictions.

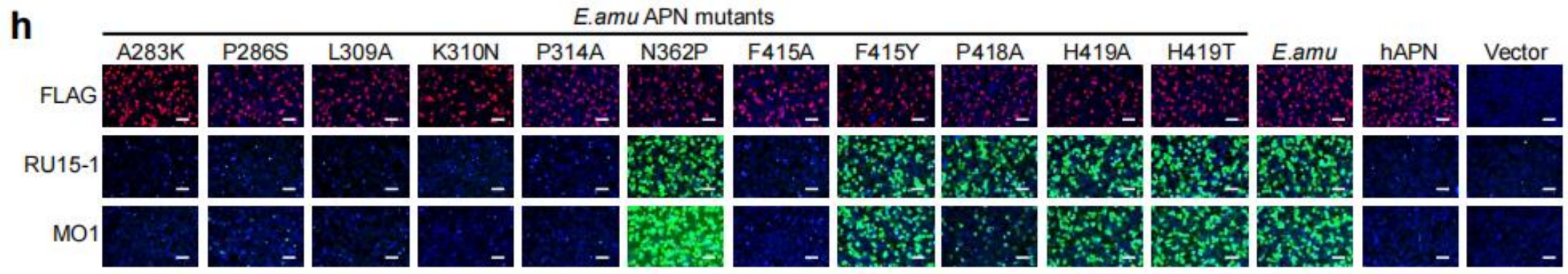

Fig. 4h | Binding efficiency of EriCoV RBD to *E.amu* APN mutants. RU15-1 and MO1 RBD–hFc binding (green) to HEK293T cells transiently expressing wild-type or mutant *E.amu* APN, assessed by immunofluorescence using an anti-hFc antibody. Scale bars: 100 μm.

Page 16, lines 392–395:

Updates:

residues identified as ***potential*** host range determination ***(Fig. 3k)*** are situated in or around the RU15-1 RBD footprint, with some of them directly contributing to the RBD interaction, such as A283$_{E.amuAPN}$, P286$_{E.amuAPN}$, and K310$_{E.amuAPN}$ (corresponding to N319$_{hAPN}$) (***Fig. 4g***)

EriCoV spike proteolytic activation

I will repeat myself further down in this section as I have only just added this general note. However, the authors have put a very generic title on this section yet have done a fraction of what they promise. What they have primarily looked at is functional potential of TMPRSS2 in EriCoVs but not broader proteolytic activation studies. If one looks at proteolytic activation in MERS-CoV, the virus is very amenable to a broad range of proteolytic activation (10.1073/pnas.1407087111). For a thorough study, and what is really warranted her, the authors need to look at a broader spectrum of proteolytic activation. They already have all the general assays in place; it would really only take an expansion of figure 5a, or broader fusion assay tests. I think this section is lacking and needs extension beyond TMPRSS2. Furthermore, the reasonably marginal effect that TMPRSS2 produces does not warrant the absolute statement of TMPRSS2 needs in the abstract and needs to be tuned down or extended once further proteolytic activation studies have been performed.

Response: We thank the reviewer for these important and constructive comments and acknowledge that the original proteolytic activation analysis was limited and overly focused on TMPRSS2. We agree with the reviewer that coronaviruses can employ multiple, context-dependent strategies for proteolytic activation, involving extracellular, plasma membrane-associated, and endosomal proteases.

In the revised manuscript, we have substantially expanded this section to include a broader panel of exogenous proteases (trypsin, TPCK-treated trypsin, pepsin, plasmin, elastase, and factor Xa), as well as representative cellular proteases (TMPRSS2, Cathepsin, and furin), using complementary biochemical, pseudovirus entry, fusion, and inhibitor-based assays (**Fig. 5** and **Supplementary Fig.10**). These experiments collectively provide a more comprehensive assessment of protease usage.

The title has been changed from the more general “EriCoV spike proteolytic activation” to “Proteases facilitate EriCoV S-mediated entry” to better reflect the scope of the data. Consistent with the reviewer’s concern, we have carefully tempered the interpretation of TMPRSS2 throughout the Abstract and Results, and now present it as a context-dependent, accessory factor rather than a dominant or required activator.

Line 413: The facilitation of S protein activation is not always merely a facilitator for S protein activation but can also be, for many coronaviruses, be a requirement determined by the macroenvironment for tropism of infection and stability. Many of the enteric tropism coronaviruses rely on the stability from a non or not completely proteolytically processed spike protein. Activation by macroenvironment proteases, such as pepsin or trypsin in the small intestine, or microenvironment proteases in the endocytic vesicular system are crucial for successful infection of many coronaviruses. For all intents and purposes, what we know about merbecoviruses is that they are enteric in hedgehogs so this is important to remember when discussing proteolytic processing.

Response: We fully agree with the reviewer's point of view regarding proteolytic activation. In the revised manuscript, we explicitly incorporate the distinction between macroenvironmental proteases (e.g., intestinal trypsin) and microenvironmental proteases (e.g., TMPRSS2, cathepsins), and discuss how these shape viral entry, stability, and tissue tropism.

Updates in Result section

Page 19, lines 454–457:

From: As indicated by our earlier pseudovirus entry data (e.g., Fig. 2i), EriCoV pseudovirus entry in HEK293T cells is highly dependent on trypsin treatment, a method commonly used to facilitate S protein activation via proteolytic cleavage.

To: Consistently, our initial pseudovirus assays showed that EriCoV entry and spike-mediated cell–cell fusion can be strongly enhanced by trypsin treatment (**Fig. 2h-i**), suggesting a potential role of extracellular soluble proteases.

Updates in Discussion section

Page 28, lines 763–765:

In particular, the strong dependence on trypsin-like proteases is consistent with an intestinal environment and supports a potential fecal–oral transmission route...

Lines 414ff: This comment links to the previous comment; the authors like to make the link back to another merbecovirus here – understandably. However, "inefficient" particle processing during particle biogenesis is completely normal for some coronaviruses. In particular the ones entering through an oro-fecal route, e.g. PEDV, are not proteolytically processed upon assembly and release. It is only upon trypsin treatment that PEDV for example gets processed (e.g. 10.1128/JVI.00297-14). Just as a cautionary sentence here as the focus seems to be on plasma membrane mediated fusion for this entire section whilst working with a) a pseudovirus only and b) in a very artificial cell and macroenvironment system for this virus.

Response: We thank the reviewer for this important clarification and fully agree with this point. We have substantially revised this section in the new submission, in which we have corrected our wording to acknowledge that incomplete or inefficient spike processing during particle biogenesis is a common feature of several enteric coronaviruses. We now explicitly discuss examples such as PEDV and related viruses, where spike proteins remain largely uncleaved upon release and require proteolytic activation by environmental proteases (e.g., trypsin) during entry.

We also appreciate the reviewer's concern regarding the original emphasis on plasma membrane-mediated fusion in the context of an over-expressing or VSV-based pseudovirus system. In response, we have revised this section to more clearly reflect a multi-step and context-dependent model of proteolytic activation. Specifically, we now distinguish between (i) pre-activation during biosynthesis or by macroenvironmental proteases prior to or during receptor engagement, (ii) activation at the plasma membrane (as captured in cell-cell fusion assays), and (iii) post-endocytic activation mediated by endosomal proteases (unable to be detected by the cell-cell fusion assay).

Furthermore, we explicitly acknowledge the limitations of the pseudovirus system and the artificial cellular and proteolytic environment used here, and have tempered our interpretation accordingly.

Updates in Result section

Page 19, lines 452–454:

From: Analysis of S protein incorporation in selected EriCoV pseudovirus particles revealed inefficient proteolytic processing during particle biogenesis, similar to what has been reported for NeoCoV (Fig. 5a).

To: Analysis of the EriCoV spike in pseudovirus particles revealed inefficient spike

processing at S1/S2 during particle biogenesis (Extended Data Fig. 10a), a feature common to enteric coronaviruses lacking an active furin cleavage site.

Updates in Result section
Page 20, lines 523–526:
Notably, given the use of a VSV-based pseudovirus system rather than the authentic viruses with potentially different preference of entry route, the relative contribution of these proteases should be interpreted with caution.

Updates in Discussion section
Page 28, lines 759–761:
Importantly, these results should be interpreted in light of the experimental systems used, including VSV-based pseudoviruses and overexpression models, which may not fully recapitulate authentic infection.

More changes could be found in the updated result section.

Line 424: I would reword that to: "functions as an accessory factor only to the APN receptor."

Response: We have revised the wording as suggested, thanks.

Figure 5 and line 427ff: I understand the general rationale the authors employ here basically using trypsin as a generic proteolytic activation tool to enhance entry of this specific coronavirus. What seems to be neglected in the general context is that trypsin may very well be the true protease that may activate EriCoVs in vivo. They do their best to find alternative proteases but for example, don't show what happens to EriCoV spikes upon protease treatment in figure 5a. To be truly comprehensive looking at what the section title suggests I would urge the authors to perform proteolytic activation assays with common proteases including cathepsins, trypsin, pepsin, plasmin, PC1, Xa, etc. Think about papers like the one by Millet & Whittaker on MERS (10.1073/pnas.1407087111) and 10.1038/s41586-025-09155-z, extended data figure 5.

Please also bare in mind that for some of the enteric coronaviruses trypsin does not merely function as a pre-activator but viruses very much require presence of trypsin during entry (e.g. PEDV, 10.1128/JVI.00297-14). Please caveat this in the biological context also in relation to low level infection that is observed.

Response: We thank the reviewer for this important and insightful comment and agree that in the original manuscript, trypsin was primarily used as a generic activation tool without sufficiently considering its potential physiological relevance, and that the analysis of proteolytic activation was not sufficiently comprehensive.

In the revised manuscript, we have substantially expanded this analysis. we now include a

broader set of proteolytic activation assays, including biochemical (western blot–based spike processing), membrane fusion, pseudovirus entry, and rcVSV-S propagation assays, using an extended panel of proteases. These include trypsin and TPCK-treated trypsin, as well as pepsin, plasmin, elastase, and factor Xa, in addition to endosomal proteases (e.g., Cathepsin L) and membrane-associated proteases (**Fig. 5** and **Extended Data Fig. 10**).

Across these assays, trypsin consistently shows the most robust effect on spike processing and viral entry, supporting the reviewer's suggestion that it may represent a physiologically relevant activating protease for EriCoVs, particularly in an enteric context. We have therefore revised the text to more explicitly acknowledge this possibility and to position trypsin not merely as an experimental tool, but as a plausible contributor to *in vivo* activation.

Together, these additions provide a more comprehensive assessment of proteolytic activation and align the scope of the analysis more closely with the reviewer' s suggestions.

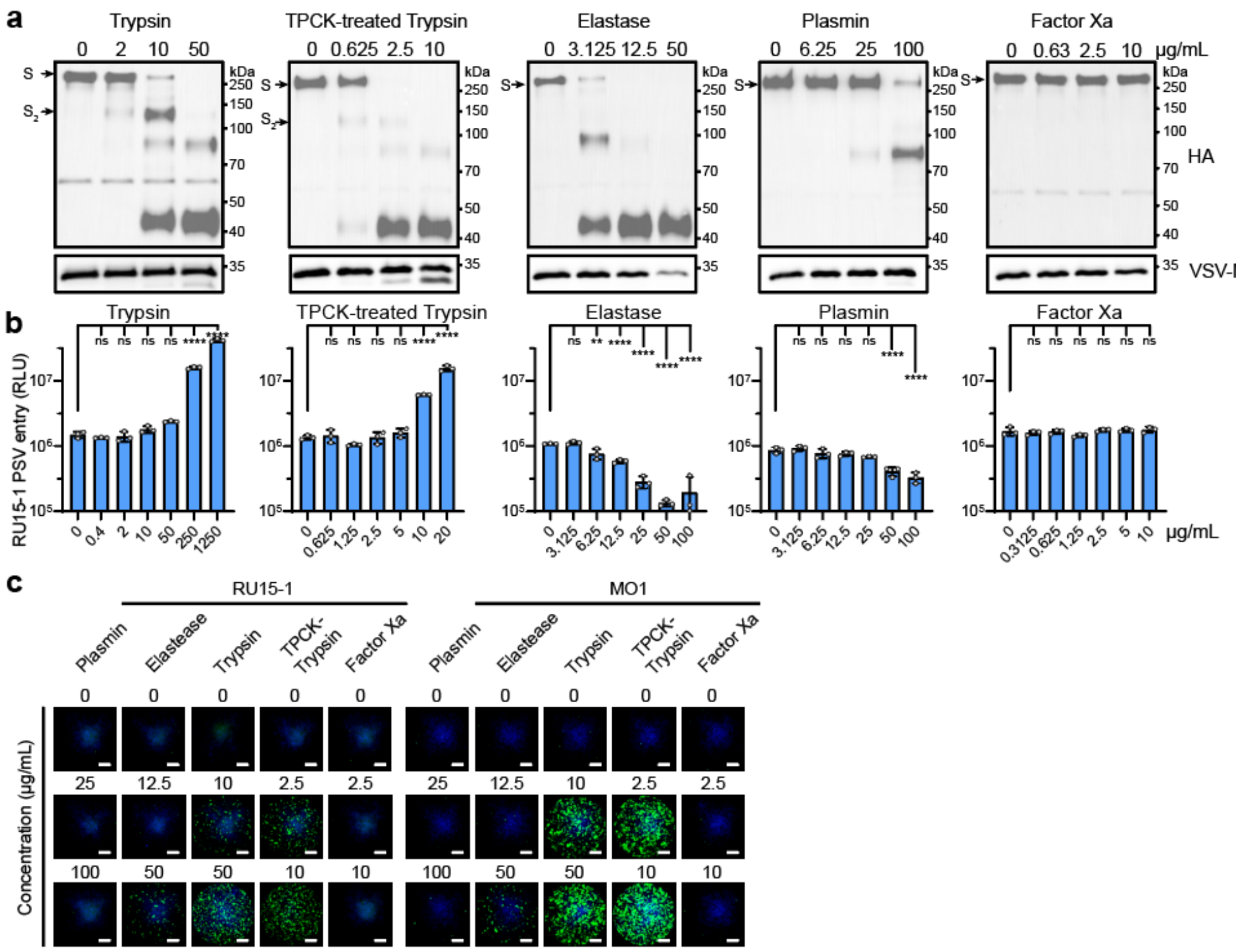

Fig. 5a-c | Functional characterization of protease-dependent EriCoV spike activation. **(a)** Detection of EriCoV S glycoprotein cleavage via Western blot using an anti-HA antibody targeting the C-terminally fused HA tag. Concentrated pseudoviruses were treated with indicated concentrations of exogenous proteases at 37 °C for 1 hour. VSV-M served as a loading control. **(b)** VSV-based pseudovirus entry of RU15-1 in HEK293T transiently expressing *E.amu* APN. Pseudoviruses were pretreated with different exogenous proteases at 37 °C for 1 hour. The predicted molecular weight of full-length Spike and the S2 corresponding to S1/S2 junction cleavage are indicated. **(c)** Cell-cell membrane fusion mediated by RU15-1-S and MO1-S in

HEK293T transiently expressing *E.amu* APN treated with indicated concentrations of exogenous proteases at 37 °C for 30 minutes. Statistical significance was determined by one-way ANOVA followed by Dunnett' s test (n = 3 independently infected wells) for panel b. *: $P<0.05$, **: $P<0.01$, ***: $P<0.005$, and ****: $P<0.001$, NS: not significant. RLU: relative light units. Scale bars: 5 mm.

Updates in Result section

Page 20, lines 520–523:

Taken together, these results support a model in which EriCoV spike proteolytic activation can be achieved through multiple pathways. Macroenvironmental proteases such as trypsin provide the most efficient activation, whereas cellular proteases, including TMPRSS2 and endosomal cathepsins, contribute in a context-dependent and strain-specific manner.

Updates in Discussion section

Page 28, lines 751–753:

Efficient infection in our experimental systems is strongly enhanced by trypsin, suggesting that trypsin-like macroenvironmental proteases may play a major role in spike activation.

Lines 757–759:

These findings are consistent with observations in other coronaviruses, especially those with enteric tropism, where proteolytic activation can occur through extracellular, cell surface, or endosomal routes.

Line 432fff: E64d is a cys protease inhibitor and whilst it inhibits cathepsins, it also inhibits a broad array of other cellular proteases. Please clarify this here – e.g. the cys-protease inhibitor E64d, used here as a proxy to assess potential Cathepsin involvement. Similarly, Camostat inhibits a variety of serine proteases, including TMPRSS2, but also trypsin, plasmin, Xa, and others. These are not specific assays and must be caveated as such. If a broader proteolytic activation panel would be performed for 5a, then maybe broader conclusions could be reached here. Camostat is also cell permeable so it cannot be assumed it only acts on surface proteases.

Furthermore, maybe expand that EK1C4 (eventually in line 453) is really a lipopeptide fusion inhibitor that can act on PM and endocytic fusion inhibition.

Response: We thank the reviewer for this important and technically insightful comment and fully agree that the inhibitors used in the original analysis are not protease-specific and require careful interpretation.

In the revised manuscript, we have explicitly clarified these limitations. Specifically, E64d is now described as a broad-spectrum cysteine protease inhibitor used as a proxy for cathepsin activity; camostat is noted to inhibit multiple serine proteases (including but not limited to TMPRSS2) ; and EK1C4 is described as a lipopeptide fusion inhibitor acting at both plasma membrane and endosomal entry steps.

To strengthen the analysis, we have conducted experiments with additional inhibitors with

higher selectivity, including inhibitors targeting TMPRSS2 (MM3122), or cathepsinB (CA-074) , cathepsin L (Z-FY-CHO) , with furin inhibitors (MI-1851) as a negative control. The results obtained with these reagents are consistent with the trends observed using E64d and camostat.

Importantly, in light of these considerations, we have revised the text to avoid overinterpretation of TMPRSS2 and highlight endosomal cathepsins are also important contributors for cellular EriCoV spike activation.

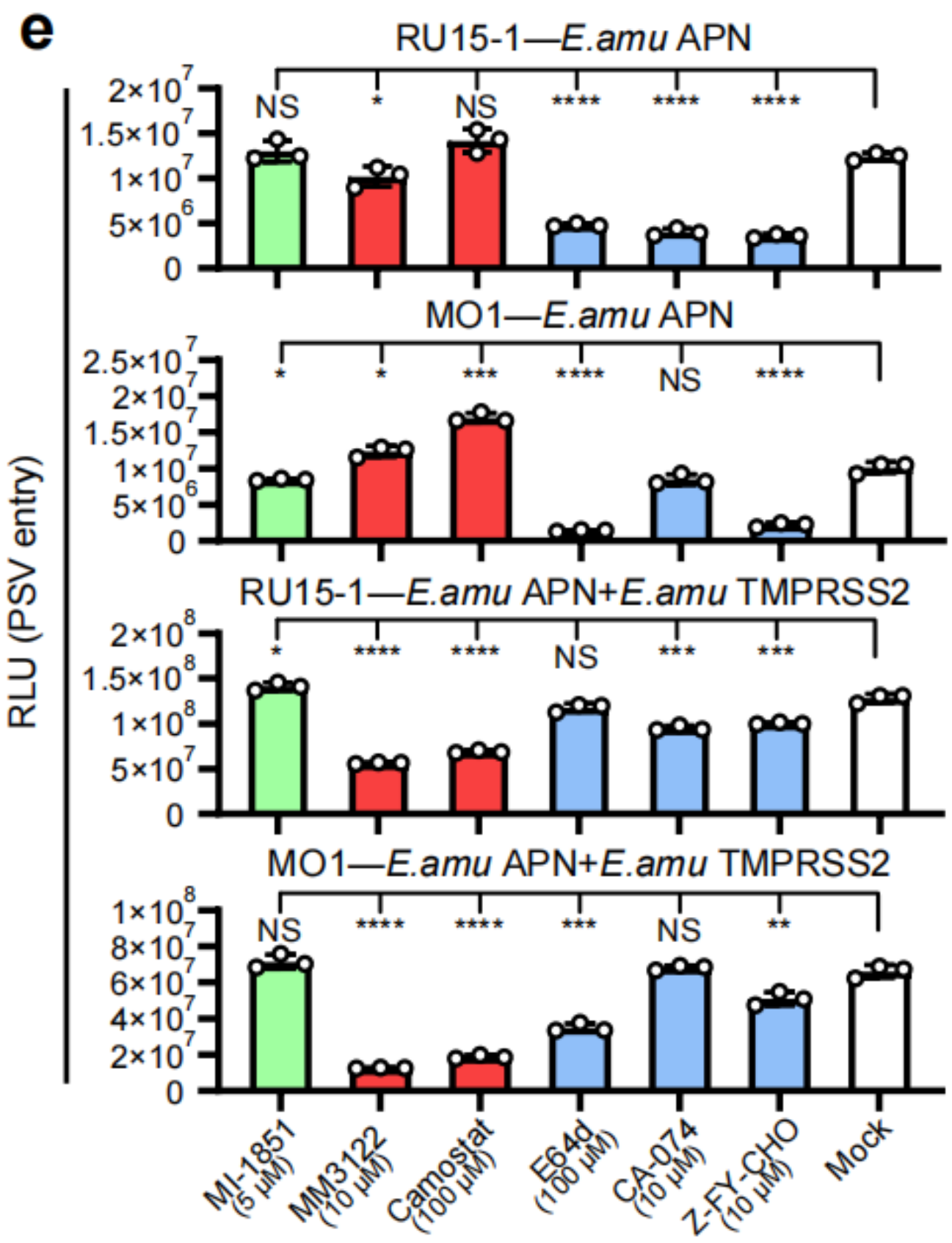

Fig. 5e | Protease inhibitor effects on EriCoV pseudovirus entry with or without TMPRSS2. RU15-1 and MO1 pseudovirus entry in HEK293T transiently expressing *E.amu* APN alone or co-expressing *E.amu* APN and *E.amu* TMPRSS2 in the presence of indicated protease inhibitors during infection. Statistical significance was determined by unpaired two-tailed *t* tests (n = 3 independently infected wells). *: P<0.05, **: P<0.01, ***: P<0.005, and ****: P<0.001, NS: not significant. RLU: relative light units. PSV: pseudovirus.

Updates in Result section

Page 19, lines 481–486:

From: Protease inhibition assay indicates that EriCoV entry can be inhibited by ***cathepsin B/L inhibitor E64d*** in 293T cells, but also becomes sensitive to ***TMPRSS2 inhibitor Camostat*** once E.amu TMPRSS2 is co-expressed.

To: Pseudovirus entry in HEK293T-*E.amu* APN cells was sensitive to ***the cysteine***

protease inhibitor E64d (used here as a proxy for cathepsin activity)...entry became partially sensitive to **Camostat (a broad-spectrum serine protease inhibitor)** and to...

Page 20, lines 516–518:

From: As expected, the OC43 HR2-derived pan-coronavirus fusion inhibitor EK1C4 peptide potently suppressed the viral amplification under the tested conditions.

To: As expected, the OC43 HR2-derived pan-coronavirus fusion inhibitor EK1C4 peptide***, which can act on both plasma membrane and endocytic fusion,*** potently suppressed the viral amplification under the tested conditions.

Page 20, lines 514–516:

These data indicate that while TMPRSS2 can facilitate cell-surface fusion under certain conditions, endosomal cathepsins remain important contributors to EriCoV spike activation.

Line 438: Any rationale why the authors now moved to CaCo-2 cells? Human enteric cell line? (also not a fully differentiated cell line so it can be polymorphic).

Response: We thank the reviewer for this important question and we are sorry for the confusion. Caco-2 cells were selected here for the following reasons:

(1) Caco-2 is a human colorectal epithelial cell line and therefore provides a more biologically relevant model for a potential enteric infection context.

(2) In contrast to HEK293T cells, Caco-2 cells endogenous expressing human TMPRSS2, enabling assessment of protease contribution under more physiological conditions.

(3) Caco-2 cells support efficient propagation of rcVSV-CoV-S and can be stably maintained over multiple days, which is important for multi-round amplification assays.

(4) They form tight cell–cell contacts, making them suitable for cell–cell fusion analyses; given their relatively low transfection efficiency, stable expression (lenti-virus based transduction) systems are more appropriate in this context.

(5) Caco-2 cells are commonly used in coronavirus research, facilitating comparison with prior studies.

In the revised manuscript, we have mentioned the rationale for using Caco-2 cells in this set of experiments.

Updates in result section

Page 19, lines 490–493:

To extend these findings to ***multi-round infection***, we generated replication-competent VSV (rcVSV) encoding the S glycoproteins of IT5-12, RU15-1, MO1, or cw_6 and evaluated its amplification ***in more biologically-relevant human colorectal cell line Caco-2 cell with endogenous TMPRSS2 expression***.

Also please specify that “can be boosted with trypsin pre-treatment of the pseudovirus”

unless in this case it is treatment during infection?

Response: We have specified that entry enhancement was achieved by treatment during infection. We have clarify it here and other places regarding the conditions of trypsin treatment in the figure legends and result section.

Updates in result section

Page 20, lines 494–495:

From: Viral amplification was observed in Caco-2 cells expressing *E.amu* APN and can be boosted with trypsin treatment (Extended Data Fig. 8c).

To: viral amplification was readily detected and could be substantially enhanced when trypsin was maintained in the culture medium during propagation (Extended Data Fig. 10e).

Updates in legend of (Extended Data Fig. 10e)

Page 67, lines 1779–1781:

Propagation of...in DMEM supplemented with 2% FBS, with or without 20 μg/mL TPCK-treated trypsin.

Figure 5f, g and associated results section 436 fff: I do only partially agree with the author's interpretation here. The fact that trypsin treatment boosts infection (significantly) and TMPRSS2 presence at the surface boosts infection slightly – 3-5x – does not support a strong involvement of TMPRSS2. In my opinion, it supports a conclusion that proteolytic activation upon receptor binding may be required for successful infection. A role that can be partially met by an OVEREXPRESSION of TMPRSS2 but may also be met by a macroenvironmental protease in vivo (i.e. trypsin, pepsin, etc). The only thing I conclude from the experiment in figure 5 is that TMPRSS2 is marginally enhancing infection here but that other proteases, similar to MERS but also similar to other (possibly enteric) coronaviruses, are playing a role as the E64d treatment and camostat treatment show. Namely, in the absence of TMPRSS2 overexpression (figure 5g), infection is far stronger with Camostat treatment than it ever is in the presence of TMPRSS2 with Camostat treatment. This actually indicates that TMPRSS2 may be having negative effects on infection rather than aiding it. And that surface fusion is possibly not a successful infection route for the pseudovirus. But please bare in mind this is a system based on VSV, which generally goes through clathrin-mediated endocytic uptake and this may not be as relevant for the actual EriCoV.

Response: We thank the reviewer for this insightful and careful interpretation of our data. Our current data do not support a dominant role for TMPRSS2 in EriCoV entry, but instead indicate that proteolytic activation is required and can be mediated by multiple proteases depending on the context and strain used.

Consistent with this view, our expanded analyses show that exogenous proteases such as trypsin produce a substantially stronger enhancement of spike activation and viral entry than TMPRSS2 overexpression, suggesting that macroenvironmental proteases are likely

more relevant *in vivo*, particularly in an enteric setting (**Fig. 5a-c**). In contrast, TMPRSS2 provides only a modest, though reproducible, enhancement and appears to act in a strain-specific (e.g., preferred by Asian strains MO1 and cw_6) and context-dependent manner (**Fig.5d and 5f**).

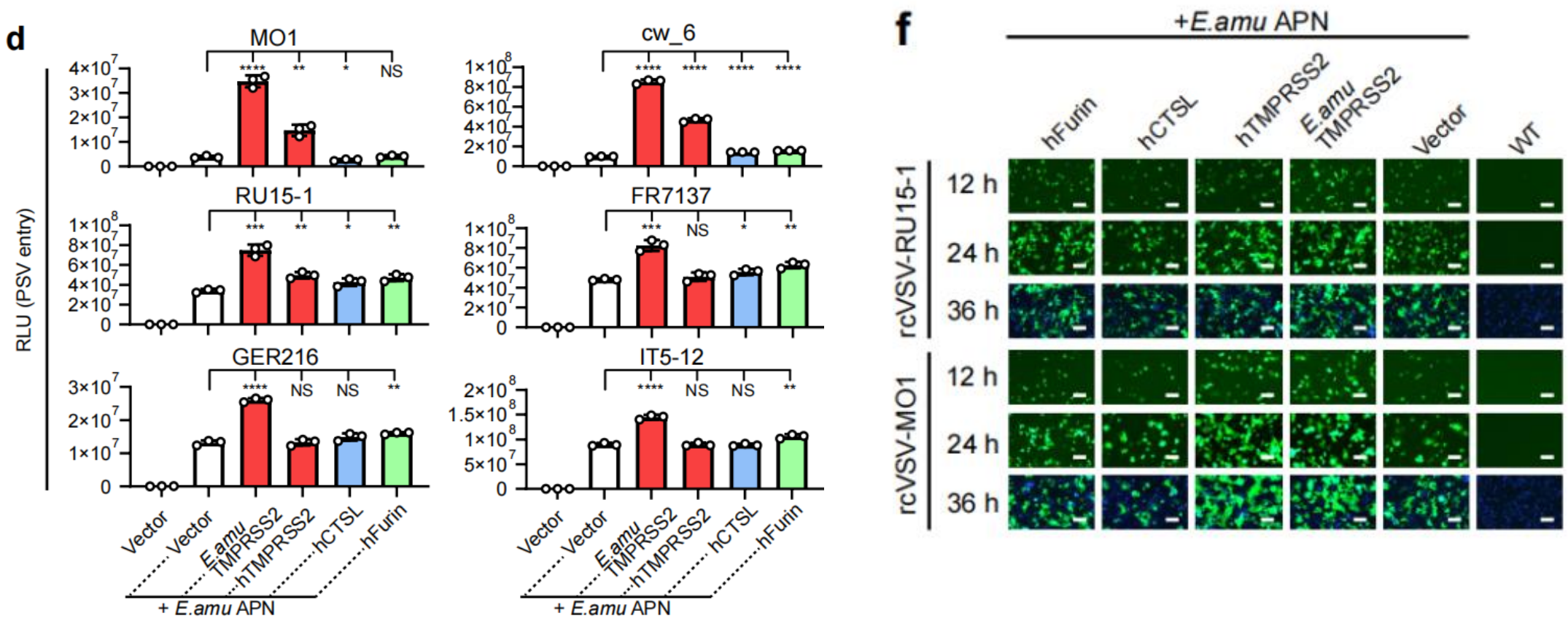

Fig. 5d,f | Pseudovirus entry or rcVSV propagation of EriCoV in HEK293T or Caco-2 expressing *E.amu* APN and endogenous proteases. (**d**) Entry efficiency of selected EriCoV pseudoviruses in HEK293T cells transiently expressing *E.amu* APN alone or co-expressing *E.amu* APN and indicated cellular proteases, including human furin (hFurin), human cathepsin L (hCTSL), human TMPRSS2 (hTMPRSS2), and hedgehog TMPRSS2 (*E.amu* TMPRSS2). Pseudoviruses were pretreated with 1.25 mg/mL trypsin. (**f**) Propagation of rcVSV-EriCoV-S in Caco-2-*E.amu* APN stably expressing the indicated proteases. Statistical significance was determined by unpaired two-tailed *t* tests (n = 3 independently infected wells). *: $P<0.05$, **: $P<0.01$, ***: $P<0.005$, and ****: $P<0.001$, NS: not significant. RLU: relative light units. PSV: pseudovirus. Scale bars: 100 µm.

We also appreciate the reviewer's observation regarding the camostat-treated conditions. To address this, we repeated the experiments using independently generated cell lines and additional controls. The apparent discrepancy was attributable to differences in cell background during stable cell line selection, rather than a negative effect of TMPRSS2. When compared within matched cellular systems, TMPRSS2 consistently confers a modest enhancement that can be inhibited by camostat or MM3122, supporting its role as a contributing factor (the revised **Fig.5g and Extended Data Fig. 10h**).

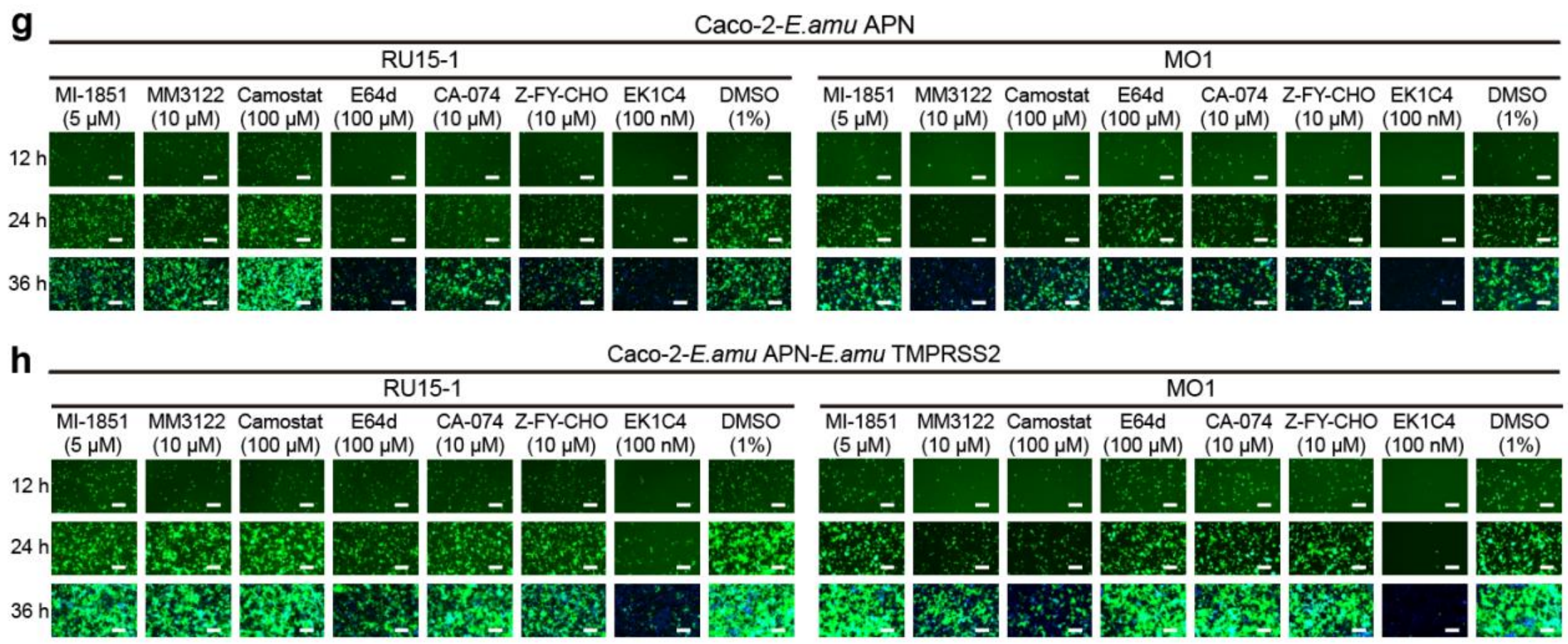

Fig. 5g and Extended Data Fig. 10h | Inhibition of protease inhibitors to rcVSV-EriCoV

propagation in Caco-2-*E.amu* APN or Caco-2-*E.amu* APN-*E.amu* TMPRSS2. Propagation of rcVSV-RU15-1 (left), and rc-VSV-MO1 (right) in Caco-2 cells stably coexpressing *E.amu* APN alone (Fig. 5g) or Caco-2 cells stably coexpressing *E.amu* APN and *E.amu* TMPRSS2 (Extended Data Fig.10h) in the presence of the indicated inhibitors during viral amplification without the presence of trypsin. Scale bars: 200 µm.

To further strengthen this conclusion, we expanded the analysis to include a broader panel of 20 different EriCoV strains. These experiments show that TMPRSS2 utilization is strain-dependent, with certain strains displaying a more pronounced sensitivity (**Extended Data Fig. 10c**).

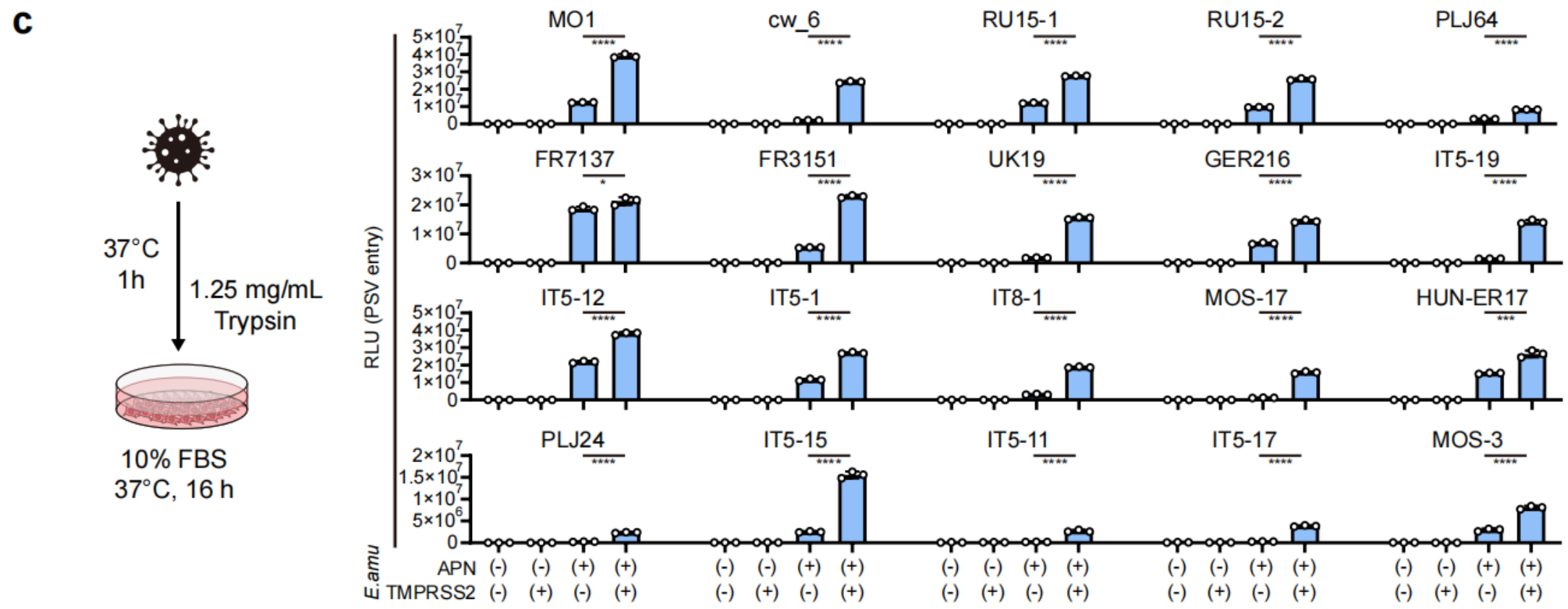

Extended Data Fig. 10c | *E.amu* TMPRSS2 boost effect across 20 EriCoV strains. Pseudoviruses entry efficiency of 20 EriCoV strains into HEK293T cells transiently expressing the *E.amu* APN, *E.amu* TMPRSS2, or both. PSVs were pretreated with 1.25 mg/mL trypsin, and infection was performed in the presence of 10% FBS. Data are presented as mean ± SD. Statistical significance was determined using unpaired two-tailed *t* tests (n = 3 independently infected wells). *: $P<0.05$, **: $P<0.01$, ***: $P<0.005$, ****: $P<0.001$, and NS: not significant. RLU: relative light units. PSV: pseudovirus.

Based on these results, we have revised the manuscript to provide a more balanced interpretation. We now conclude that proteolytic activation can be efficiently mediated by macroenvironmental proteases such as trypsin, and to a lesser extent by cellular proteases including TMPRSS2 or endosomal cathepsins depending on the cell background. We also clarify that TMPRSS2 likely acts as an accessory factor with potential relevance for tissue tropism determination, rather than a primary requirement for EriCoV entry.

Finally, we fully acknowledge the limitations that VSV-based pseudovirus system may have different features compared with authentic viruses, as highlighted by the reviewer, a limitation now acknowledged in the discussion section.

Result section:

Page 20, lines 520–526:

Taken together, these results support a model in which EriCoV spike proteolytic activation can be achieved through ***multiple pathways***. Macroenvironmental proteases such as trypsin provide the most efficient activation, whereas cellular proteases, including TMPRSS2 and endosomal cathepsins, contribute in a

context-dependent and strain-specific manner. Notably, given the use of a ***VSV-based pseudovirus*** system rather than the authentic viruses with potentially different preference of entry route, the relative contribution of these proteases should be ***interpreted with caution***.

Discussion section:

Page 28, lines 750–761:

In addition to receptor binding, our data indicate that proteolytic activation represents a critical determinant of EriCoV entry. Efficient infection in our experimental systems is strongly enhanced by trypsin, suggesting that ***trypsin-like macroenvironmental*** proteases may play a major role in spike activation. In addition, hedgehog TMPRSS2, and to a lesser extent the human version, provides a ***modest enhancement under overexpression conditions***, and its contribution appears to be strain-dependent. Endosomal cathepsins can also facilitate entry, particularly in the absence of surface proteases, indicating that EriCoVs can utilize multiple proteolytic pathways. These findings are consistent with observations in other coronaviruses, especially those with enteric tropism, where proteolytic activation can occur through ***extracellular, cell surface, or endosomal routes***. Importantly, these results should be interpreted in light of the experimental systems used, including VSV-based pseudoviruses and overexpression models, which may ***not fully recapitulate authentic infection***.

Neutralizing antibodies against EriCoV

I don't have much to say on this experiment. Monoclonal antibody therapies have shown limited long-term efficacy in preventing spread but can aid individual patients and could possibly help in a small localised outbreak scenario.

Response: We thank the reviewer for the comment on the antibody section and are pleased that the significance of our work is recognized.

Discussion

The discussion section is short and I would like to see a bit more discussion of the risk of cross-species spillover. Discussion of the binding pocket and what the risks are of spillover in marginally susceptible species, such as felines and rats. As highlighted earlier in the respective section, it is a shame that the authors did not assess the susceptibility of natural predators of hedgehogs, such as badgers in the biological context.

Similarly, once proteolytic activation has been studied a bit more broadly, I would like to see some discussion on the route of transmission and infection (based on the biological

Response: We have substantially expanded the Discussion to address cross-species spillover risk, including structural constraints at the receptor interface, susceptibility of marginal hosts, and ecological considerations such as species interactions and transmission routes. We have also integrated the expanded proteolytic activation data (see above) into a broader discussion of infection route and tissue tropism.

Discussion: cross-species spillover risk

This restricted tropism is governed by a small number of residues within APN domain 2, including positions that affect ***steric compatibility and glycosylation***. This suggests that, under current conditions, EriCoVs face a ***substantial barrier*** to cross-species transmission. While current data suggest a relatively low risk though the ***natural predators*** of hedgehogs, such as badgers, that we tested were resistant to EriCoV recognition, the detectable, albeit weak, compatibility with ***felid APN*** of several European strains highlights that relatively ***minor changes at the receptor-binding interface, either in APN or RBD, could lower the host barrier*** in certain species. Given the close association between humans and domestic cats, ***continued surveillance of feline populations*** may be warranted. In addition, there might be other ***species ecologically related to hedgehogs*** that could be monitored for spillover events, especially those ***without the restrictive N319$_{hAPN}$ glycosylation*** in the interaction interface, which theoretically display a lower host range barrier.

Discussion: route of transmission and infection

In vivo, protease availability is likely shaped by tissue environment, suggesting that protease usage may vary with ***transmission route and host species***. In particular, the strong dependence on trypsin-like proteases is consistent with an ***intestinal environment*** and supports a ***potential fecal–oral transmission route***, whereas adaptation to respiratory proteases such as TMPRSS2 could represent a ***facilitating factor*** for ***tissue tropism shifts/expansions*** and affect ***pathogenicity***, as is observed in Delta and Omicron variants of SARS-CoV-2.

Reviewer #3 (Remarks to the Author):

Liu et al. demonstrate that European and Asian hedgehog coronaviruses (EriCoVs) utilize hedgehog aminopeptidase N (APN) as a physiological receptor. Using a broad panel of EriCoV spike proteins and APN-expressing cells, combined with cryo-EM structural analysis, the authors provide a **comprehensive** study. These analyses are **excellent** and impressive, offering clear evidence for receptor characterization. However, I have the following comments to address.

Response: We thank the reviewer for the positive assessment of our study and for recognizing the strength of our combined functional and structural analyses. We have carefully considered the reviewer's comments and have revised the manuscript accordingly, as outlined in detail below.

Major Comments

1. The authors found that R. norvegicus (R.nor) APN did not support RU15-1 or MO1 binding (via Fc-fusion RBD proteins) and only exhibited detectable binding with the FR7137 RBD. However, the constructs for the Fc-fusion proteins appear inconsistent across species; some include the entire S1 domain, while others include only the RBD. The S1 domain alone can be particularly unstable, and the exposure of hydrophobic interfaces (originally the S2 interface) might facilitate non-specific binding. The authors should clearly state these differences in the expressed domains and discuss how this species specificity relates to amino acid substitutions in both the APNs and the RBD/S1 domains, based on their cryo-EM structures.

Response: We thank the reviewer for this important point. We agree that mixing S1-Fc and RBD-Fc constructs could introduce ambiguity, particularly due to potential non-specific interactions from exposed hydrophobic surfaces. We therefore clarify that all APN tropism analyses, including the *R.nor* results, were performed using RBD-Fc constructs across all EriCoV strains. The limited S1-Fc construct (IT5-19, UK19, FR7137, and FR3151) related data previously shown (in previous **Extended Data Fig. 7f-g**) were acquired to evaluate the impact of selected RBD polymorphisms, not for species specificity assessment, and have now been removed to ensure consistency. All conclusions are now supported ***exclusively by RBD-Fc-based*** assays and the corresponding text ***in the method section*** has been updated accordingly.

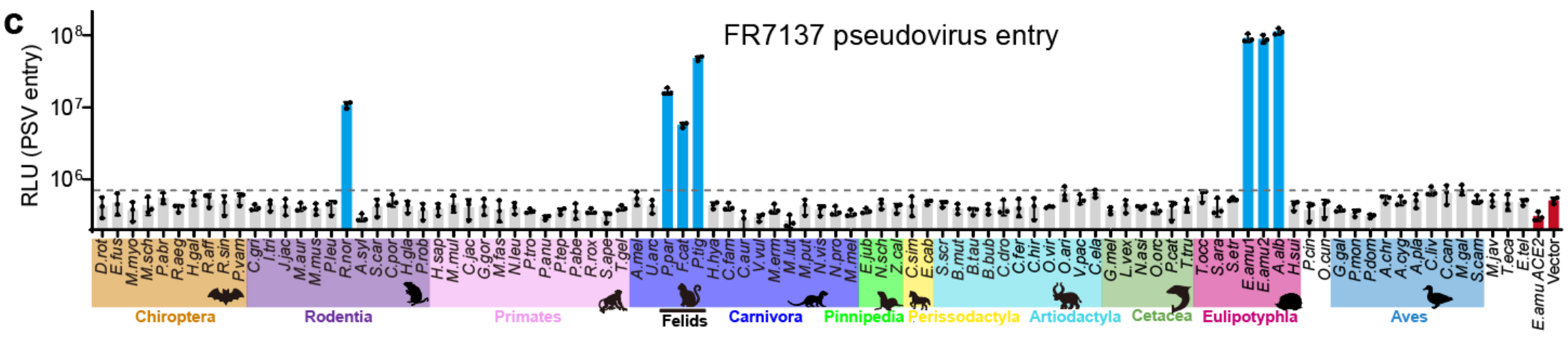

Fig. 3c | VSV-based pseudoviruses entry of FR7137 in HEK293T transiently expressing 93 APN orthologs. Data are presented as mean ± SD. RLU: relative light units. PSV: pseudovirus.

Regarding the observed strain-specific binding phenotypes, Importantly, we further validated the strain-specific receptor compatibility using pseudovirus entry assays, which confirmed that FR7137 can utilize R.nor APN, consistent with the RBD-Fc binding results (**new Fig. 3c**). We addressed the underlying mechanism by integrating our cryo-EM structural data with sequence analysis. Overall, species specificity is primarily determined by the degree of amino acid compatibility at the RBD-virus binding interface, particularly involving residues within APN domain 2 and the receptor-binding motif (RBM) of the RBD (**revised Fig.4g**).

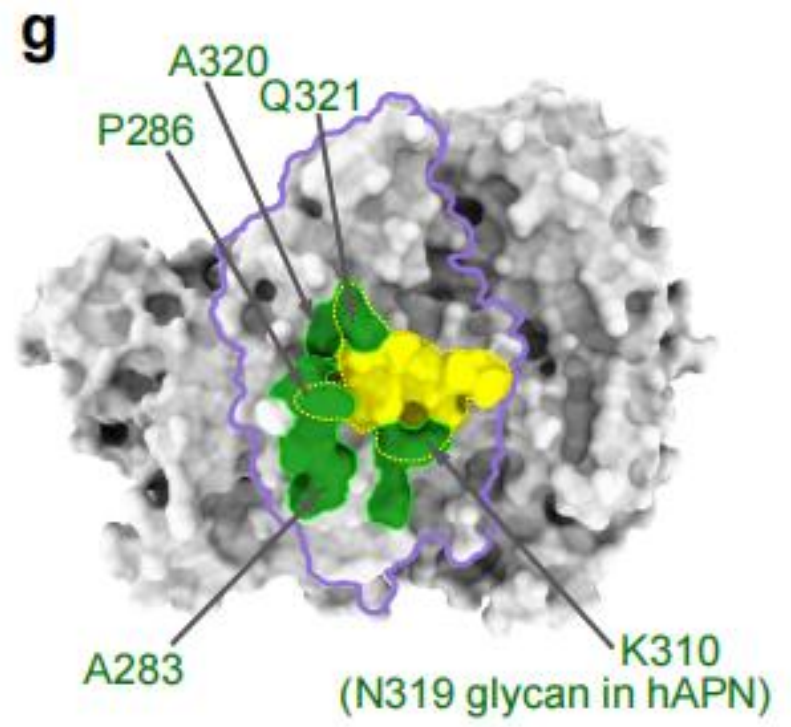

Fig. 4g | The overlap of binding interface and host range determinants in domain 2 of hedgehog APN. Summary of the host range determinants (green) identified in this study and RU15-1 RBD binding interface (yellow dashed line) mapped onto *E.amu* APN surface. Residue positions in secondary structures are indicated; domain 2 is outlined in purple.

That domain 2 of APN exhibits substantial sequence variability across species, leading to differences in residue positioning, electrostatic properties, and glycosylation patterns at the binding interface (**Fig. 3k**). For example, in hedgehog (*E.amu)* APN, residues H419 promotes a hydrophobic interaction interface with the RU15-1 RBD (**Fig. 4b**), whereas in *R.nor* APN, the corresponding residue is substituted with T, resulting in a weakened hydrophobic interaction. Furthermore, the N362 glycan in *E.amu* APN forms a stable hydrogen bond with G535 in the RU15-1 RBD (**Fig. 4b**), while in *R.nor* APN, this position is substituted with P, resulting in the loss of this interaction. These variation, along with other residue substitutions, collectively disrupt the receptor-virus interface, preventing *R.nor* APN from supporting RU15-1 binding (cf. **Reviewer Fig. R6** below).

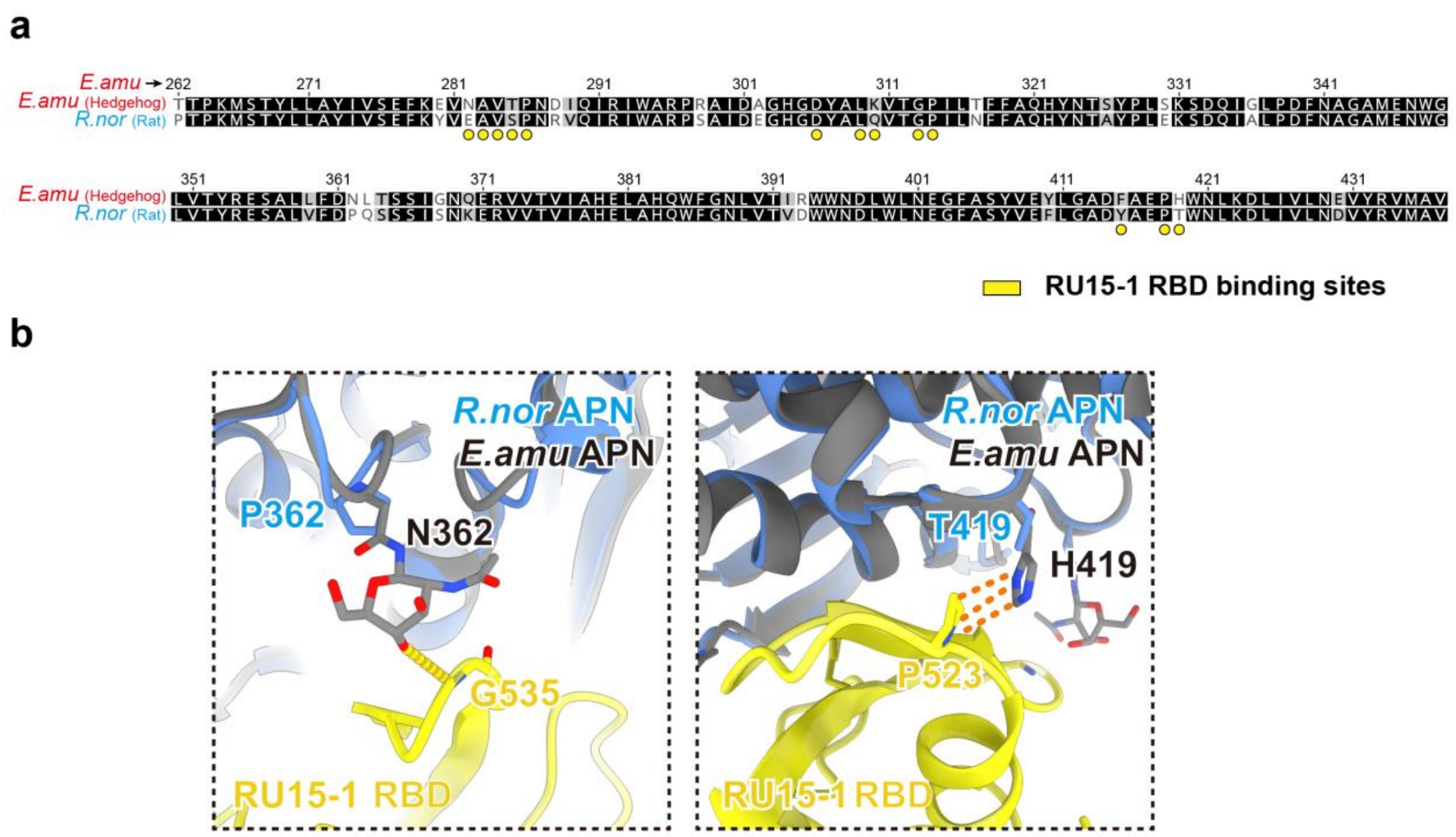

Reviewer Fig. R6 | Sequence and structural analysis of the interaction between RU15-1 and *E.amu* APN or *R.nor* APN. (a) The binding sites located within 4 Å of RU15-1 RBD bound to *E.amu* APN are indicated by yellow circles. (**b**) Comparison of the interaction interface between RU15-1 RBD and indicated APN orthologs. R.nor APN structure predicted by AlphaFold3 and superimposed with the RU15-1 RBD—E.amu APN complex. E.amu APN and R.norvegicus APN were colored grey and blue, respectively; RU15-1 RBD was shown in yellow. All structures were depicted in cartoon representation, with key residues highlighted as sticks. Hydrophobic interactions and hydrogen bonds are denoted by orange and yellow dashed lines, respectively.

On the viral side, the cryo-EM structure reveals that the RU15-1 RBD engages APN domain 2 through its RBM via a combination of hydrogen bonds and hydrophobic interactions (**Fig. 4b**). The conservation or variation of these interface residues among different viral strains further modulates receptor compatibility, which explains why FR7137 can sub-optimally engage *R.nor* APN (revised **Fig. 3b-d**).

Taken together, these results support a model in which EriCoV species specificity is governed by structural compatibility at the virus–receptor interface. Amino acid substitutions within APN domain 2 define receptor compatibility, while variation in RBD interface residues further tunes strain-specific receptor adaptation. This bidirectional molecular compatibility collectively shapes the host range of EriCoVs.

Discussion

Page 28, lines 728–736:

This restricted tropism is governed by ***a small number of residues within APN domain 2***, including positions that affect ***steric compatibility*** and ***glycosylation***. This suggests that, under current conditions, EriCoVs face a substantial barrier to cross-species transmission. While current data suggest a relatively low risk though the natural predators of hedgehogs, such as badgers, that we tested were resistant to EriCoV recognition, the detectable, albeit weak, compatibility with felid APN of several European strains highlights that relatively minor changes at the receptor-binding interface—either in APN or the viral RBD—could lower the host barrier.

2. Details regarding the recombinant Spike constructs used for cryo-EM are missing. The authors should clarify whether they introduced stabilizing mutations, such as prolines. If such mutations were used, they might account for the dominance of the “closed” conformation.

Response: We thank the reviewer for this important point. We have now clarified the details of the recombinant Spike constructs used for cryo-EM in the revised Methods section.

Methods

Page 43, lines 1214–1225:

For Cryo-EM and BLI assays, DNA sequences encoding E.amu APN protein (residues 60–960) and RU15-1 RBD (residues 366–574; GenBank: XCN28775.1) were obtained

from NCBI. An N-terminus CD5 signal peptide and a C-terminus His-tag were added to both constructs via PCR. The DNA sequence encoding the full-length RU15-1 Spike protein (residues 1–1262; GenBank: XCN28775.1) was obtained from NCBI. A C-terminal T4 fibritin trimerization motif (foldon), followed by an HRV 3C protease cleavage site and a Twin-Strep tag, was added via PCR. These constructs were cloned into the pCAGGS mammalian expression vectors.
Importantly, no stabilizing mutations (such as proline substitutions) were introduced in the Spike constructs. Therefore, the predominance of the closed conformation observed in our cryo-EM structure is unlikely to be an artifact of engineered stabilization, but rather reflects an intrinsic conformational property of the RU15-1 Spike under the experimental conditions.

3. The PPI2 prediction is neither cited nor fully described. It is unclear which tool was used and whether the prediction is reliable.
We thank the reviewer for pointing this out. We have now clarified the details of the PPI2 prediction in the Methods section of the revised manuscript, including the tool used, methodological description, and appropriate citations.

Citation:
Rodrigues, C.H.M., Myung, Y., Pires, D.E.V., & Ascher, D.B. mCSM-PPI2: predicting the effects of mutations on protein–protein interactions. Nucleic Acids Research ***47****, W338–W344 (2019).*

Importantly, mCSM-PPI2 is a well-established and widely used tool in the field, having been cited extensively (over 300 citations) and applied in diverse high-impact studies, including recent work in *Nature Communications* (2025), *Nature Medicine* (2023), and *Nature Reviews Cancer* (2025). These applications support the general reliability of the method for evaluating mutation effects on protein–protein interactions.

Methods
Page 52, lines 1630–1638:
Specifically, the PPI2 prediction was performed using the mCSM-PPI2 web server (https://biosig.lab.uq.edu.au/mcsm_ppi2/), which predicts the effects of mutations on protein–protein interactions based on graph-based structural signatures (Rodrigues et al., *Nucleic Acids Research*, 2019).The structure of the hAPN–RU15-1 complex was used as the template for all calculations after removal of solvent molecules and glycosylation site. In brief, the experimentally determined structure of the wild-type protein complex was used as input (in PDB format), and mutations were defined based on residue positions corresponding to the structural model. The predicted effects of mutations on binding affinity were evaluated in terms of changes in binding free energy (ΔΔG), as implemented in the mCSM-PPI2 framework.

Minor Comments
1. A figure showing an amino acid sequence comparison of APNs and EriCoVs, including

a binding counter/matrix, would greatly assist the reader's understanding.

Response: We thank the reviewer for this helpful suggestion. In response to your suggestion, along with a related comments by review 2, we have revised the **Fig.3k** to improve residue annotation. Specifically, the sequence alignment now displays residue numbering corresponding to each species, and we consistently adopt *E.amu* APN residue numbering in both **Fig. 3k** and **Fig. 4h** to align with the structural analysis. We believed the updated **Fig. 3k** would greatly improve the clarity and make the section easier to follow.

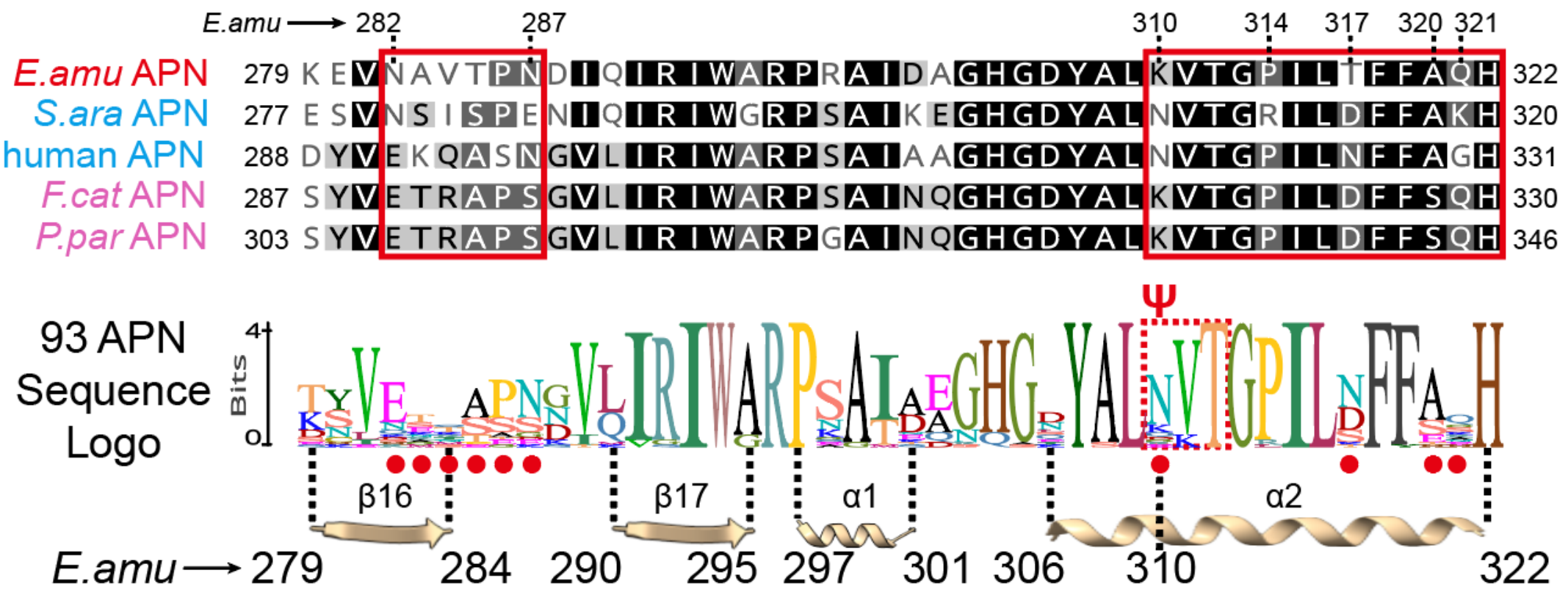

Fig. 3k | Sequence alignment of selected APN orthologs and logo plot displaying the polymorphisms across 93 APN orthologs. Sequence logo plot displaying the polymorphisms across APN orthologs. Key secondary structures and predicted residues involved in host range determination according to data of panel 3i-j and Extended Data Fig.6 were marked with red dots. Red boxes: predicted hot-spot regions contributing to host range determination. N319$_{hAPN}$ glycosylation sequon is indicated with red dashed box, and the modification site marked with Ψ.

2. Lines 255-256: The sentence "Substituting domain 2 of E. amu APN with its human counterpart abolished binding, while substituting E. amu Domain 2 with human APN counterpart restored binding..." is confusing. Please clarify the direction of these substitutions.

Response: Sorry for the confusing. We have revise the text to make it more easy to follow.

Page 11, lines 275–277:

From: Substituting domain 2 of *E. amu* APN with its human counterpart abolished binding, while substituting *E. amu* Domain 2 with human APN counterpart restored binding...

To: ***Replacement of*** domain 2 ***in*** *E.amu* APN with ***the*** human counterpart abolished binding, ***whereas reciprocal substitution of*** *E.amu* Domain 2 ***in*** human APN counterpart restored binding,

3. The inconsistent use of "hAPN/human APN" and "hTMPRSS2/human TMPRSS2" may be confusing. Since proteins from various species are compared throughout the manuscript, I suggest unifying the terminology to "human APN" and "human TMPRSS2" for clarity.

Response: We thank the reviewer for this helpful suggestion. We have standardized the terminology throughout the manuscript by replacing "hAPN" and "hTMPRSS2" with "human APN" and "human TMPRSS2" for improved clarity and consistency.

For conciseness and to maintain figure Readability, abbreviated forms are retained in certain contexts (e.g., subscripts, labels, and space-limited figure panels), where their meaning is clearly defined.

4. Lines 310-311: To avoid confusion with the "closed" state of the Spike protein (even though only the RBD was used), the phrase “...ensuring that all E. amu APN particles adopted a 'closed' conformation” should perhaps be revised to “...ensuring that all E. amu APN molecules in the particles adopted a 'closed' conformation.”
Response: We thank the reviewer for this helpful suggestion. We have revised the text accordingly to clarify that the “closed” conformation refers specifically to *E.amu* APN molecules to avoid potential confusion.

Page 15, lines 342–343:
From: ensuring that all *E.amu* APN particles adopted a “closed” conformation.
To: ensuring that all *E.amu* APN ***molecules in*** the particles adopted a “closed” conformation.

5. Figure 3g: Two shifted peaks are observed for WT E. amu APN with MO1-RBD, whereas only one is observed with RU15.1. The authors should provide a comment on this observation.
Response: We thank the reviewer for pointing out this observation. Upon re-examination, we found that the apparent difference in peak patterns is attributable to heterogeneity in transfection efficiency and the associated gating strategy used during flow cytometry analysis. Specifically, **variations in expression levels** led to differences in the distribution of APN-positive cell populations.

Importantly, this does not affect the overall interpretation, as the binding trend remains consistent with the conclusions drawn from other assays. However, we agree that this feature may cause confusion to the reader. In addition, as this data is partially redundant with the results shown in **Fig. 3f**, we have **removed** previous **Fig. 3g** from the revised manuscript to improve clarity and streamline data presentation.

6. Figure 4 (panels a, b, or c?), its legend, and the related text (lines 319-330): The authors should indicate or mention K310, which corresponds to the N-glycosylation site in human APN.
Response: We thank the reviewer for this helpful suggestion. We have now explicitly indicated residue $K310_{E.amuAPN}$ in **Fig. 4b**, its legend, and the corresponding text to improve clarity.

Inserted text:
Page 15, lines 357–359:
Notably, the key position of K310 in *E.amu* APN forms a canonical N-linked glycosylation site in human and many other species, which may introduce steric hindrance at the

virus-receptor interface.

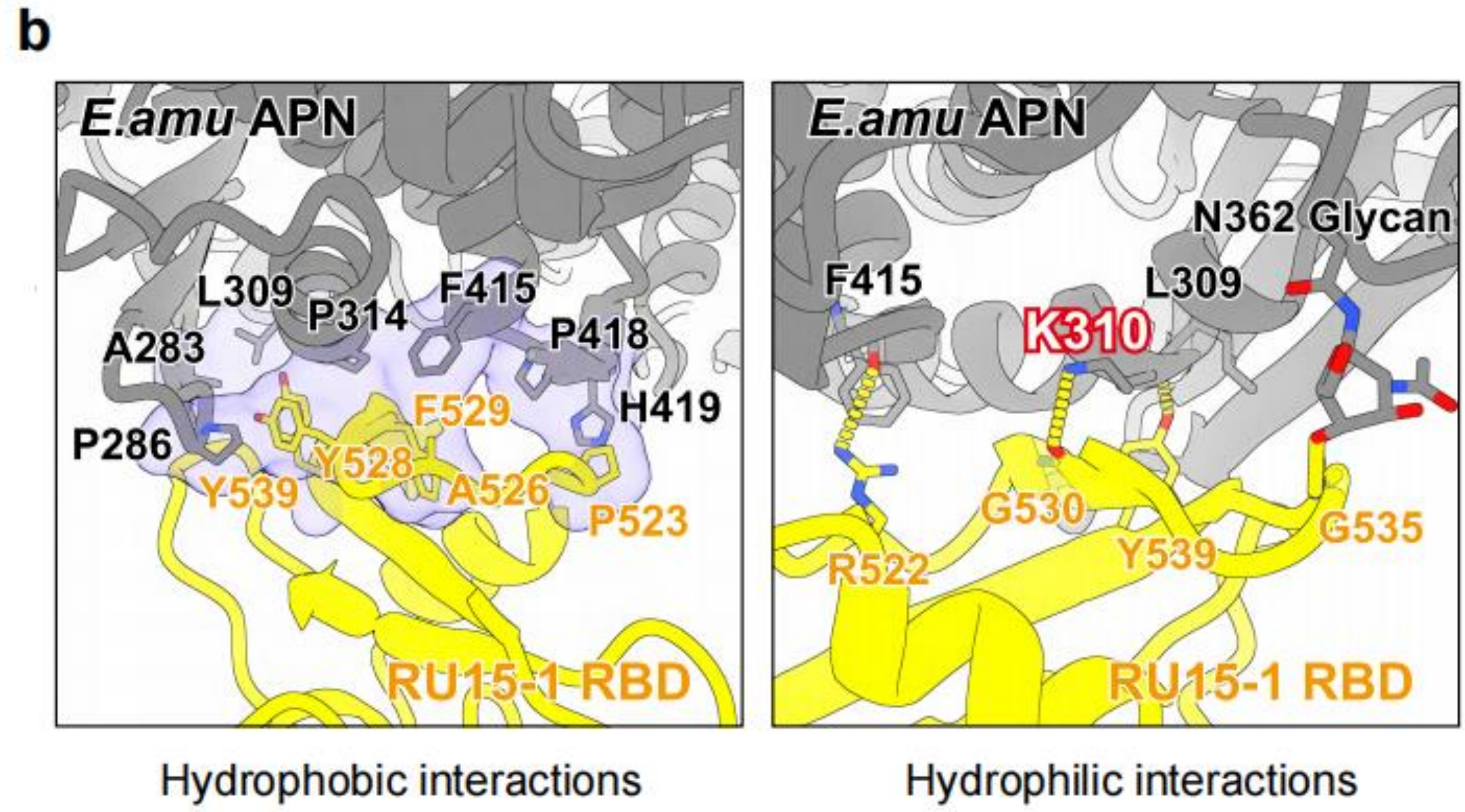

Fig. 4b Interaction details between RU15-1 RBD and E.amu APN. Structures are shown as cartoon representations with key residues rendered as sticks. The hydrophobic pocket and hydrogen bonds are shown as a blue shadow with semi-transparency and yellow dashed lines, respectively. The position (K310) that glycosylated in human APN is highlighted with red outline.

7. Figure 4f: For a clearer comparison of the receptor-binding interfaces, the RBDs in all complexes should be displayed at the same angle.

Response: Updated as suggested, thank you for the helpful suggestion.

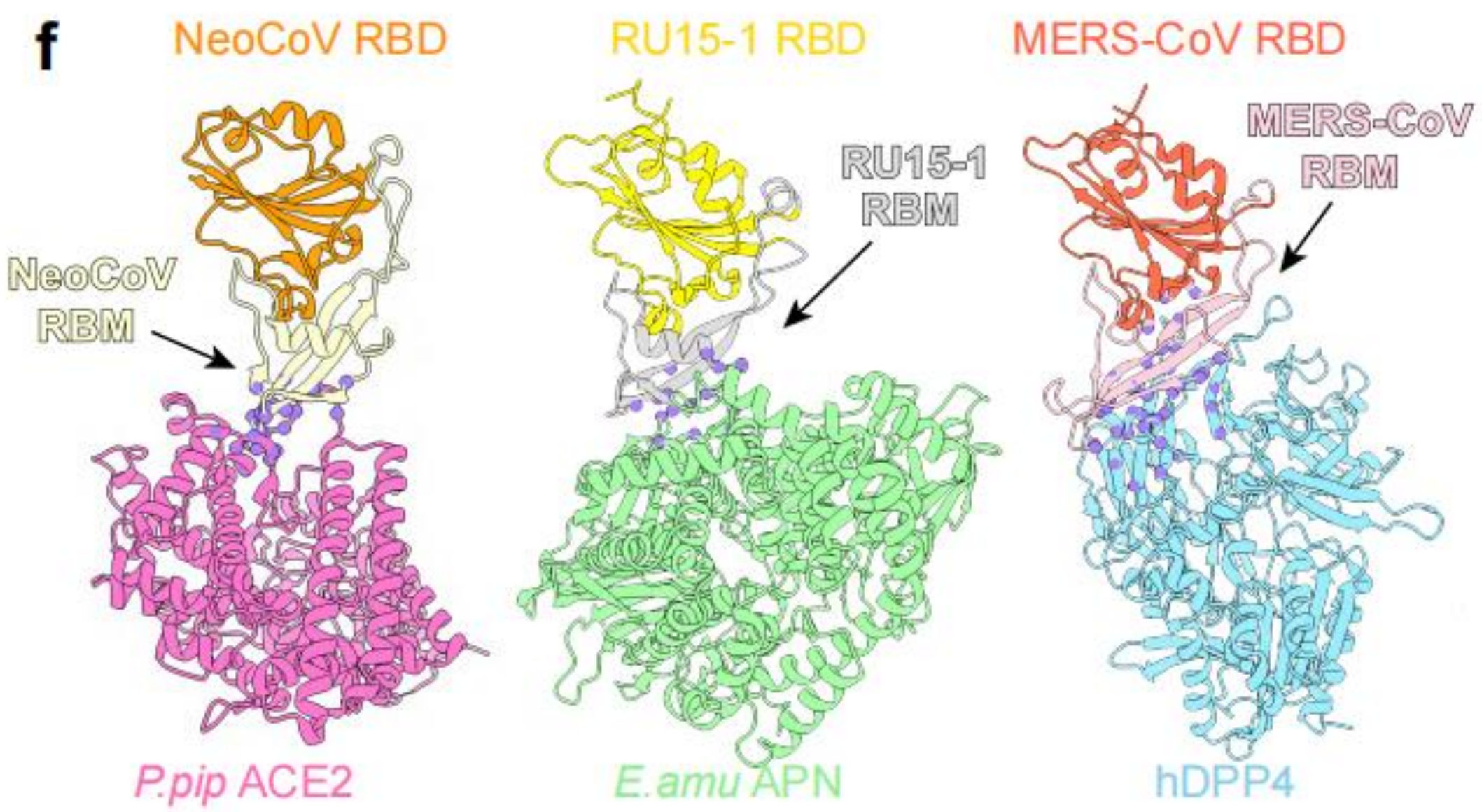

Fig. 4f Structural comparison of three merbecovirus binding modes with distinct receptors: NeoCoV–*P.pip* ACE2 (PDB: 7WPO), EriCoV–*E.amu* APN (PDB: 9W6D) and MERS-CoV–hDPP4 (PDB: 4KR0). Right: Zoomed-in view showing the superimposed RBMs from each virus. All structures are shown in cartoon representation. The P.pip ACE2, *E.amu* APN, and hDPP4 are colored hot pink, light green, and sky blue, respectively. The RBDs of NeoCoV, EriCoV, and MERS-CoV are colored dark orange, gold, and tomato, respectively, with their corresponding RBMs highlighted in wheat, silver, and light pink.

Point-by-Point Response letter for Nature Microbiology

Manuscript title: Convergent utilization of APN receptor by hedgehog merbecoviruses
Corresponding authors: **Huan Yan**, Xiangxi Wang, Yunlong Cao, Linlin Bao
Manuscript ID: NMICROBIOL-26010366C

Reviewer #1 (Remarks to the Author):

The authors have addressed my concerns

We thank the reviewer for the positive evaluation and are pleased that the concerns have been satisfactorily addressed.

Reviewer #2 (Remarks to the Author):

Dear authors,

I can only give back my sincere thanks for what is a very thorough follow-up and response to my feedback. This was already a very good manuscript and as a I highlighted, comprehensive and easy to follow; it is now even more so.

I think this manuscript can now stand very proud as a great example of one health research of infectious agents where often we don't have a full virus but equally conducting thorough and safe in vitro work are vital for risk assessment and biological understanding.

I strongly recommend this manuscript for acceptance.

We sincerely thank the reviewer for the positive assessment of our manuscript and for the constructive comments throughout the review process. We greatly appreciate your recognition of our work and your strong recommendation for acceptance.

Reviewer #3 (Remarks to the Author):

I would like to thank the authors for their detailed responses to my previous comments. I apologize for introducing these structure-related concerns at this stage, as they should have been raised during the initial review. However, upon further inspection of the deposited data, I believe the following points require clarification or correction to ensure the highest quality of the manuscript:

Based on the PDB validation reports, I have concerns regarding the following points:

1) PDB IDs: 9XW6 and 9XWA (Validation reports of PDB)

While the interface with the RBD is at a reasonable resolution and the model building appears generally plausible, the modeling of the RBD itself seems somewhat overstrained or physically unnatural (as reflected in the Validation Reports, pp. 39–40 (9XW6) and pp. 54–55 (9XWA), respectively).

2) PDB ID: 9XWM

There are several critical indicators of potential issues with data processing and model quality (Extended Data Fig. 12a and Validation report of PDB):

• Unnatural FSC Curve: The FSC curve exhibits an unnatural profile, dropping abruptly and unusually smoothly from a flat plateau.

• High Clashscore: The clashscore is exceptionally high (19), which raises concerns about the stereochemical quality of the model.

• Signs of Overfitting: The presence of radial map artifacts suggests potential overfitting, which is further supported by the notably low Q-scores in the outer regions of the molecule.

The authors might had better check DAQ sore of Prof. Kihara's laboratory of Purdue University (https://daqdb.kiharalab.org/) to evaluate the structures and should address these concerns or correct the models accordingly.

We thank Reviewer #3 for the careful re-inspection of the deposited structural data and for raising these important structure-validation concerns. We fully agree that the reliability of the cryo-EM maps, atomic models and validation statistics is essential for supporting the structural conclusions of this manuscript. In response to the reviewer's comments, we performed a comprehensive re-evaluation of the three structures specifically mentioned by the reviewer, including 9XW6, 9XWA and 9XWM.

1) PDB IDs: 9XW6 and 9XWA (Validation reports of PDB)

While the interface with the RBD is at a reasonable resolution and the model building appears generally plausible, the modeling of the RBD itself seems somewhat overstrained or physically unnatural (as reflected in the Validation Reports, pp. 39–40 (9XW6) and pp. 54–55 (9XWA), respectively).

Response:

We thank the reviewer for raising this concern. We re-examined the model quality of the RBD region in the EriAPN–RU15-1 RBD complex structures 9XW6 and 9XWA.

For 9XWA, the poor-fit fraction of the RBD chains was reduced from 57%/56% to 38%/37% (pp. 2), and the atom inclusion values of RBD chains C and D increased from 0.261/0.270 to 0.455/0.461 (pp. 57), respectively. These improvements address the map–model fit concerns reflected in pp. 54–55 of the original validation report.

For 9XW6, the original particles set contained both APN-only particles and RBD-bound APN particles, which likely contributed to the relatively weaker density of the RBD region and affected the local validation metrics of the RBD model.To address this issue, we performed supervised 3D classification in RELION to enrich the RBD-bound APN particles. This classification yielded a high-quality RBD-bound particle subset, accounting for approximately 35% of the particles, which was then used for reconstruction, model correction and real-space refinement. In the revised maps, the RBD density was improved.

We have re-submitted the revised maps and coordinates to the PDB and generated new PDB validation reports (27OA). The corresponding cryo-EM processing workflow, cryo-EM statistics table and PDB validation results have been updated in the revised manuscript. These revisions improve the model quality of the RBD region but do not alter the main structural interpretation or conclusions regarding the EriAPN–RU15-1 RBD complex.

The corresponding data have been uploaded to Google Drive at the following link:
https://drive.google.com/drive/folders/1rEycs6gWTRF3a7tJBqzTtjfuHdS2pmPI?usp=drive_link

2) PDB ID: 9XWM

There are several critical indicators of potential issues with data processing and model quality (Extended Data Fig. 12a and Validation report of PDB):

• Unnatural FSC Curve: The FSC curve exhibits an unnatural profile, dropping abruptly and unusually smoothly from a flat plateau.

Response:

We appreciate the reviewers' careful attention to the anomalous behavior of the FSC curve in the MO1 − 2Fab (PDB ID: 9XWM) reconstruction. In response, we reprocessed the MO1 − 2Fab dataset using CryoSPARC and repeated both homogeneous and local refinements.

In the repeated homogeneous refinement, the FSC curve exhibited a gradual decay trend without a significant plateau, consistent with the FSC curve obtained from our initial homogeneous refinement. In contrast, after applying a mask and performing repeated local refinement, the overall map resolution improved, but the FSC curve showed a similar short plateau region in the medium resolution range, followed by a gradual decline *(Figure 1)*. Notably, the masks used in the two independent local refinements were not identical: the first mask was relatively tighter with a soft padding width of 5 pixels; the second mask was relatively looser with a soft padding width of 12 pixels. *(Figure 2)* This suggests that the shape of the FSC curve may be related to the influence of the mask on the FSC calculation. Similar FSC curves exhibiting a short plateau followed by a decline have been observed in published cryo-electron microscopy workflows *(Figure 3)*.

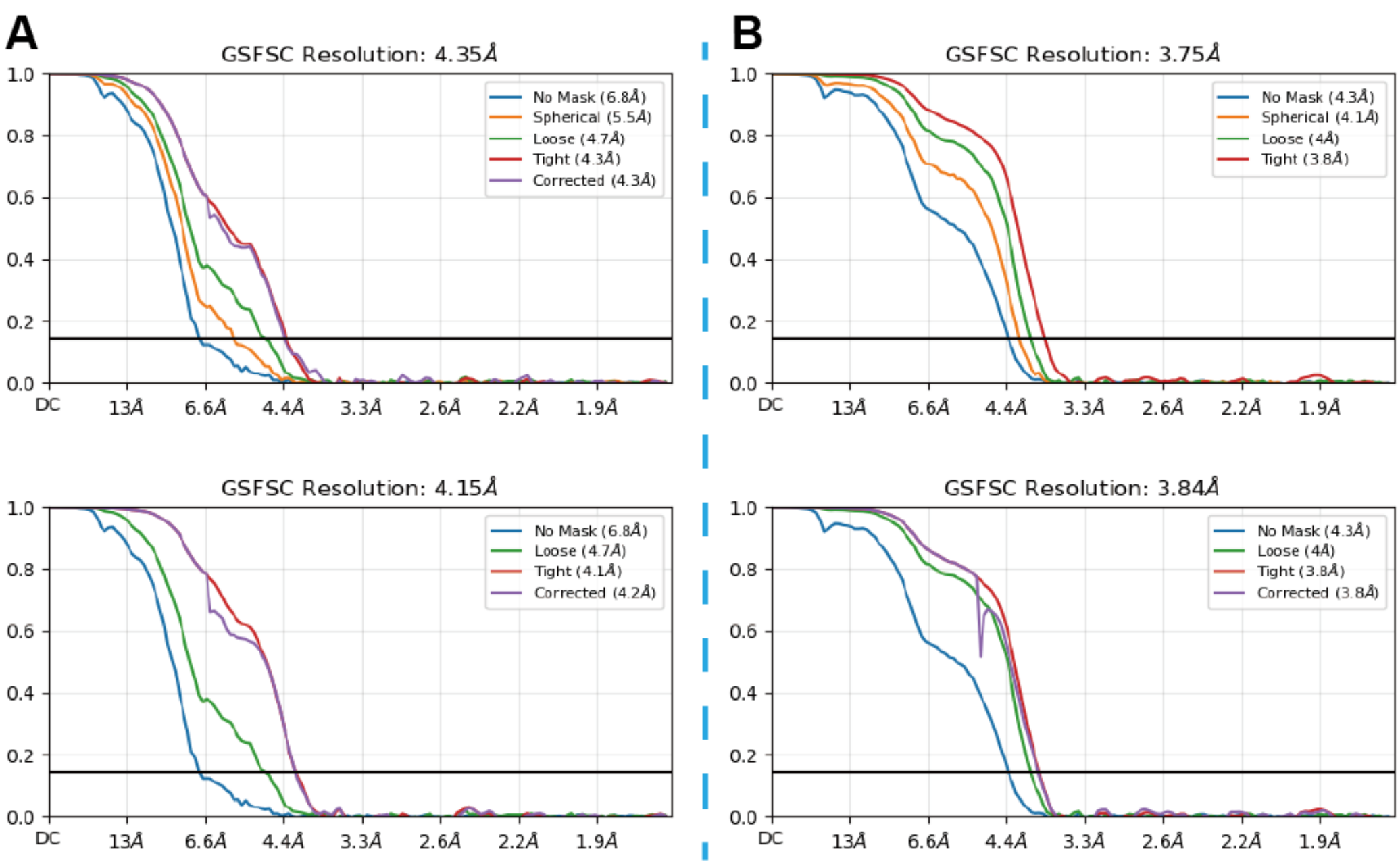

Figure 1 | Gold-standard FSC curves after repeated refinement of the MO1－2Fab reconstruction. Gold-standard FSC (GSFSC) curves were calculated after repeated homogeneous refinement (**A**) and local refinement (**B**) of the MO1－2Fab reconstruction. The upper panels show the FSC curves automatically generated by cryoSPARC, whereas the lower panels show the FSC curves recalculated after FSC-mask auto-tightening.

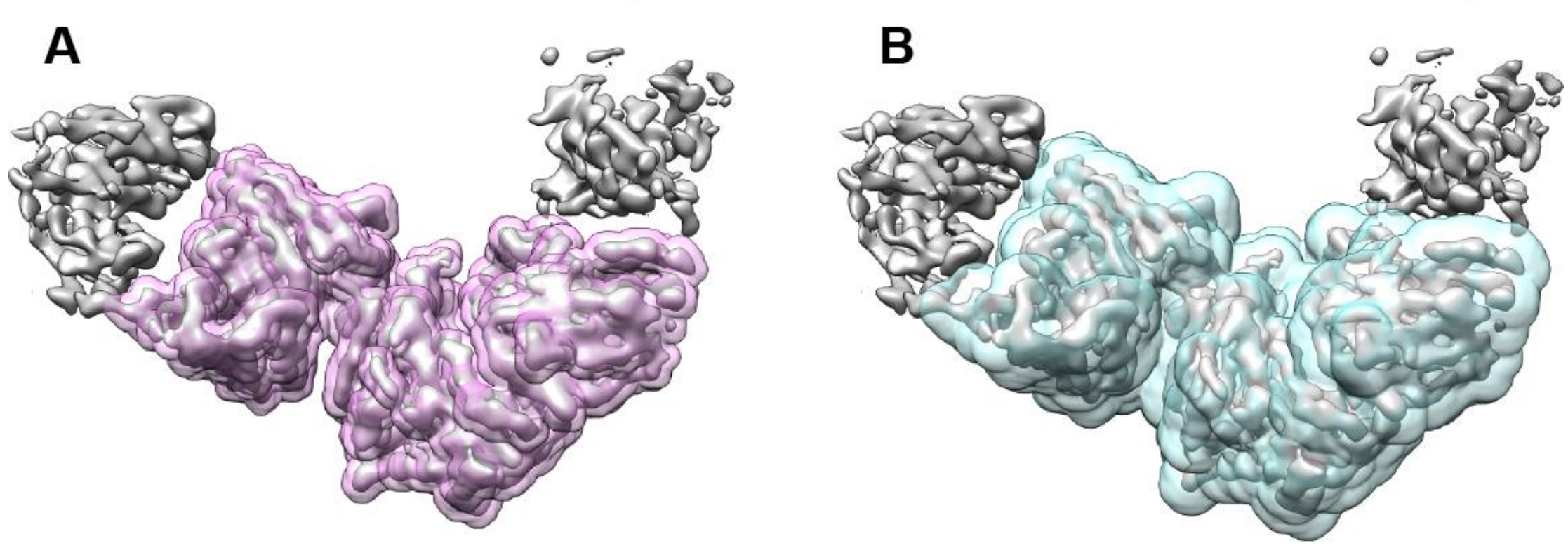

Figure 2 | Different masks used for the two rounds of local refinement. Input mask.mrc files used for the two rounds of local refinement of the MO1–2Fab reconstruction are shown. **A**, the

mask used in the first round of local refinement, with a soft padding width of 5 pixels. **B**, the mask used in the second round of local refinement, with a soft padding width of 12 pixels. The gray density represents regions outside or not fully covered by the refinement mask, whereas the semi-transparent colored density indicates the masked region used for local refinement.

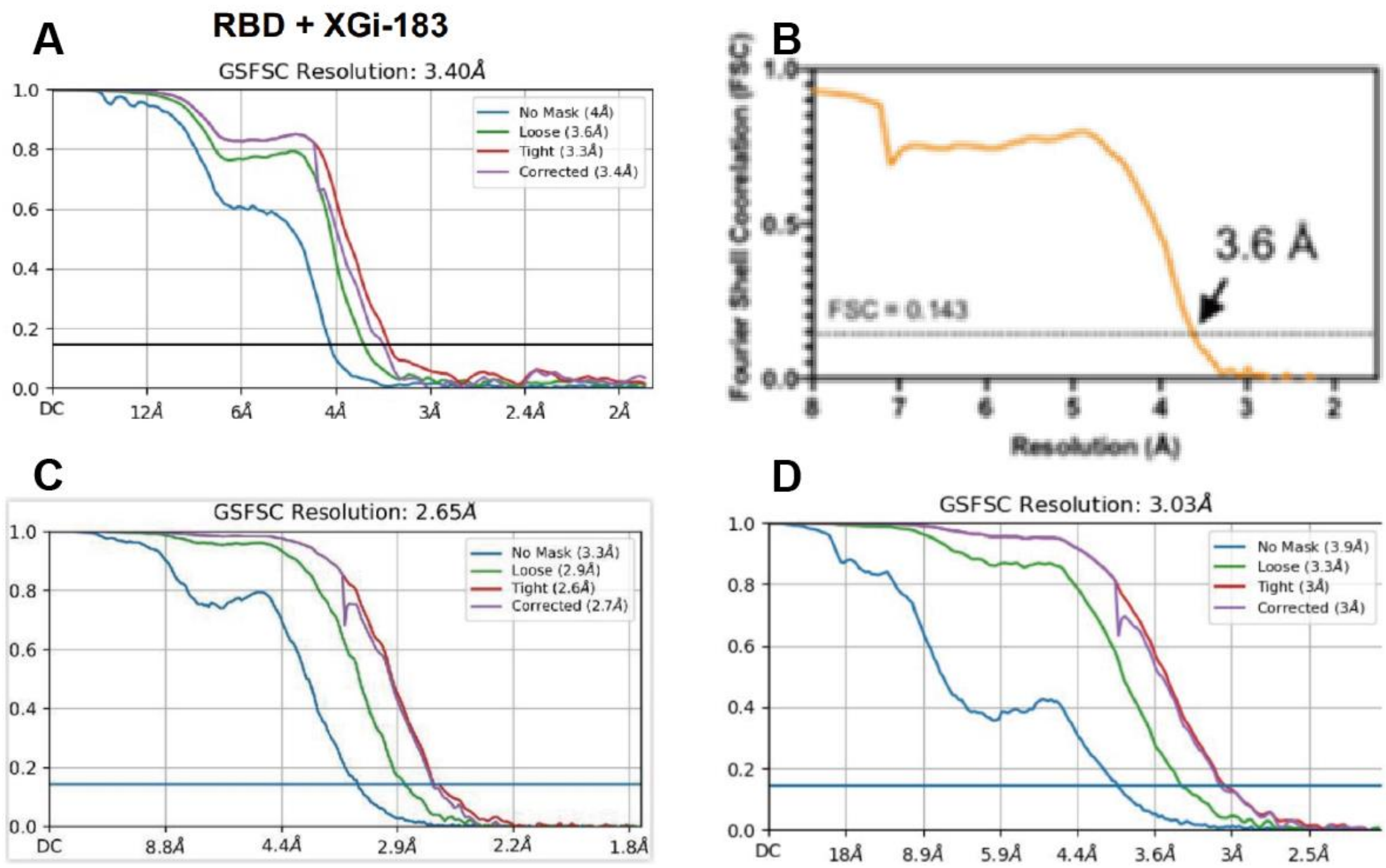

Figure 3 | Representative FSC curves reported in previously published cryo-EM studies. FSC curves reported in cryo-EM data-processing workflows from previously published studies are shown to illustrate similar FSC curve profiles observed across different samples and refinement procedures. **A**, gold-standard FSC curve generated during cryo-EM data collection and processing of the EG.5.1 S/XGi-183 complex[1]. **B**, FSC curve reported in the cryo-EM data-processing workflow for SARS-CoV-2 S in complex with RBD-chAb-25[2]. **C**, gold-standard FSC curve generated during cryo-EM data processing of the Prototype S-L4.56-L5.34 complex[3]. **D**, gold-standard FSC curve generated during cryo-EM single-particle analysis of the apo Omicron spike[4]. Panels **A–C** correspond to different antigen–Fab/antibody complexes, whereas panel D represents an antibody-free apo spike structure.

We further examined half-maps A and B, automatically generated by CryoSPARC after local refinement. The two half-maps are not identical, indicating that the plateau-like FSC curves were not caused by missubmission or reuse of the same half-map. Furthermore, these half-maps are direct outputs of the CryoSPARC refinement, not generated by sharpened maps, locally filtered maps, low-pass-filtered maps, or other post-processing final maps.

One possible explanation is that, in specific datasets, the mask in local refinement removes significant solvent region noise, increasing the relative contribution of the intermediate-resolution signal in the complex; simultaneously, the same mask applied to two half-maps may introduce mask-induced correlation [5, 6].

To avoid overly optimistic estimates of resolution, we adopted a more conservative resolution estimate from the reprocessed local refinement and updated the resolution of the MO1－2Fab complex structure to 3.75 Å. We also updated Extended Data Fig. 12a accordingly, demonstrating the revised cryo-electron microscopy data processing workflow and FSC curves, and revised relevant information in the text and structure statistics tables. These revisions do not change the structural interpretation of the MO1－2Fab complex, but provide a more conservative and transparent assessment of spectral resolution.

Furthermore, we updated the submission via PDB OneDep and generated a new wwPDB validation report for the revised model. The updated homogeneous refinement and local refinement half-maps, sharpened maps, atomic model, and validation reports are provided as review documents or uploaded to the following link for independent review by reviewers. https://drive.google.com/drive/folders/1rEycs6gWTRF3a7tJBqzTtjfuHdS2pmPI?usp=drive_link

• High Clashscore: The clashscore is exceptionally high (19), which raises concerns about the stereochemical quality of the model.

Response:

We thank the reviewer for pointing out this issue. We agree that the originally deposited 9XWM model had a relatively high clashscore, indicating that the local stereochemical quality of the model required further improvement. To address this concern, we re-examined and rebuilt the atomic model using the map obtained from the second round of local refinement. Specifically, we first re-fitted the PDB model into the updated cryo-EM map, manually inspected and corrected the major close contacts and local geometric outliers highlighted in the validation report using Coot, and then performed multiple rounds of real-space refinement using Phenix real-space refinement (Cryo-EM).

After iterative model rebuilding and real-space refinement, the stereochemical quality of the model was substantially improved. The Phenix/MolProbity clashscore was reduced to 9.73. The corresponding structural statistics have been updated in the relevant table, and a new wwPDB validation report has been generated (Clashscore:10). These corrections improve the local geometry of the model but do not alter the overall structural interpretation or main conclusions regarding the MO1–2Fab complex.

• Signs of Overfitting: The presence of radial map artifacts suggests potential overfitting, which is further supported by the notably low Q-scores in the outer regions of the molecule.

Response:

We thank the reviewer for pointing out this concern. We carefully re-examined the features in the 9XWM map and model that could be interpreted as potential overfitting, including the radial map artifacts and the relatively low Q-scores in the peripheral regions of the molecule. We reasoned that the local mask used in the original local refinement was relatively tight, with a soft padding width of 5 pixels, which may have contributed to mask-related artifacts and reduced the apparent local map–model support in weaker peripheral density regions.

To address this issue, we regenerated a looser local-refinement mask by increasing the soft padding width to 12 pixels and repeated the local refinement using this revised mask. Compared with the original reconstruction, the revised map showed reduced radial artifacts and improved local density continuity and map–model agreement in the peripheral regions. We then re-examined, manually corrected, and real-space refined the atomic model against the updated map, followed by generation of a new PDB validation report. The revised validation results showed improved Q-scores in the peripheral regions, indicating improved agreement between the model and the local density.

The updated cryo-EM processing workflow, FSC curves, structural statistics, and validation results have been incorporated into Extended Data Fig. 9a and the Supplementary Table X. These revisions improve the local quality assessment of the 9XWM map and model, but do not alter the overall structural interpretation or the main conclusions regarding the MO1–2Fab complex.

The authors might had better check DAQ sore of Prof. Kihara's laboratory of Purdue University (https://daqdb.kiharalab.org/) to evaluate the structures and should address these concerns or correct the models accordingly.

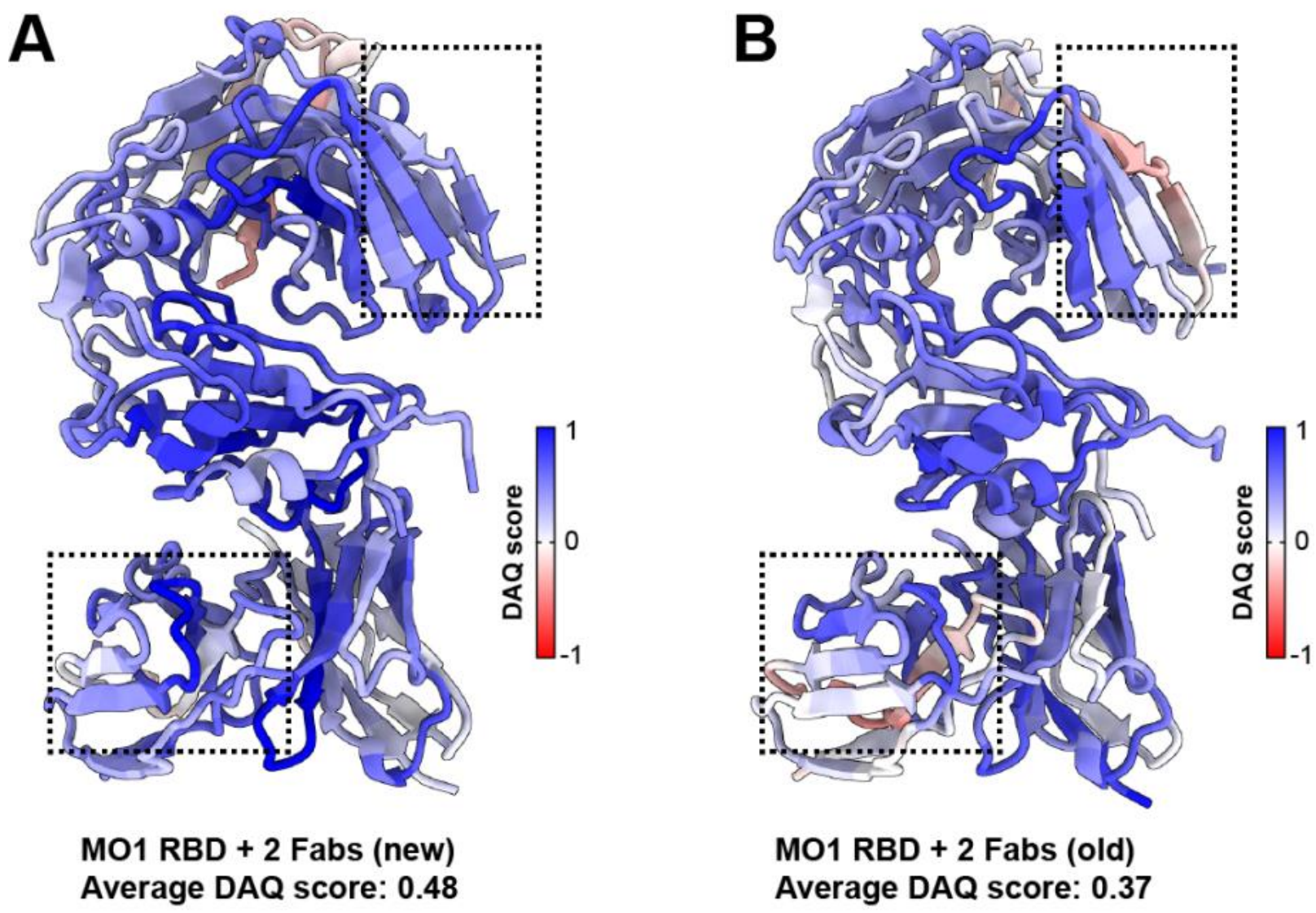

Figure 4 | Comparison of DAQ scores between the revised and original MO1–2Fabs complex models. (**A**) The revised MO1–2Fabs complex model coloured by residue-wise DAQ score, with an average DAQ score of 0.48. (**B**) The original MO1–2Fabs complex model coloured by residue-wise DAQ score, with an average DAQ score of 0.37. The colour gradient indicates the degree of local model–map agreement, with blue representing higher DAQ scores and red representing lower DAQ scores. Dashed boxes highlight representative peripheral regions showing improved DAQ scores in the revised model.

Response:

We thank the reviewer for this helpful suggestion. Following the reviewer's recommendation, we further evaluated the MO1–2Fab structure using the DAQ-score method developed by the Kihara

laboratory, in order to independently assess the local agreement between the atomic model and the cryo-EM map.

We performed DAQ-score analysis for both the original and revised MO1–2Fab models. After repeated local refinement, manual model correction and real-space refinement, the average DAQ score of the MO1–2Fab complex increased from 0.37 in the original model to 0.48 in the revised model. The residue-wise DAQ-score colouring further showed that the revised model contains fewer low-scoring regions in the molecular periphery, particularly in parts of the peripheral Fab regions, indicating improved local model–map agreement in these areas. In addition, we have cited the relevant DAQ-score references in method section of the revised manuscript to provide the methodological basis for this analysis.

Reference：
[1] Xie M, Qiu Y, Zhao X, et al. Orphan broadly RBD-binding antibodies annotate three remaining conserved RBD epitopes along SARS-CoV-2 evolution[J]. Nature Communications, 2025, 16(1): 10566.
[2] Su S C, Yang T J, Yu P Y, et al. Structure-guided antibody cocktail for prevention and treatment of COVID-19[J]. PLoS Pathogens, 2021, 17(10): e1009704.
[3] Guo S, Zheng Y, Gao Z, et al. Dosing interval regimen shapes potency and breadth of antibody repertoire after vaccination of SARS-CoV-2 RBD protein subunit vaccine[J]. Cell Discovery, 2023, 9(1): 79.
[4] Zhao Z, Zhou J, Tian M, et al. Omicron SARS-CoV-2 mutations stabilize spike up-RBD conformation and lead to a non-RBM-binding monoclonal antibody escape[J]. Nature communications, 2022, 13(1): 4958.
[5] Penczek P A. Reliable cryo-EM resolution estimation with modified Fourier shell correlation[J]. IUCrJ, 2020, 7(6): 995-1008.
[6] Chen S, McMullan G, Faruqi A R, et al. High-resolution noise substitution to measure overfitting and validate resolution in 3D structure determination by single particle electron cryomicroscopy[J]. Ultramicroscopy, 2013, 135: 24-35.

Point-by-Point Response letter for Nature Microbiology

Manuscript title: Convergent utilization of APN receptor by hedgehog merbecoviruses

Corresponding authors: Huan Yan, Xiangxi Wang, Yunlong Cao, Linlin Bao

Manuscript ID: NMICROBIOL-26010366D

Reviewer #3 (Remarks to the Author):

I would like to thank the authors for their detailed response and for providing the updated information and models regarding their cryo-EM analysis.

General Comments: The revised models and statistics look improved; the global FSC resolution has been adjusted to a more realistic value (from 2.0 Å to over 3.x Å), and the density maps for the RBD-receptor binding interface—the primary focus of this study—look reasonable.

Response: *We sincerely thank the reviewers for their careful evaluation of our cryo-EM data and for their constructive comments and suggestions. We have revised them accordingly.*

However, there are still several noticeable flaws in the detailed model building. At the current resolution, discussing specific side-chain orientations and detailed interactions is at the baseline threshold of feasibility. Since it is difficult to verify every single detail of the coordinates, the authors must thoroughly re-examine and further refine the models under their own responsibility.

Response: *We thank the reviewer for this important and constructive comment. Following the reviewer's suggestion, we thoroughly re-examined all cryo-EM density maps and corresponding atomic models included in this study. During this process, we carefully inspected the local map quality, side-chain fitting, glycan modeling, loop regions, and model–map agreement. We further refined the models where appropriate and removed or adjusted coordinates that were not sufficiently supported by the density, including poorly resolved loops, unsupported glycan rings, and questionable side-chain rotamers (more details in reviewer Figure 2).*

The five cryo-EM structures reported in this study, including the E.amu APN–RU15-1 RBD complex, E.amu APN–RU15-1 RBD (focused reconstruction), RU15-1 Spike, RU15-1 RBD + 2Fabs, and MO1 RBD + 2Fabs, were resolved at 3.41 Å, 3.55 Å, 3.34 Å, 3.84 Å, and 3.79 Å (updated data, Figure 4), respectively. The quality of the ***E.amu APN–RU15-1 RBD complex*** *and the corresponding* ***focused reconstruction*** *supports the sidechain rotamer and interaction interpretation of the binding interface (****Figure R1a****). For the* ***RU15-1 RBD + 2Fabs*** *and* ***MO1 RBD + 2Fabs*** *structures, which were resolved at relatively lower resolutions of* ***3.84 Å*** *and* ***3.79 Å****, respectively, we agree that interpretation of specific side-chain orientations and atomic interactions requires additional caution. Therefore, we have carefully checked the local densities (****Figure R1b and R1c****) and revised the description of side-chain-level interactions where necessary to avoid over-interpretation.*

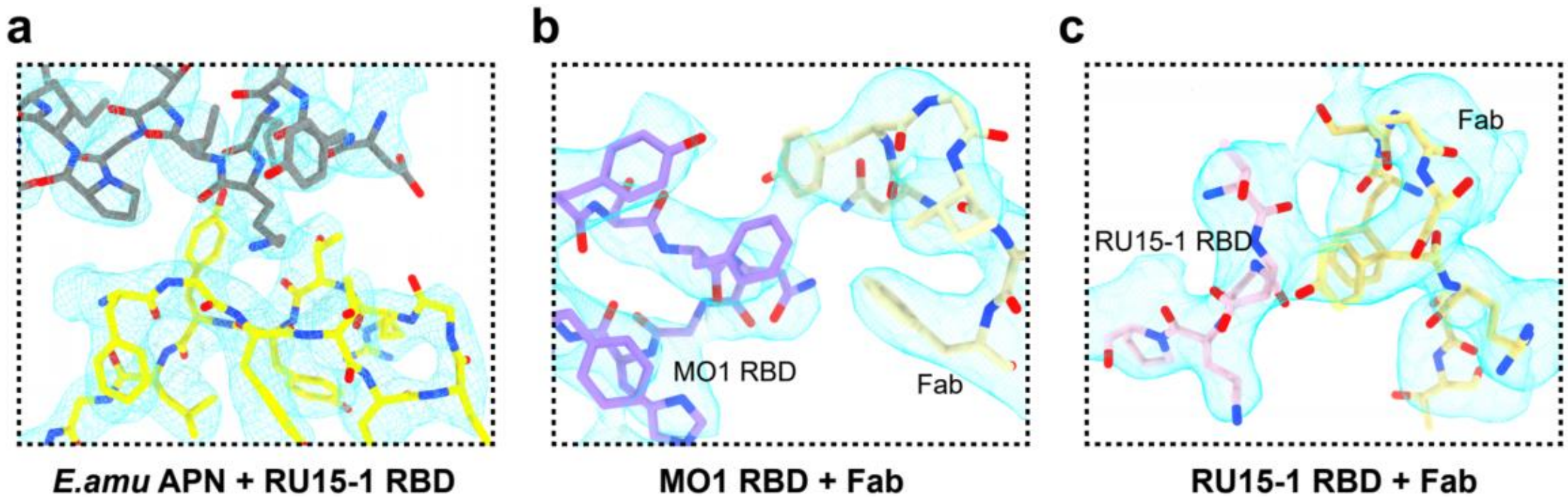

Figure R1. Map densities of RBD and receptor or antibodies

Revised manuscript:

To define the neutralization mechanisms, we structurally characterized BD23-0177/BD23-0152 and BD23-0027/BD23-0087 as representative RU15-1 and MO1 mAbs, with reconstructions determined at 3.84 Å and 3.79 Å, respectively (Extended Data Fig. 9a,b). BD23-0177 recognizes the RU15-1 RBM tip, with residues T537, Y539, R494, and D525 positioned near the antibody interface and potentially contributing to polar interactions….

The following specific points regarding model quality and map processing should be addressed: Glycan and Model Quality: There are prominent discrepancies in the model building (please see the examples, the attached pdf file of maps). For example, specifically, there are regions where clear glycan densities are visible but no models have been built. Conversely, in other areas, models have been placed where no corresponding density exists in the map. The authors need to carefully inspect the entire structure and correct these modeling errors.

Response: *We thank the reviewer for carefully examining the model quality and for pointing out these important issues regarding glycan modeling and local coordinate–map agreement.*

Following the reviewer's suggestion, we carefully re-inspected the cryo-EM density maps and atomic coordinates for all structures reported in this study. To ensure that the models are built conservatively and faithfully according to the experimental density, we made the following corrections.

*First, for the **E.amu APN–RU15-1 RBD complex** and the **focused reconstruction of the E.amu APN–RU15-1 RBD**, we re-evaluated all modeled N-linked glycans on both APN and RU15-1 RBD. The number of modeled sugar rings at each N-glycosylation site was adjusted according to the corresponding density, as summarized in the **Table R1** and **Figure R2**. In addition, we removed the coordinates of the RU15-1 RBD loop comprising residues **563–568**, for which no clear density was observed in the APN–RU15-1 RBD complex. We also corrected the unsupported rotamer of **Y564 in chain A** of the MO1+2Fabs structure (**Figure R2**).*

Reviewer Table R1: The number of modeled sugar rings at each N-glycosylation site

	E.amu APN										RU15-1 RBD			
N-glycosylation site	100	122	225	324	362	565	595	618	732	811	387	399	476	482
Number of sugar rings modeled	2	1	2	1	2	0	2	2	1	1	1	0	0	0

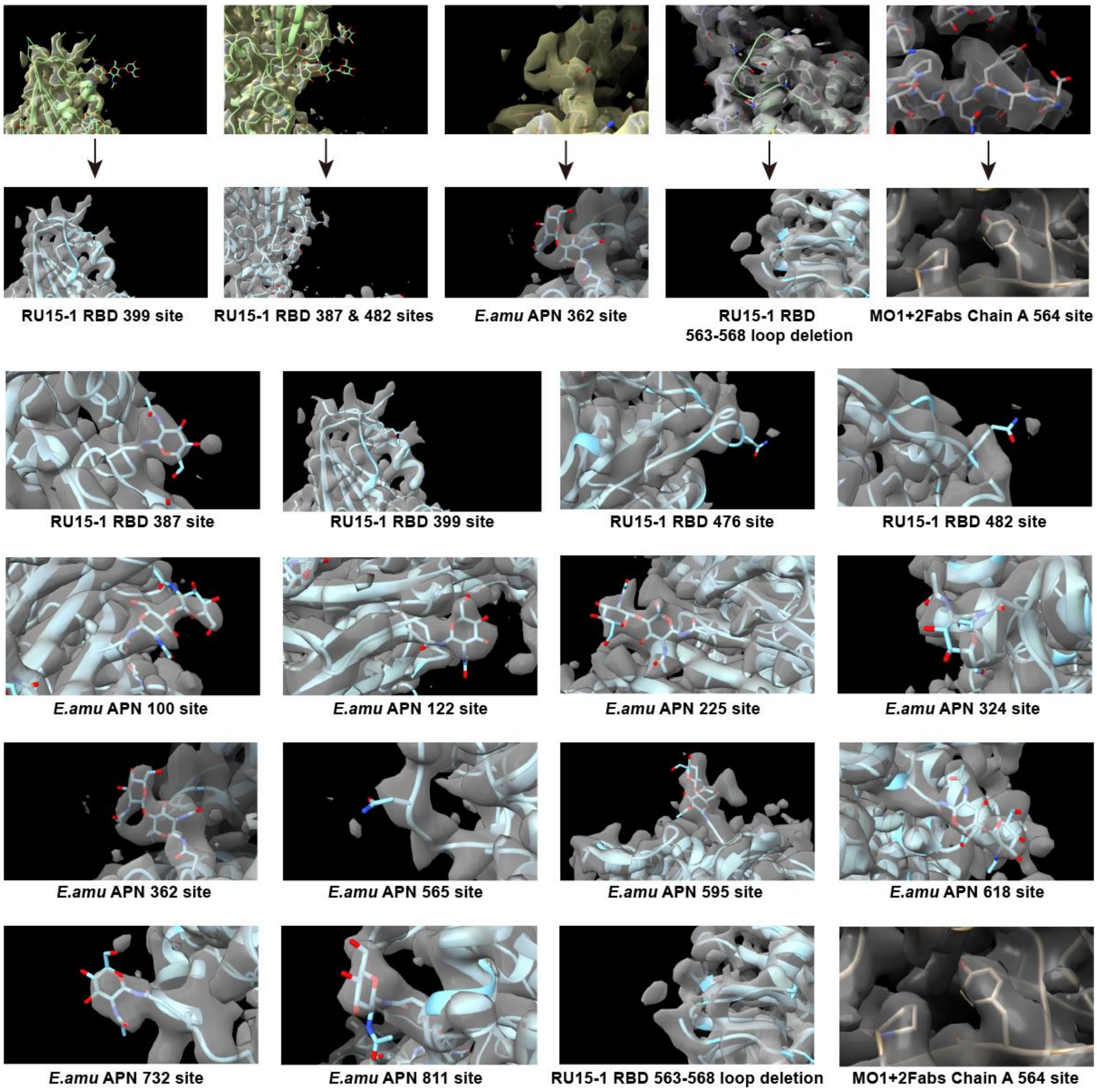

Figure R2. Map densities of typical N-glycosylation sites and other important areas

*Second, we comprehensively re-examined all other PDB models and maps included in this study. In the RU15-1 Spike structure, we removed glycan rings lacking supporting density at several N-glycosylation sites, including **N126, N152, N202, N233, N730, N750, N846, N1134, and N1185**. We also removed poorly resolved/disordered loop regions, including residues **575–578** and **852–860**, where the density was insufficient to support reliable model building.*

We believe these revisions have improved the agreement between the atomic models and the cryo-EM density maps. The revised coordinates and validation statistics have been updated accordingly, and the cryo-EM data collection, refinement, and validation table in the manuscript has also been updated.

Map Sharpening and B-factors: All submitted maps appear to be sharpened, and they show signs of over-sharpening (e.g., discontinuous/broken densities). The authors should inspect the unsharpened maps for verification. Furthermore, rather than relying solely on the default sharpened maps automatically generated by the CryoSPARC refinement job, it is highly recommended to run independent sharpening jobs. The authors should test multiple B-factor values to find the optimal

sharpening level that balances detail and continuity.

Response: We thank the reviewer for this helpful suggestion. We agree that inappropriate sharpening may introduce discontinuous or broken densities and may affect model interpretation. Therefore, we carefully re-evaluated the sharpening procedure for all cryo-EM maps associated with this study.

*Specifically, for the five cryo-EM density maps reported in this work, we performed additional independent sharpening tests rather than relying solely on the default sharpened maps generated by the CryoSPARC refinement jobs. For each map, we compared maps generated under several sharpening conditions, including no additional B-factor sharpening, the CryoSPARC-estimated B factor, and one or two intermediate user-defined B-factor values between these conditions. In total, 3–4 sharpening conditions were examined for each map. We assessed the resulting maps by comparing both high-frequency structural details and the continuity of local densities, particularly in glycan regions, loop regions, and receptor/RBD interface regions. Representative comparisons are shown in **Figure R3**.*

*For the **E.amu APN–RU15-1 RBD focused reconstruction**, **RU15-1 Spike**, **RU15-1 RBD + 2Fabs**, and **MO1 RBD + 2Fabs** maps (**Figure R3a, 3b, 3d and 3e**), the maps generated using the CryoSPARC-estimated B factors preserved density continuity while maintaining sufficient high-frequency details. Therefore, we retained these maps as the final maps.*

*For the **E.amu APN–RU15-1 RBD complex** map (**Figure R3c**), however, the map sharpened using the CryoSPARC-estimated B factor showed fragmented densities near the terminal region of the RBD. To better balance high-frequency details and density continuity, we selected a user-defined B factor of **−80 Å²** for the final sharpened map. This revised map has been used for model inspection and has been uploaded to the EMDB database.*

*It is also worth noting that, for determining the **E.amu APN–RU15-1 RBD complex structure**, focused reconstruction effectively improved the continuity of the density around the terminal region of the RBD. This was one of the reasons why we adopted focused reconstruction in the final stage of 3D reconstruction and model interpretation.*

Accordingly, we have updated the submitted cryo-EM map files where appropriate and revised the cryo-EM data collection, refinement, and validation table in the manuscript.

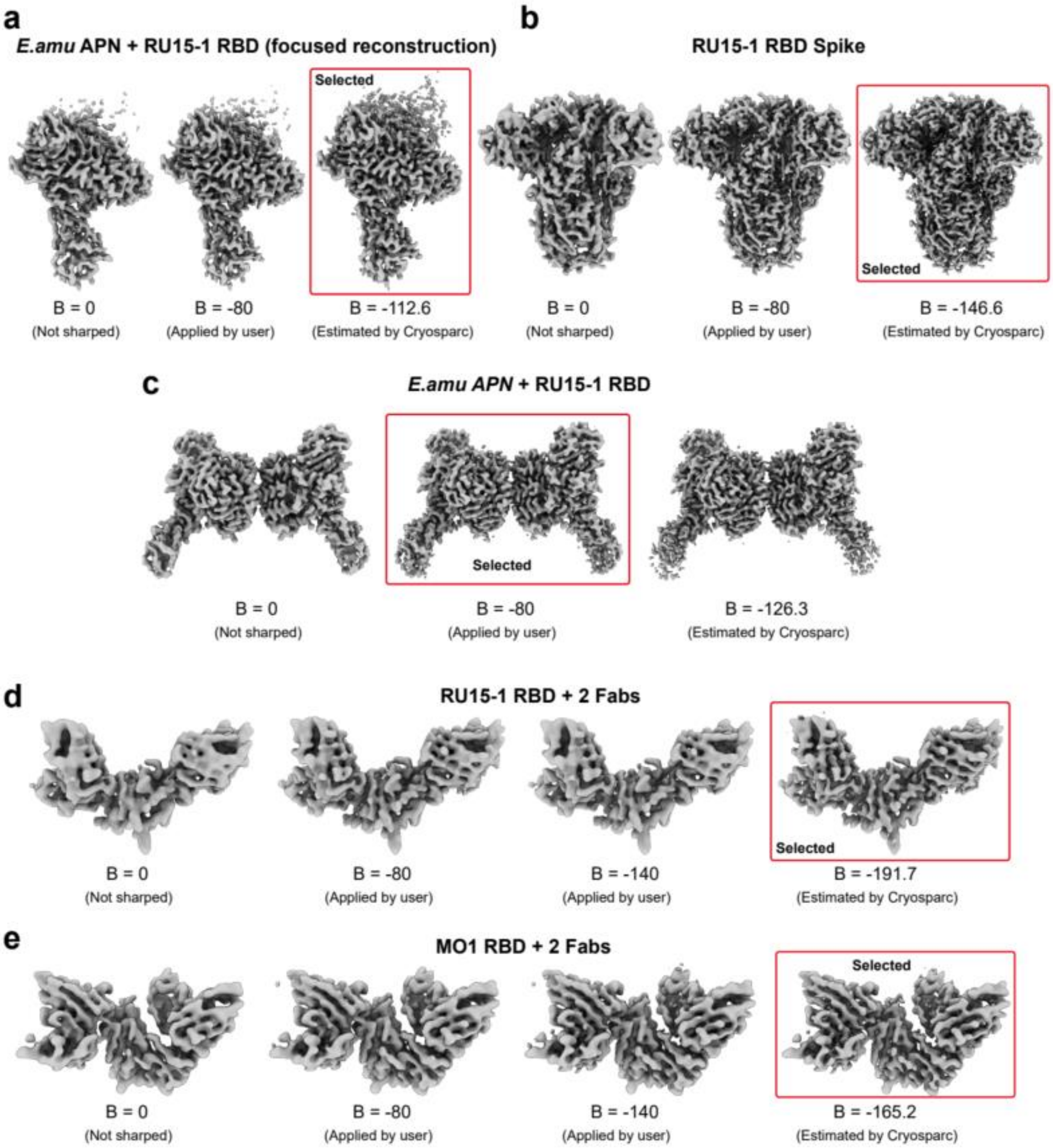

Reviewer Figure R3. Cryo-EM structures generated under different B factors.
Negative B-factor values indicate map sharpening. The maps were generated under different B-factor sharpening conditions to compare the balance between high-frequency details and density continuity. Because the density value distributions vary substantially among maps sharpened with different B factors, the maps are displayed at individually adjusted contour levels. For each map, the contour level was chosen to clearly show the overall structure while minimizing background noise. In ***panel a****, the fragmented densities shown in the three enlarged views correspond to the non-focused APN–RBD monomer after focused reconstruction and represent residual noise rather than interpretable structural density.*

Mask Soft Padding Width: The mask soft padding used in the analysis should be re-evaluated. According to the official CryoSPARC guide, a soft padding width of 5×(current resolution/pixel size) is generally recommended. While this width may be narrowed for smaller targets, the authors

should first test a width of approximately 23 Å derived from this standard formula to ensure appropriate masking and prevent potential artifacts. (Reference: https://guide.cryosparc.com/processing-data/tutorials-and-case-studies/mask-selection-and-generation-in-ucsf-chimera)

Response: We thank the reviewer for this helpful suggestion regarding the mask soft padding width. Following the reviewer's recommendation, we re-evaluated the mask soft padding used in the refinement.
*According to the CryoSPARC guideline, the recommended soft padding width can be estimated as **5 × (current resolution / pixel size)**. For this reconstruction, the reported resolution is **3.75 Å** and the pixel size is **0.82 Å/pixel**, giving an estimated padding width of approximately **23 pixels**:*

$$5 \times 3.75 / 0.82 = 22.9 \text{ pixels}$$

*We therefore tested a soft padding width of **23 pixels**. The resulting reconstruction reached a global resolution of **3.79 Å**, which is only slightly different from the previous reconstruction obtained using a **12-pixel** soft padding width, which reached **3.75 Å**. Importantly, comparison of the two maps showed no substantial difference in the overall density quality or local structural interpretation (**Figure R4**).*
*To ensure consistency with the recommended masking procedure and to minimize potential masking-related artifacts, we have updated the corresponding cryo-EM map generated using the **23-pixel** soft padding width. The revised map, FSC curve, Local resolution map and the updated cryo-EM refinement and validation statistics have been included in the revised submission.*

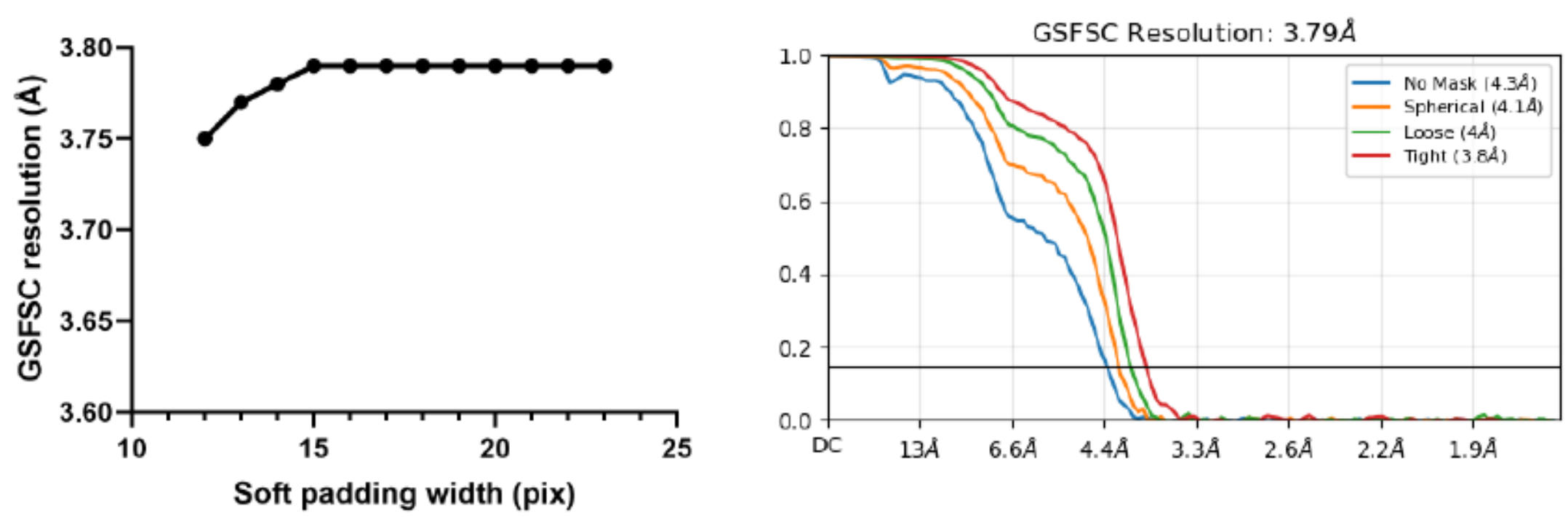

Figure R4. The relation between resolution and soft padding width and FSC curve of cryo-EM map generated using the 23-pixel soft padding width.

The following references have been added to the method section:

1. Rosenthal, P. B. & Henderson, R. Optimal determination of particle orientation, absolute hand, and contrast loss in single-particle electron cryomicroscopy. J Mol Biol 333, 721–745 (2003).
2. Chen, S. et al. High-resolution noise substitution to measure overfitting and validate resolution in 3D structure determination by single particle electron cryomicroscopy. Ultramicroscopy 135, 24–35 (2013).
3. Falkner, B. & Schröder, G. F. Cross-validation in cryo-EM-based structural modeling. Proc Natl Acad Sci U S A 110, 8930–8935 (2013).

The revised structural data, including the updated maps and PDB coordinate files, have been uploaded to Google Drive and can be accessed via the link below:
https://drive.google.com/drive/folders/1uZe_FI-Pm6zYkqg9c6Suhh45harRDaw4?usp=drive_link

Liu, et al., Nature Microbiology manuscript ID: NMICROBIOL-26010366D
Revised title "***Convergent use of Aminopeptidase N receptor by hedgehog merbecoviruses***"

Point-by-point responses to Reviews' comments:

Reviewer #3 (Remarks to the Author):

Thank you very much for your hard work and detailed response to address all the comments. I highly appreciate your thorough efforts in improving the manuscript and the models.

Since all the points have been successfully addressed, please make sure to deposit and update the refined models in the PDB accordingly.

Response:
We sincerely thank the reviewer for the positive assessment and constructive comments. The refined models have been deposited and updated in the PDB accordingly, and the updated PDB validation reports have been provided.
